# Stimuli-Responsive Thiele’s Hydrocarbon Derivatives: Potential Inversion, Strong Electronic Coupling, and Influence of Brønsted/Lewis Acids and Bases

**DOI:** 10.1021/jacsau.5c01512

**Published:** 2026-02-16

**Authors:** Alok Mahata, Nicolás. I. Neuman, Manuel Pech, Jonas Schmidt, Luis I. Domenianni, Peter Vöhringer, Biprajit Sarkar

**Affiliations:** † Institut für Anorganische Chemie, Universität Stuttgart Pfaffenwaldring 55, Stuttgart 70569, Germany; ‡ Institut für Chemie und Biochemie, Anorganische Chemie, Freie Universität Berlin, Fabeckstraße 34-36, Berlin 14195, Germany; § Instituto de Desarrollo Tecnológico para la Industria Química, Colectora Ruta Nacional 168, Km 0, Paraje El Pozo, Santa Fe S3000ZAA, Argentina; ∥ Clausius-Institut für Physikalische und Theoretische Chemie, Rheinische Friedrich-Wilhelms-Universität Bonn,Wegelerstraße 12, Bonn 53115, Germany

**Keywords:** diradicaloid, Thiele hydrocarbon, stimuli response, potential inversion, electronic coupling

## Abstract

Stimuli-responsive diradicaloid systems often display fascinating and tunable electrochemical, optical, and magnetic properties. Herein, we present the design and synthesis of a series of nitrogen-containing Thiele’s hydrocarbon derivatives (**2** ^**Ph**^, **2** ^**Py**^, and **2** ^**Pz**^), based on phenyl, pyridinyl, and pyrazinyl spacers, with tunable electrochemical, electronic, and optical properties. By systematically increasing the number of nitrogen atoms in the molecular backbone, we observed consistent trends in both their absorption and their redox properties. A detailed investigation on the different oxidation states of **2** ^**Ph**^, which displays potential inversion, was also performed, including the intermediate radical cationic state (**2** ^**Ph**^ **RC**). This intermediate represents an intriguing example of a mixed-valent species whose thermodynamic stability, as calculated by the comproportionation constant (*K*_c_), is diminishingly small, whereas the electronic coupling is strong, making it a borderline class II–III mixed-valence compound. External modulation through coordination with Brønsted and Lewis acids reversibly altered the optical responses of **2** ^**Py**^ and **2** ^**Pz**^. This work highlights both structural and stimuli-responsive strategies for tuning organic diradicaloid systems, thereby opening new avenues for their applications in functional materials and coordination chemistry.

## Graphical abstract

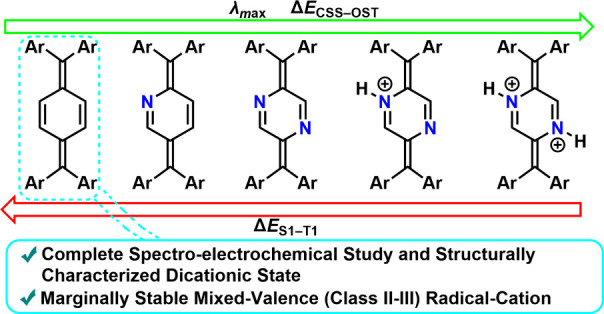

## Introduction

Organic diradicaloids play a crucial role in many fields of chemistry and materials science.^1–3^ Following Gomberg’s pioneering discovery of the persistent triphenylmethyl radical in 1900,^4^ Thiele introduced the first stable organic diradicaloid α,α,α′,α′-tetraphenyl-*p*-quinodimethane, commonly known as Thiele’s hydrocarbon (TH) (Figure 1).^5^ To attain aromaticity in its central ring, TH can be represented by two resonance forms: a closed-shell quinoid and an open-shell benzenoid (Figure 1).^6^ Because of this characteristic, several derivatives of Thiele’s hydrocarbon have been developed, showing potential applications in organic electronics, spintronics, singlet fission materials, and photodynamic therapy.^7–9^ Several strategies, such as varying spacer lengths, changing substituents in a spacer, and exploring syn–anti isomerism, have been employed to modulate their diradical character and optoelectronic properties.^10–13^ Recent studies have proven that incorporation of heteroatoms (B, S, O, N, etc.) onto the π-skeletons is an alternative strategy to modulate a diradical character and properties of organic diradicaloids.^14–18^ Although tuning their diradical character and thus their photophysical and electronic properties through changes in internal structure is well established, modulation through external stimuli such as acid–base coordination has been less explored, primarily due to the stability concerns.^19,20^ The introduction of heteroatoms opens up additional tunability through Brønsted or Lewis acid–base coordination. A representative example was reported by Dou and co-workers, who successfully synthesized a boron containing organic diradicaloid that shows dynamic modulation of diradical character by simple Lewis acid–base coordination (Figure 1).^21^ A year later, Wang and co-workers reported a nitrogen-centered diradical dication featuring oxygen as the heteroatom, which could reversibly bind to Lewis acids to fine-tune its diradical character (Figure 1).^22^ More recently, Yang and co-workers designed a nitrogen-centered diradicaloid in which protonation of the same nitrogen atom significantly altered the molecule’s photophysical and electronic properties (Figure 1).^23^

**Figure 1:**
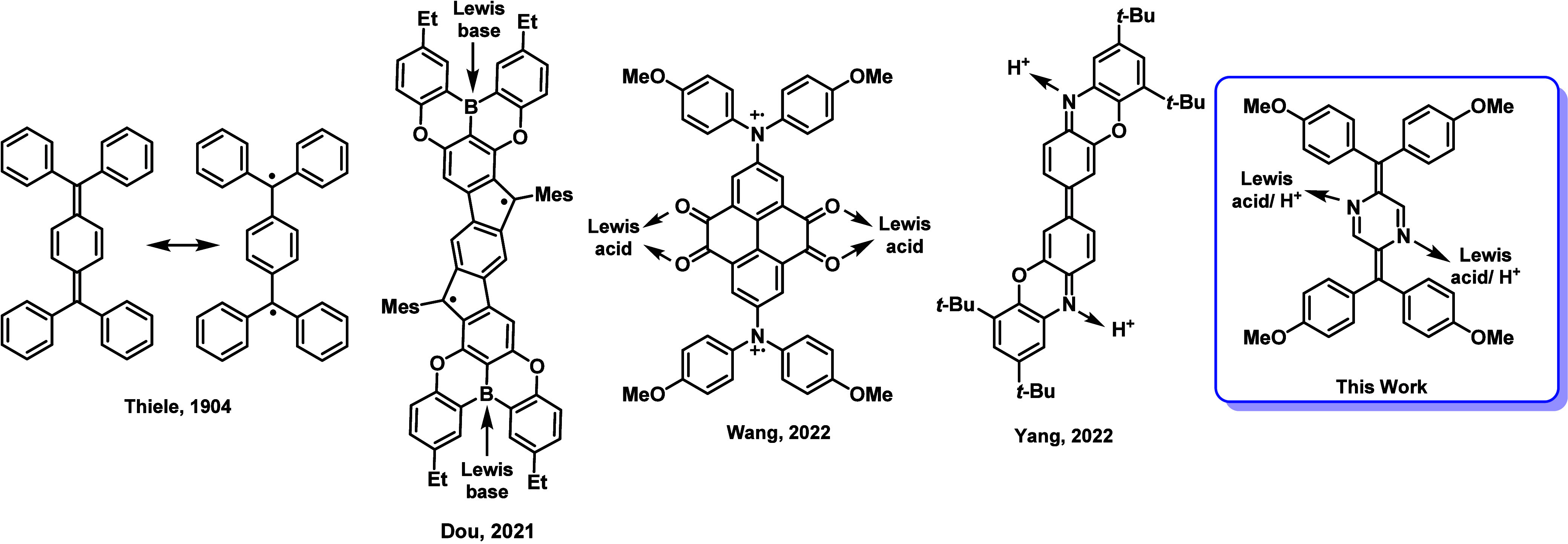
Chemical structures of organic diradicaloids.

In this context, we were interested and motivated to expand this chemistry to the most classical type of organic diradicaloids, namely, TH derivatives. To enhance the kinetic stability, 4-methoxy-substituted aryl end groups were employed. We synthesized a series of TH-based diradicaloids using phenyl, pyridinyl, and pyrazinyl spacers, introducing a sequential increase in heteroatom doping (0, 1, and 2 nitrogen atoms, respectively). To the best of our knowledge, while a few recent studies have examined pyrazine-based TH systems, the pyridine-based TH derivative has not yet been reported.^24^ A clear and systematic trend was observed in the electronic and photophysical properties across the series, progressing from phenyl to pyridyl to pyrazinyl spacers. Although nitrogen atoms have previously been used to tune the physicochemical properties of diradicaloids, systematic investigation into the effect of increasing heteroatom content has not been previously explored. Additionally, we studied the further tunability of these diradicaloids through coordination with Lewis and Brønsted acids. Remarkably, this coordination was reversible, and the original diradicaloid structure could be regenerated by the addition of a stronger base, such as *N*,*N*-dimethylaminopyridine (DMAP), displacing the coordinated acid from pyridine or pyrazine. Furthermore, for the TH derivative with the phenyl spacer, we observed potential inversion, and a case of mixed-valency wherein strong electronic coupling is observed despite diminishingly small values of the comproportionation constant (*K*_c_). The potential inversion makes these molecules potentially useful two-electron transfer reagents. Additionally, the observed strong electronic coupling despite a small *K*_c_ value has fundamental implications for the understanding of electron transfer and electronic coupling with consequences for diverse fields such as molecular electronics, materials science, energy conversion, and biological systems. Overall, this study offers new insights into both internal and external control mechanisms for tuning the properties of classical organic diradicaloid systems.

## Results and Discussion

### Syntheses and Characterization

For the synthesis of these diradicaloids, we followed a reductive dearomatization reaction of the corresponding diol compounds (Scheme 1).^25^ Lithiation of 4-bromoanisole using *n*-butyllithium, followed by reaction with terephthaloyl chloride (in the case of **1** ^**Ph**^) or corresponding dimethylester (in the case of **1** ^**Py**^ and **1** ^**Pz**^) yielded the corresponding diol compounds **1** ^**Ph**^, **1** ^**Py**^, and **1** ^**Pz**^. All the compounds were characterized by ^1^H, ^13^C NMR spectroscopy, elemental analysis, and high-resolution mass spectrometry. The ^1^H NMR spectra of **1** ^**Ph**^ and **1** ^**Pz**^ show a characteristic broad singlet signal for the OH proton at 2.76 and 5.10 ppm, respectively (Figures S1 and S5). On the other hand, the spectrum of **1** ^**Py**^ exhibits two OH signals, one at 2.91 ppm and another at 6.16 ppm, indicating the influence of the N atom, which breaks the local symmetry in this system (Figure S3). **1** ^**Pz**^ was further characterized by a single-crystal XRD measurement. Colorless block crystals of **1** ^**Pz**^ were obtained via slow evaporation of a concentrated ethyl acetate solution. The molecular structure from XRD reveals C1–C2 (1.5361(14) Å) and C1–O1 (1.4261(12) Å) bond lengths consistent with a typical single bond (Figure 2). The comparable C2–N1 and C3–N1 bond lengths of 1.3348(13) and 1.3387(13) Å, respectively, together with the intermediate between single and double bond character of C2–C3 (1.3923(14) Å) suggest the benzenoid nature of the pyrazine ring.

**Scheme 1:**
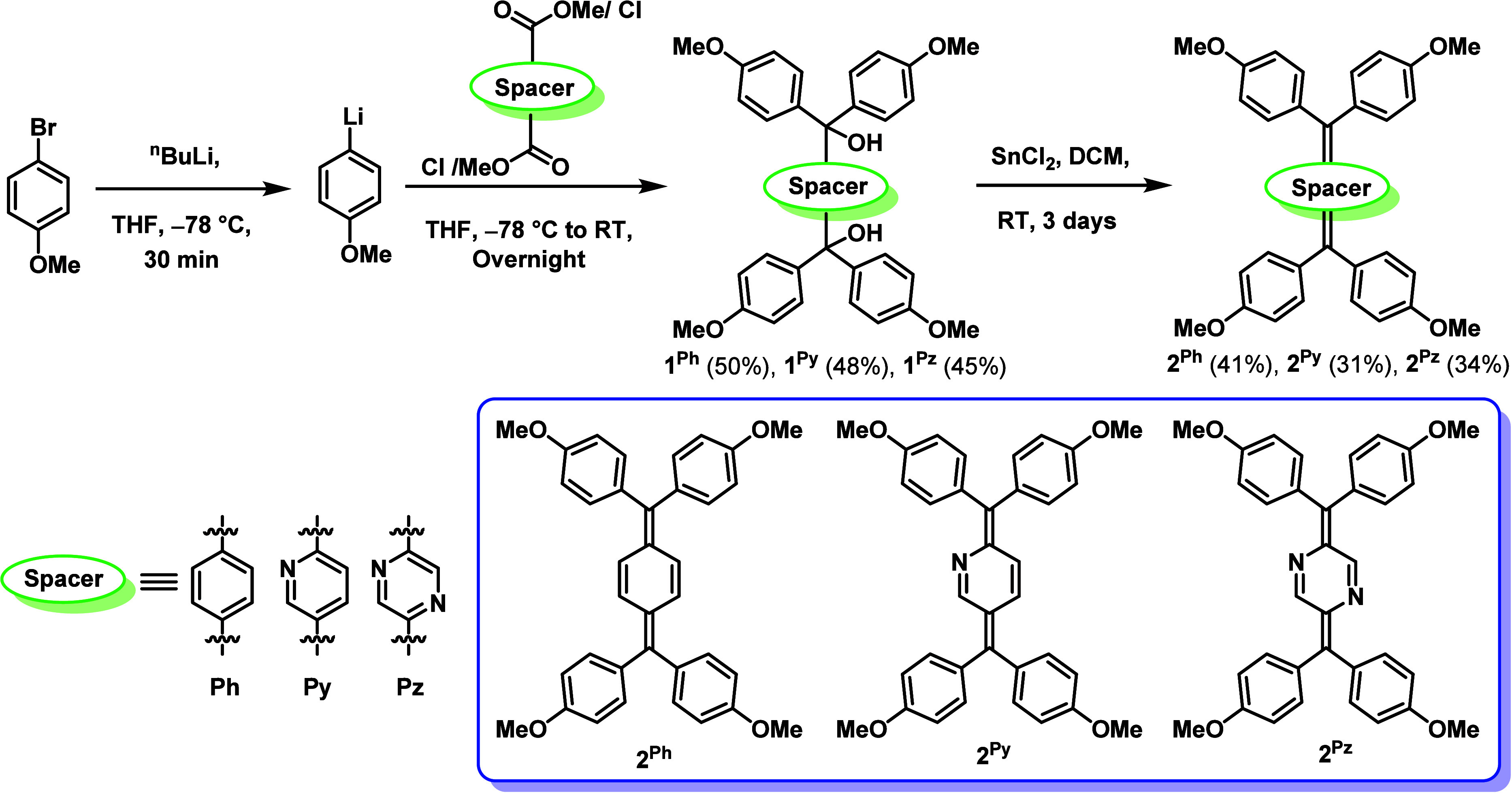
Synthesis of **1** ^**Ph**^, **1** ^**Py**^, **1** ^**Pz**^, **2** ^**Ph**^, **2** ^**Py**^ and **2** ^**Pz**^

**Figure 2:**
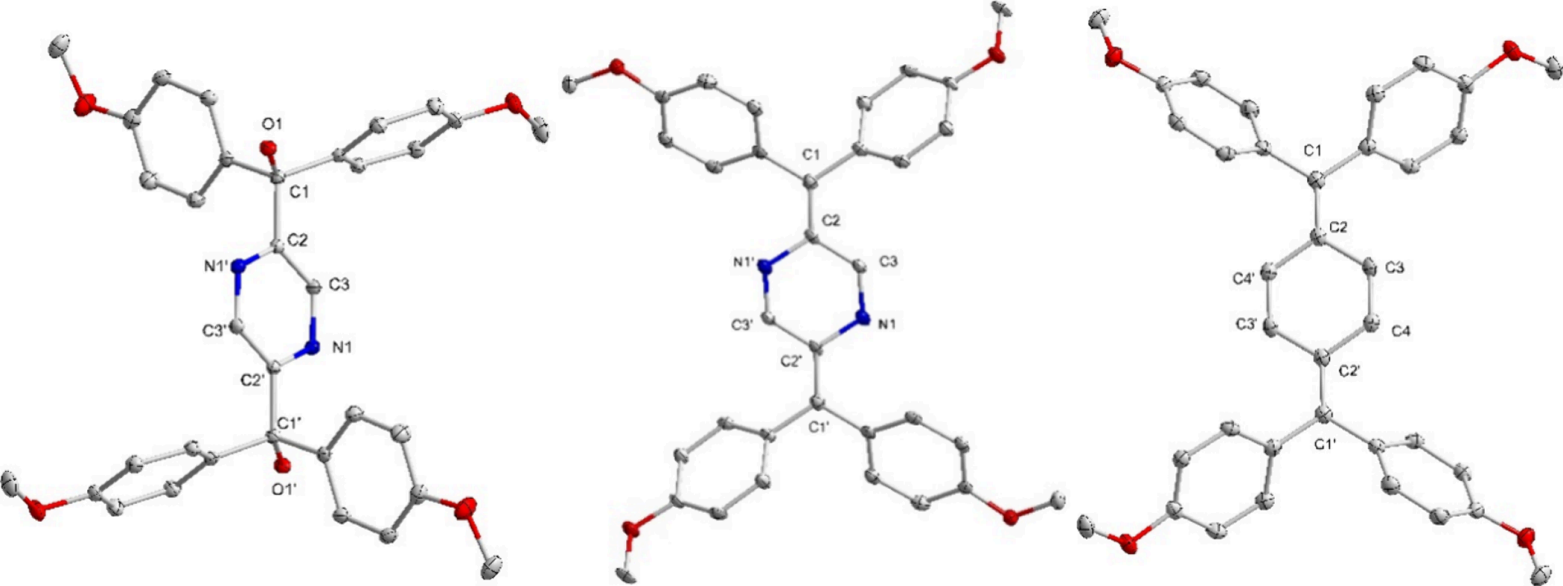
Molecular structure of **1** ^Pz^ (left), **2** ^Pz^ (middle), and **2** ^Ph^ (right) with thermal ellipsoids at the 50% probability level. All H atoms are omitted for clarity reasons. Selected bond lengths [Å] **1** ^Pz^: C1–C2 1.5361(14); C2–C3 1.3923(14); C3–N1 1.3387(13); C2–N1′ 1.3348(13); C1–O1 1.4261(12). **2** ^Pz^: C1–C2 1.376(4); C2–C3 1.435(4); C3–N1 1.300(4); C2–N1′ 1.416(3). **2** ^Ph^: C1–C2 1.381(2); C2–C3 1.451(2); C3–C4 1.347(2); C2–C4′ 1.449(2).

Reductive dearomatization reactions of **1** ^**Ph**^, **1** ^**Py**^, and **1** ^**Pz**^ were carried out using SnCl_2_ as the reductant and DCM as the solvent under an inert atmosphere, affording the diradicaloids **2** ^**Ph**^, **2** ^**Py**^, and **2** ^**Pz**^, respectively (Scheme 1). In the case of **2** ^**Ph**^, the color of the solution changed from light yellow to orange, as expected for phenyl spacer-based diradicaloids of TH derivatives. However, for **2** ^**Py**^ and **2** ^**Pz**^, the blue-violet color of the reaction mixture indicates the possibility of further coordination to the Sn derivatives or HCl through the nitrogen atom of the pyridine and pyrazine spacers, respectively. Despite this, orange-red-colored crystalline pure **2** ^**Py**^ and **2** ^**Pz**^ were successfully isolated after sequential aqueous work up with NH_4_Cl, NaHCO_3_, and brine solutions. All compounds were characterized by ^1^H, ^13^C NMR spectroscopy, elemental analysis, and high-resolution mass spectrometry. The ^1^H NMR spectra show well resolved signals shifted upfield compared to the corresponding diol compounds, consistent with a disruption of the aromatic ring current and formation of predominantly quinoid structures in the ground state (Figures S7–S15). To check the possibility of populating the triplet state at higher temperatures, variable temperature NMR experiments on **2** ^**Pz**^ were performed. No spectral broadening was observed until 100 °C, indicating a closed-shell singlet ground state with a large singlet–triplet energy gap (Figure S16). Variable temperature EPR experiments on **2** ^**Ph**^, **2** ^**Py**^, and **2** ^**Pz**^ (Figures S79–S81) further confirm the quinoid nature of the compounds at a measurable temperature range.

Slow evaporation of the concentrated dichloroethane (DCE) solutions of **2** ^**Ph**^, **2** ^**Py**^, and **2** ^**Pz**^ provided single crystals suitable for single-crystal XRD measurements. The molecular structure obtained from the crystal data reveals that the C1–C2 bond lengths in **2** ^**Ph**^, **2** ^**Py**^, and **2** ^**Pz**^ are 1.381(2), 1.381(2), and 1.376(4) Å, respectively, which are comparable to the parent TH compound (1.381(3) Å) (Figure 2).^26^ The C2–C3 and C3–C4 bond lengths in the case of **2** ^**Ph**^ are 1.451(2) and 1.347(2) Å, corresponding to typical single and double bond, respectively. This clearly supports the quinoid character of the central ring, with a bond length alternation (BLA) of around 0.104 Å. Similarly, in **2** ^**Pz**^, the C2–N1 and C3–N1 bond lengths are 1.416(3) and 1.300(4) Å, respectively, indicating a quinoid nature of the pyrazine ring, with a BLA of 0.116 Å. These diradicaloid compounds (**2** ^**Ph**^, **2** ^**Py**^, and **2** ^**Pz**^) are stable under ambient conditions in the solid state for several weeks and in solution for several days. Notably, all workup procedures were performed under ambient conditions using regular (nondry) solvents. However, in solution, slow reoxidation to the parent diol compounds was observed over time (Figure S27). In the case of **2** ^**Pz**^, further oxidation led to the formation of a keto derivative (**2** ^**Pz**^ **·O**_**2**_), isolated during a crystallization attempt under ambient conditions (Figure S39), which indicates the radical behavior of these compounds.^27,11a^

### Photophysical Properties, Cyclic Voltammetry, and Spectroelectrochemistry

Ultraviolet–visible–near-infrared (UV–vis–NIR) spectroscopy of the diradicaloid shows absorption maximum for **2** ^**Ph**^, **2** ^**Py**^, and **2** ^**Pz**^ at 450, 469, and 475 nm, respectively (Figure 3). A clear trend was observed with an increase in the number of heteroatoms in the system; the absorption maximum exhibited a progressive redshift. Time-dependent density functional theory (TD-DFT) calculations, performed using ORCA,^28^ suggest that the lowest-energy electronic transition corresponds to a π→π* excitation from the HOMO to the LUMO (Tables S10–S12). These frontier molecular orbitals are predominantly localized on the central spacer unit with only minor contributions from the terminal aryl groups (Figure 3 and Figures S83–S88).

**Figure 3:**
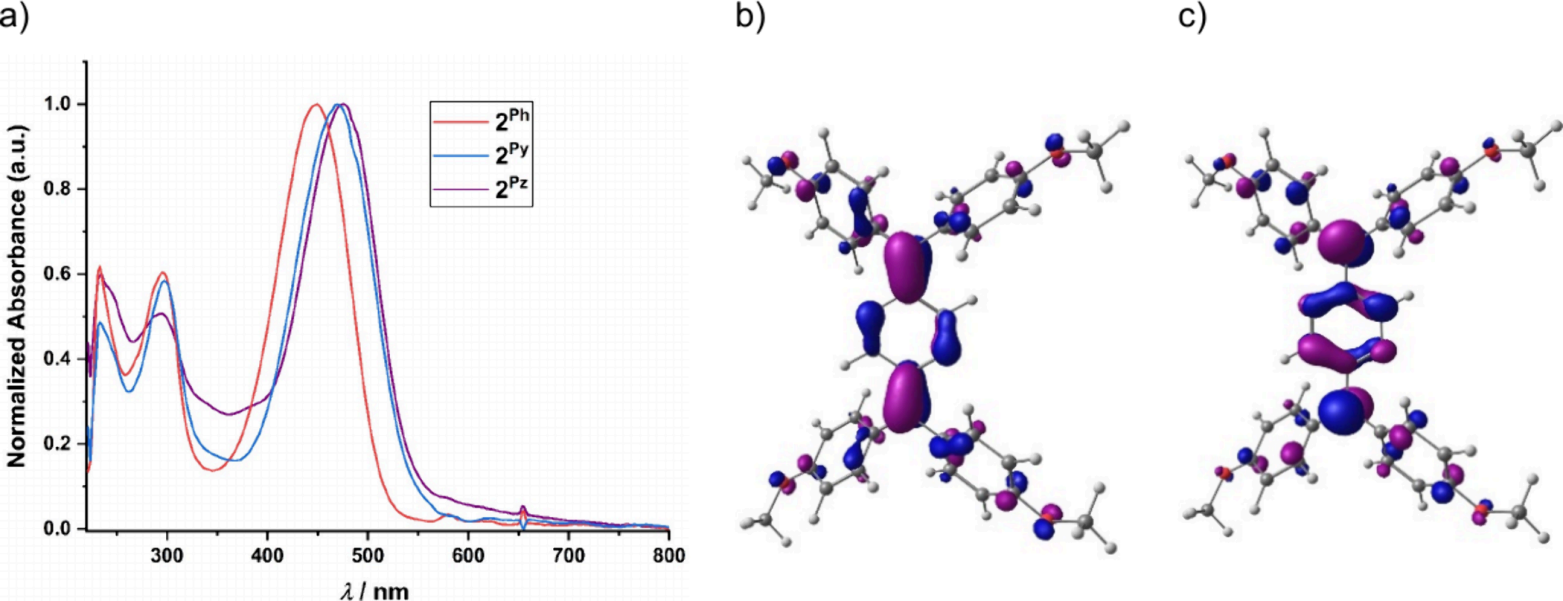
(a) Comparison of UV–vis–NIR spectra of compounds **2** ^**Ph**^, **2** ^**Py**^, and **2** ^**Pz**^ in DCM at a concentration around 13.5 μM at room temperature. (b) HOMO of **2** ^Pz^ (isosurface value = 0.045). (c) LUMO of **2** ^**Pz**^ (isosurface value = 0.045).

The calculation of the energy difference between the closed-shell singlet and open-shell triplet (Δ*E*_CSS–OST_) state shows an increasing trend from **2** ^**Ph**^ to **2** ^**Py**^ to **2** ^**Pz**^, indicating a progressive destabilization of the triplet state upon nitrogen doping (Figure 8 and Table S9). In order to analyze the electronic structure of the compounds **2** ^**Ph**^, **2** ^**Py**^, and **2** ^**Pz**^, we conducted broken-symmetry DFT calculations as a function of the C–C bond lengths between the C atom in the para position of the linker and the radical C atom, as well as CASSCF studies for three different C–C bond lengths (1.38, 1.46, and 1.56 Å). The relative singlet (BS) and triplet (T) energies for the three neutral molecules as a function of the C–C distance are shown in Figure S104. In all cases, the singlet is considerably stabilized compared to the triplet (Δ*E*_BS‑T_ ∼110 kJ/mol for a C–C distance of 1.38 Å, which decreases to ∼20 kJ/mol for the longer C–C distances between 1.52 and 1.56 Å). The character of the ground state varies from an open-shell singlet for the longest distances to a closed-shell singlet for most of the C–C distance range. This is evidenced by the calculation of the diradical character *y*_0_ of **2** ^**Ph**^, **2** ^**Py**^, and **2** ^**Pz**^ for each C–C distance,^29^ from the occupation of the highest occupied and lowest unoccupied natural orbitals (HONO and LUNO). These results, shown in Table S20, indicate that the diradical character is only of some importance for the longest C–C distances (1.56 Å), being 0.16 for **2** ^**Pz**^, 0.27 for **2** ^**Py**^, and 0.29 for **2** ^**Ph**^. For the intermediate distances (1.46 Å), the diradical characters are 0.01, 0.03, and 0.07, respectively, while for shortest distances (1.38 Å), the values are zero. The low diradical character is in agreement with the large singlet–triplet gaps.

For **2** ^**Ph**^, **2** ^**Py**^, and **2** ^**Pz**^ with the three C–C distances, we performed CASSCF/NEVPT2 calculations using an active space of 2 electrons in two orbitals (CAS­(2,2)). The orbital diagrams, including the adjacent orbitals that are not part of the active space, are shown in Figures S100–S102. The ground state configurations in all cases correspond to closed-shell singlets with the sole exception of **2** ^**Py**^ with a C–C distance of 1.46 Å, which appears to have a predominantly open-shell singlet ground state. The compositions and relative energies of one triplet and three singlet states for each calculated structure are shown in Table S18. It is possible that a CAS­(2,2) calculation is too small to capture correlation effects, as well as the nuances of a relatively extended π system. Therefore, for **2** ^**Py**^, we explored calculations with larger active spaces (CAS­(4,4), CAS­(6,6), and in one case CAS­(8,8)). The orbital energy diagram for **2** ^**Py**^ with a C–C distance of 1.46 Å and four different active spaces is shown in Figure S103. It is observed that the anomalous open-shell singlet ground state predicted by the CAS­(2,2) calculation is no longer present when the active space is enlarged. The energies of the orbitals, as well as the exact weight of two possible electronic configurations in the ground state, change slightly upon increasing the active space size, but the qualitative description is the same: a closed-shell singlet ground state in all cases. A summary of the state compositions and energies for CASSCF/NEVPT2 calculations of **2** ^**Py**^ is given in Table S19. It appears that for our purposes, a CAS­(2,2) active space is large enough to reveal the correct ground state in most cases, with a larger CAS­(4,4) calculation being useful when ambiguities arise.

We also calculated the excited state energies, important for various photophysical processes such as thermally activated delayed fluorescence (TADF) and singlet fission, of these diradicaloids.^30^ The energy gap between the S_1_ and T_1_ excited states (Δ*E*_S1–T1_) follows the reverse order, decreasing from **2** ^**Ph**^ to **2** ^**Py**^ to **2** ^**Pz**^ (Figure 8 and Table S9). All compounds are weakly emissive at room temperature. The emission intensity increases slightly at lower temperature, but because of the broad nature of the spectra and low intensity, measurements of lifetime or quantum yield were not possible (Figures S62–S65). To further elucidate the origin of the observed emission band, the effect of solvent polarity and temperature on the absorption and emission spectra of **2** ^**Py**^ was investigated. Figure 4 shows the linear UV–vis absorption spectra of **2** ^**Py**^ measured at 293 K in dichloromethane (DCM) and toluene solutions, together with the corresponding emission spectra recorded at both 185 and 293 K. The absorption maximum of **2** ^**Py**^ in solution exhibits a negligible solvent dependence, indicating that the Franck–Condon region of the excited state is only weakly polar. In contrast, the emission spectra of **2** ^**Py**^ reveal pronounced solvent- and temperature-dependent shifts, consistent with emission from a zwitterionic S_1_ state, as previously reported for related Thiele-type systems by Perepichka and co-workers.^11b^ At 185 K, the emission peaks are at 700 nm in toluene but redshift to 782 nm in DCM, reflecting the enhanced stabilization of the charge-separated excited state in the more polar solvent. At 293 K, increased thermal motion leads to an emission peaking at 736 nm in DCM, while the emission maximum in toluene remains essentially unchanged at 700 nm. These observations demonstrate that emission of **2** ^**Py**^ originates (i) from a strongly polar excited state and (ii) from an excited state ensemble that is inhomogeneously distributed, presumably along the low-frequency torsional degrees of freedom. The emission spectral profile thus reflects the excited-to-ground state energy gap sensed by a temperature-dependent torsional distribution of molecules.

**Figure 4:**
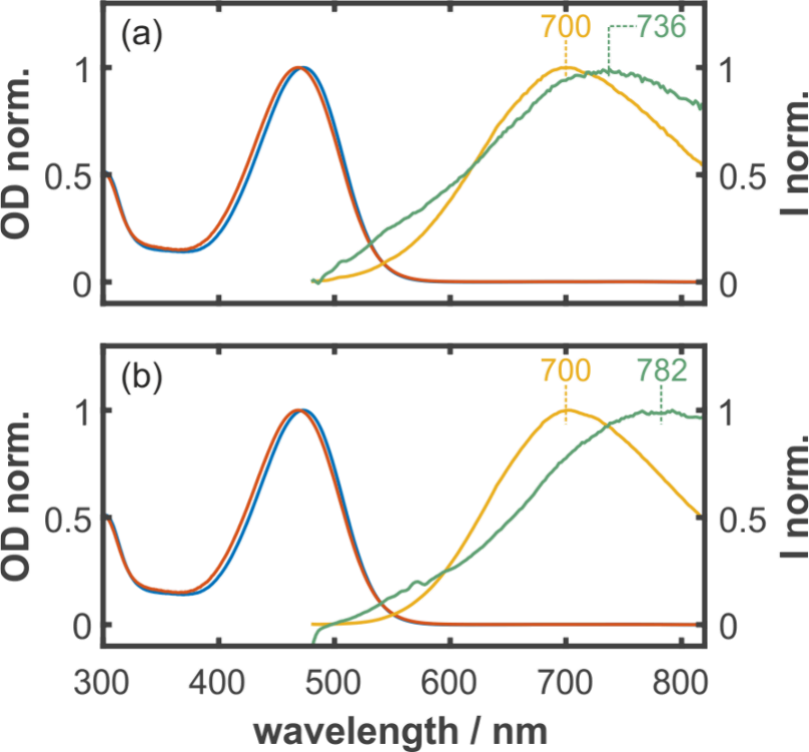
(a, b) Normalized UV–vis spectra of **2** ^Py^ at 293 K in DCM (red) and toluene (blue) solutions. (a) Normalized Emission spectra of **2** ^Py^ in DCM (green) and Toluene (yellow) at 293 K. (b) Normalized emission spectra of **2** ^Py^ in DCM (green) and toluene (yellow) at 185 K.

To help rationalize the relatively low fluorescence quantum yield compared to the polyhalogenated derivatives reported by Perepichka et al. and Blasi et al.,^11b,20b^ femtosecond UV-pump/visible-probe spectroscopy was carried out with excitation at 470 nm, corresponding to the lowest-energy π–π* absorption band. Data were collected for **2** ^**Py**^ in DCM at room temperature. A broader photophysical study, including **2** ^**Pz**^ and **2** ^**Ph**^, is currently underway and will be reported elsewhere.

Figure 5 shows transient absorption spectra for a variety of pump–probe delays. At the earliest delays (Figure 5a), two induced absorption bands peaking at 384 and 769 nm are detected, which are separated by the negative ground-state bleaching band. Within less than 200 fs, the red band partially decays while shifting slightly to the blue (from ∼770 to 763 nm). At the same time, another induced absorption appears at a wavelength of 562 nm, thereby giving rise to an apparent isosbestic point at 657 nm (cf. asterisk). The early time spectrotemporal evolution is indicative of an initial structural relaxation of the system away from the Franck–Condon region together with solvent reorganization and intramolecular vibrational redistribution.

**Figure 5:**
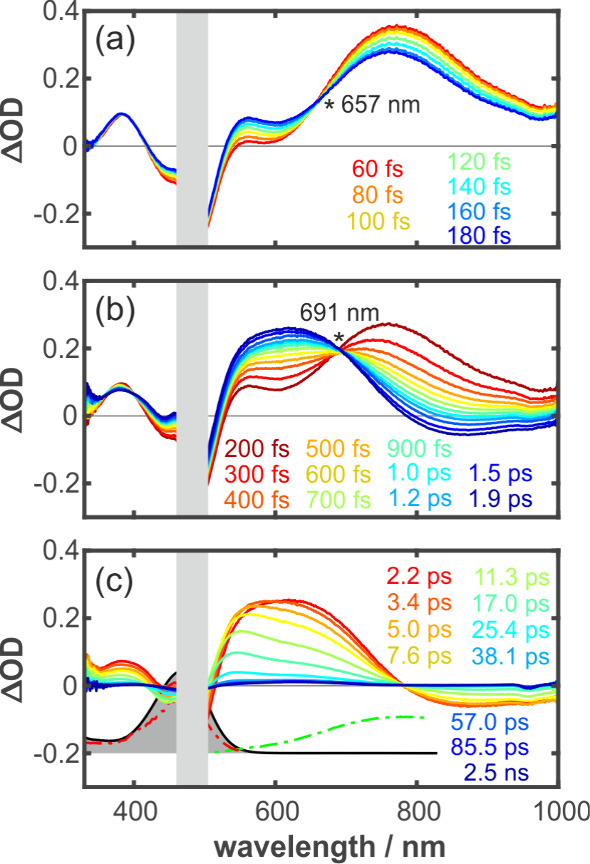
(a–c) Transient absorption spectra of **2** ^Py^ at selected pump–probe delays. Colored curves represent the experimental data recorded after 470 nm excitation. Colors correspond to the indicated time delays. Asterisks denote isosbestic points. Strong pump scatter was removed from the figure and is denoted in light gray. (a) Transient absorption spectra recorded immediately after optical excitation; an isosbestic point is observed at 657 nm. (b) Transient absorption spectra recorded between 200 fs and 1.9 ps; an isosbestic point is observed at 691 nm. (c) Late-time transient absorption spectra (≤2.2 ps). The shaded black solid line shows the **2** ^Py^ UV–vis spectra, while the red and green dashed lines show the excitation (measured at 725 nm) and emission spectra (excited at 469 nm), respectively. These were vertically offset for the sake of clarity.

On intermediate delays (Figure 5b), the broad 770 nm band continues to decay and eventually gives way to a net-negative band that is maximal around 820 nm. In light of its enormous redshift relative to the ground-state’s linear absorption, it must be due to excited state stimulated emission. Simultaneously, the 562 nm absorption builds up even more, it broadens, and it shifts to the red to adopt a final peak position of ∼620 nm. This delay window features another isosbestic point at 691 nm, which is clearly indicative of a simple population transfer between two distinct excited states, namely, the local but relaxed excited state and a distinct highly polar excited state from which stimulated emission is observed.

On longer time scales (Figure 5c), all spectral features, including excited state absorption, stimulated emission, and ground-state bleach, decay almost to zero within approximately 40 ps, indicating rapid nonradiative deactivation back to the ground state.

Our data indicate that the photophysics of **2** ^**Py**^ are mechanistically similar to those recently reported for other Thiele-type hydrocarbons.^11b,20b^ As pointed out by Liu et al.^11b^ and Punzi et al.,^20b^ the ground state is predominantly closed-shell and partially quinoidal, and photoexcitation initially populates a bright singlet π→π* state (S_1_). Subsequently, sequential excited-state relaxations give rise to two isosbestic points in the transient spectra, which is consistent with rapid intramolecular reorganization followed by population transfer into a charge-separated zwitterionic configuration, denoted Z_1_.

However, unlike the systems reported previously, the zwitterionic Z_1_ state of **2** ^**Py**^ exhibits a very short lifetime and, consequently, has an extremely small fluorescence quantum yield, thus rendering it weakly emissive. This behavior is attributed to efficient nonradiative decay from the relaxed zwitterionic minimum, likely facilitated by strong vibronic coupling and a small Z_1_–S_0_ energy gap. Overall, the fundamental mechanistic framework—photoexcitation followed by local intramolecular relaxation and formation of a zwitterionic excited state—is shared among all Thiele-type systems studied so far, with our pyridine-containing derivative **2** ^**Py**^ lying at the extreme limit of fast nonradiative decay and weak emission.

Cyclic voltammetry studies of **2** ^**Ph**^, **2** ^**Py**^, and **2** ^**Pz**^ exhibit two one-electron oxidation processes. For **2** ^**Ph**^, in DCM with Bu_4_NPF_6_ as the supporting electrolyte, the oxidations are reversible and appear as a single, reversible two-electron oxidation event, pointing to potential inversion (Figure 6). This observation points to a rather limited stability of the intermediate radical cation state under those conditions, with a *K*_c_ for the thermodynamic stability of the odd-electron species approaching a statistical value of 4.^31^ However, when the measurement was performed using an electrolyte with a weakly coordinating anion such as Bu_4_NBArF_24_, the separation between the oxidation steps was clearly visible at *E*_1/2_ = −96 and 104 mV vs FcH/FcH^+^ (Figure 6). An electrochemical potential difference of 0.2 V between the two oxidation events suggests a comproportionation constant (*K*_c_) of 2.5 × 10^3^ at room temperature. As the separation between the oxidation steps is only observed in the presence of a weakly coordinating anion, the origin of this separation is likely predominantly electrostatic in nature.^31^ In contrast to **2** ^**Ph**^, the oxidation processes of **2** ^**Py**^ and **2** ^**Pz**^ are irreversible (Figures S72 and S73), likely due to radical quenching of the intermediate radical cations via interactions with the nitrogen heteroatoms.^24b^

**Figure 6:**
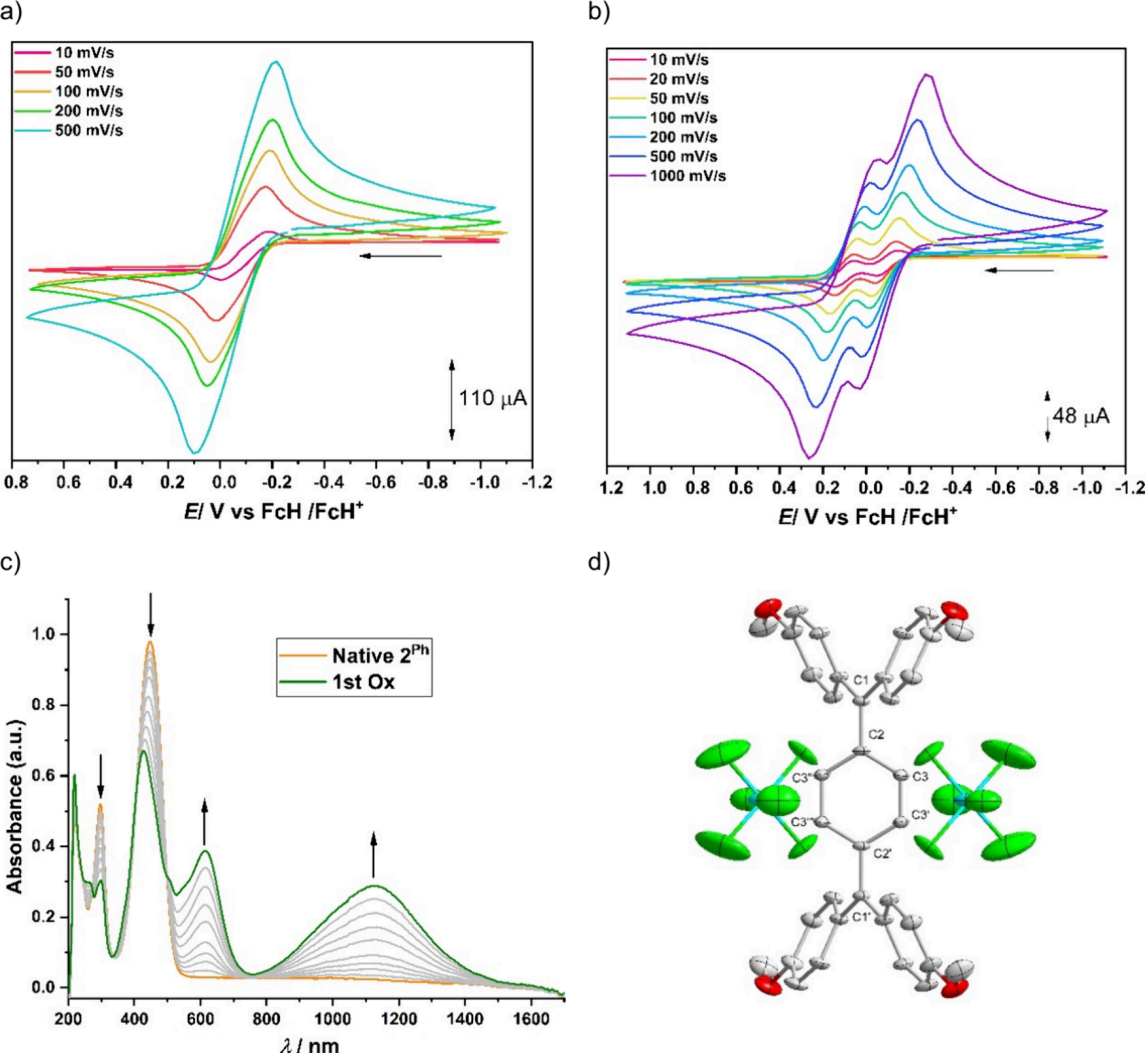
(a) CV of **2** ^Ph^ in DCM/0.1 M Bu_4_NPF_6_ was measured at a GC working electrode at different scan rates and room temperature. (b) CV of **2** ^Ph^ in DCM/0.02 M Bu_4_NBArF_24_ was measured at a GC working electrode at different scan rates and room temperature. (c) Changes in the UV–vis–NIR spectra of **2** ^Ph^ at concentration around 25 μM in DCM/0.2 M Bu_4_NPF_6_ during the first oxidation with a Au working electrode at room temperature. (d) Molecular structure of **2** ^Ph^DC with thermal ellipsoids at the 50% probability level. All H atoms are omitted for clarity reasons. Selected bond lengths [Å]: C1–C2 1.461(6); C2–C3 1.398(4); C3–C3′ 1.381(7); C2–C3″ 1.398(4).

Chemical oxidation of **2** ^**Ph**^ using 2.5 equiv of NOSbF_6_ results in the formation of the corresponding dicationic species **2** ^**Ph**^ **DC** (Scheme 2). This compound was characterized using ^1^H NMR spectroscopy, elemental analysis, as well as high-resolution mass spectrometry. Single crystals suitable for XRD analysis were obtained by slow diffusion of Et_2_O into a concentrated acetonitrile solution of **2** ^**Ph**^ **DC**. The molecular structure from XRD reveals a C1–C2 bond length of 1.461(6) Å, significantly longer than that in **2** ^**Ph**^ and comparable C2–C3 and C3–C3′ bond lengths (1.451(2) and 1.347(2) Å, respectively), suggesting a restoration of benzenoid character in the central phenyl ring upon oxidation (Figure 6 and Scheme 2).

**Scheme 2:**
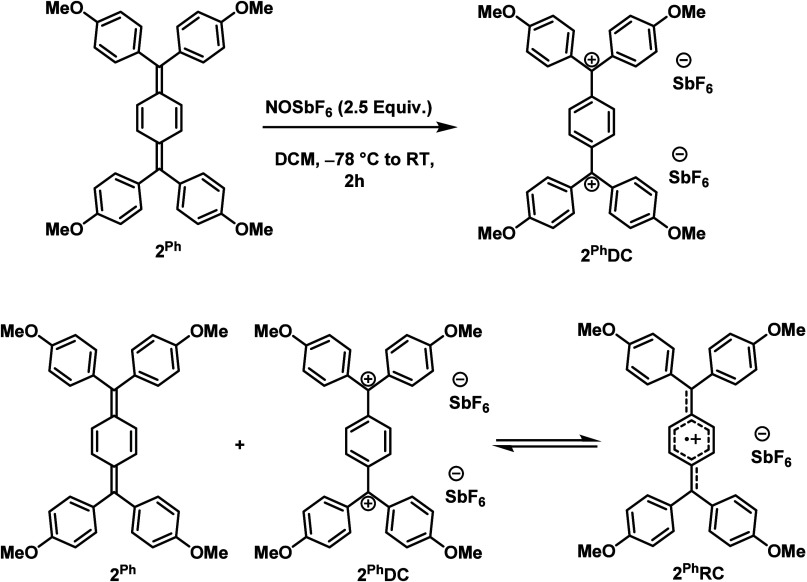
Synthesis of **2** ^**Ph**^, **2** ^**Ph**^ **DC**, and **2** ^**Ph**^ **RC**

During the oxidation reaction of **2** ^**Ph**^, the solution color initially changed from orange to olive-green and finally to the corresponding pink-red color of the dication **2** ^**Ph**^ **DC**. This indicates the possibility of observing and identifying the intermediate radical cation. UV–vis–NIR spectroelectrochemistry studies of **2** ^**Ph**^ in DCM/0.1 M Bu_4_NPF_6_ revealed the formation of new bands at 620 and 1130 nm at the onset of the oxidation process, which are attributed to the radical cation **2** ^**Ph**^ **RC** (Figure 6). These bands rapidly disappeared during the same oxidation step with the concomitant formation of a new absorption band at 510 nm, consistent with the final formation of **2** ^**Ph**^ **DC** (Figure S75), thereby suggesting the formation as well as the limited stability of the intermediate radical cation state. The spectral changes during the overall two-electron oxidation step could be neatly separated into two sets based on isosbestic points (Figures S74–S76 and Figure 6). Spectroelectrochemical measurements with Bu_4_NBArF_24_ as a supporting electrolyte delivered a similar picture as above. This observation points to the fact that the weakly coordinating anion does not intrinsically influence the electronic coupling and electronic structures of these systems but only influences the separation between the oxidation steps through predominantly electrostatic interactions (see CV section above). Inspired by the observations during the spectroelectrochemical measurements, a comproportionation reaction of **2** ^**Ph**^ and **2** ^**Ph**^ **DC** was carried out. Gratifyingly, this reaction resulted in the formation of the expected olive-green radical cation **2** ^**Ph**^ **RC** (Scheme 2). UV–vis–NIR spectroscopy study of the reaction mixture confirms the formation of **2** ^**Ph**^ **RC**, showing characteristic absorption bands at 620 and 1130 nm (Figure 7). The stability of the radical cation toward disproportionation is solvent dependent, most likely due to solubility issues (Figure S52). Highly polar solvents like acetonitrile (ACN) stabilize the dication (**2** ^**Ph**^ **DC**) while nonpolar solvents such as toluene stabilize the diradicaloid (**2** ^**Ph**^) form much more than the radical cation, so the equilibrium shifts to the left side. By contrast, moderately polar solvents such as DCM and *o*-difluorobenzene (*o*-DFB) stabilize the radical cation (**2** ^**Ph**^ **RC**), thereby shifting the equilibrium to the right-hand side. These solvents thus provide ideal conditions for characterizing such a radical cationic species. The room-temperature X-band EPR spectrum of **2** ^**Ph**^ **RC** in DCM exhibits a line-rich signal centered at *g* = 2.0031, consistent with an organic radical.^32^ The spectrum could be simulated with isotropic ^1^H couplings of 4.4, 2.2, and 0.7 MHz for the 4 Ph-*H*, 8 ortho-, and 8 meta-*H* from aryl groups, respectively (Figure 7). The singly occupied molecular orbital (SOMO), spin density plot of **2** ^**Ph**^ **RC**, and Löwdin spin density distribution, calculated using DFT, indicate a symmetric distribution of the spin density throughout the π-system (Figure 7 and Figures S91 and S99).

**Figure 7:**
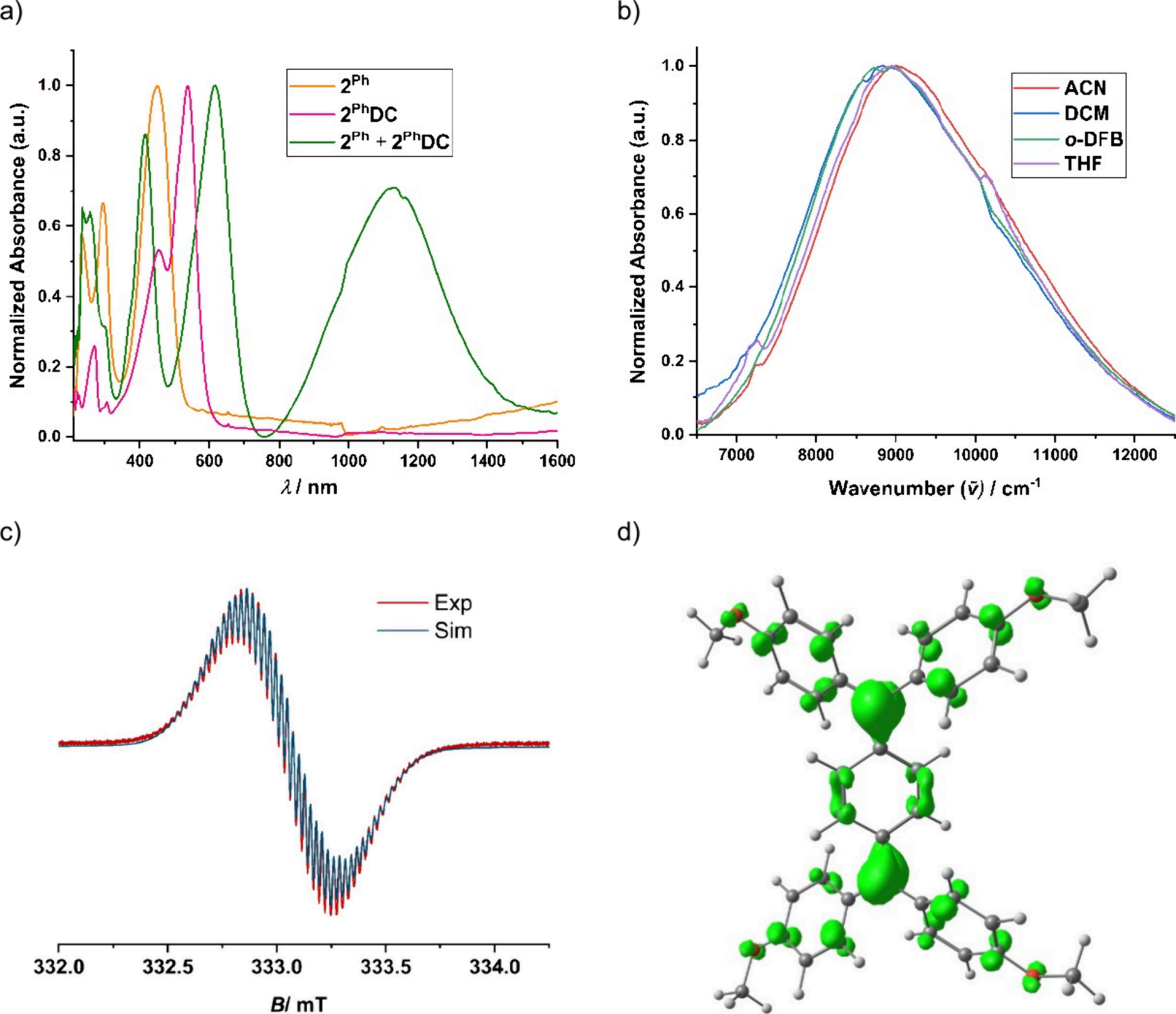
(a) Comparison of UV–vis–NIR spectra of compounds **2** ^Ph^ and **2** ^Ph^ **DC** and comproportionation reaction of **2** ^Ph^ and **2** ^Ph^ **DC** in DCM at room temperature. (b) Solvent dependence of the IV-CT band of **2** ^Ph^ **RC** measured at room temperature. (c) Experimental (red) and simulated (blue) X-band EPR spectra of **2** ^Ph^ **RC** in DCM measured at room temperature. Best-fit simulation parameters: *g*_iso_ = 2.0031, lwpp = 0.0315, a­(4H) = 4.4449 MHz (Ph-*H*), a­(8H) = 2.2435 MHz (*o*-Ar-*H*), and a­(8H) = 0.7417 MHz (*m*-Ar-*H*). (d) Spin density plot of radical cation **2** ^Ph^ **RC** (isosurface 0.0035).

Although the EPR spectroscopy and DFT calculation suggest that the nature of the radical cation is a delocalized class III type mixed-valence (MV) compound (according to Robin–Day classification),^33^ we have analyzed the intervalence charge transfer (IV-CT) band using UV–vis-NIR spectroscopy, which is regarded as a more reliable method for MV classification.^34^ The IV-CT band of **2** ^**Ph**^ **RC** in DCM (λ_max_ = 1130 nm, ${\mathrm{\upsilon ̅}}_{\max}$ = 8850 cm^–1^) shows a Gaussian shape with a slight cut off on the low-energy side, indicating a borderline class II–class III system (Figure 7). The electronic coupling integral (*V*) was calculated based on the IV-CT band using the Marcus–Hush theory, taking into account the absorption maximum ( ${\mathrm{\upsilon ̅}}_{\max}$ = 8850 cm^–1^), the bandwidth at half-height ( ${\mathrm{\upsilon ̅}}_{1/2}$ = 2420 cm^–1^), the molar absorptivity (ε = 33400 M^–1^ cm^–1^), and the distance (*R*) between two radical centers (5.7 Å; from the DFT optimized geometry) as per eq 1.

$$V=\{[2.06\times 10^{-2}({\mathrm{\upsilon ̅}}_{\max}{\mathrm{\upsilon ̅}}_{1/2}\varepsilon {)}^{1/2}]/R\}$$ 1

Applying eq 1 to **2** ^**Ph**^ **RC** yields a coupling integral of *V* = 3057 cm^–1^, which approaches, but is less than, λ/2 (4425 cm^–1^), supporting its assignment as a borderline class II–class III system. The solvent dependence of the IV-CT band (Δυ̅(DCM/ACN) ca. 150 cm^–1^) suggests a change in dipole moment upon electron transfer (Figure 5). Furthermore, the value of 2*V*/λ = 0.69 satisfies the criterion 0 < 2*V*/λ < (1 – Δυ̅/λ) and not completely but almost fits with the class II–III transition regime proposed by Sutin et al. (0.7 < 2 V/λ < 1), further confirming the presence of class II character in **2** ^**Ph**^ **RC**.^35^ These results are comparable to those reported for the nitrogen analogue of **2** ^**Ph**^ **RC**, i.e., *N*,*N*,*N*′,*N*′-tetra-4-methoxyphenyl-*p*-phenylenediamine radical cation system.^36^ In several reports in the literature, the comproportionation constant, *K*_c_, which represents the thermodynamic stability of the mixed-valent species in comparison to its neighboring homovalent counterparts, has often been directly correlated with the electronic coupling within the mixed-valent compound. As was pointed out early by Richardson and Taube, *K*_c_ is a complex term involving contributions from a host of parameters.^31^ We present here an intriguing example of a mixed-valent compound that has a diminishingly small *K*_c_ value but displays very strong electronic coupling.

In the present case, steric repulsion between *ortho*-hydrogens from the aryl end group and the phenyl spacer H makes the system nonplanar. This deviation from the planarity likely contributes to the limited stability of the intermediate radical cation state, favoring its disproportionation into the neutral species **2** ^**Ph**^ and the dication **2** ^**Ph**^ **DC**.^37^ In contrast, similar chemical oxidation reactions in the case of **2** ^**Py**^ and **2** ^**Pz**^ did not yield clean products. Instead, complex mixtures were formed, and we were unable to isolate or characterize any defined oxidized species from these reactions.

### Tuning through Acids and Bases

To explore the potential for tuning the properties of the diradicaloids through external stimuli, protonation reactions of **2** ^**Py**^ and **2** ^**Pz**^ were carried out using dimethylformamide-trifluoromethanesulfonic acid adduct (DMF·HOTf) as an acid source (Scheme 3). The reaction of **2** ^**Py**^ and **2** ^**Pz**^ with 1 equiv of DMF·HOTf afforded the corresponding dark blue-green colored monoprotonated species **3** ^**Py**^ and **3** ^**Pz**^, respectively. In the case of pyrazine-based system, a doubly protonated **4** ^**Pz**^ could also be prepared using an excess amount (3.0 equiv) of acid. A comproportionation reaction between isolated neutral **2** ^**Pz**^ and diprotonated **4** ^**Pz**^ forms the monoprotonated **3** ^**Pz**^ compound, demonstrating the reversibility of these acid–base transformations (Scheme 3). All protonated species were fully characterized by multinuclear NMR spectroscopy, elemental analysis, and high-resolution mass spectrometry. The ^1^H NMR spectra of **3** ^**Py**^, **3** ^**Pz**^, and **4** ^**Pz**^ display broad singlet peaks corresponding to the N–H protons (Figures S18, S21, and S24). In addition, a general downfield shift of all other proton signals compared to the spectra of **2** ^**Py**^ and **2** ^**Pz**^ confirms the development of positive charge(s) within the molecules upon protonation. Notably, **4** ^**Pz**^ exhibits more pronounced downfield shifts than **3** ^**Pz**^, consistent with its higher degree of protonation and formation of a doubly cationic species (Figure S24).

**Scheme 3:**
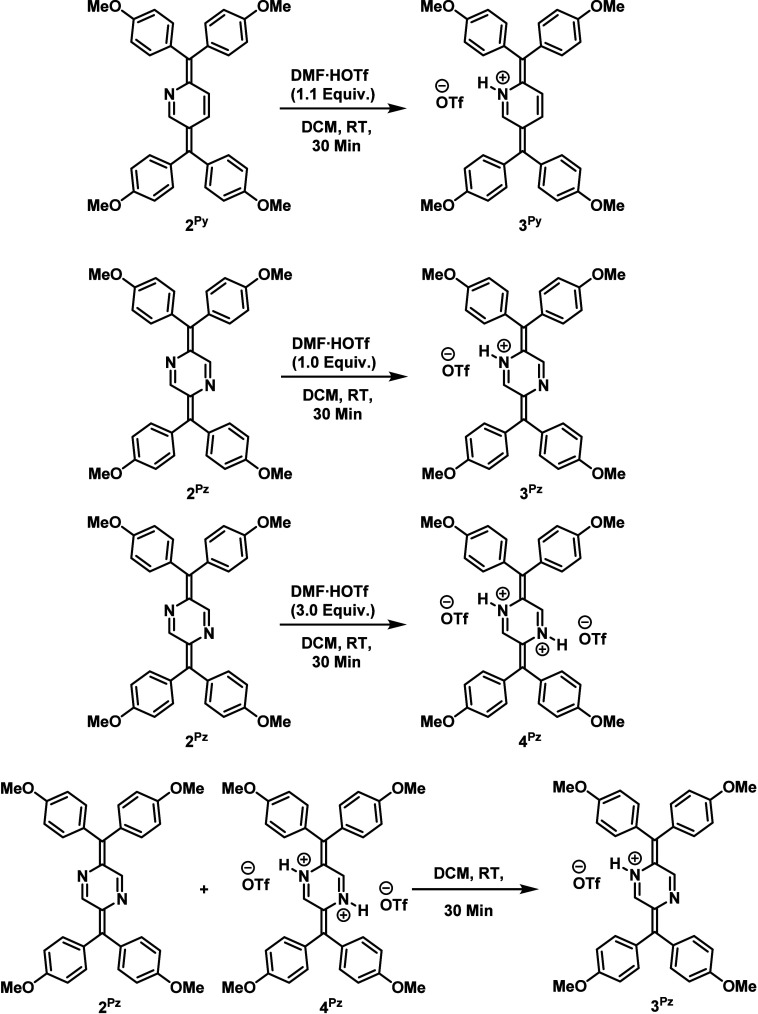
Synthesis of **3** ^**Py**^, **3** ^**Pz**^, and **4** ^**Pz**^

Single crystals of **3** ^**Py**^ and **4** ^**Pz**^, suitable for XRD analysis, were obtained by slow diffusion of Et_2_O into a concentrated solution of the compound in DCM and acetonitrile, respectively. The molecular structure from the crystal reveals C1–C2 bond lengths of 1.388(10) Å for **3** ^**Py**^ and 1.378(4) Å for **4** ^**Pz**^, which are comparable to those observed in **2** ^**Py**^ and **2** ^**Pz**^ (1.381(2) and 1.377(4) Å, respectively). Other bond lengths are also comparable, supporting the quinoid nature of these cationic compounds in their ground states (Table 1 and Figures S37 and S38).

**Table 1:** Selected Bond Lengths (Å)

bond	2^Ph^	2^Py^ ^a^	2^Pz^	3^Py^	4^Pz^	**TH** ^26^
C1–C2	1.381(2)	1.381(2)	1.376(4)	1.388(10)	1.378(4)	1.381(3)
C2–C3	1.451(2)		1.435(4)	1.457(10)	1.411(4)	1.449(3)
C3–C4	1.347(2)			1.355(9)		1.346(3)
C2–N1			1.416(4)	1.390(9)	1.411(4)	
C3–N1			1.300(4)		1.296(4)	

a Because of symmetry related disorder, the esd values of the bond lengths in the pyridine spacer are very high (Figure S34).^38^

Although protonation did not significantly alter the structural parameters of the diradicaloids, UV–vis–NIR spectroscopy measurements of these cationic compounds revealed pronounced shifts in their absorption maxima (λ_max_). In the pyridine-based system, λ_max_ shifted from 469 nm for **2** ^**Py**^ to 603 nm for **3** ^**Py**^ (Figure S55). Similarly, in the pyrazine-based systems, λ_max_ values were observed at 475, 574, and 620 nm for **2** ^**Pz**^, **3** ^**Pz**^, and **4** ^**Pz**^ respectively, showing a clear redshift with increasing protonation (Figures S57 and S58). Compound **4** ^**Pz**^ was found to be unstable at very low concentrations, readily dissociating into **3** ^**Pz**^ and HOTf (Figure S59). To better understand the formation and stability of these protonated species at lower concentrations, an acid–base titration study was conducted, monitored by UV–vis–NIR spectroscopy. The appearance of multiple isosbestic points during the titration of **2** ^**Pz**^ with DMF·HOTf suggested a direct formation of **3** ^**Pz**^ (Figure 8). However, formation of **4** ^**Pz**^ was only observed upon the addition of excess acid (more than 5 equiv), indicating its thermodynamic instability under dilute conditions (Figure 8). Emission spectra of these adducts remain weakly emissive. Although an increase in emission intensity was observed during a similar acid–base titration of **2** ^**Pz**^ with DMF·HOTf when performed at an excitation wavelength of 515 nm (isosbestic point), the intensity was still insufficient for a lifetime or quantum yield measurement (Figures S66–S69). DFT calculations on **3** ^**Pz**^ and **4** ^**Pz**^ revealed an additional increase in the energy gap between the closed-shell singlet and open-shell triplet states (Δ*E*_CSS–OST_), consistent with enhanced singlet-state stabilization upon protonation. At the same time, the singlet–triplet excited state energy gap (Δ*E*_S1–T1_) decreased, suggesting potential implications for tuning excited state dynamics and photophysical properties through controlled protonation (Figure 8).

**Figure 8:**
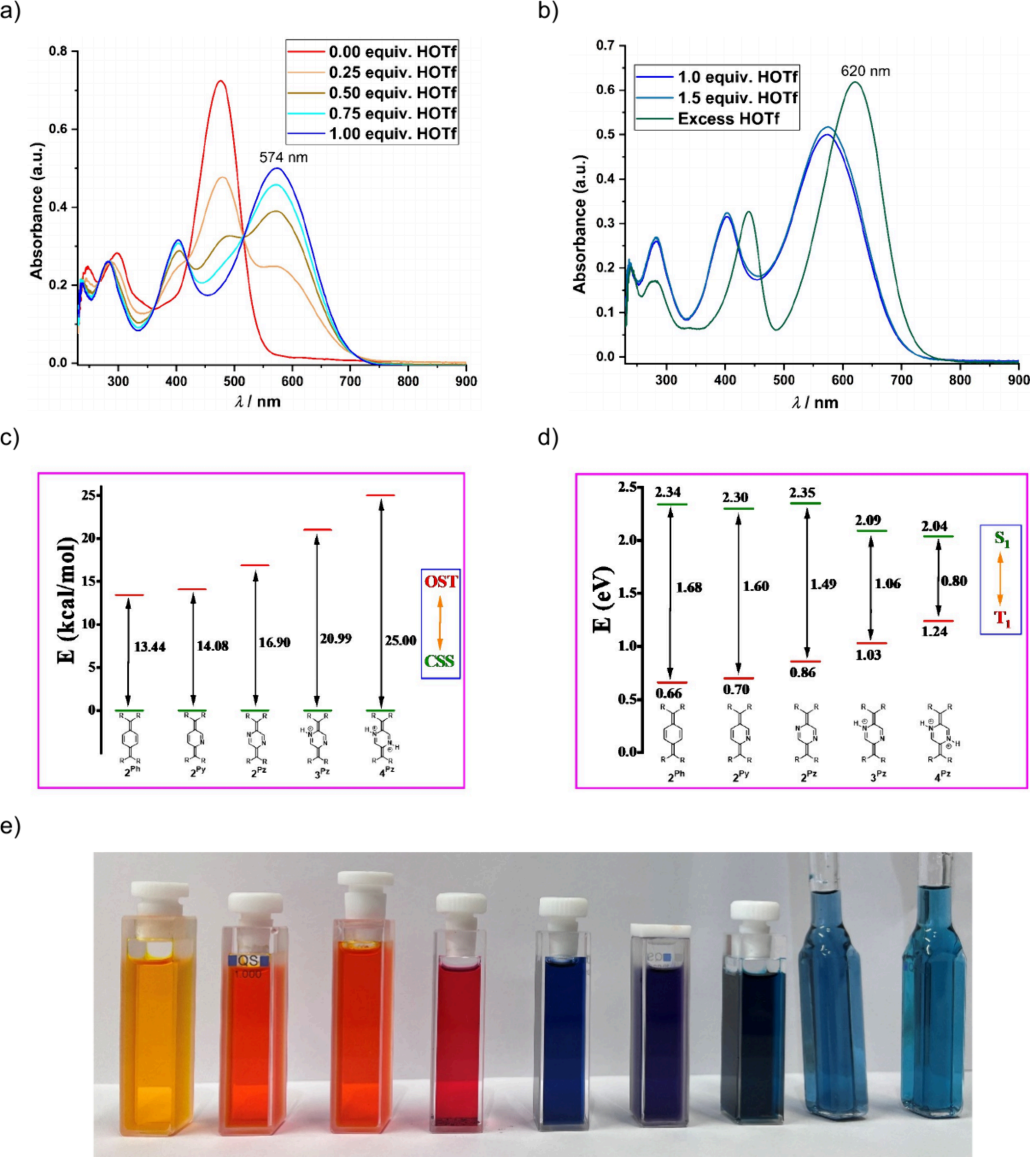
(a) UV–vis–NIR spectra during titration of **2** ^Pz^ with DMF·HOTf to form **3** ^Pz^, measured at room temperature. (b) UV–vis–NIR spectra during titration of **2** ^Pz^ with DMF·HOTf to form **4** ^Pz^ from **3** ^Pz^, measured at room temperature. (c) Calculated energy gap (in kcal/mol) between close shell singlet and open-shell triplet states (Δ*E*_CSS‑OST_). Energy of CSS was considered as 0 for better presentation. (d) Calculated energy gap (in eV) between S_1_ and T_1_ excited states (Δ*E*_S1__–T1_). (e) Photograph of DCM solutions of **2** ^Ph^, **2** ^Py^, **2** ^Pz^, **2** ^Ph^ **DC**, **3** ^Py^, **3** ^Pz^, **4** ^Pz^, **2** ^Py^ **LA**, and **2** ^Pz^ **LA** (from left to right).

To confirm the reversibility of this acid–base coordination process, deprotonation reactions of **3** ^**Py**^ and **4** ^**Pz**^ were performed using DMAP as a base (Scheme 4). In both cases, the starting neutral orange-red diradicaloids **2** ^**Py**^ and **2** ^**Pz**^ were regenerated within a few minutes from corresponding protonated compound **3** ^**Py**^ and **4** ^**Pz**^.

**Scheme 4:**
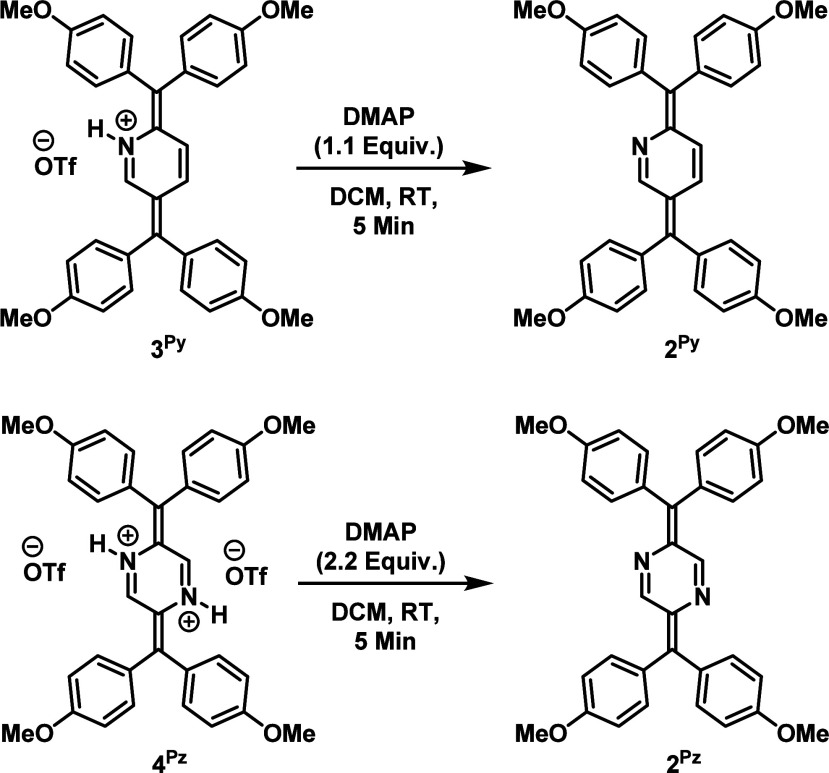
Reaction of **3** ^**Py**^ and **4** ^**Pz**^ with DMAP

A similar observation was made when the reactivity of **2** ^**Py**^ and **2** ^**Pz**^ was tested with Lewis acid tris­(pentafluorophenyl)­borane, B­(C_6_F_5_)_3_. NMR-controlled reactions suggest the formation of the expected Lewis acid adducts **2** ^**Py**^ **LA** and **2** ^**Pz**^ **LA**, respectively (Figures S28–S31). Most likely, due to the dynamic nature of the B–N coordination bond, originating from steric repulsion between aryl end groups and C_6_F_5_ rings,^39^ we were unable to isolate single crystals of these adducts for X-ray diffraction analysis. However, UV–vis–NIR spectroscopy measurements exhibited a similar redshift of the absorption maxima (λ_max_) of these Lewis acids adducts **2** ^**Py**^ **LA** and **2** ^**Pz**^ **LA** compared to the starting diradicaloids **2** ^**Py**^ and **2** ^**Pz**^, indicating significant electronic perturbation upon coordination with the Lewis acid (Figures S60 and S61).

## Conclusions

In conclusion, we have successfully synthesized a series of TH derivatives **2** ^**Ph**^, **2** ^**Py**^, and **2** ^**Pz**^, incorporating an increasing number of nitrogen heteroatoms (0, 1, and 2, respectively). Photophysical and computational studies of these diradicaloids revealed a clear trend in their electronic and excited state properties that correlates with the degree of nitrogen incorporation. A detailed investigation of different oxidation states of **2** ^**Ph**^, which displays potential inversion, was also performed. The intermediate radical cationic state (**2** ^**Ph**^ **RC**) was characterized. This species represents a fascinating category of mixed-valent compounds that display both a very low comproportionation constant (*K*_c_) and very strong electronic coupling at the same time. Although coordination with acids induced minimal structural changes, it produced significant redshifts in the absorption maxima, reflecting substantial perturbation of their electronic properties. These coordination reactions were reversible, and the neutral diradicaloids could be regenerated using a stronger base such as DMAP. Overall, this study demonstrated both internal (heteroatom substitution) and external (Brønsted or Lewis acid coordination) strategies for fine-tuning the properties of classical organic diradicaloid systems. Additionally, the introduction of nitrogen atoms into the diradicaloid backbone provides promising opportunities for their application as ligands in transition metal complexes. Investigations along these directions are currently ongoing in our laboratory.

## Supplementary Material



## Notes

Details of synthesis, NMR crystallography, single-crystal X-ray diffraction, electrochemistry, UV–vis–NIR spectroscopy, EPR spectroscopy, spectroelectrochemistry, emission spectroscopy, transient absorption spectroscopy, and DFT calculations (PDF)
