# Supplementary material for: Stimuli-Responsive Thiele’s Hydrocarbon Derivatives: Potential Inversion, Strong Electronic Coupling, and Influence of Brønsted/Lewis Acids and Bases
Source: JACS Au. 2026 Feb 16;6(3):1654–66. doi: 10.1021/jacsau.5c01512 (PMC13014270; doi:10.1021/jacsau.5c01512)
Supplement: Supplementary file 1 [file au5c01512_si_001.pdf]

Supporting Information for

Stimuli Responsive Thiele's Hydrocarbon Derivatives: Potential Inversion, Strong Electronic Coupling and Influence of Brønsted/Lewis Acids-Bases

Alok Mahata, Nicolás. I. Neuman, Manuel Pech, Jonas Schmidt, Luis I. Domenianni, Peter Vöhringer and Biprajit Sarkar*

A. Mahata, Manuel Pech, Prof. Dr. B. Sarkar

Institut für Anorganische Chemie, Universität Stuttgart, Pfaffenwaldring 55, 70569 Stuttgart, Germany

Prof. Dr. B. Sarkar

Institut für Chemie und Biochemie, Anorganische Chemie, Freie Universität Berlin, Fabeckstraße 34-36, 14195 Berlin, Germany

Email: b.sarkar@fu-berlin.de

Dr. N.I. Neuman

Instituto de Desarrollo Tecnológico para la Industria Química, Colectora Ruta Nacional 168, Km 0, Paraje El Pozo (S3000ZAA) Santa Fe, Argentina

J. Schmidt, Dr. L. I. Domenianni, Prof. Dr. P. Vöhringer

Clausius-Institut für Physikalische und Theoretische Chemie, Rheinische Friedrich-Wilhelms-Universität Bonn, Wegelerstraße 12, 53115 Bonn, Germany

1. Instrumental 2
2. Synthetic procedures 3
3. NMR Spectroscopy.......... 11
4. NMR Spectroscopy controlled reactions.......... 24
5. Crystal structures and crystallographic data.......... 28
6. UV-Vis-NIR Spectroscopy.......... 36
7. Emission Spectroscopy.......... 47
8. Cyclic voltammetry and UV-Vis-NIR Spectro-electrochemistry.......... 50
9. EPR Spectroscopy.......... 55
10. Computational details 57
11. References 94

1. Instrumental

Unless otherwise noted, all reactions were performed using standard Schlenk-line techniques under an inert atmosphere of argon (Linde, Argon 4.8, purity ≥ 99.998) or an MBraun glove box fitted with a gas purification and recirculation unit. Commercially available chemicals were used without further purification. Dimethyl 2,5-pyrazine dicarboxylate, DMF·HOTf, and NBu_4BArF_{24} were synthesized using literature reported procedures.[1] Solvents were available from MBRAUN MB-SPS-800 solvent system and additionally degassed using standard techniques. ^{1}H NMR, ^{13}C NMR, ^{11}B NMR, and ^{19}F NMR were recorded on Bruker 250 and 400 MHz spectrometers. Chemical shifts are reported in ppm (relative to the TMS signal) with reference to the residual solvent peaks.[2] Multiplets are reported as follows: singlet (s), duplet (d), triplet (t), quartet (q), septet (sept), and multiplet (m). NMRs under the exclusion of air were conducted using oven-dried and argon-flushed J. Young's NMR tubes.

Cyclic voltammograms were recorded with a Palm Sens 4 potentiostat in anhydrous and degassed DCM with 0.1 м NBu_4PF_6 (dried, > 99.0%, electrochemical grade, Fluka) or 0.02 м NBu_4BArF_{24} as supporting electrolyte. Concentrations of the compounds were about $1\cdot10^{-4}$ M. A three-electrode setup was used with a glassy carbon working electrode, a coiled platinum wire as a counter electrode, and a coiled silver wire as a pseudo-reference electrode. The ferrocene/ferrocenium couple was used as an internal reference. UV/Vis spectra were recorded on J&M TIDAS, Avantas, or Jasco UV-Vis-NIR spectrophotometer. UV/vis spectroelectrochemical measurements were carried out in an optically transparent thin-layer electrochemical (OTTLE)[3] cell (CaF_2 windows) with a gold mesh working electrode, a platinum-mesh counter electrode, and a silver-foil pseudo-reference. EPR spectra at the X-band frequency (ca. 9.5 GHz) were obtained with a Magnettech MS 5000 benchtop EPR spectrometer equipped with a rectangular TE 102 cavity and a TC HO4 temperature controller. The measurements were carried out in synthetic quartz glass tubes. Emission measurements of $\mathbf{2^{Pz}}$ at different acid concentrations were carried out using a Shimadzu RF-6000 spectrofluorophotometer and other measurements using a commercial fluorescence spectrometer (Edinburgh Instruments FS5) equipped with a cryostat (Oxford Instruments Optistat DN). A fluorescence cuvette (Hellma QS) with a path length of 10 mm was used. Solutions of $\mathbf{2^{Ph}}$, $\mathbf{2^{Py}}$, $\mathbf{2^{Pz}}$, $\mathbf{3^{Py}}$, as well as $\mathbf{3^{Pz}}$ in spectroscopic grade DCM (99.8%, Thermo Scientific) were prepared to achieve an absorbance of 0.3 OD at the given excitation wavelengths. Solutions were thermally equilibrated for a period of 30 minutes prior to each measurement at the given temperatures. The fluorescence spectrometer was set to a bandwidth of 5 nm for excitation, 2 nm for emission, a dwell time of 5 s, and a step size of 2 nm.

X-ray data were collected on a Bruker Kappa Apex Duo system at 295(2), 160(2), 110(2), or 100(2) K, using graphite-monochromated MoKα radiation (λα= 0.71073 Å). The strategy for the data collection was evaluated by using the Smart software. The data were collected by the standard omega scan or omega + phi scan techniques, and were scaled and reduced using Saint+ and SADABS software. The structures were solved by direct methods using SHELXS-97 or intrinsic phasing using SHELXL-2014/7 and refined by full matrix least-squares, refining on F2. Non-hydrogen atoms were refined anisotropically.[4-6] Structures were solved using SHELXS-97 and the software OLEX2, while refinement was carried out on F2 against all independent reflections by the full matrix least-squares method using the SHELXL-97 program. All non-hydrogen atoms were refined using anisotropic thermal parameters.[4-6]

Transient Absorption Spectroscopy: Femtosecond UV-pump/visible-probe transient absorption measurements were carried out using a commercial Ti:sapphire oscillator–regenerative amplifier laser system (Newport Spectra-Physics Solstice Ace). This front-end system delivered 800 nm–centered pulses with a duration of ~60 fs at a repetition rate of 1 kHz, which were used to synchronously pump two optical parametric amplifiers (OPA, TOPAS Prime, Light Conversion). The first OPA was pumped with 330 mW average power and tuned to 1250 nm. A small fraction of its signal output was focused into a rotating CaF_2 substrate to generate a white-light continuum (WLC) probe with a usable spectral bandwidth spanning 380–950 nm. The second optical parametric amplifier, was pumped by a beam with an average power of 1.1 W. The second OPA was pumped with 1.1 W average power, and UV pump pulses were produced via fourth-harmonic generation of its idler output. Temporal overlap between pump and probe pulses was controlled using a motorized optical delay stage in the probe beam path. The white-light probe beam was focused into the sample, and subsequently directed to a polychromator equipped with a CCD array detector. The pump beam was focused into the sample using a fused-silica lens with a focal length of 400 mm at an angle of 5° relative to the probe beam. To ensure homogeneous excitation, the

pump focus was positioned slightly behind the sample, resulting in a spot diameter (~400 μm) larger than that of the probe. The sample solution was circulated using a gear pump through a commercial flow-through quartz cell (Hellma, QS) with an optical path length of 1 mm.

Linear Absorption spectroscopy: Absorption spectra of solutions of **2**^{Py} in spectroscopic grade DCM (99.8 %, Thermo Scientific) and spectroscopic grade toluene (≥99.9 %, Uvasol®) were carried out using a Perkin Elmer Lambda 365+ UV/Vis spectrometer and Hellma QS cuvettes (1 mm and 10 mm pathlengths). The spectra of the measured sample solutions were referenced against the respective solvent spectra and the spectrometer was set to a scan speed of 240 nm/min, 1 nm slit width, and 1 nm data interval.

2. Synthetic procedures

Synthesis of **1**^{Ph}

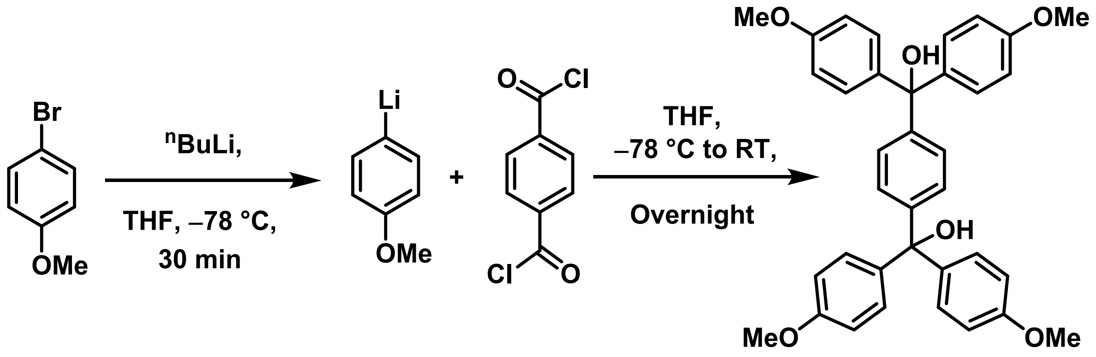

1^{Ph}

1^{Ph} was synthesized following a slightly modified literature reported procedure.[7] A solution of 4-bromoanisole (1.25 mL, 10 mmol) in dry THF (40 mL) was charged in a 100 mL Schlenk flask under argon atmosphere. The solution was cooled down to –78 °C and n-butyllithium (4 mL, 10 mmol, 2.5 M in hexane) was added dropwise. After the addition was completed, the reaction mixture was stirred for 30 minutes at the same temperature. Then a solution of terephthaloyl chloride (0.4 g, 2 mmol) in dry THF (20 mL) was added dropwise. The formed reaction mixture was allowed to warm to room temperature and stirred overnight. 20mL NH_4Cl solution was added to quench the reaction. The suspension was extracted three times with ethyl acetate (3 × 100 mL), the combined organic phases were dried over Na_2SO_4 and the solvent was removed under reduced pressure. The remaining light yellow oil was re-dissolved in 20 mL ethyl acetate and 60 mL Hexane was added. The mixture was triturated for 5 minutes, the precipitate formed was filtered , washed with 30 mL hexane and dried under vacuum to get pure **1**^{Ph} as an off-white solid. Yield: 564 mg (1.0 mmol, 50%).

^{1}H-NMR ($CDCl_3$, 250 MHz) *δ*: 7.20 – 7.15 (mixture of d, 8H, Ar-*H*, and s, 4H, Ph-*H*), 6.82 (d, 10Hz, 8H, Ar-*H*), 3.79 (s, 12H, O-C*H*$_3$), 2.76 (bs, 2H, O*H*) ppm.
^{13}C{^{1}H}-NMR ($CDCl_3$, 63 MHz) *δ*: 158.7, 146.2, 139.5, 129.2, 127.4, 113.3, 81.4, 55.4 ppm.
EA: calc. ($C_{36}H_{34}O_6$, 562.66 g/mol) C 76.85 H 6.09; found C 76.38 H 6.01
MS(ESI): m/z = 545.23 $[M\text{-}OH^-]^+$.
HRMS(ESI): calc ($C_{36}H_{33}O_5$) m/z = 545.2323; found m/z = 545.2309.

Synthesis of **1**^{Py}

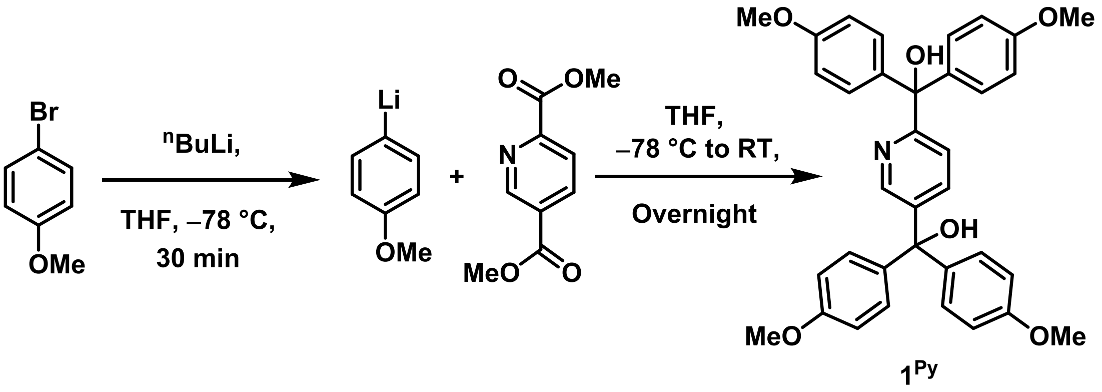

A solution of 4-bromoanisole (1.25 mL, 10 mmol) in dry THF (40 mL) was charged in a 100 mL Schlenk flask under argon atmosphere. The solution was cooled down to –78 °C and n-butyllithium (4 mL, 10 mmol, 2.5 M in hexane) was added dropwise. After the addition was completed, the reaction mixture was stirred for 30 minutes at same temperature. Then a solution of Dimethyl 2,5-pyridine dicarboxylate (0.39 g, 2 mmol) in dry THF (20 mL) was added dropwise. The formed reaction mixture was allowed to warm to room temperature and stirred overnight. 20mL NH_4Cl solution was added to quench the reaction. The suspension was extracted three times with ethyl acetate (3 × 100 mL), the combined organic phases were dried over Na_2SO_4 and the solvent was removed under reduced pressure. The remaining brown oil was redissolved in 20 mL ethyl acetate and 60 mL Hexane was added. The mixture was triturated for 10 minutes, the formed precipitate was filtered, washed with 30 mL hexane and dried under vacuum to get pure $\mathbf{1^{Py}}$ as an off-white solid. Yield: 541 mg (0.96 mmol, 48%).

^{1}H-NMR ($CDCl_3$, 250 MHz) *δ*: 8.47 (s, 1H, Py-*H*), 7.62 (d, 7.5 Hz, 1H, Py-*H*), 7.20 – 6.84 (m, 8H, Ar-*H*, and 1H, Py-*H*), 6.84 (bs, 8H, Ar-*H*), 6.16 (bs, 1H, O*H*), 3.79 (s, 12H, O-C*H*$_3$), 2.91 (s, 1H, O*H*) ppm.
^{13}C{^{1}H}-NMR ($CDCl_3$, 63 MHz) *δ*: 162.2, 159.1, 158.9, 146.7, 141.9, 138.4, 138.3, 136.6, 129.4, 129.1, 122.2, 113.7, 113.4, 80.3, 80.1, 55.43, 55.38 ppm.
EA: calc. ($C_{35}H_{33}NO_6$, 563.65 g/mol) C 74.58 H 5.90 N 2.49; found C 74.36 H 5.89 N 2.45
MS(ESI): m/z = 564.24 $[M+H]^+$.
HRMS(ESI): calc ($C_{35}H_{34}NO_6$) m/z = 564.2381; found m/z = 564.2364.

Synthesis of $\mathbf{1^{Pz}}$

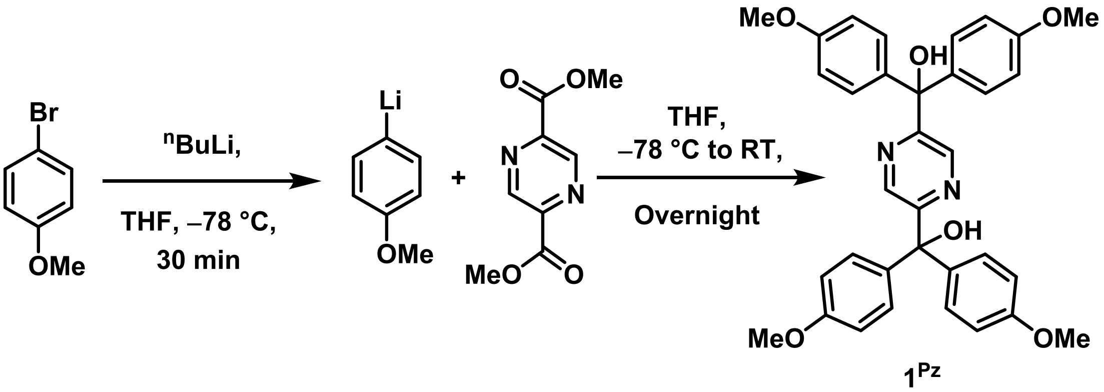

A solution of 4-bromoanisole (1.25 mL, 10 mmol) in dry THF (40 mL) was charged in a 100 mL Schlenk flask under argon atmosphere. The solution was cooled down to –78 °C and n-butyllithium (4 mL, 10 mmol, 2.5 M in hexane) was added dropwise. After the addition was completed, the reaction mixture was stirred for 30 minutes at the same temperature. Then a solution of Dimethyl 2,5-pyrazine dicarboxylate (0.39 g, 2 mmol) in dry THF (20 mL) was added dropwise. The reaction mixture was allowed to warm to room temperature and stirred overnight. 20mL NH_4Cl solution was added to quench the reaction. The suspension was extracted three times with ethyl acetate (3 × 100 mL), the combined organic phases were

dried over Na_2SO_4 and the solvent was removed under reduced pressure. The remaining light yellow solid was washed with 10 mL ethyl acetate and dried under vacuum to get pure **1^{Pz}** as an off-white solid. Yield: 510 mg (0.90 mmol, 45%).

^{1}H-NMR ($CDCl_3$, 250 MHz) *δ*: 8.39 (s, 2H, Pz-*H*), 7.18 (d, 7.5 Hz, 8H, Ar-*H*), 6.86 (d, 7.5 Hz, 8H, Ar-*H*), 5.10 (bs, 2H, O*H*), 3.80 (s, 12H, O-C*H$_3$*) ppm.
^{13}C{^{1}H}-NMR ($CDCl_3$, 63 MHz) *δ*: 159.2, 158.0, 141.9, 137.4, 129.3, 113.7, 79.6, 55.4 ppm.
EA: calc. ($C_{34}H_{32}N_2O_6$, 564.64 g/mol) C 72.32 H 5.81 N 4.96; found C 72.90 H 5.75 N 4.89
MS(ESI): m/z = 565.23 $[M+H]^+$.
HRMS(ESI): calc ($C_{34}H_{33}N_2O_6$) m/z = 565.2333; found m/z = 565.2315.

Synthesis of **2^{Ph}**

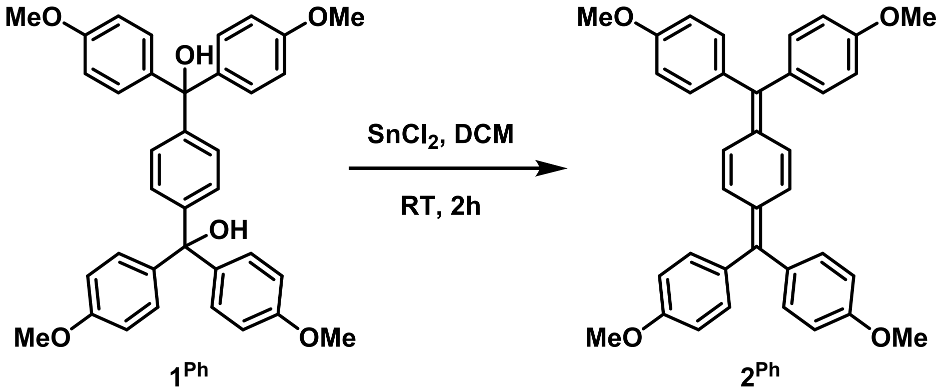

A mixture of **1^{Ph}** (450 mg, 0.8 mmol, 1 equiv.) and $SnCl_2$ (455 mg, 2.4 mmol, 3 equiv.) was added to an oven-dried Schlenk flask and dissolved in dry DCM (20 mL). The reaction mixture was stirred at room temperature for two hours. During this time, the color of the mixture changed from light yellow to orange-red. All the volatiles were removed and the product was purified using flash chromatography using DCM as eluent. The solvent was removed using a rotary evaporator. The obtained solid was further washed with 10mL acetone and dried under vacuum to get pure **2^{Ph}** as an orange solid. Yield: 175 mg (0.33 mmol, 41%).

^{1}H-NMR ($CDCl_3$, 250 MHz) *δ*: 7.16 (d, 7.5Hz, 8H, Ar-*H*), 6.85 (d, 7.5Hz, 8H, Ar-*H*), 6.75 (s, 4H, Ph-*H*), 3.82 (s, 12H, O-C*H$_3$*) ppm.
^{13}C{^{1}H}-NMR ($CDCl_3$, 63 MHz) *δ*: 159.0, 135.0, 132.7, 132.0, 128.6, 113.5, 55.4 ppm.
^{1}H-NMR (CD_2Cl_2, 250 MHz) *δ*: 7.13 (bs, 8H, Ar-*H*), 6.86 (d, 7.5Hz, 8H, Ar-*H*), 6.71 (s, 4H, Ph-*H*), 3.81 (s, 12H, O-C*H$_3$*) ppm.
EA: calc. ($C_{36}H_{32}O_4$, 528.65 g/mol) C 81.79 H 6.10; found C 81.76 H 6.13
MS(ESI): m/z = 529.24 $[M+H]^+$.
HRMS(ESI): calc ($C_{36}H_{33}O_4$) m/z = 529.2373; found m/z = 529.2360.

Synthesis of **2^{Py}**

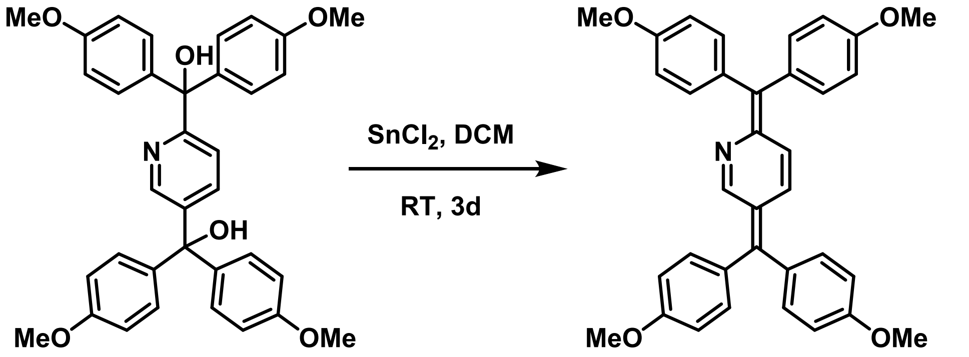

1^{Py} **2**^{Py}

A mixture of **1**^{Py} (451 mg, 0.8 mmol, 1 equiv.) and $SnCl_2$ (455 mg, 2.4 mmol, 3 equiv.) was added to an oven-dried Schlenk flask and dissolved in dry DCM (20 mL). The reaction mixture was stirred at room temperature for two hours. During this time, the color of the mixture changed from light yellow to blue-violet. 50 mL saturated aqueous NH_4Cl solution was added to the reaction mixture. The mixture was extracted with DCM (3 x 100 mL), the organic phases were combined and washed with saturated 100mL $NaHCO_3$ solution. The color of the organic phase turned to red, and it was further washed with Brine solution. The combined organic ~~phased~~ phases ~~was~~ were dried over anhydrous $MgSO_4$ and the solvent was evaporated using a rotary evaporator. The obtained solid was further washed with 10mL Acetone and dried under vacuum to get pure **2**^{Py} as an orange-red solid. Yield: 132 mg (0.25 mmol, 31%).

^{1}H-NMR ($C_2D_2Cl_4$, 250 MHz) *δ*: 8.32 (s, 1H, Py-*H*), 7.38 (d, 7.5Hz, 2H, Ar-*H*), 7.24 – 7.18 (m, 6H, Ar-*H*), 6.91 – 6.75 (m, 8H, Ar-*H*, and 1H, Py-*H*), 6.64 (d, 7.5Hz, 1H, Py-*H*), 3.85 – 3.82 (three s, 12H, O-C*H₃*) ppm.
^{13}C{^{1}H}-NMR ($C_2D_2Cl_4$, 63 MHz) *δ*: 159.6, 159.4, 158.8, 158.6, 157.0, 141.3, 141.2, 137.3, 134.0, 133.7, 133.6, 133.2, 132.8, 132.6, 132.3, 127.4, 126.8,125.6, 113.5, 113.4, 112.7, 55.4, 55.3 ppm.
^{1}H-NMR (CD_2Cl_2, 250 MHz) *δ*: 8.26 (d, 2.5Hz, 1H, Py-*H*), 7.30 (d, 7.5Hz, 2H, Ar-*H*), 7.21 – 7.15 (m, 6H, Ar-*H*), 6.91 – 6.86 (m, 6H, Ar-*H*), 6.80 (d, 7.5Hz, 2H, Ar-*H*), 6.73 (dd, 7.5Hz, 2.5Hz, 1H, Py-*H*), 6.63 (d, 7.5Hz, 1H, Py-*H*), 3.83 – 3.80 (four s, 12H, O-CH_3) ppm.
EA: calc. ($C_{35}H_{31}NO_4$, 529.64 g/mol) C 79.37 H 5.90 N 2.64; found C 77.48 H 5.89 N 2.64
MS(ESI): m/z = 530.23 $[M+H]^+$.
HRMS(ESI): calc ($C_{35}H_{32}NO_4$) m/z = 530.2326; found m/z = 530.2321.

Synthesis of **2**^{Pz}

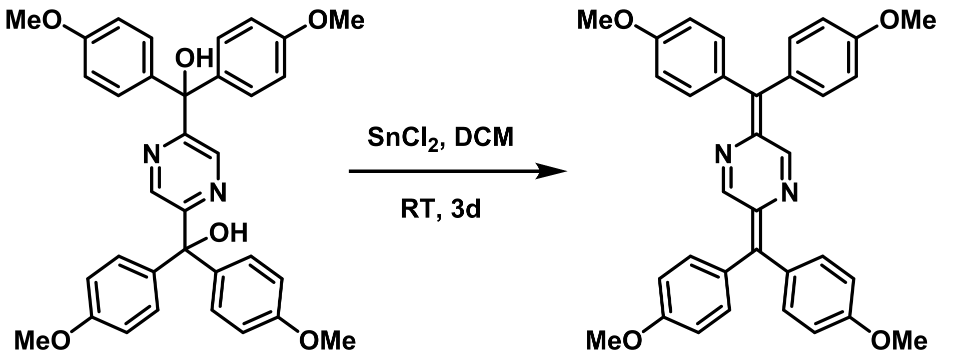

1^{Pz} **2**^{Pz}

A mixture of **1**^{Pz} (452 mg, 0.8 mmol, 1 equiv.) and $SnCl_2$ (455 mg, 2.4 mmol, 3 equiv.) was added to an oven-dried Schlenk flask and dissolved in dry DCM (20 mL). The reaction mixture was stirred at room temperature for two hours. During this time, the color of the mixture changed from light yellow to blue-violet. 50 mL saturated aqueous NH_4Cl solution was added to the reaction mixture. The mixture was extracted with DCM (3 x 100 mL), the organic phases were combined and washed

with saturated 100mL $NaHCO_3$ solution. The color of the organic phase turned to red, and it was further washed with Brine solution. The combined organic phases were dried over anhydrous $MgSO_4$ and the solvent was evaporated using a rotary evaporator. The obtained solid was further washed with 10mL acetone and dried under vacuum to get pure **2^{Pz}** as an orange-red solid. Yield: 145 mg (0.27 mmol, 34%).

^{1}H-NMR ($C_2D_2Cl_4$, 250 MHz) *δ*: 8.16 (s, 2H, Pz-*H*), 7.42 (d, 7.5 Hz, 4H, Ar-*H*), 7.26 (d, 7.5 Hz, 4H, Ar-*H*), 6.92 (d, 7.5 Hz, 4H, Ar-*H*), 6.86 (d, 7.5 Hz, 4H, Ar-*H*), 3.86 (s, 6H, O-C*H$_3$*), 3.83 (s, 6H, O-C*H$_3$*) ppm.

^{13}C{^{1}H}-NMR ($C_2D_2Cl_4$, 63 MHz) *δ*: 159.8, 159.4, 155.0, 141.1, 135.6, 113.7, 132.9, 132.1, 131.8, 113.6, 112.9, 55.4, 55.3 ppm.

^{1}H-NMR (CD_2Cl_2, 250 MHz) *δ*: 8.12 (s, 2H, Pz-*H*), 7.35 (d, 10 Hz, 4H, Ar-*H*), 7.24 (d, 7.5 Hz, 4H, Ar-*H*), 6.92 (d, 7.5 Hz, 4H, Ar-*H*), 6.83 (d, 10 Hz, 4H, Ar-*H*), 3.84 (s, 6H, O-C*H$_3$*), 3.81 (s, 6H, O-C*H$_3$*) ppm.

EA: calc. ($C_{34}H_{30}N_2O_4$, 530.62 g/mol) C 76.96 H 5.70 N 5.28; found C 76.27 H 5.82 N 4.92

MS(ESI): m/z = 531.23 $[M+H]^+$.

HRMS(ESI): calc ($C_{34}H_{31}N_2O_4$) m/z = 531.2278; found m/z = 531.2277.

Synthesis of **2^{Ph}DC**

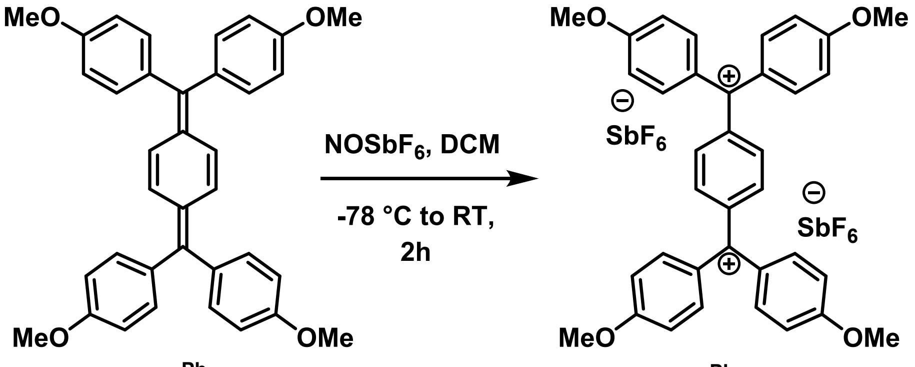

2^{Ph} 2^{Ph}DC

A mixture of **2^{Ph}** (52 mg, 0.1 mmol, 1 equiv.) and $NOSbF_6$ (55 mg, 0.21 mmol, 2.1 equiv.) was added to an oven-dried Schlenk flask and 10 mL DCM was added at –78 °C. After addition, the reaction mixture was allowed to warm to room temperature and stirred for two hours. During the reaction, the color of the mixture changed from orange to green and finally pink-red. All the volatiles were removed and the obtained product was further washed with 10mL Et_2O and dried under vacuum to get pure **2^{Ph}DC** as a metallic-green solid. Yield: 93 mg (0.093 mmol, 93%). Because of the low solubility of the product, we were not able to get a well resolved ^{13}C NMR spectrum.

^{1}H-NMR (CD_2Cl_2, 250 MHz) *δ*: 7.80 (d, 7.5Hz, 8H, Ar-*H*), 7.74 (s, 4H, Ph-*H*), 7.41 (d, 7.5Hz, 8H, Ar-*H*), 4.18 (s, 12H, O-C*H$_3$*) ppm.

EA: calc. ($C_{36}H_{32}F_{12}O_4Sb_2$, 1000.15 g/mol) C 43.23 H 3.23; found C 43.15 H 3.25

MS(ESI): m/z = 264.11 $[M - 2SbF_6^{2-}]^{++}$.

HRMS(ESI): calc $(C_{36}H_{32}O_4)^{++}$ m/z = 264.1145; found m/z = 264.1141.

Disproportionation reaction of **2^{Ph}** and **2^{Ph}DC** to form **2^{Ph}RC**

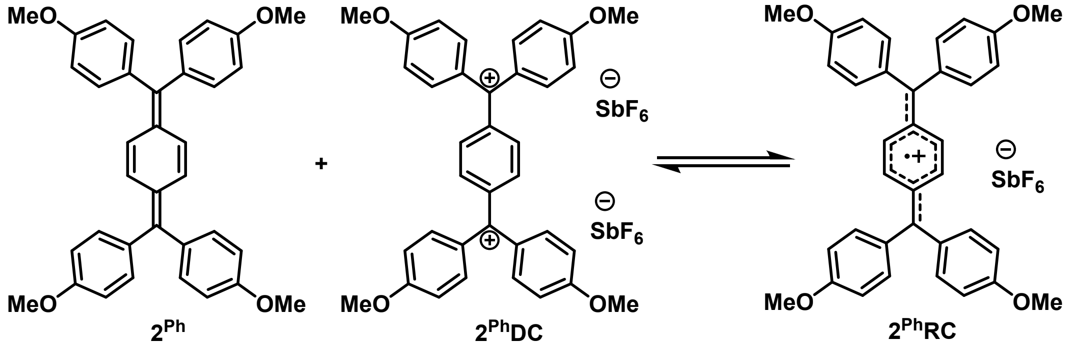

A mixture of **2^{Ph}** (27 mg, 0.05 mmol, 1 equiv.) and **2^{Ph}DC** (50 mg, 0.05 mmol, 1 equiv.) was added to an oven-dried Schlenk flask and 10 mL DCM was added. The color of the mixture changed from orange to green immediately. All the volatiles were removed under vacuum and the obtained solid was used to characterize **2^{Ph}RC** using EPR and UV-vis-NIR Spectroscopy (Figure S49-S54, S78).

Synthesis of **3^{Py}**

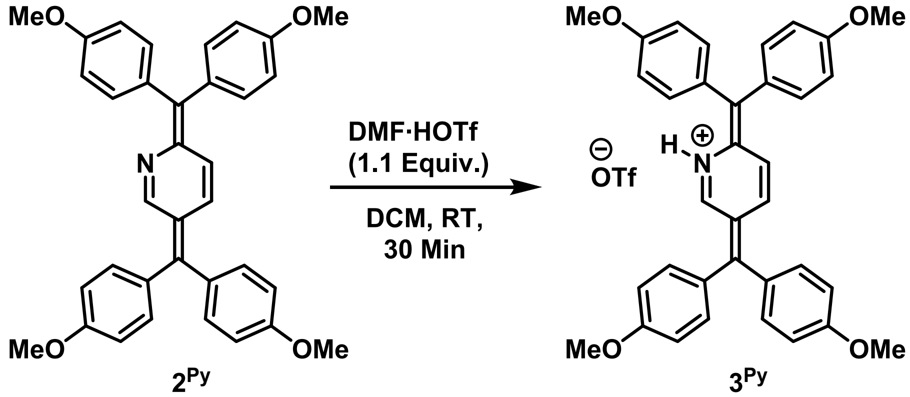

A mixture of **2^{Py}** (106 mg, 0.2 mmol, 1 equiv.) and DMF·HOTf (49 mg, 0.22 mmol, 1.1 equiv.) was added to an oven-dried Schlenk flask and dissolved in dry DCM (10 mL). An immediate color change from orange-red to blue was observed. The reaction mixture was stirred at room temperature for 30 minutes. 30 mL Et_2O was added and resulting dark blue to black precipitate was filtered. The obtained solid was further washed with 10mL Et_2O and dried under vacuum to get pure **3^{Py}** as a dark blue solid. Yield: 88 mg (0.13 mmol, 65%).

^{1}H-NMR (CD_3CN, 250 MHz) *δ*: 11.18 (bs, 1H, N-*H*), 7.85 (d, 2.0Hz, 1H, Py-*H*), 7.33 – 7.17 (m, 8H, Ar-*H*), 7.06 – 6.95 (m, 8H, Ar-*H*), 6.88 (dd, 10Hz, 2Hz, 1H, Py-*H*), 6.78 (d, 10Hz, 1H, Py-*H*), 3.88 – 3.82 (four s, 12H, O-C*H₃*) ppm.
^{13}C{^{1}H}-NMR (CD_3CN, 63 MHz) *δ*: 164.2, 163.7, 162.0, 161.7, 159.7, 158.7, 135.3, 135.1, 134.6, 133.3, 133.1, 132.0, 131.9, 131.7, 131.5, 130.4, 129.8, 126.2,124.9, 115.9, 115.4, 115.2, 115.0, 56.4, 56.2, 56.1 ppm.
^{19}F-NMR (CD_3CN, 376 MHz) *δ*: -79.3 ppm.
EA: calc. ($C_{36}H_{32}F_3NO_7S$, 679.71 g/mol) C 63.62 H 4.75 N 2.06; found C 61.89 H 4.75 N 2.28
MS(ESI): m/z = 530.23 [M-OTf]$^+$.
HRMS(ESI): calc ($C_{35}H_{32}NO_4$) m/z = 530.2326; found m/z = 530.2317.

Synthesis of **3^{Pz}**

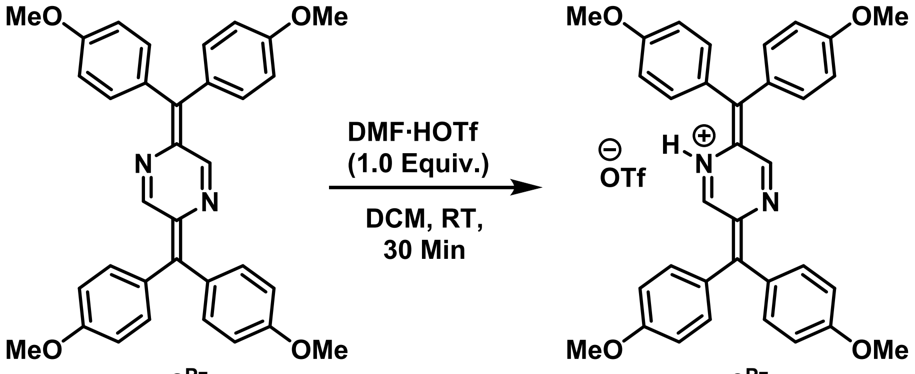

2^{Pz} **3^{Pz}**

A mixture of **2^{Pz}** (106 mg, 0.2 mmol, 1 equiv.) and DMF·HOTf (45 mg, 0.2 mmol, 1.0 equiv.) was added to an oven-dried Schlenk flask and dissolved in dry DCM (10 mL). An immediate color change from orange-red to blue was observed. The reaction mixture was stirred at room temperature for 30 minutes. 30 mL Et_2O was added and the resulting precipitate was filtered. The obtained solid was further washed with 10mL Et_2O and dried under vacuum to get pure **3^{Pz}** as a dark blue solid. Yield: 79 mg (0.116 mmol, 58%).

^{1}H-NMR (CD_3CN, 250 MHz) δ: 11.41 (bs, 1H, N-*H*), 7.94 (s, 2H, Pz-*H*), 7.43 – 7.33 (m, 8H, Ar-*H*), 7.08 – 7.04 (m, 8H, Ar-*H*), 3.88 (s, 12H, O-C*H₃*) ppm.

^{13}C{^{1}H}-NMR (CD_3CN, 63 MHz) δ: 163.7, 163.6, 154.6, 134.9, 134.6, 130.4, 130.2, 115.4, 56.4 ppm.

^{19}F-NMR (CD_3CN, 376 MHz) δ: -79.3 ppm.

EA: calc. ($C_{35}H_{31}F_3N_2O_7S$, 680.70 g/mol) C 61.76 H 4.59 N 4.12; found C 59.68 H 4.86 N 3.82.

MS(ESI): m/z = 531.23 $[M-OTf^-]^+$.

HRMS(ESI): calc ($C_{34}H_{31}N_2O_4$) m/z = 531.2278; found m/z = 531.2271.

Synthesis of **4^{Pz}**

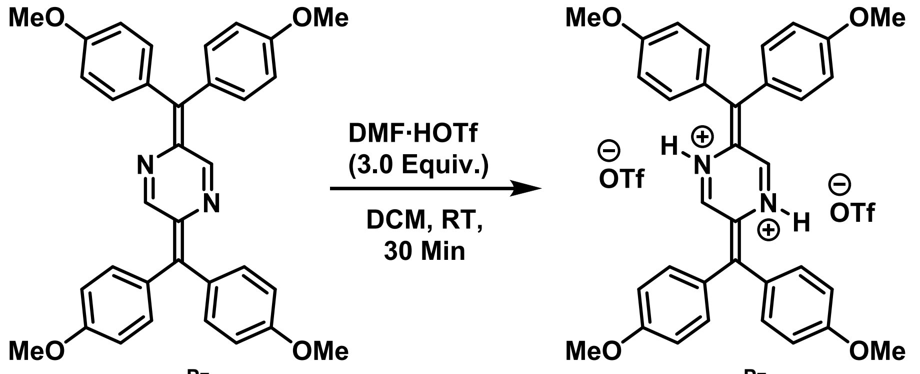

2^{Pz} **4^{Pz}**

A mixture of **2^{Pz}** (106 mg, 0.2 mmol, 1 equiv.) and DMF·HOTf (135 mg, 0.6 mmol, 3.0 equiv.) was added to an oven-dried Schlenk flask and dissolved in dry DCM (10 mL). An immediate color change from orange-red to deep green was observed. The reaction mixture was stirred at room temperature for 30 minutes. 10 mL Et_2O was added and the resulting precipitate was filtered. The obtained solid was further washed with 10mL Et_2O and dried under vacuum to get pure **4^{Pz}** as a dark green solid. Yield: 151 mg (0.182 mmol, 92%).

^{1}H-NMR (CD_3CN, 250 MHz) δ: 11.87 (bs, 2H, N-*H*), 7.92 (s, 2H, Pz-*H*), 7.51 – 7.41 (m, 8H, Ar-*H*), 7.15 – 7.10 (m, 8H, Ar-*H*), 3.92 (s, 12H, O-C*H₃*) ppm.

$^{13}C\{^1H\}$-NMR (CD_3CN, 63 MHz) δ: 166.2, 165.6, 158.0, 157.0, 135.8, 135.1, 129.5, 128.4, 125.2, 116.4, 116.1, 56.8, 56.7 ppm.

^{19}F-NMR (CD_3CN, 376 MHz) δ: -79.3 ppm.

EA: calc. ($C_{36}H_{32}F_6N_2O_{10}S_2$, 830.77 g/mol) C 52.05 H 3.88 N 3.37; found C 52.20 H 3.88 N 3.56.

MS(ESI): m/z = 531.23 [M-HOTf-OTf$^-$]$^+$.

HRMS(ESI): calc ($C_{34}H_{31}N_2O_4$) m/z = 531.2278; found m/z = 531.2271.

Alternative synthesis of **3^{Pz}** by comproportionation reaction of **2^{Pz}** and **4^{Pz}**

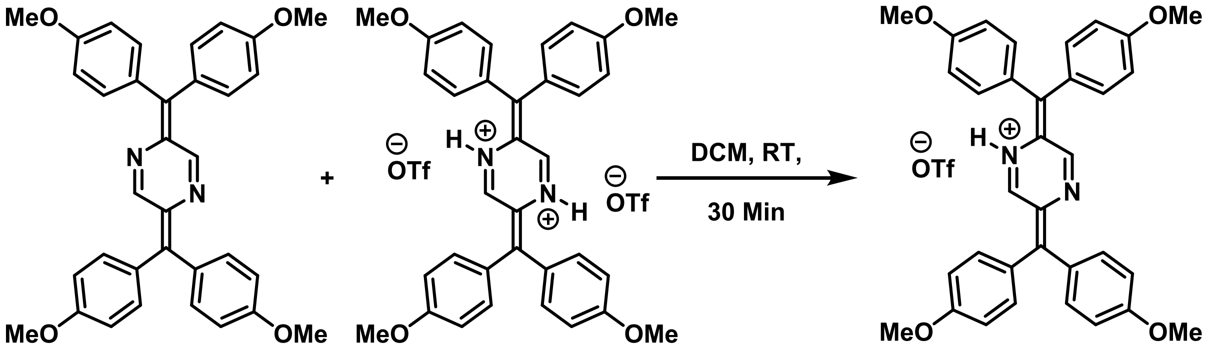

2^{Pz} **4^{Pz}** **3^{Pz}**

A mixture of **2^{Pz}** (53 mg, 0.1 mmol, 1 equiv.) and **4^{Pz}** (83 mg, 0.1 mmol, 1 equiv.) was added to an oven-dried Schlenk flask and dissolved in dry DCM (10 mL). The color of the reaction mixture turned blue. The reaction mixture was stirred at room temperature for 30 minutes. All the volatiles were removed, the obtained solid was washed with 20mL Et_2O and dried under vacuum to get pure **3^{Pz}** as a dark blue solid. Yield: 113 mg (0.166 mmol, 83%).

Reaction of **3^{Py}** with DMAP

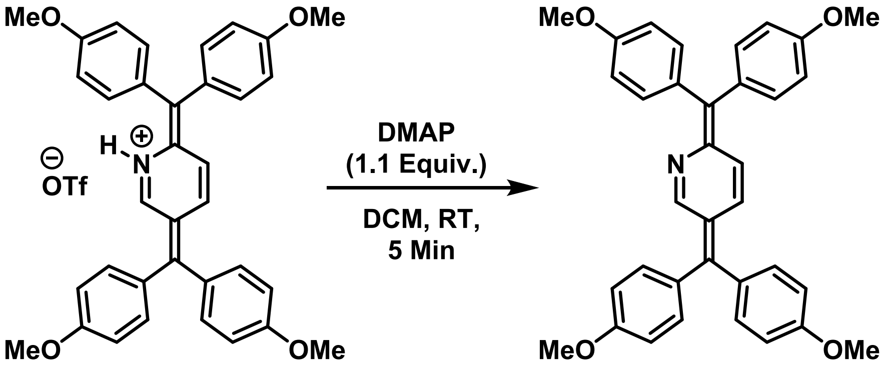

3^{Py} **2^{Py}**

A mixture of **3^{Py}** (136 mg, 0.2 mmol, 1 equiv.) and 4-dimethylaminopyridine (27 mg, 0.22 mmol, 1.1 equiv.) was added to an oven-dried Schlenk flask and dissolved in dry DCM (10 mL). The color of the reaction mixture changed from blue to orange-red. The reaction mixture was stirred at room temperature for 5 minutes. All the volatiles were removed and 5 mL acetone was added. The obtained precipitate was filtered, washed with 5 mL acetone remove DMAP·HOTf and excess DMAP, and dried under vacuum to get back pure **2^{Py}** as an orange-red solid. Yield: 87 mg (0.164 mmol, 82%).

Reaction of **4^{Pz}** with DMAP

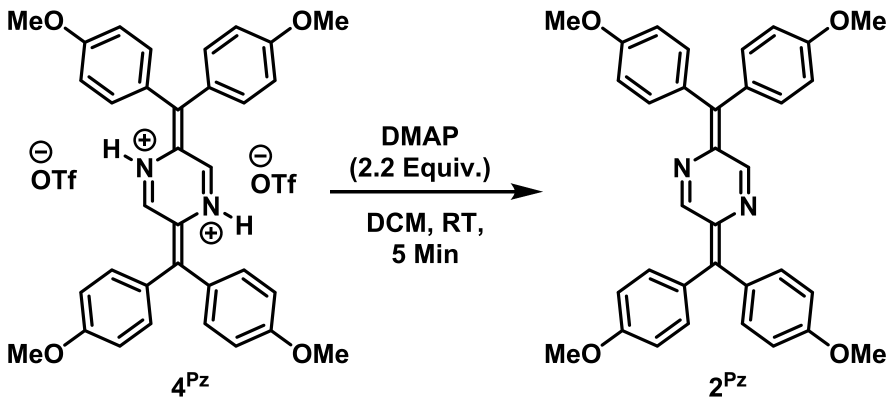

A mixture of **4**^{Pz} (166 mg, 0.2 mmol, 1 equiv.) and 4-dimethylaminopyridine (54 mg, 0.44 mmol, 2.2 equiv.) was added to an oven-dried Schlenk flask and dissolved in dry DCM (10 mL). The color of the reaction mixture changed from blue to orange-red. The reaction mixture was stirred at room temperature for 5 minutes. All the volatiles were removed and 5 mL acetone was added. The obtained precipitate was filtered, washed with 5 mL acetone to remove DMAP.HOTf and excess DMAP, and dried under vacuum to get back pure **2**^{Pz} as an orange-red solid. Yield: 96 mg (0.18 mmol, 90%).

3. NMR Spectroscopy

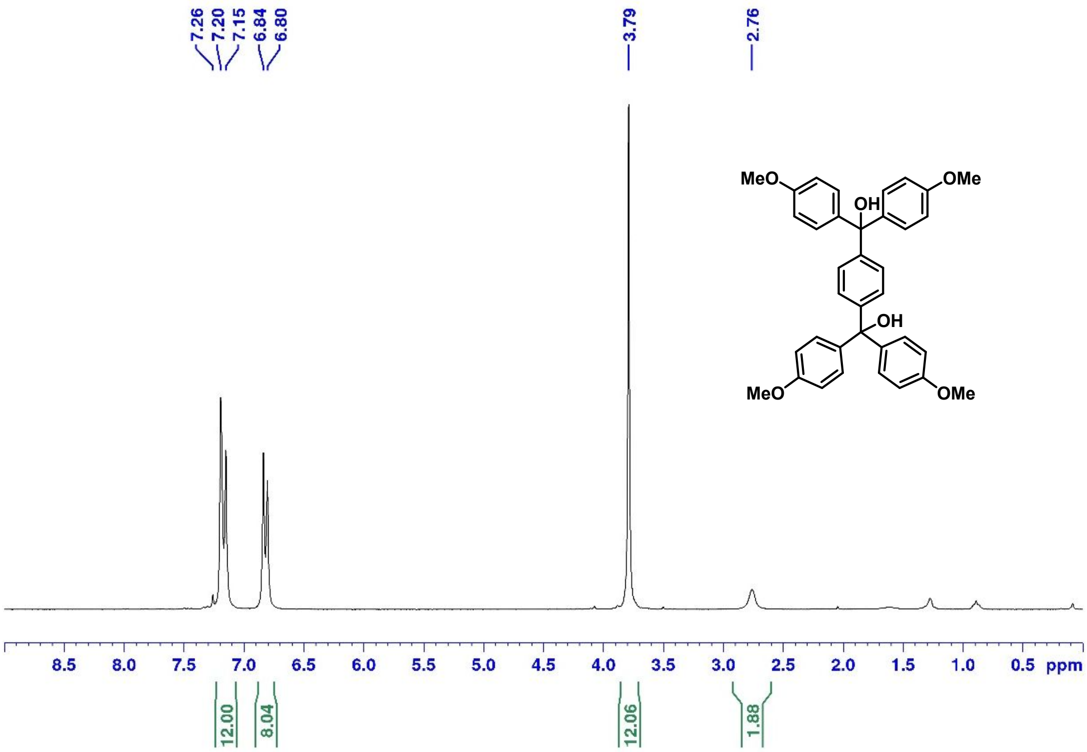

Figure S1. ^{1}H-NMR-Spectrum of **1**^{Ph} ($CDCl_3$, 250 MHz).

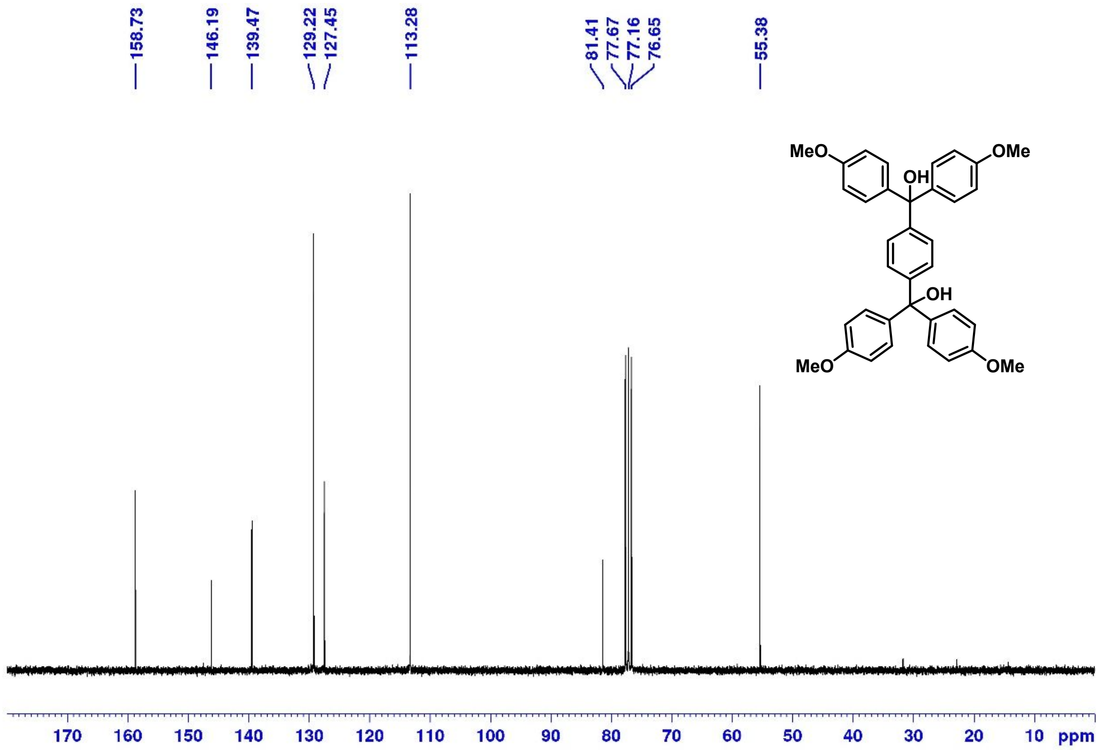

Figure S2. $^{13}C\{^{1}H\}$-NMR-Spectrum of **1[Ph]** ($CDCl_3$, 63 MHz).

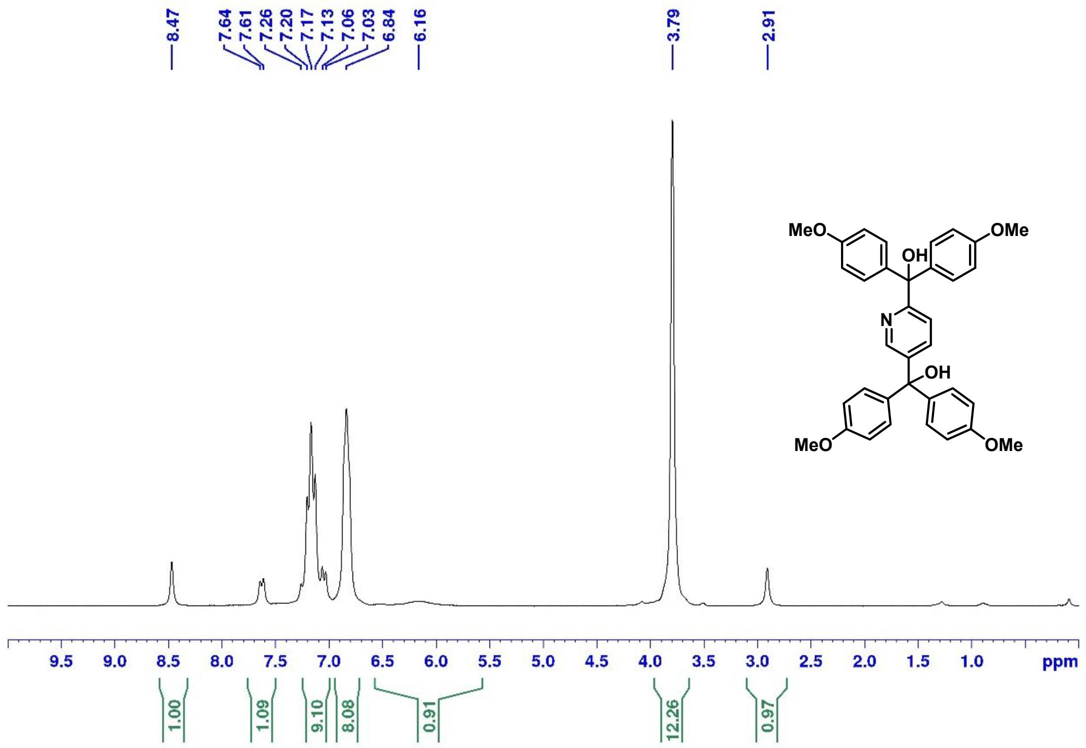

Figure S3. ^{1}H-NMR-Spectrum of **1[Py]** ($CDCl_3$, 250 MHz).

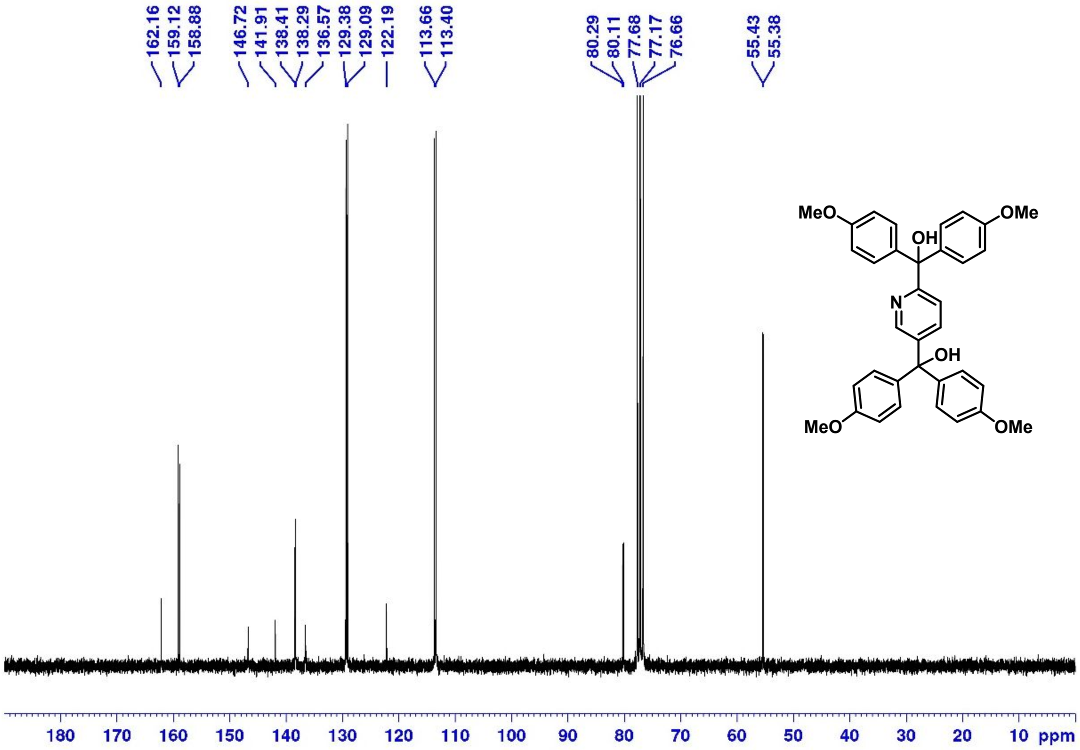

Figure S4. $^{13}C\{^{1}H\}$-NMR-Spectrum of $\mathbf{1^{Py}}$ ($CDCl_3$, 63 MHz).

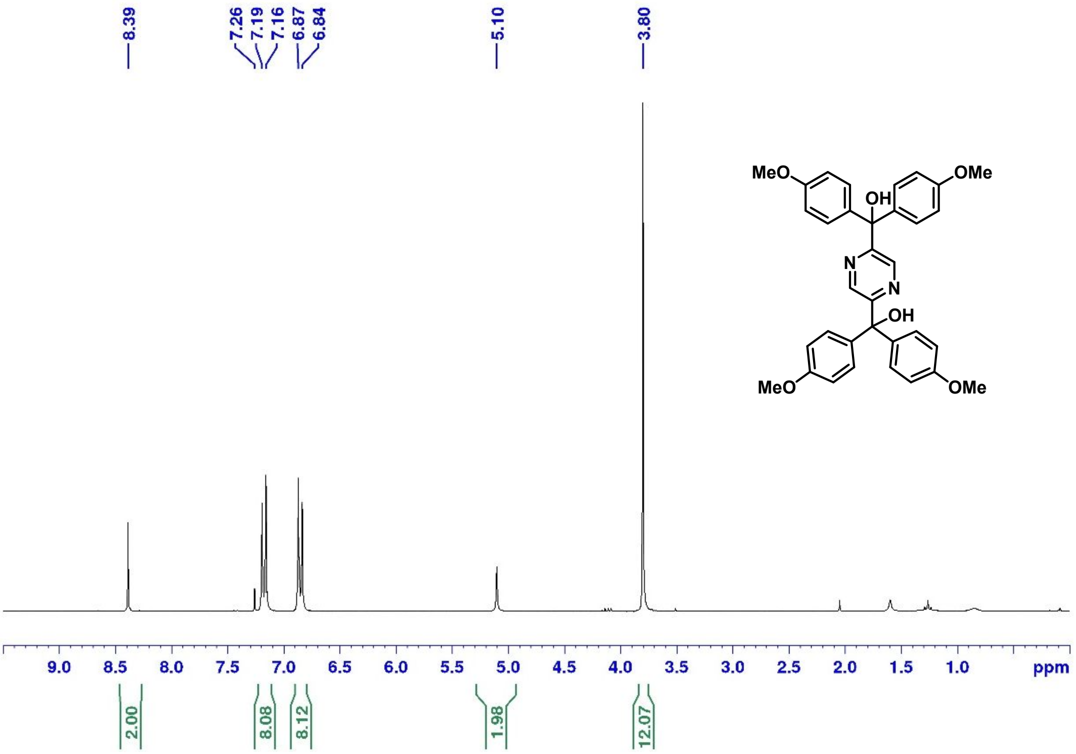

Figure S5. ^{1}H-NMR-Spectrum of $\mathbf{1^{Pz}}$ ($CDCl_3$, 250 MHz).

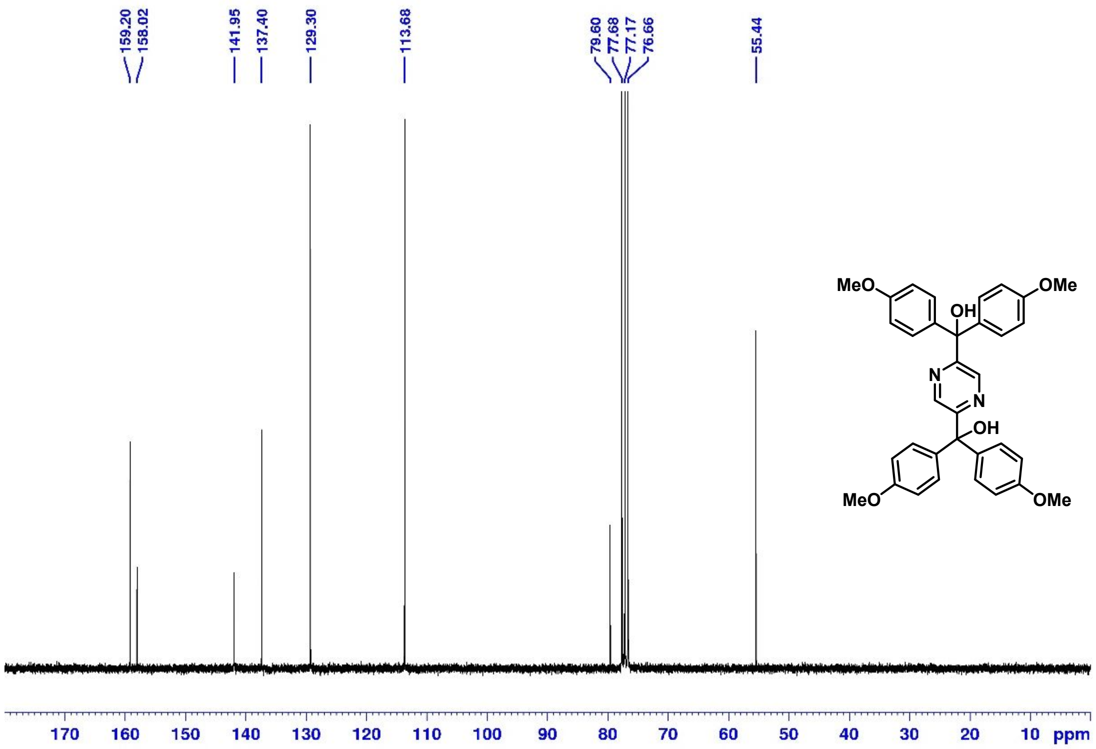

Figure S6. $^{13}C\{^{1}H\}$-NMR-Spectrum of **1^{Pz}** ($CDCl_3$, 63 MHz).

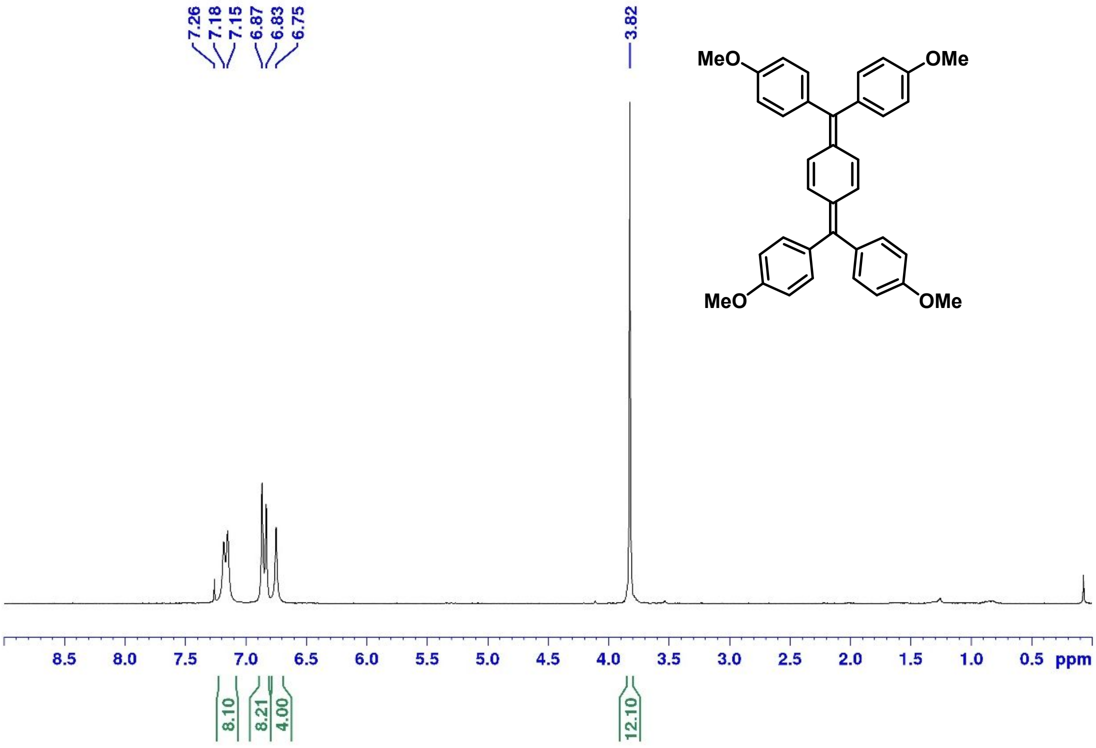

Figure S7. ^{1}H-NMR-Spectrum of **2^{Ph}** ($CDCl_3$, 250 MHz).

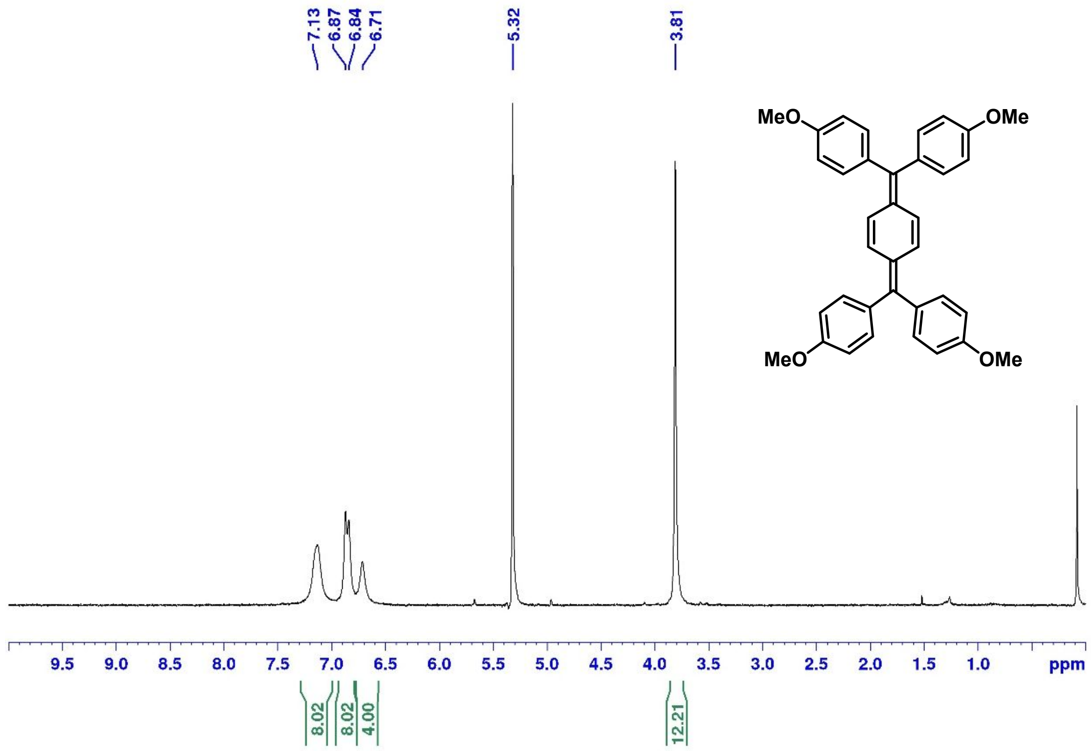

Figure S8. ^{1}H-NMR-Spectrum of **2^{Ph}** (CD_2Cl_2, 250 MHz).

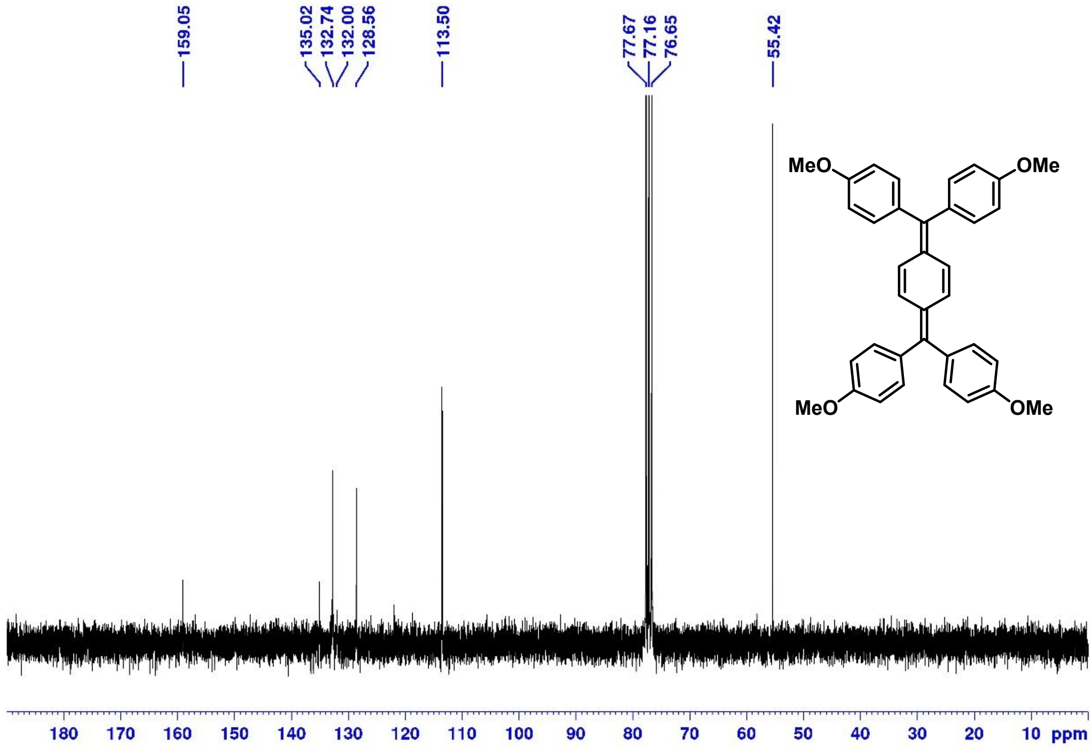

Figure S9. $^{13}C\{^{1}H\}$-NMR-Spectrum **2^{Ph}** ($CDCl_3$, 63 MHz).

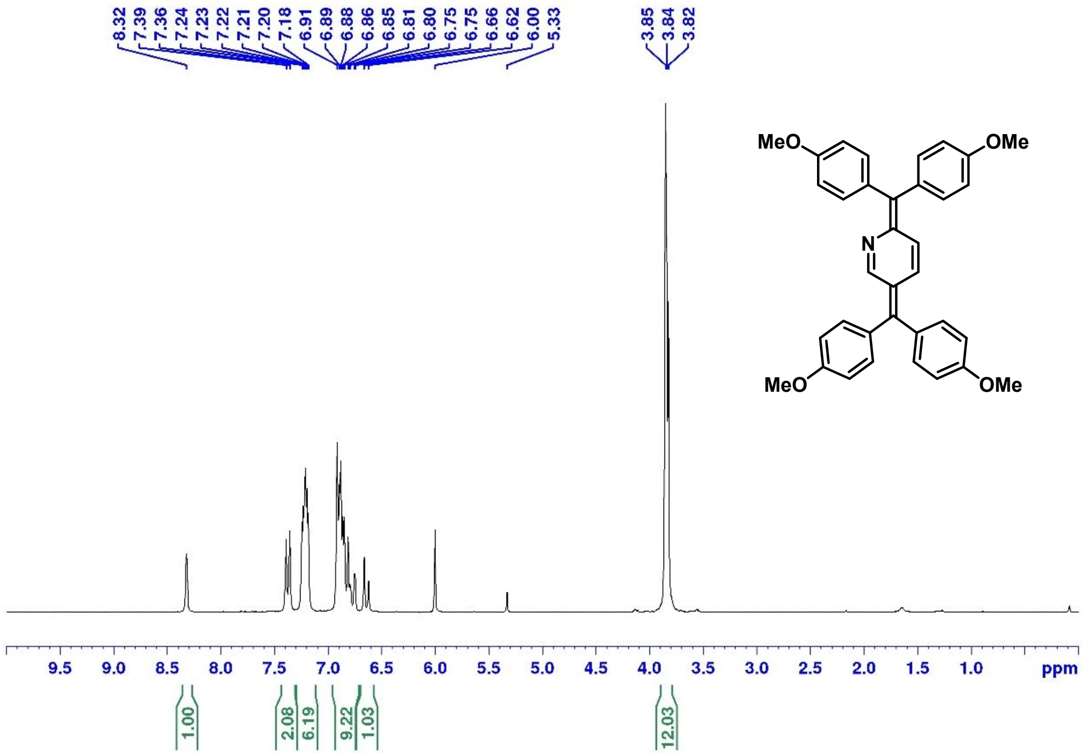

Figure S10. ^{1}H-NMR-Spectrum of **2^{Py}** ($C_2D_2Cl_4$, 250 MHz).

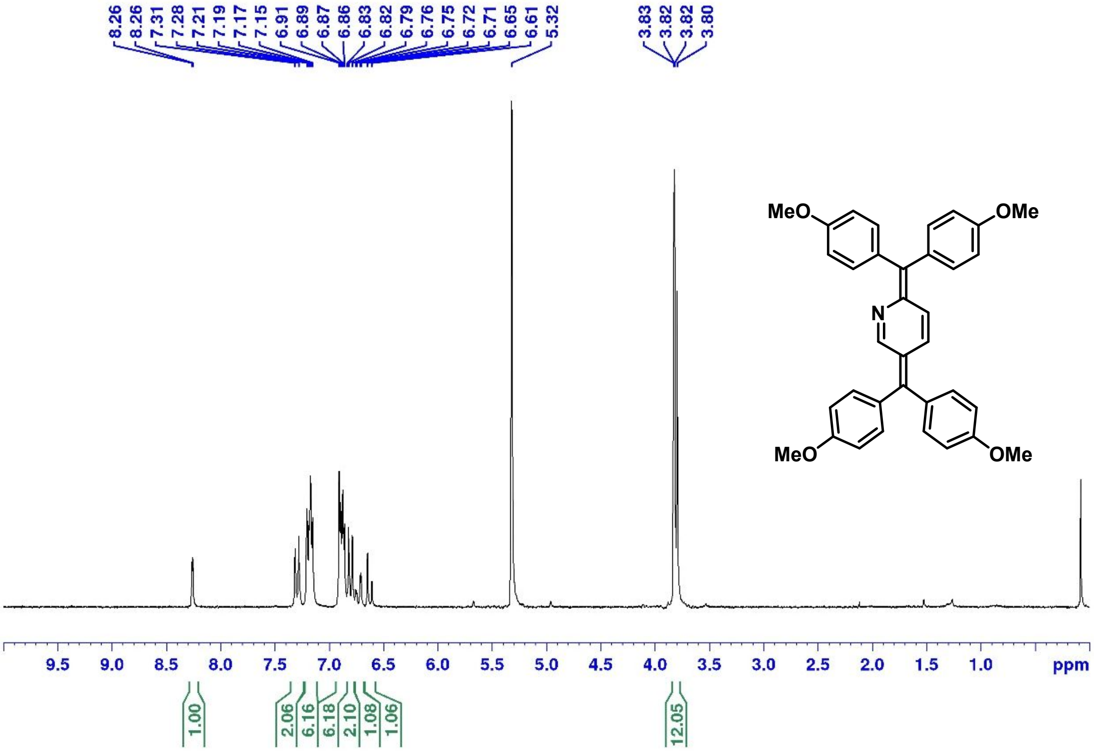

Figure S11. ^{1}H-NMR-Spectrum of **2^{Py}** (CD_2Cl_2, 250 MHz).

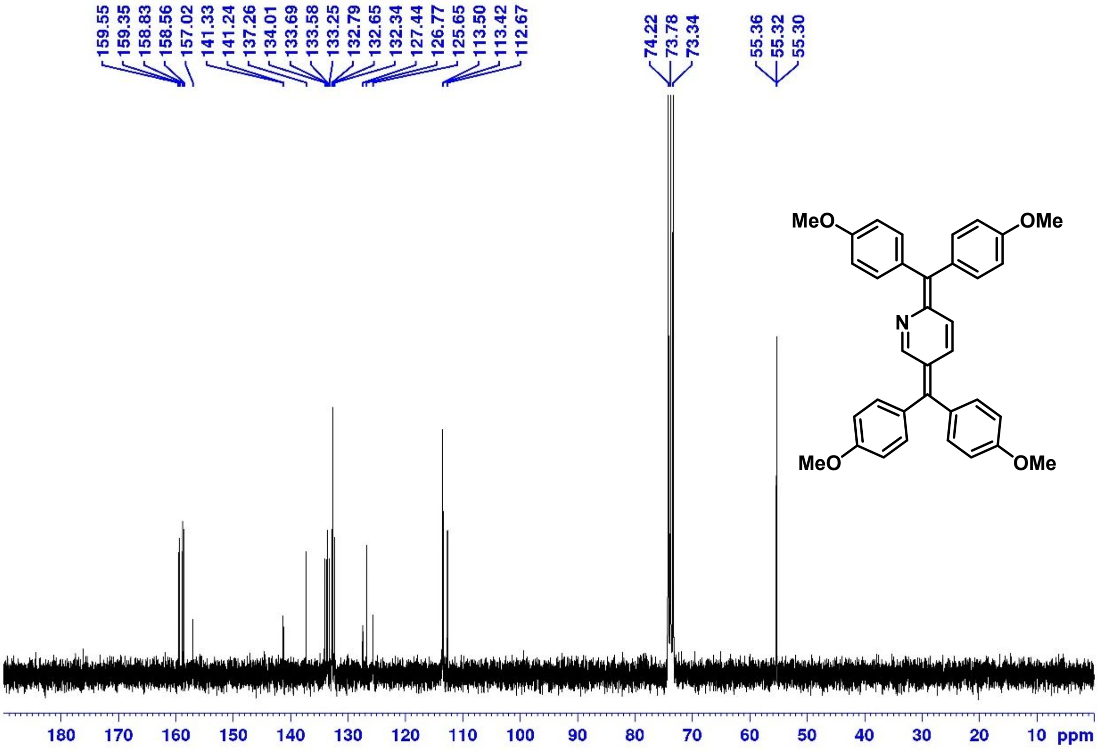

Figure S12. $^{13}C\{^{1}H\}$-NMR-Spectrum **2^{Py}** ($C_2D_2Cl_4$, 63 MHz).

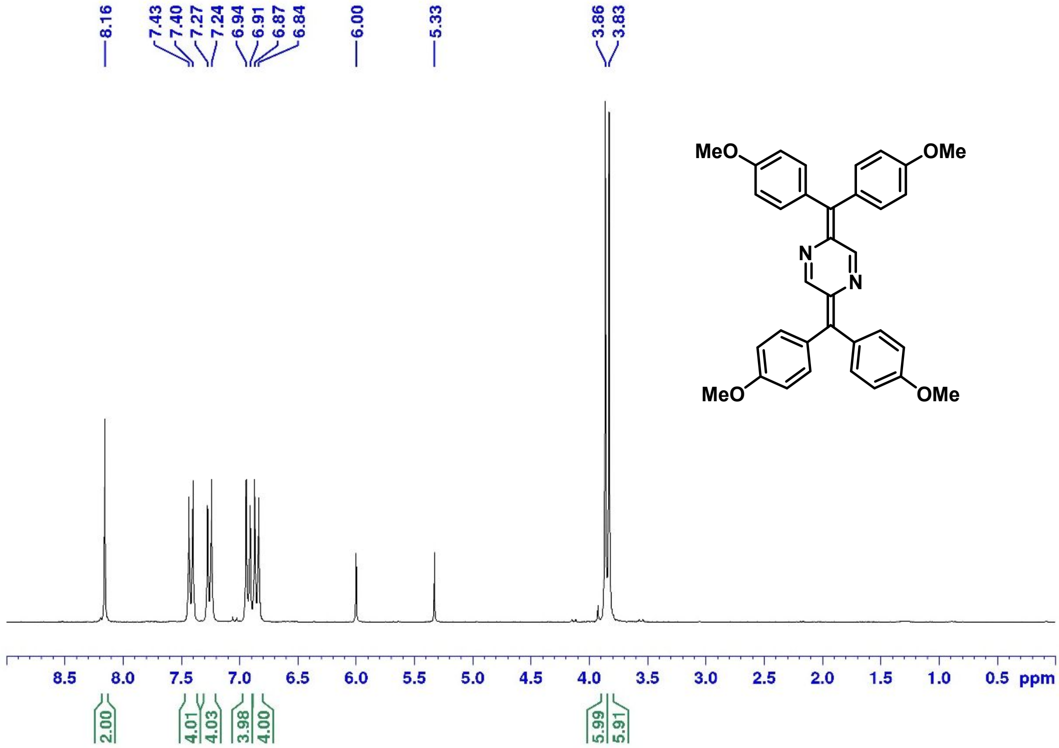

Figure S13. ^{1}H-NMR-Spectrum of **2^{Pz}** ($C_2D_2Cl_4$, 250 MHz).

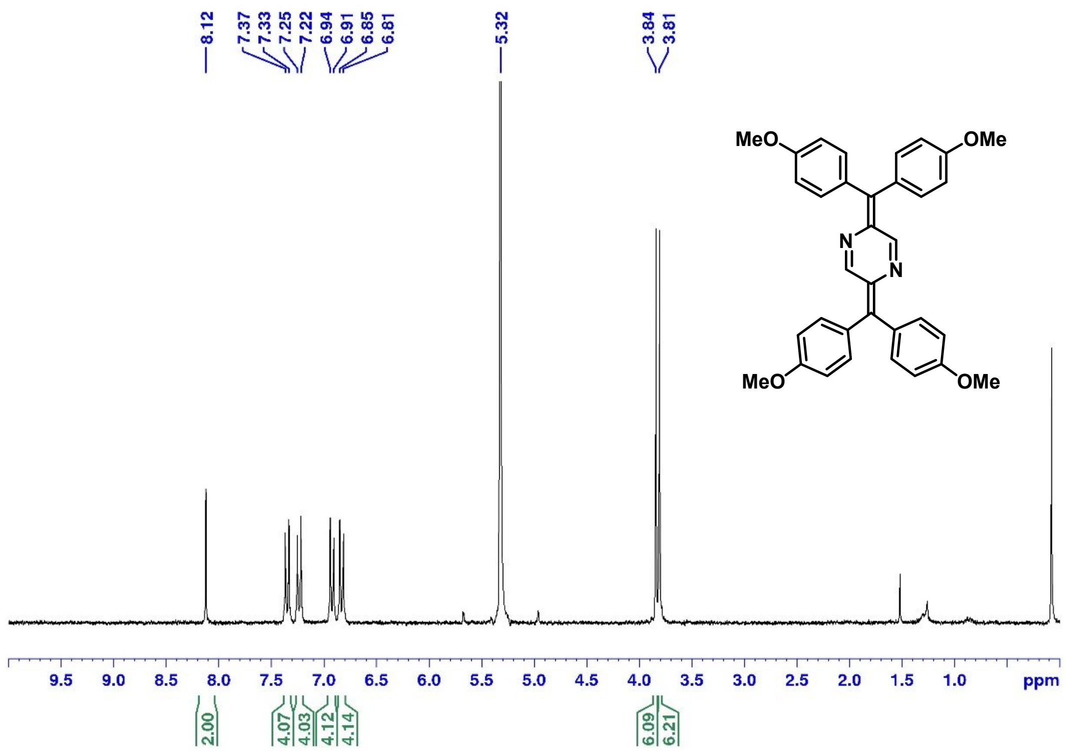

Figure S14. ^{1}H-NMR-Spectrum of **2^{Pz}** (CD_2Cl_2, 250 MHz).

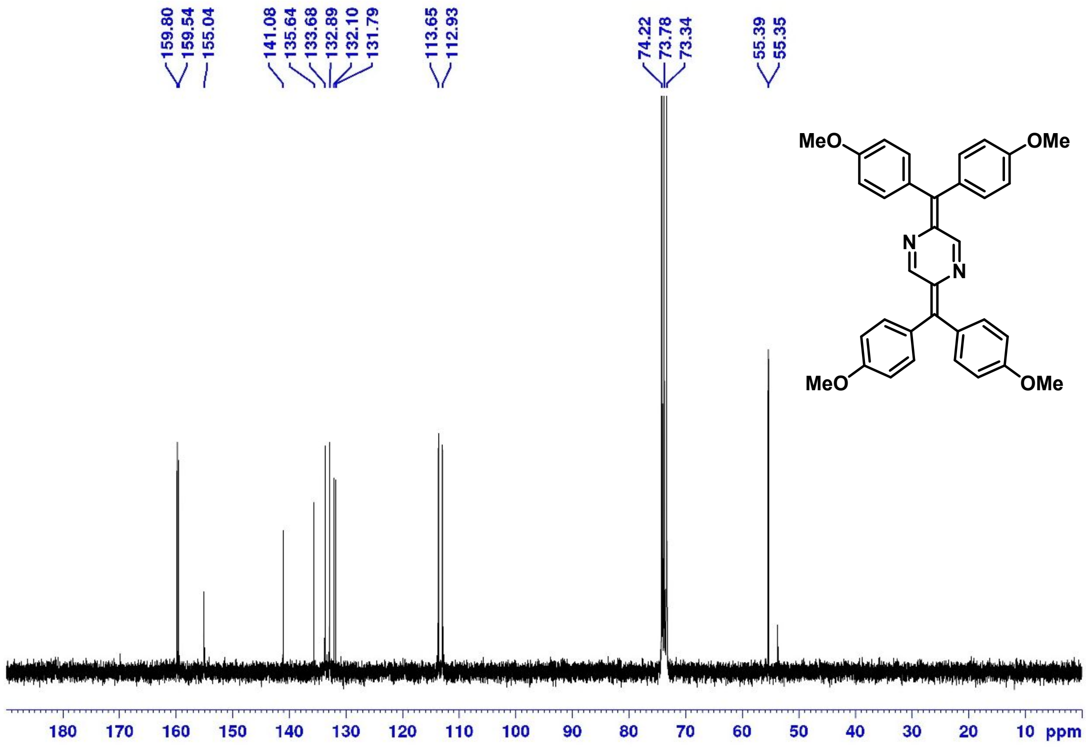

Figure S15. ^{13}C{^{1}H}-NMR-Spectrum **2^{Pz}** ($C_2D_2Cl_4$, 63 MHz).

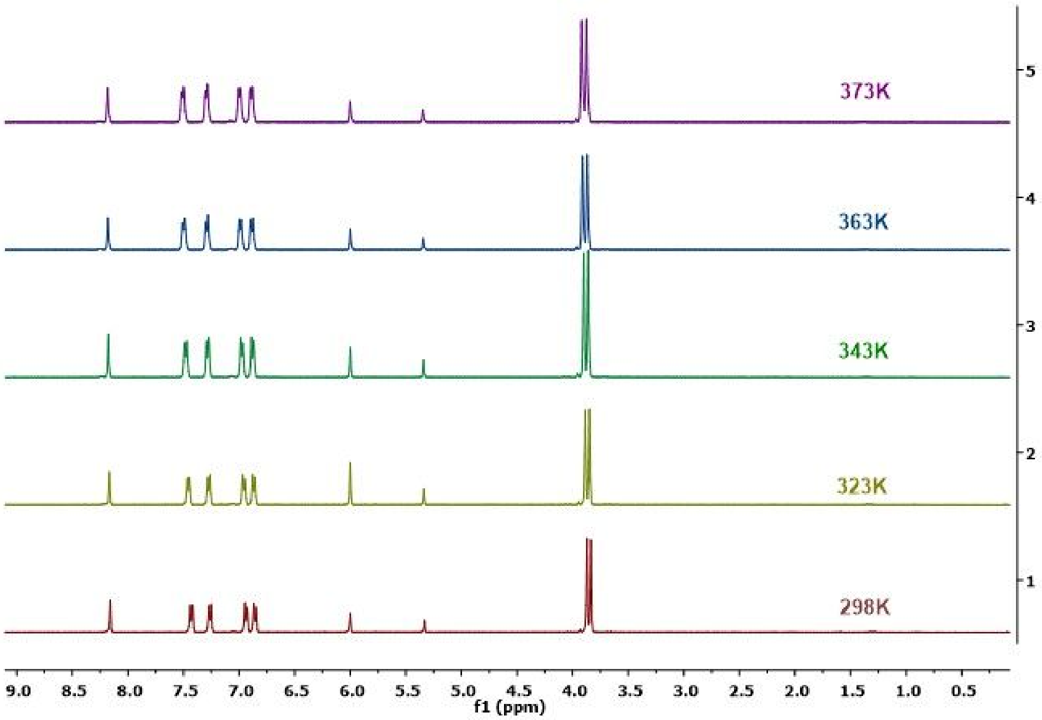

Figure S16. Variation temperature ^{1}H-NMR-Spectrum of **2^Pz** ($C_2D_2Cl_4$, 400 MHz).

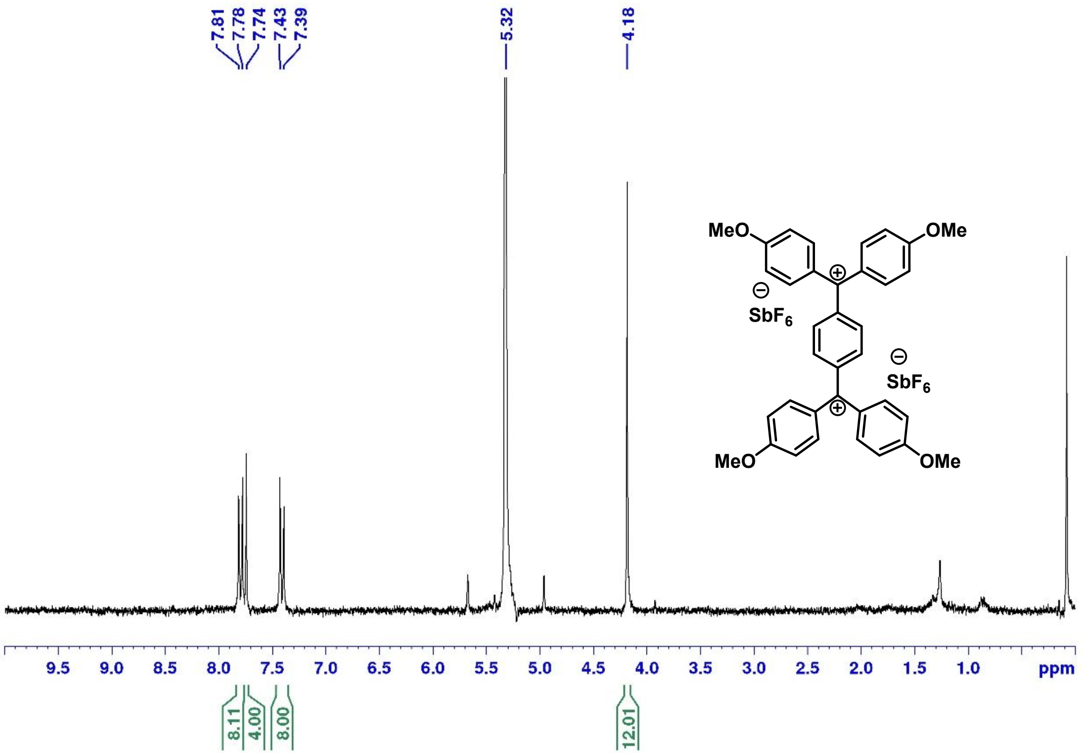

Figure S17. ^{1}H-NMR-Spectrum of **2^PhDC** (CD_2Cl_2, 250 MHz).

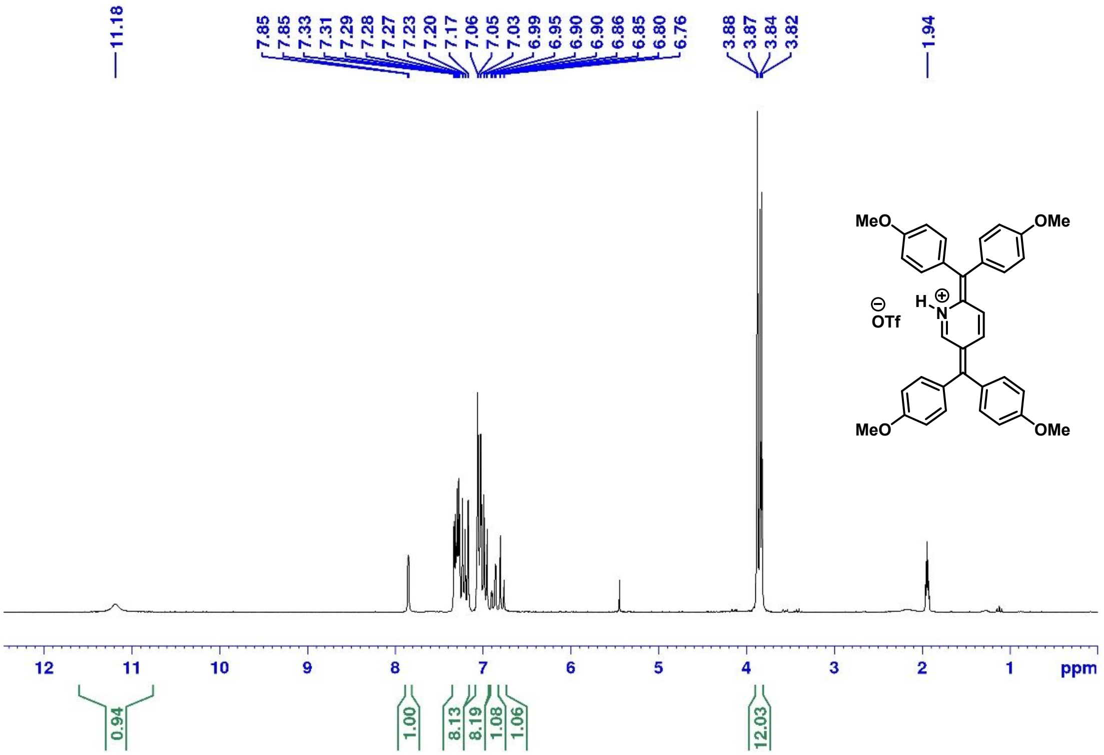

Figure S18. ^{1}H-NMR-Spectrum of **3^{Py}** (CD_3CN, 250 MHz).

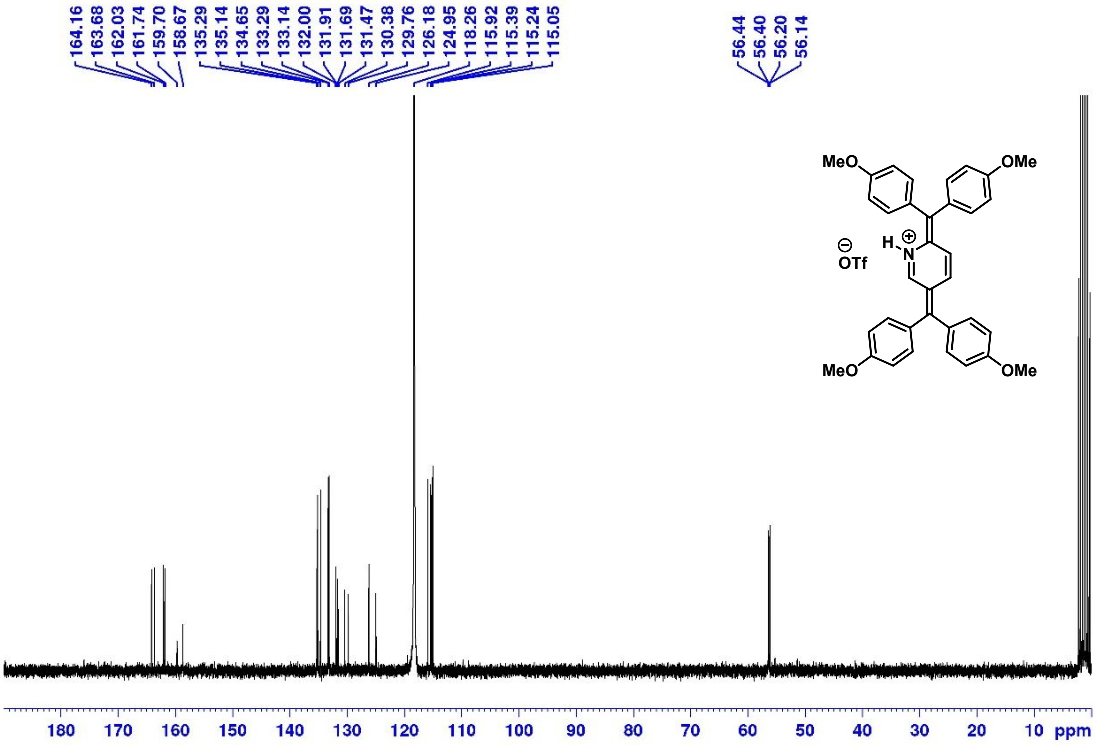

Figure S19. $^{13}C\{^{1}H\}$-NMR-Spectrum **3^{Py}** (CD_3CN, 63 MHz).

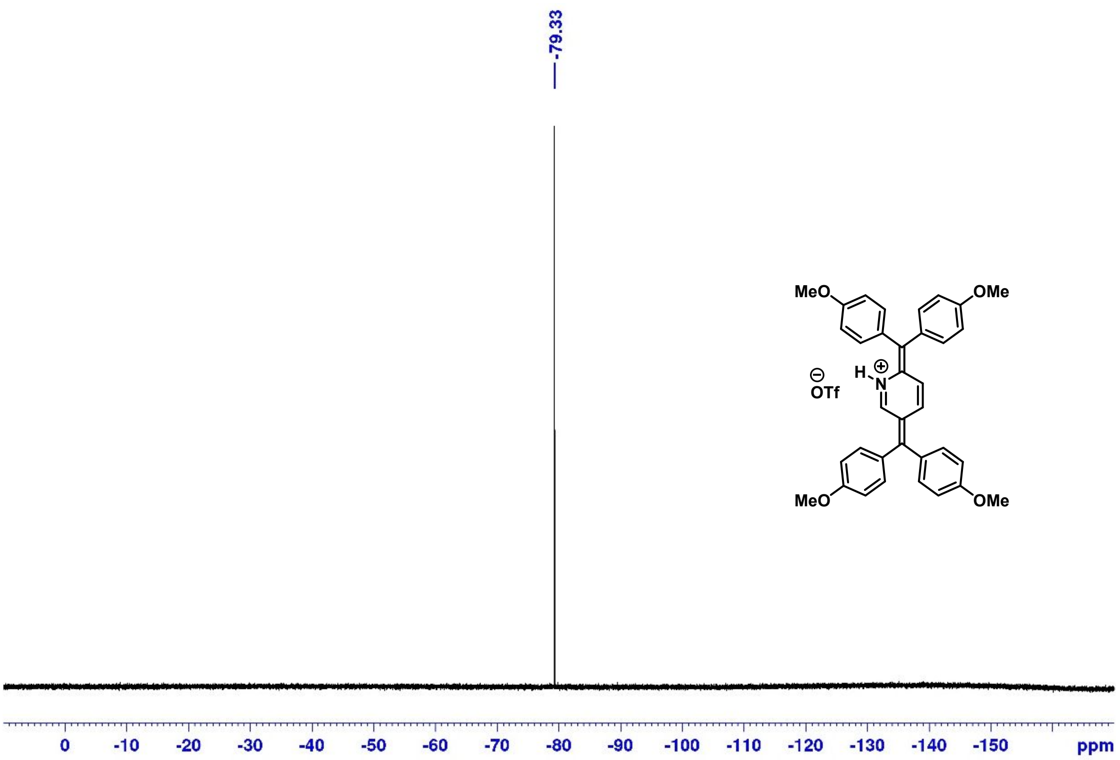

Figure S20. ^{19}F-NMR-Spectrum of $\mathbf{3^{Py}}$ (CD_3CN, 376 MHz).

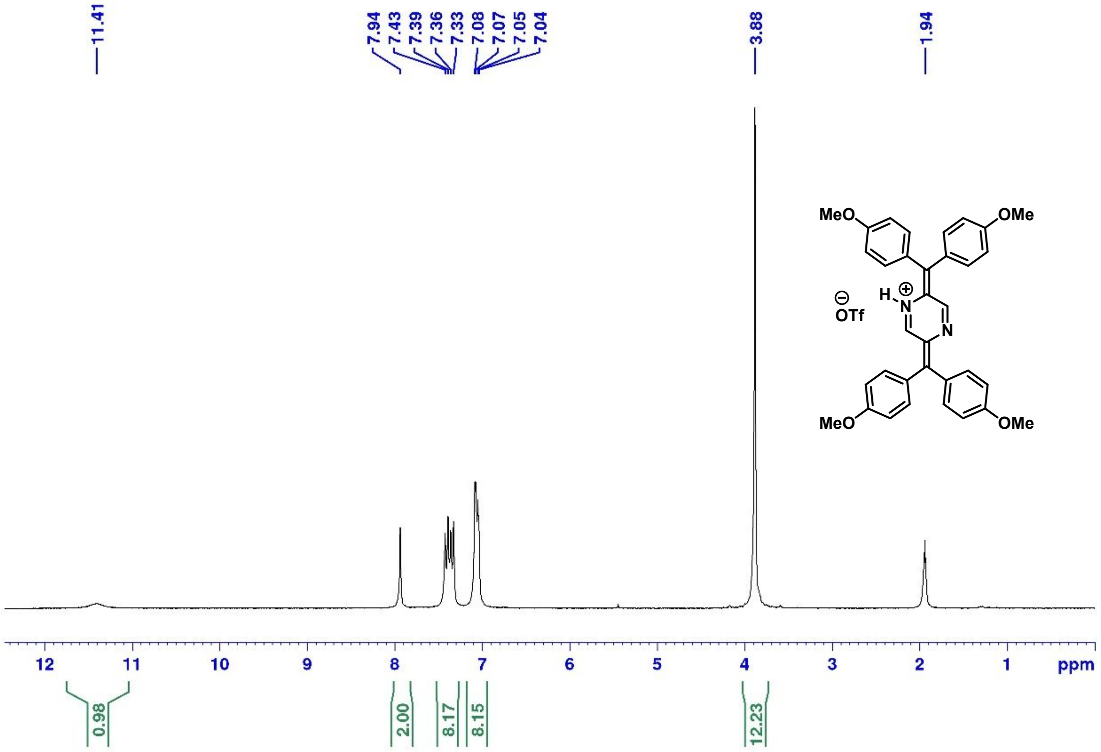

Figure S21. ^{1}H-NMR-Spectrum of $\mathbf{3^{Pz}}$ (CD_3CN, 250 MHz).

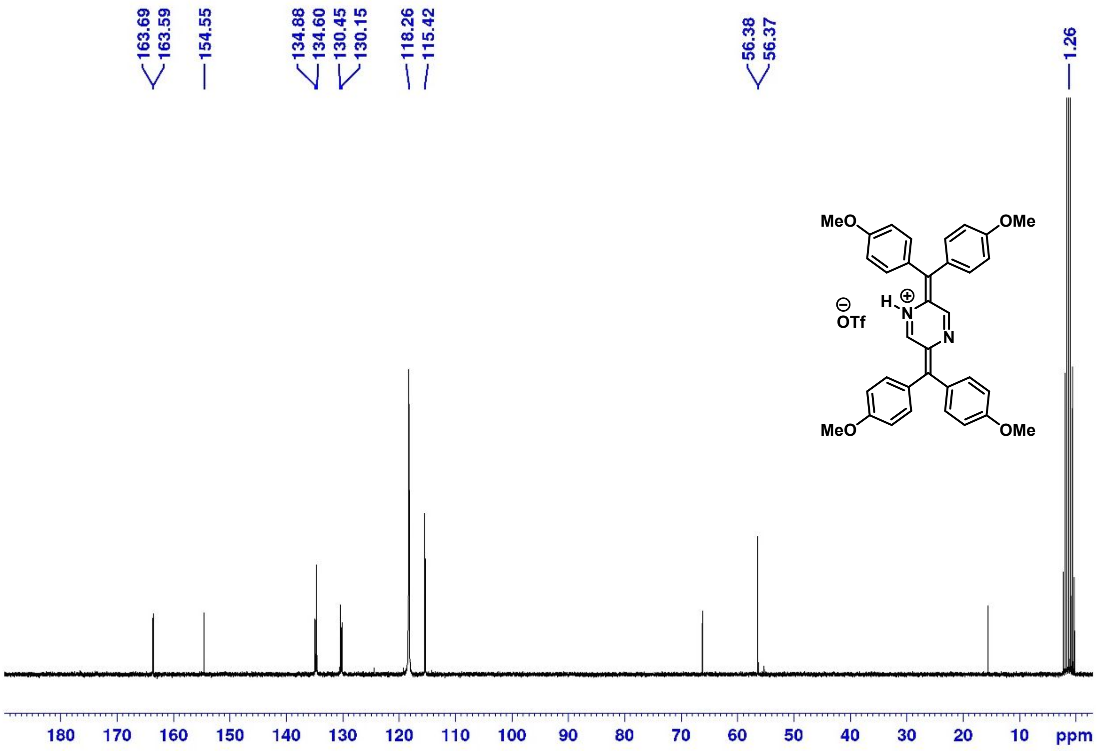

Figure S22. $^{13}C\{^{1}H\}$-NMR-Spectrum **3^{Pz}** (CD_3CN, 63 MHz).

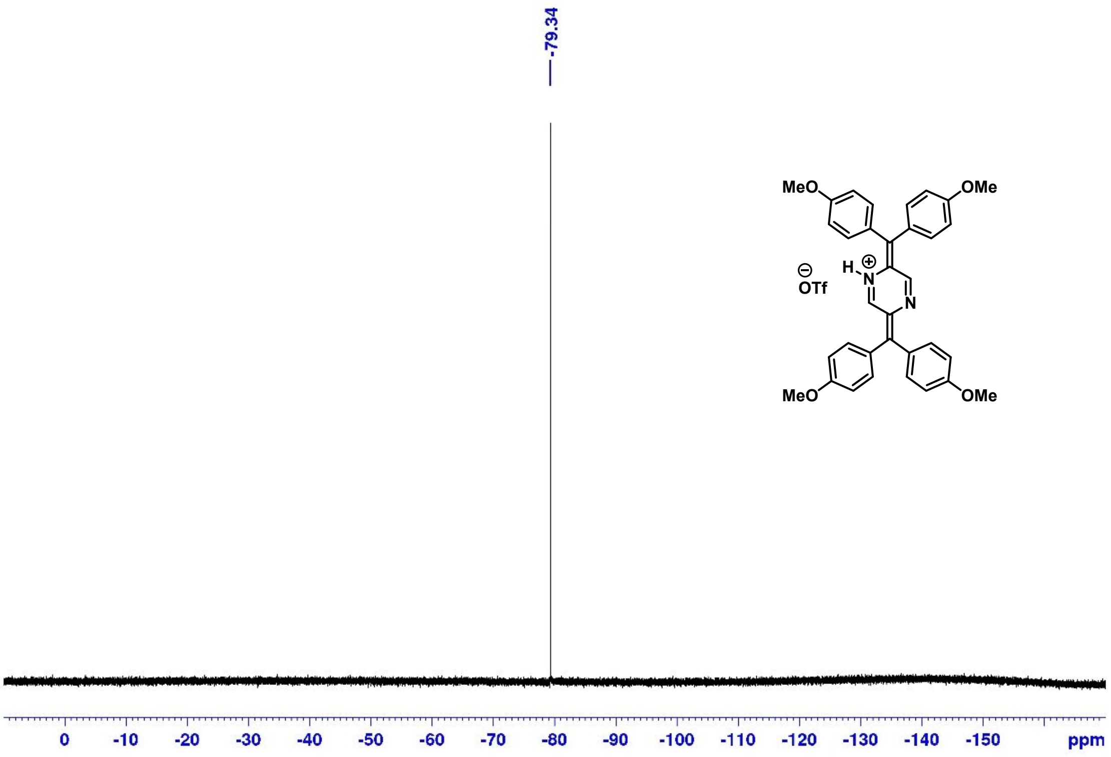

Figure S23. ^{19}F-NMR-Spectrum of **3^{Pz}** (CD_3CN, 376 MHz).

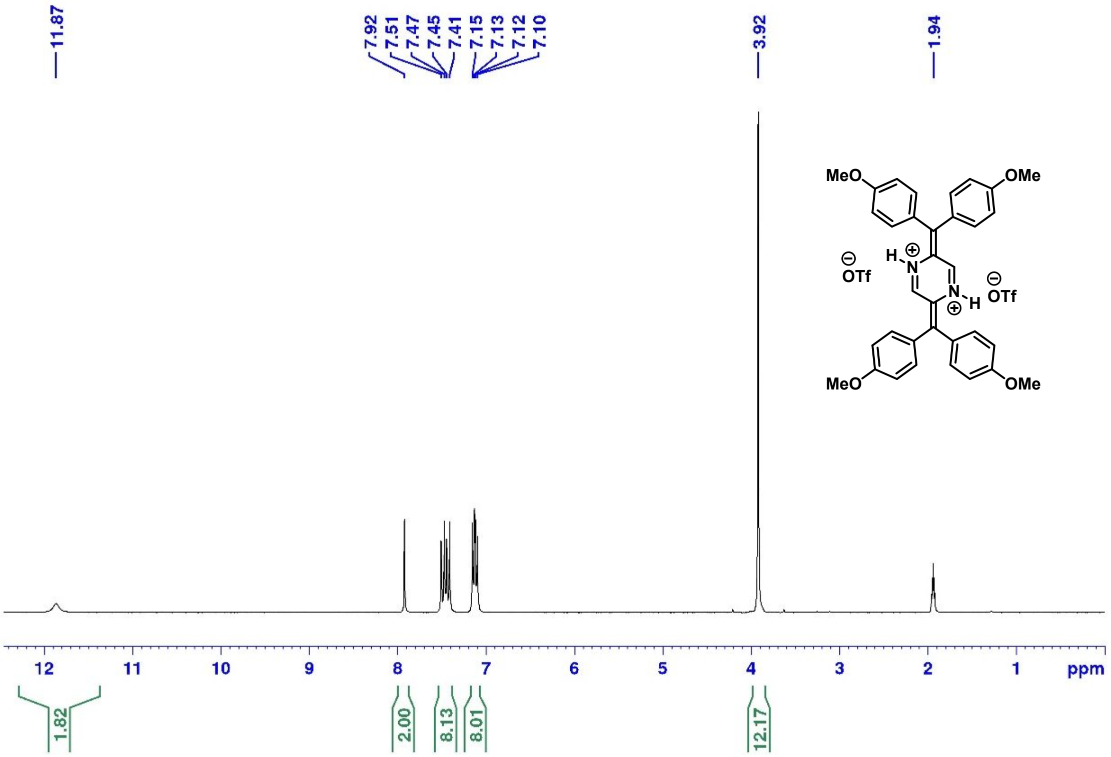

Figure S24. ^{1}H-NMR-Spectrum of **4^{Pz}** (CD_3CN, 250 MHz).

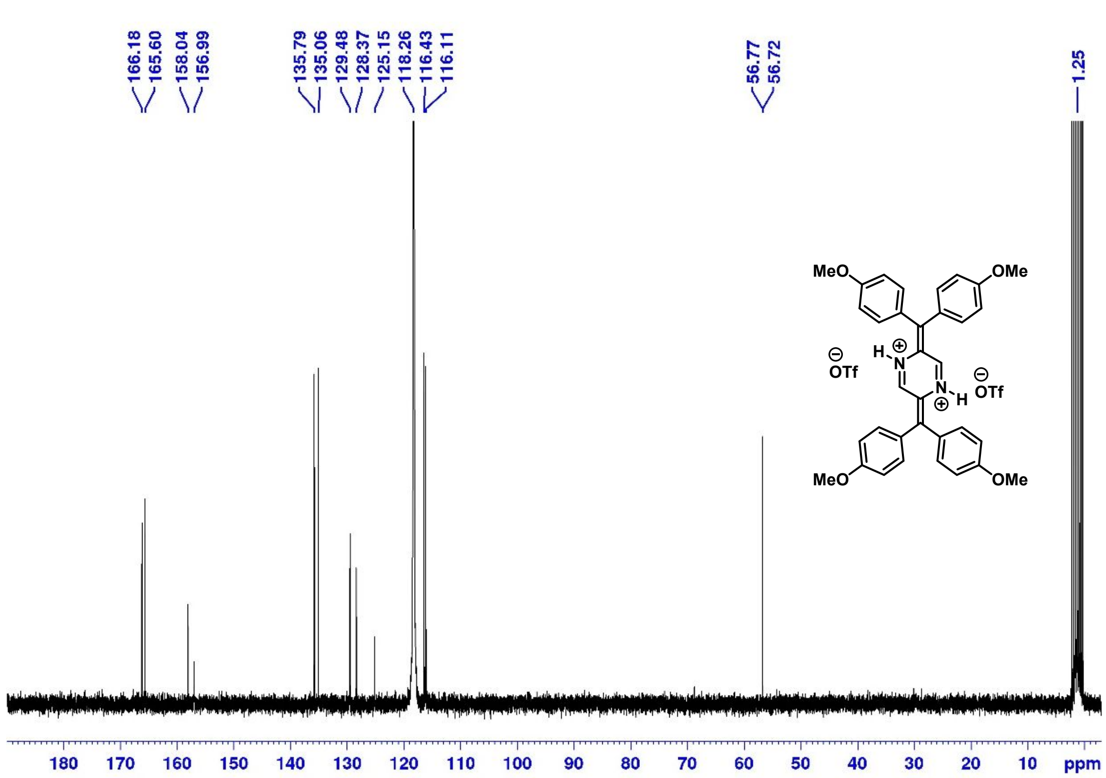

Figure S25. ^{13}C{^{1}H}-NMR-Spectrum **4^{Pz}** (CD_3CN, 63 MHz).

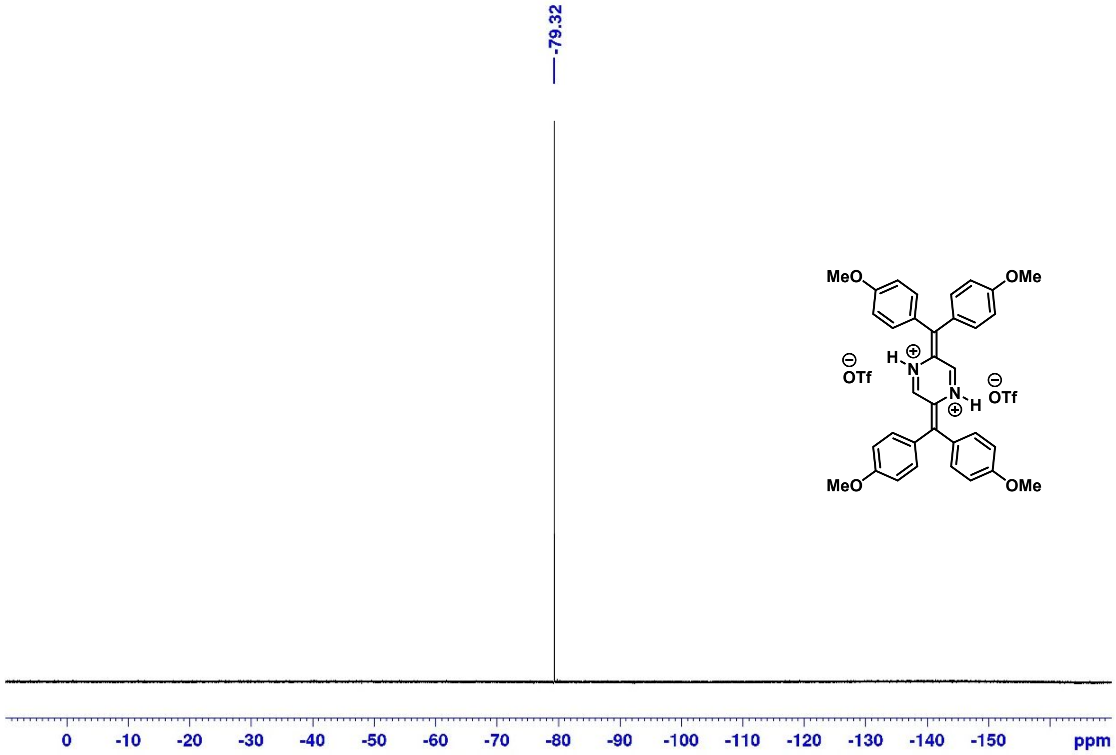

Figure S26. ^{19}F-NMR-Spectrum of **4^{Pz}** (CD_3CN, 376 MHz).

4. NMR Spectroscopy controlled reactions

Stability of **2^{Pz}** under oxygen atmosphere

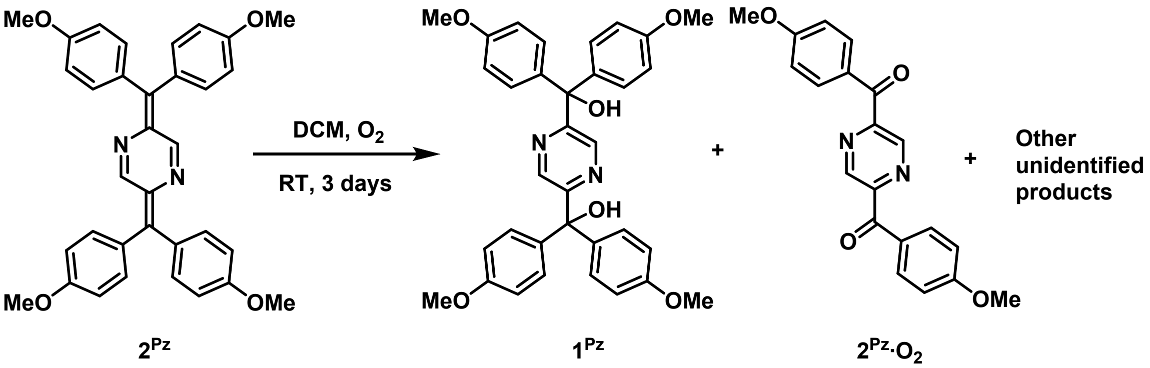

2^{Pz} (10 mg, 0.02 mmol, 1 equiv.) was dissolved in 10 mL regular DCM (non-dry) in a Schlenk flask. Oxygen gas was purge through the mixture for 15 minutes to saturate the solution with O_2. The flask was sealed with a stopcock and the mixture was stirred for 3 days at room temperature under an oxygen atmosphere. A small color change from orange to yellow was observed during the reaction. All volatiles were removed and a ^{1}H-NMR spectrum of the crude products in $CDCl_3$ was recorded. The NMR spectrum suggests the formation of **1^{Pz}**, a keto compound **2^{Pz}·O$_2$** (which was also isolated as single crystal during the crystallization attempt of **2^{Pz}** under ambient conditions) (Figure S39), mostly unreacted starting **2^{Pz}**, and some un-identified side products.

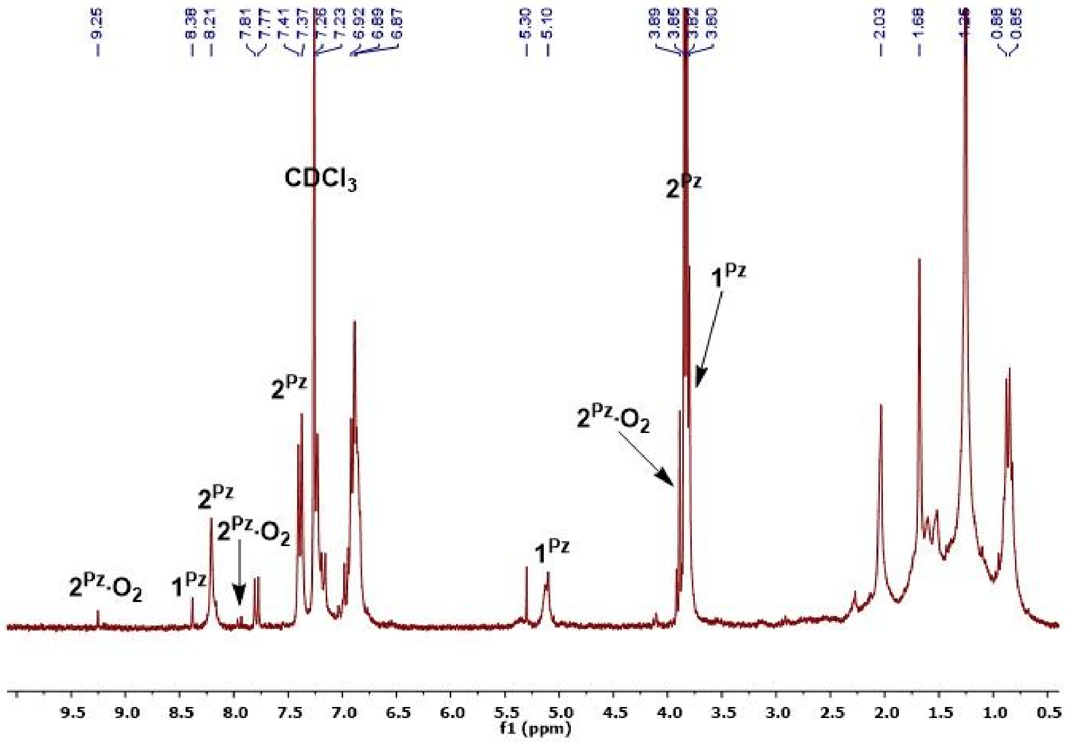

Figure S27. ^{1}H-NMR-Spectrum of crude reaction mixture of **2^{Pz}** upon reaction with O_2 under oxygen atmosphere (CD_2Cl_2, 250 MHz).

<u>Reaction of **2^{Py}** and **2^{Pz}** with Lewis acid Tris(pentafluorophenyl)borane (TPB)</u>

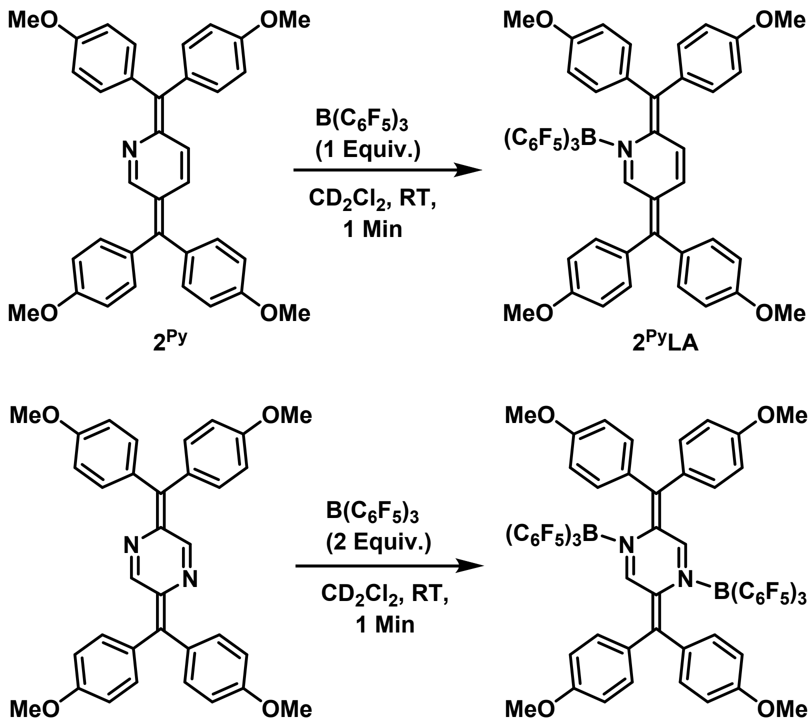

A mixture of **2^{Py}** (5 mg, 0.01 mmol, 1 equiv.) and $B(C_6F_5)_3$ (5 mg, 0.01 mmol, 1 equiv.) or **2^{Pz}** (5 mg, 0.01 mmol, 1 equiv.) and $B(C_6F_5)_3$ (10 mg, 0.02 mmol, 1 equiv.) was placed into a J Young NMR tube and dissolved in dry CD_2Cl_2. The tube was shaken for 1 minute to form the corresponding Lewis acid adducts **2^{Py}LA** and **2^{Pz}LA**. Changes in the ^{1}H-NMR spectrum are comparble to the starting compounds **2^{Py}** and **2^{Pz}**, and ^{11}B- and ^{19}F-NMR spectra are comparable to TPBm

which suggests the formation of the adduct. A red shift in the UV-vis-NIR spectra further confirms the formation of **2^{Py}LA** and **2^{Pz}LA** (Figure S60, S61).

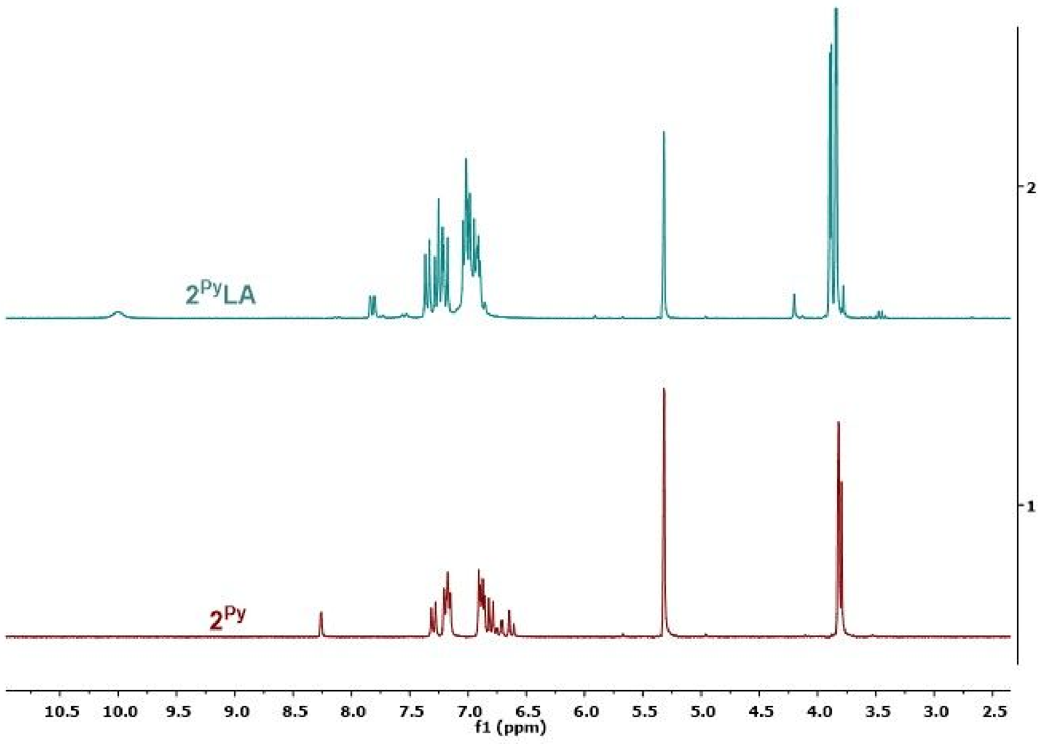

Figure S28. Change in the ^{1}H-NMR-Spectrum of **2^{Py}** upon reaction with TPB to form **2^{Py}LA** (CD_2Cl_2, 250 MHz).

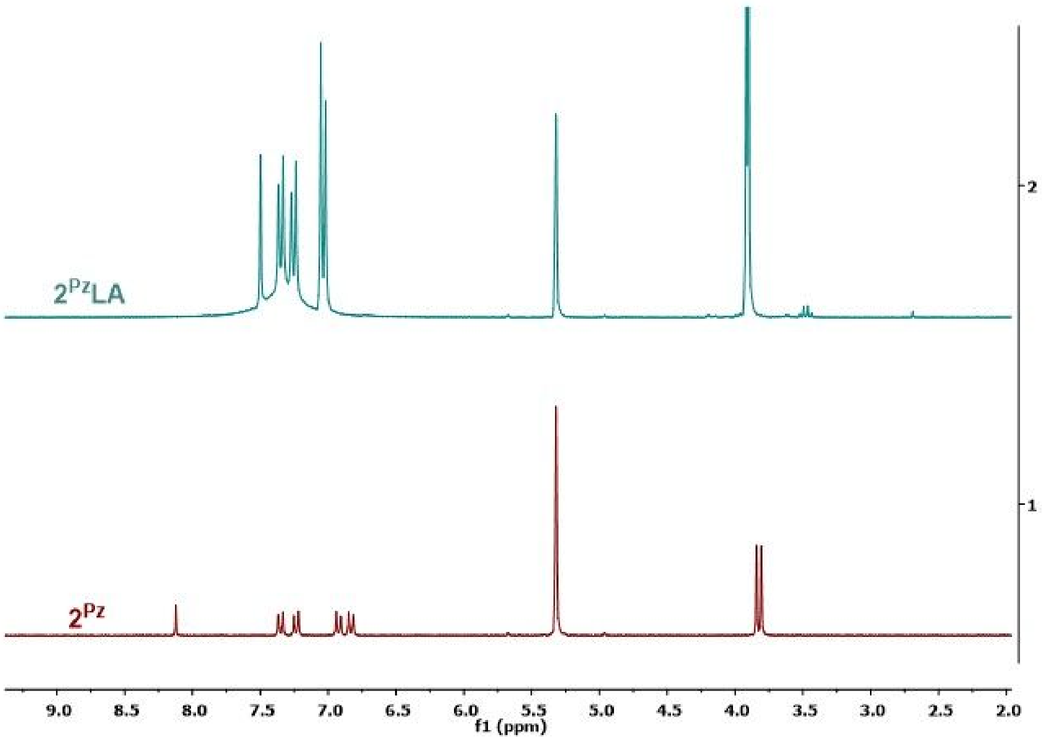

Figure S29. Change in the ^{1}H-NMR-Spectrum of **2^{Py}** upon reaction with TPB to form **2^{Py}LA** (CD_2Cl_2, 250 MHz).

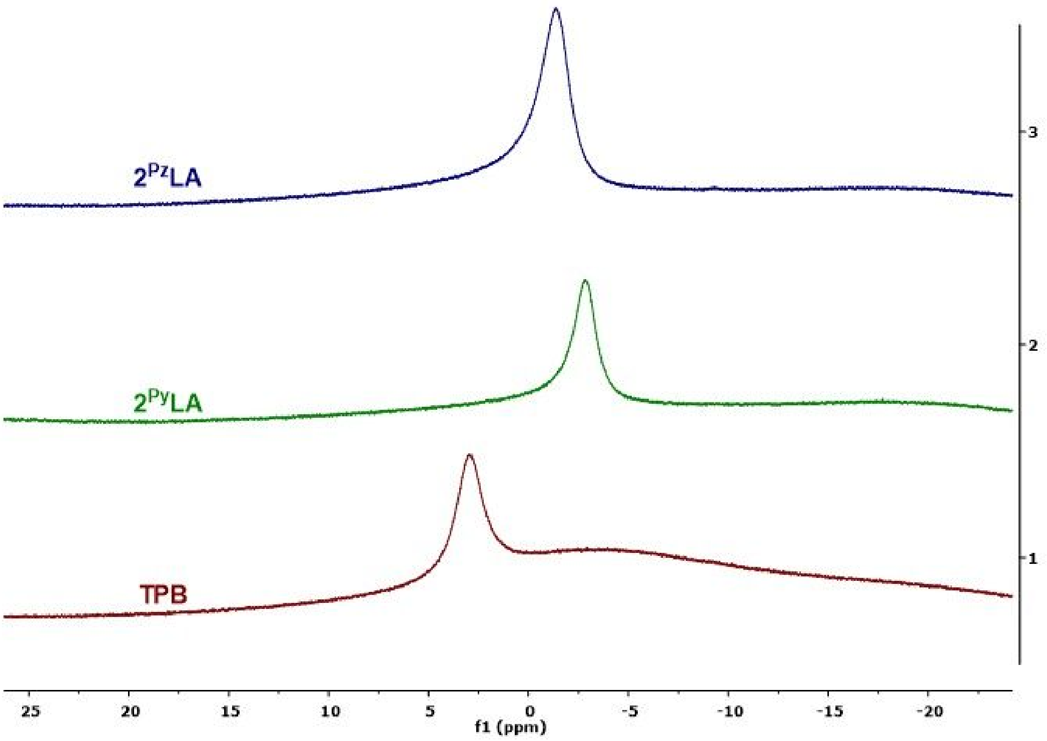

Figure S30. Change in the ^{11}B-NMR-Spectrum of TPB upon reaction with **2^{Py}** and **2^{Pz}** to form **2^{Py}LA** and **2^{Pz}LA** (CD_2Cl_2, 250 MHz).

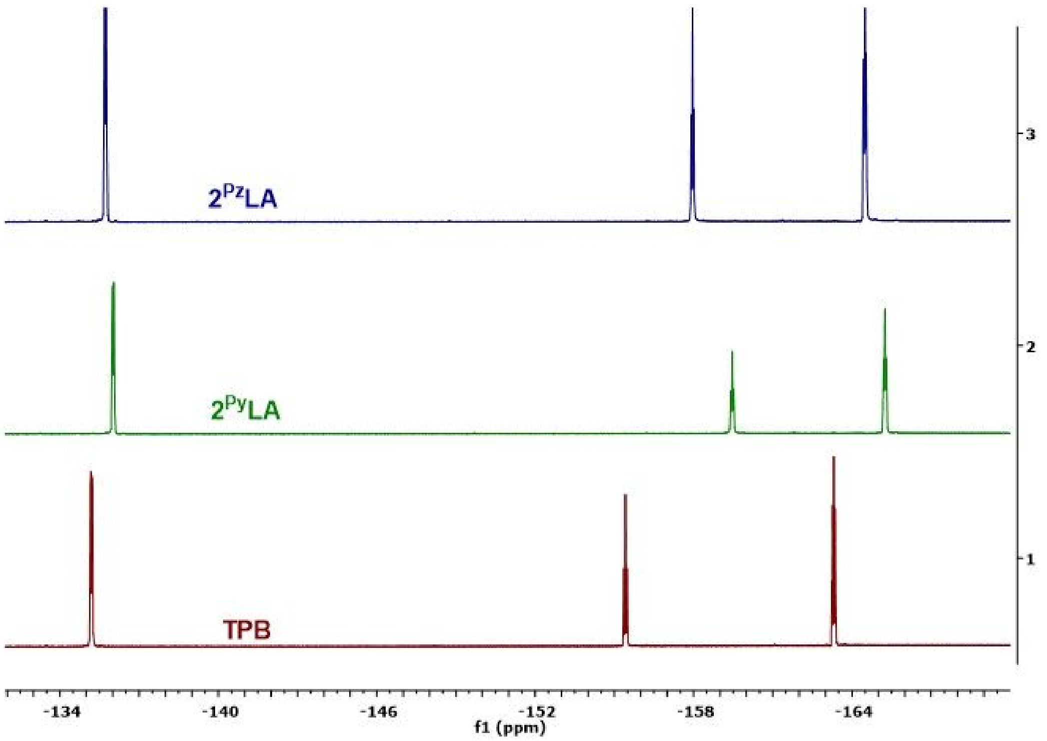

Figure S31. Change in the ^{19}F-NMR-Spectrum of TPB upon reaction with **2^{Py}** and **2^{Pz}** to form **2^{Py}LA** and **2^{Pz}LA** (CD_2Cl_2, 250 MHz).

5. Crystal structures and crystallographic data

Crystal data and structure refinement of **1**^{Pz} (CCDC #2478909)

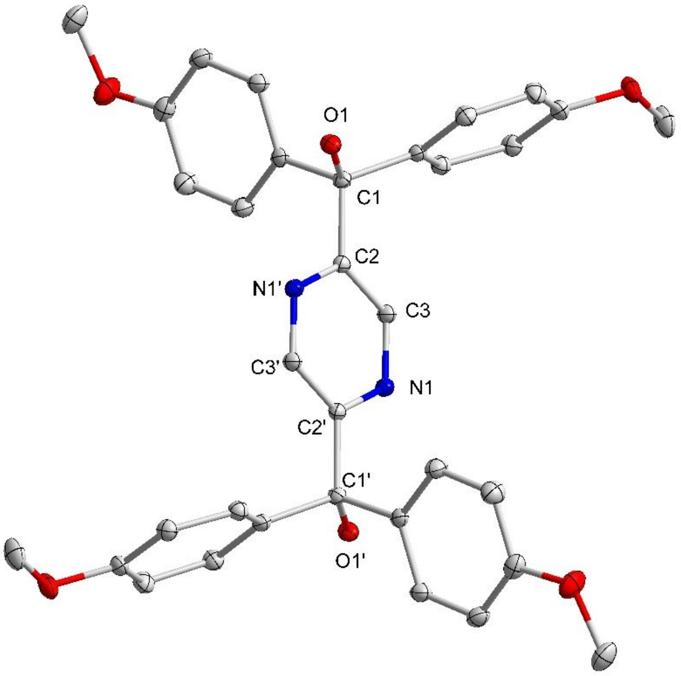

Figure S32. XRay solid-state structure of **1**^{Pz} Ellipsoids are all set to 50% probability.

Table S1. Crystal data and structure refinement for **1**^{Pz}.

Identification code	**1**^{Pz}
Empirical formula	$C_{34}H_{32}N_2O_6$
Formula weight	564.61
Temperature/K	99.97
Crystal system	triclinic
Space group	P-1
a/Å	6.4329(5)
b/Å	9.2899(7)
c/Å	12.5444(10)
α/°	110.031(4)
β/°	93.333(4)
γ/°	90.533(4)
Volume/Å^3	702.78(10)
Z	1
ρ_{calc}g/cm^3	1.334
μ/mm^{-1}	0.092
F(000)	298.0
Crystal size/mm^3	0.321 × 0.318 × 0.245
Radiation	MoKα (λ = 0.71073)
2Θ range for data collection/°	3.464 to 56.612
Index ranges	-8 ≤ h ≤ 8, -12 ≤ k ≤ 12, -16 ≤ l ≤ 15
Reflections collected	10067
Independent reflections	3464 [R_{int} = 0.0224, R_{sigma} = 0.0225]
Data/restraints/parameters	3464/0/193
Goodness-of-fit on F^2	1.057
Final R indexes [I>=2σ (I)]	R_1 = 0.0406, wR_2 = 0.1065

Final R indexes [all data]	R_1 = 0.0461, wR_2 = 0.1117
Largest diff. peak/hole / e Å^{-3}	0.36/-0.32

Crystal data and structure refinement of **2^{Ph}** (CCDC #2478914)

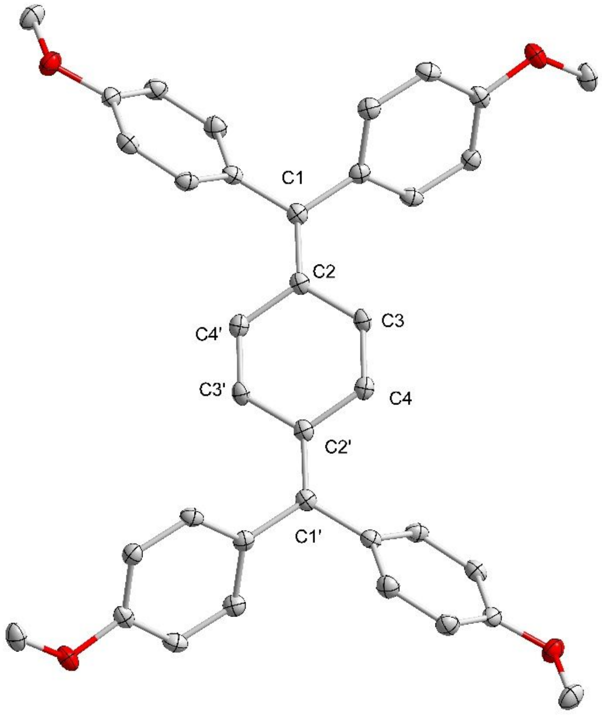

Figure S33. X-Ray solid-state structure of **2^{Ph}**. Ellipsoids are all set to 50% probability.

Table S2. Crystal data and structure refinement for **2^{Ph}**.

Identification code	**2^{Ph}**
Empirical formula	$C_{36}H_{32}O_4$
Formula weight	528.61
Temperature/K	100.01
Crystal system	monoclinic
Space group	$P2_1/c$
a/Å	14.2157(14)
b/Å	6.6403(6)
c/Å	14.3032(15)
α/°	90
β/°	93.186(6)
γ/°	90
Volume/Å^3	1348.1(2)
Z	2
ρ_{calc}g/cm^3	1.302
μ/mm^{-1}	0.084
F(000)	560.0
Crystal size/mm^3	0.256 × 0.189 × 0.062
Radiation	MoKα (λ = 0.71073)
2Θ range for data collection/°	5.706 to 53.016
Index ranges	-17 ≤ h ≤ 17, -8 ≤ k ≤ 8, -17 ≤ l ≤ 17
Reflections collected	16763
Independent reflections	2752 [R_{int} = 0.0885, R_{sigma} = 0.0679]
Data/restraints/parameters	2752/0/183
Goodness-of-fit on F^2	0.997
Final R indexes [I>=2σ (I)]	R_1 = 0.0432, wR_2 = 0.0860
Final R indexes [all data]	R_1 = 0.0834, wR_2 = 0.0964

Largest diff. peak/hole / e Å^{-3}	0.19/-0.24

Crystal data and structure refinement of **2^{Py}** (CCDC # 2478946)

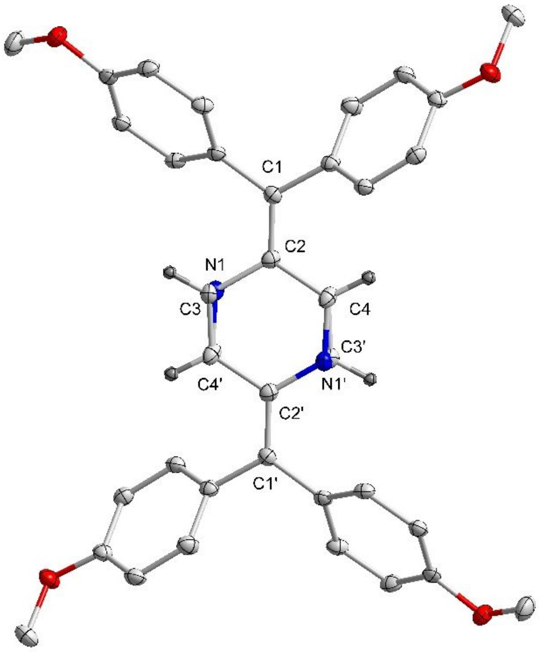

Figure S34. X-Ray solid-state structure of **2^{Py}**. Ellipsoids are all set to 50% probability. All H atoms are omitted for clarity reasons. Selected bond lengths [Å]: C1–C2 1.381(2); C2–C4 1.446(2); C2–C3 1.49(2); C3–C4 1.31(2); C4–N1 1.355 (19); C2–N1 1.365(18).

Table S3. Crystal data and structure refinement for **2^{Py}.**

Identification code	**2^{Py}**
Empirical formula	$C_{35}H_{30}NO_4$
Formula weight	528.60
Temperature/K	99.99
Crystal system	monoclinic
Space group	$P2_1/c$
a/Å	14.2145(10)
b/Å	6.5541(4)
c/Å	14.3024(10)
α/°	90
β/°	93.203(4)
γ/°	90
Volume/Å^3	1330.38(16)
Z	2
ρ_{calc}g/cm^3	1.320
μ/mm^{-1}	0.086
F(000)	558.0
Crystal size/mm^3	0.301 × 0.242 × 0.089
Radiation	MoKα (λ = 0.71073)
2Θ range for data collection/°	2.87 to 55.006
Index ranges	-18 ≤ h ≤ 14, -8 ≤ k ≤ 8, -18 ≤ l ≤ 18
Reflections collected	19434
Independent reflections	3064 [R_{int} = 0.0522, R_{sigma} = 0.0425]
Data/restraints/parameters	3064/12/186
Goodness-of-fit on F^2	1.032

Final R indexes [I>=2σ (I)]	R_1 = 0.0470, wR_2 = 0.0984
Final R indexes [all data]	R_1 = 0.0748, wR_2 = 0.1092
Largest diff. peak/hole / e Å^{-3}	0.38/-0.23

Crystal data and structure refinement of **2^{Pz}** (CCDC # 2478915)

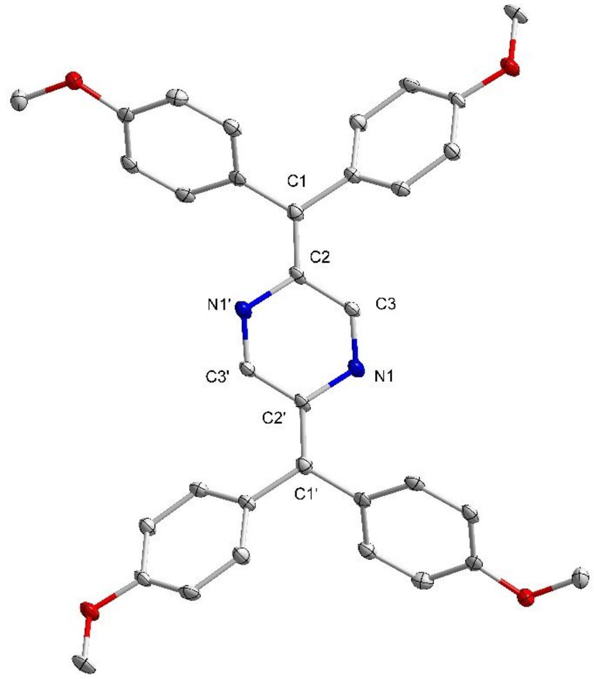

Figure S35. X-Ray solid-state structure of **2^{Pz}**. Ellipsoids are all set to 50% probability.

Table S4. Crystal data and structure refinement for **2^{Pz}**.

Identification code	**2^{Pz}**
Empirical formula	$C_{34}H_{30}N_2O_4$
Formula weight	530.60
Temperature/K	99.99
Crystal system	monoclinic
Space group	$P2_1/c$
a/Å	14.222(6)
b/Å	6.508(3)
c/Å	14.247(6)
α/°	90
β/°	93.208(13)
γ/°	90
Volume/Å^3	1316.6(10)
Z	2
ρ_{calc}g/cm^3	1.338
μ/mm^{-1}	0.088
F(000)	560.0
Crystal size/mm^3	0.19 × 0.11 × 0.08
Radiation	MoKα (λ = 0.71073)
2Θ range for data collection/°	5.728 to 53.042
Index ranges	-17 ≤ h ≤ 17, -8 ≤ k ≤ 8, -17 ≤ l ≤ 17
Reflections collected	9558
Independent reflections	2717 [R_{int} = 0.0853, R_{sigma} = 0.1013]
Data/restraints/parameters	2717/0/183
Goodness-of-fit on F^2	1.020

Final R indexes [I>=2σ (I)]	R_1 = 0.0720, wR_2 = 0.1824
Final R indexes [all data]	R_1 = 0.1219, wR_2 = 0.2146
Largest diff. peak/hole / e Å^{-3}	0.47/-0.39

Crystal data and structure refinement of **2^{Ph}DC** (CCDC # 2478932)

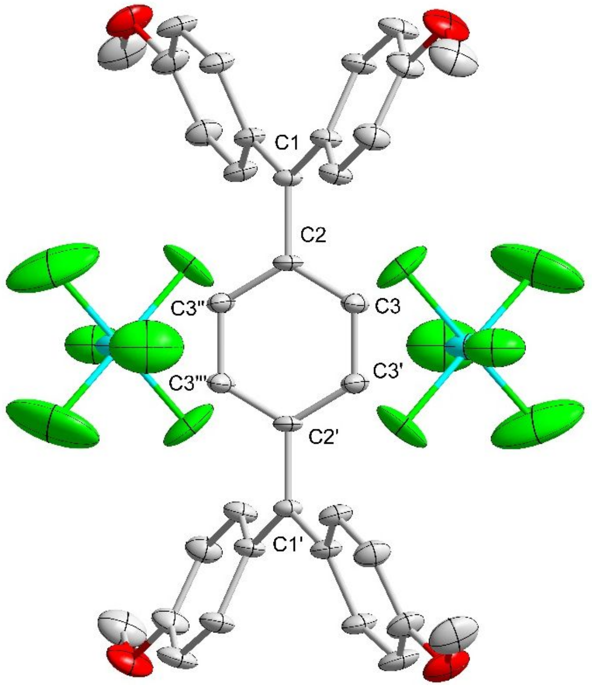

Figure S36. X-Ray solid-state structure of **2^{Ph}DC**. Ellipsoids are all set to 50% probability.

Table S5. Crystal data and structure refinement for **2^{Ph}DC**.

Identification code	**2^{Ph}DC**
Empirical formula	$C_{36}H_{32}F_{12}O_4Sb_2$
Formula weight	1000.11
Temperature/K	159.99
Crystal system	monoclinic
Space group	C2/m
a/Å	15.7575(13)
b/Å	16.5781(13)
c/Å	9.3501(7)
α/°	90
β/°	123.704(4)
γ/°	90
Volume/Å^3	2032.0(3)
Z	2
ρ_{calc}g/cm^3	1.635
μ/mm^{-1}	1.420
F(000)	980.0
Crystal size/mm^3	0.45 × 0.32 × 0.25
Radiation	MoKα (λ = 0.71073)
2Θ range for data collection/°	3.962 to 56.666
Index ranges	-20 ≤ h ≤ 17, -22 ≤ k ≤ 22, -7 ≤ l ≤ 12
Reflections collected	9570
Independent reflections	2620 [R_{int} = 0.0349, R_{sigma} = 0.0311]
Data/restraints/parameters	2620/6/135
Goodness-of-fit on F^2	1.114

Final R indexes [I>=2σ (I)]	R_1 = 0.0589, wR_2 = 0.1831
Final R indexes [all data]	R_1 = 0.0699, wR_2 = 0.1945
Largest diff. peak/hole / e Å^{-3}	1.64/-1.11

Crystal data and structure refinement of **3^{Py}** (CCDC # 2478931)

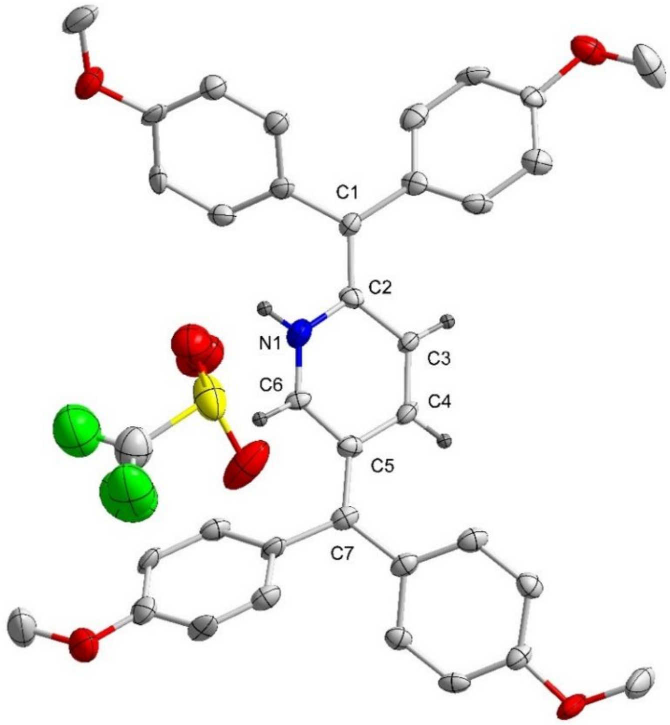

Figure S37. X-Ray solid-state structure of **3^{Py}**. Ellipsoids are all set to 50% probability. All H atoms are omitted for clarity reasons. Selected bond lengths [Å]: C1–C2 1.388(10); C2–C3 1.457(10); C3–C4 1.355(9); C2–N1 1.390(9).

Table S6. Crystal data and structure refinement for **3^{Py}**.

Identification code	**3^{Py}**
Empirical formula	$C_{36}H_{32}F_3NO_7S$
Formula weight	679.68
Temperature/K	159.99
Crystal system	monoclinic
Space group	C2/c
a/Å	29.278(7)
b/Å	7.9928(19)
c/Å	28.894(6)
α/°	90
β/°	111.147(6)
γ/°	90
Volume/Å^3	6306(2)
Z	8
ρ_{calc}g/cm^3	1.432
μ/mm^{-1}	0.173
F(000)	2832.0
Crystal size/mm^3	0.31 × 0.06 × 0.03
Radiation	MoKα (λ = 0.71073)
2Θ range for data collection/°	2.982 to 51.01
Index ranges	-34 ≤ h ≤ 35, -9 ≤ k ≤ 9, -34 ≤ l ≤ 21
Reflections collected	20568
Independent reflections	5771 [R_{int} = 0.1265, R_{sigma} = 0.1656]
Data/restraints/parameters	5771/55/464

Goodness-of-fit on F^2	1.043
Final R indexes [I>=2σ (I)]	R_1 = 0.1183, wR_2 = 0.2888
Final R indexes [all data]	R_1 = 0.2320, wR_2 = 0.3499
Largest diff. peak/hole / e Å^{-3}	0.53/-0.44

Crystal data and structure refinement of **4^Pz** (CCDC # 2478919)

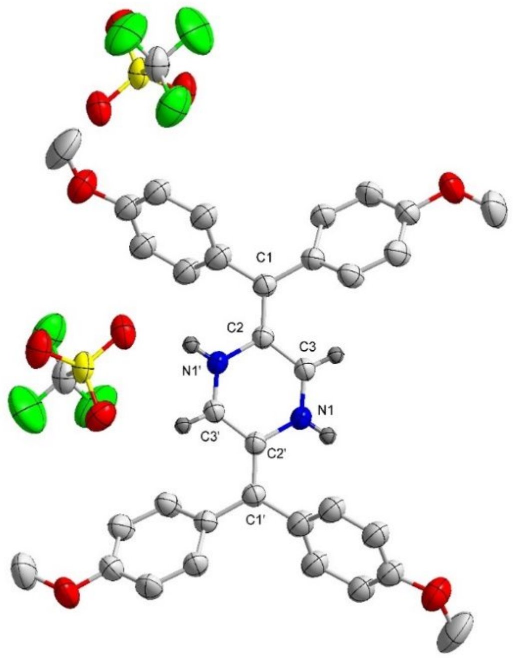

Figure S38. X-Ray solid-state structure of **4^{Pz}**. Ellipsoids are all set to 50% probability. All H atoms are omitted for clarity reasons. Selected bond lengths [Å]: C1–C2 1.381(3); C2–C3 1.411(4); C2–N1 1.411(4); C3–N1 1.296(4).

Table S7. Crystal data and structure refinement for **4^{Pz}**.

Identification code	**4^{Pz}**
Empirical formula	$C_{36}H_{32}F_{5.96}N_2O_{10}S_2$
Formula weight	829.99
Temperature/K	296.15
Crystal system	triclinic
Space group	P-1
a/Å	6.63810(10)
b/Å	11.8946(2)
c/Å	13.1683(3)
α/°	70.7960(10)
β/°	84.4100(10)
γ/°	75.9180(10)
Volume/Å^3	952.20(3)
Z	1
ρ_{calc}g/cm^3	1.447
μ/mm^{-1}	2.053
F(000)	428.0
Crystal size/mm^3	0.665 × 0.341 × 0.329
Radiation	CuKα (λ = 1.54178)
2Θ range for data collection/°	7.11 to 125.962
Index ranges	-7 ≤ h ≤ 7, -12 ≤ k ≤ 13, -15 ≤ l ≤ 15
Reflections collected	7304
Independent reflections	3009 [R_{int} = 0.0264, R_{sigma} = 0.0391]
Data/restraints/parameters	3009/0/272
Goodness-of-fit on F^2	1.101
Final R indexes [I>=2σ (I)]	R_1 = 0.0699, wR_2 = 0.1825

Final R indexes [all data]	R_1 = 0.0738, wR_2 = 0.1856
Largest diff. peak/hole / e Å^{-3}	0.89/-0.45

Crystal data and structure refinement of $\mathbf{2^{Pz}{\cdot}O_2}$ (CCDC # 2478933)

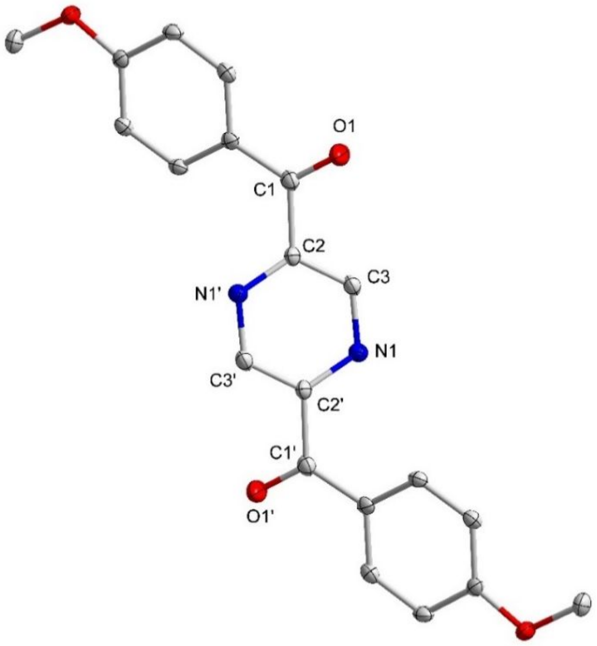

Figure S39. X-Ray solid-state structure of $\mathbf{2^{Pz}{\cdot}O_2}$. Ellipsoids are all set to 50% probability. All H atoms are omitted for clarity reasons. Selected bond lengths [Å]: C1–C2 1.510(3); C2–C3 1.390(3); C2–N1 1.338(2); C3–N1 1.336(2); C1–O1 1.224(2).

Table S8. Crystal data and structure refinement for $\mathbf{2^{Pz}{\cdot}O_2}$.

Identification code	$\mathbf{2^{Pz}{\cdot}O_2}$
Empirical formula	$C_{40}H_{32}N_4O_8$
Formula weight	696.69
Temperature/K	110.0
Crystal system	monoclinic
Space group	$P2_1/n$
a/Å	3.8544(6)
b/Å	10.7128(16)
c/Å	19.014(2)
α/°	90
β/°	94.105(7)
γ/°	90
Volume/Å^3	783.1(2)
Z	1
ρ_{calc}g/cm^3	1.477
μ/mm^{-1}	0.105
F(000)	364.0
Crystal size/mm^3	0.48 × 0.16 × 0.11
Radiation	MoKα (λ = 0.71073)
2Θ range for data collection/°	4.296 to 53.036
Index ranges	$-4 \le h \le 4$, $-13 \le k \le 12$, $-22 \le l \le 23$
Reflections collected	5646
Independent reflections	1613 [R_{int} = 0.0428, R_{sigma} = 0.0601]
Data/restraints/parameters	1613/0/119
Goodness-of-fit on F^2	1.002
Final R indexes [I>=2σ (I)]	R_1 = 0.0458, wR_2 = 0.0949

Final R indexes [all data]	R_1 = 0.0838, wR_2 = 0.1103
Largest diff. peak/hole / e Å^{-3}	0.24/-0.23

6. UV-Vis-NIR Spectroscopy

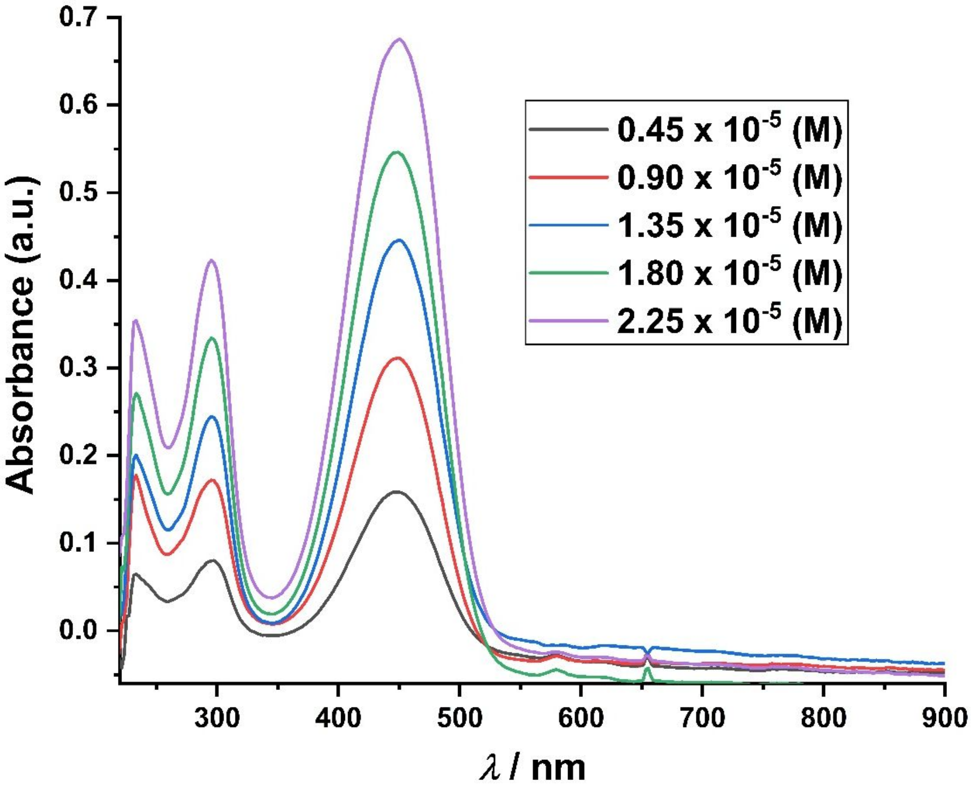

Figure S40. Uv–vis–NIR spectra of **2**$^{\mathbf{Ph}}$ in DCM at room temperature.

.

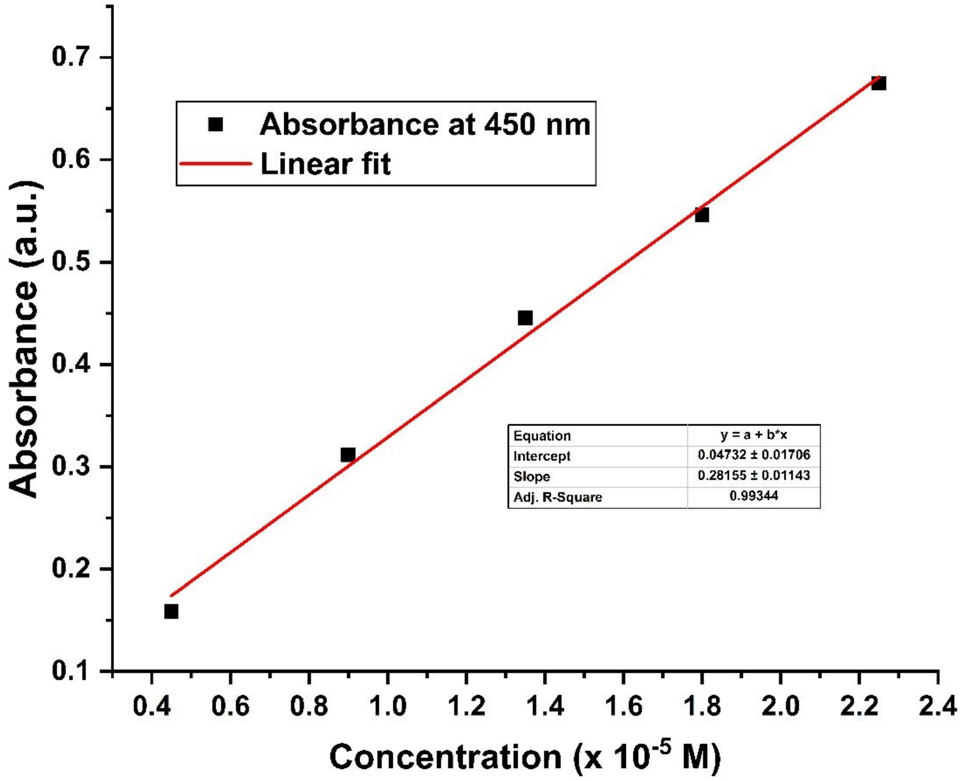

Figure S41. Linear regression of 2^{Ph} at 450 nm.

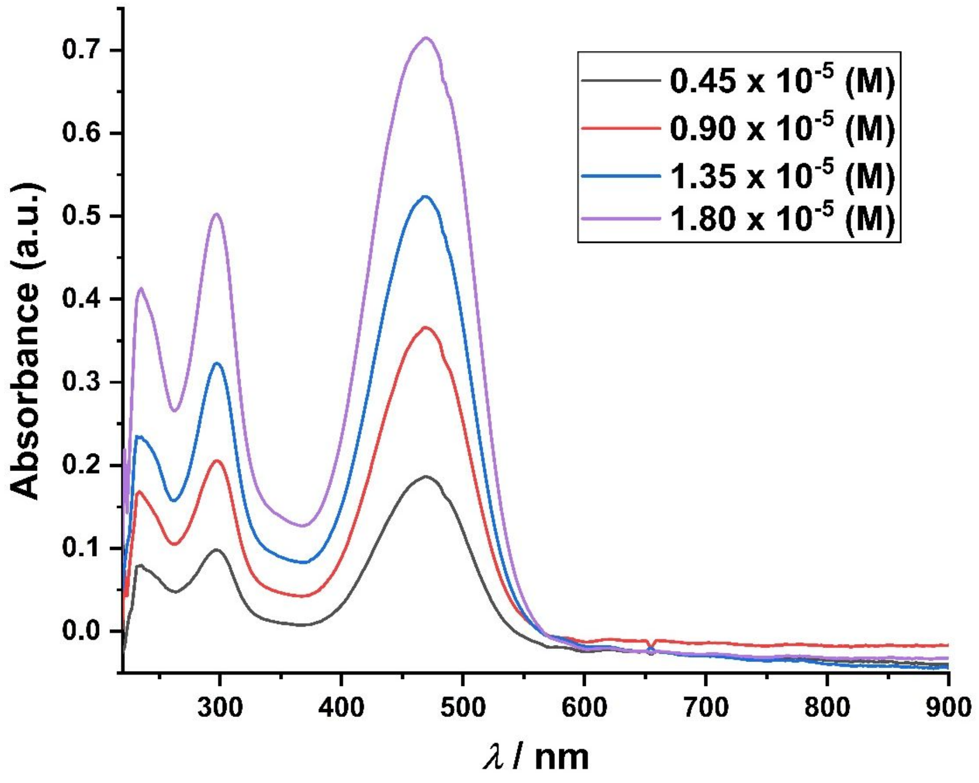

Figure S42. Uv–vis–NIR spectra of 2^{Py} in DCM at room temperature.

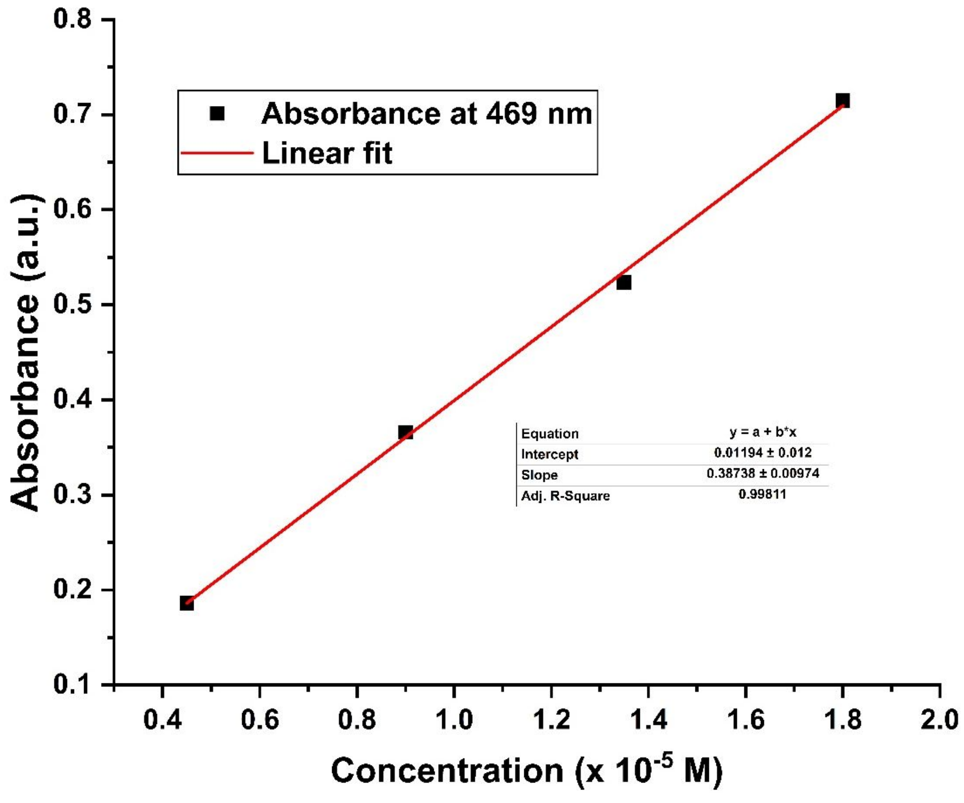

Figure S43. Linear regression of 2^{Py} at 469 nm.

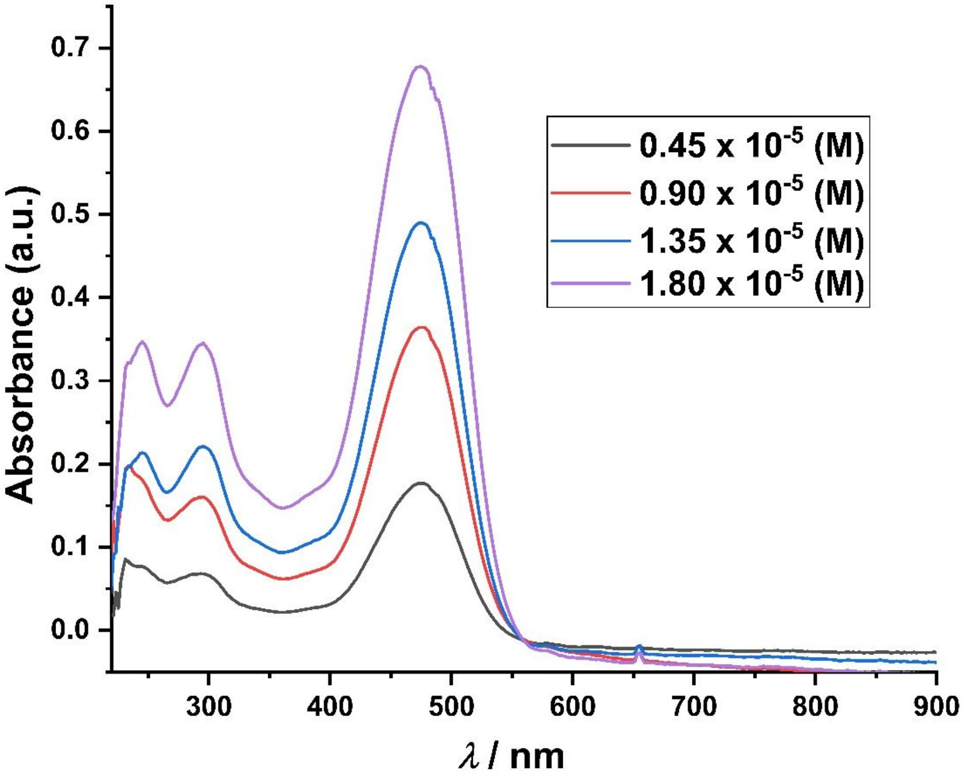

Figure S44. Uv–vis–NIR spectra of **2**^{Pz} in DCM at room temperature.

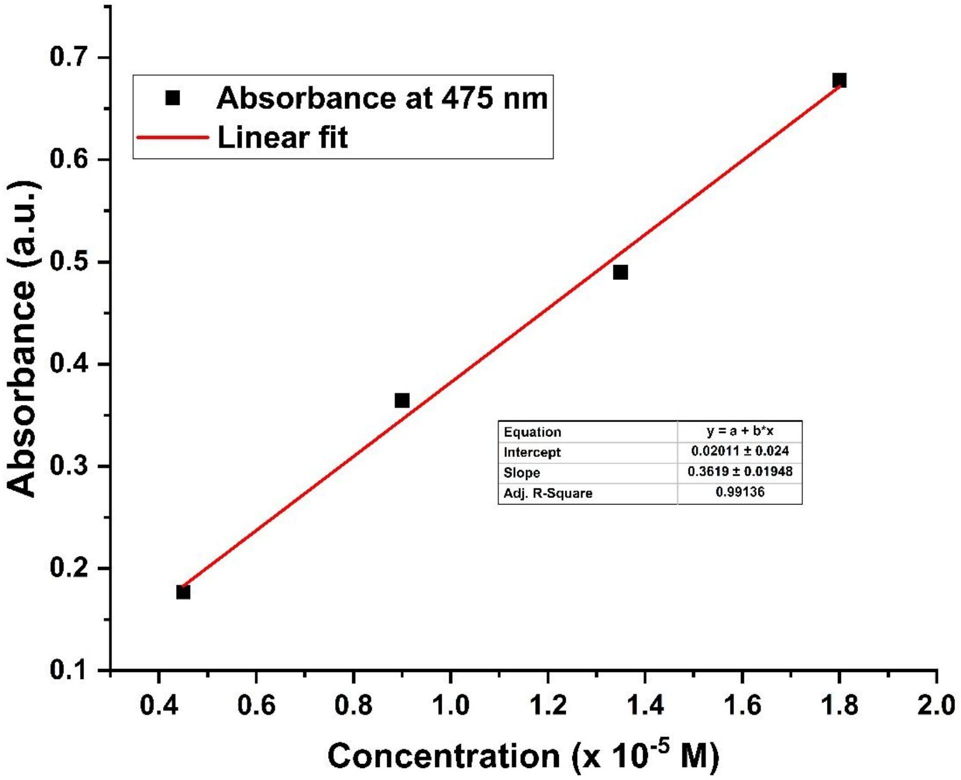

Figure S45. Linear regression of **2**^{Pz} at 475 nm.

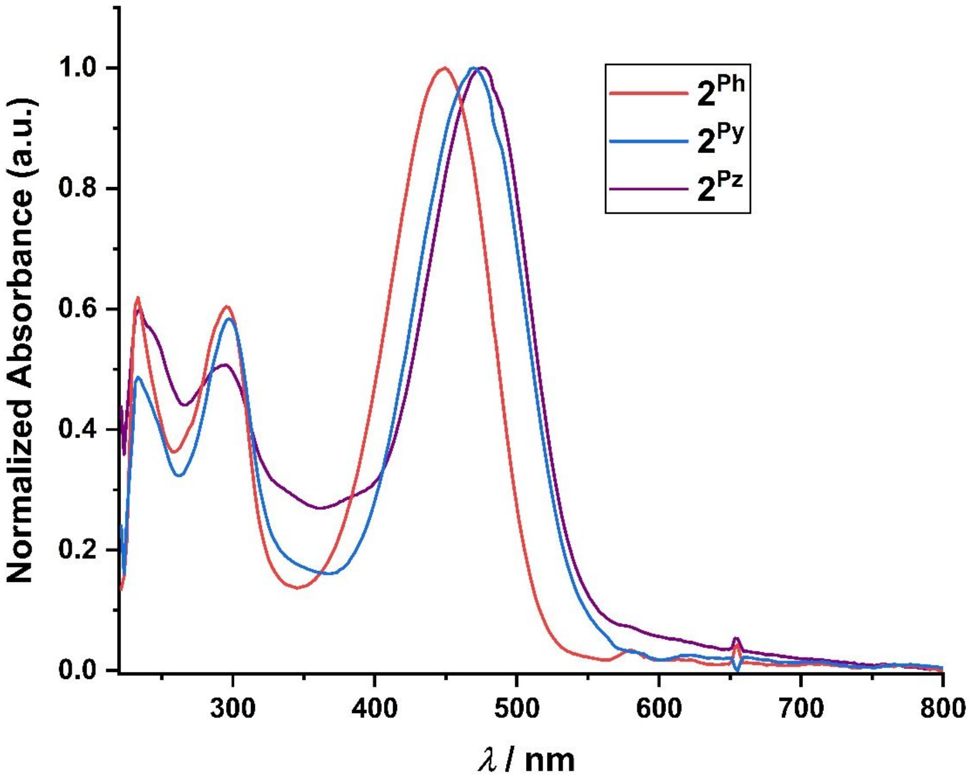

Figure S46. Normalized Uv–vis–NIR spectra of **2^{Ph}**, **2^{Py}**, and **2^{Pz}** in DCM at room temperature.

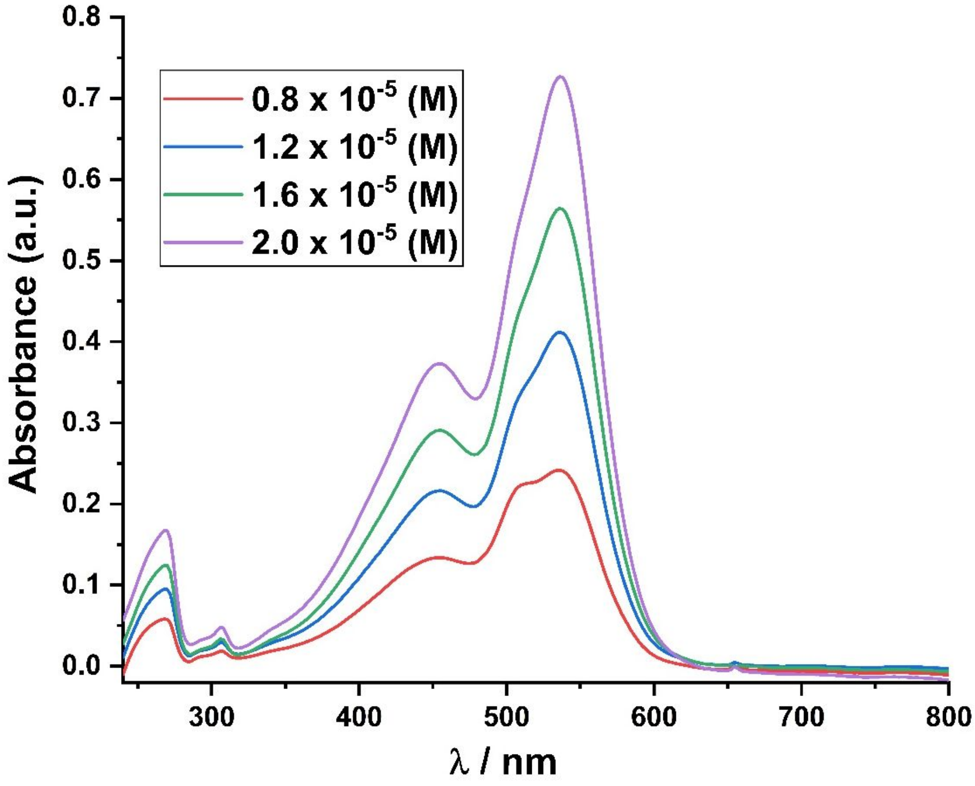

Figure S47. Uv–vis–NIR spectra of **2^{Ph}DC** in DCM at room temperature.

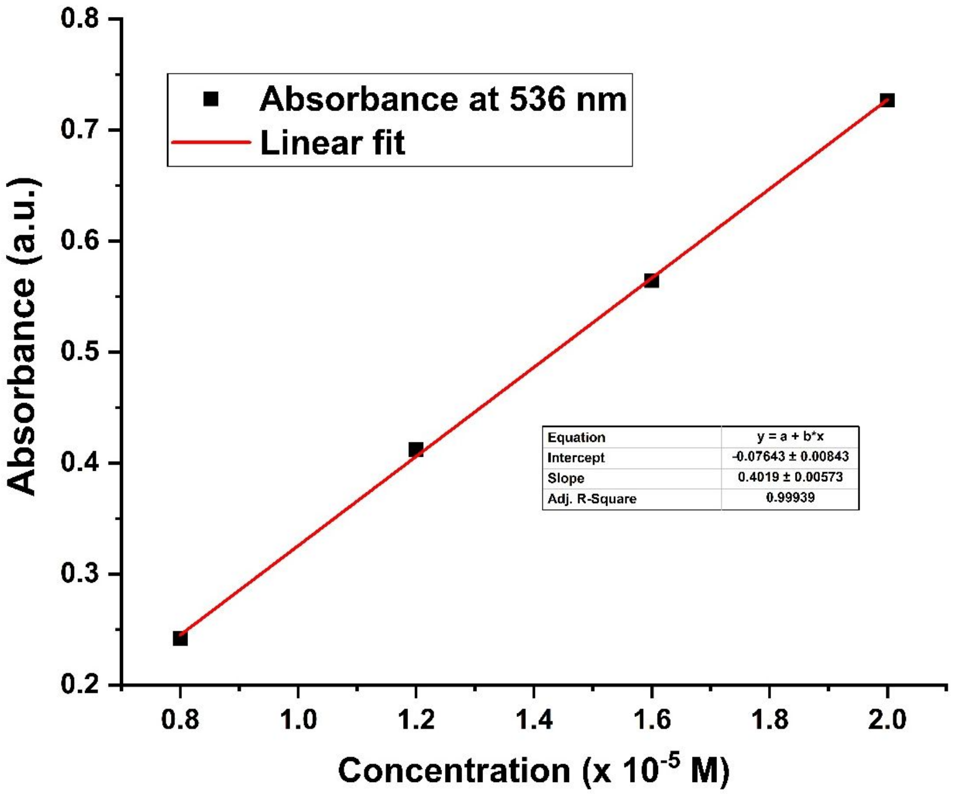

Figure S48. Linear regression of **2^Ph^DC** at 536 nm.

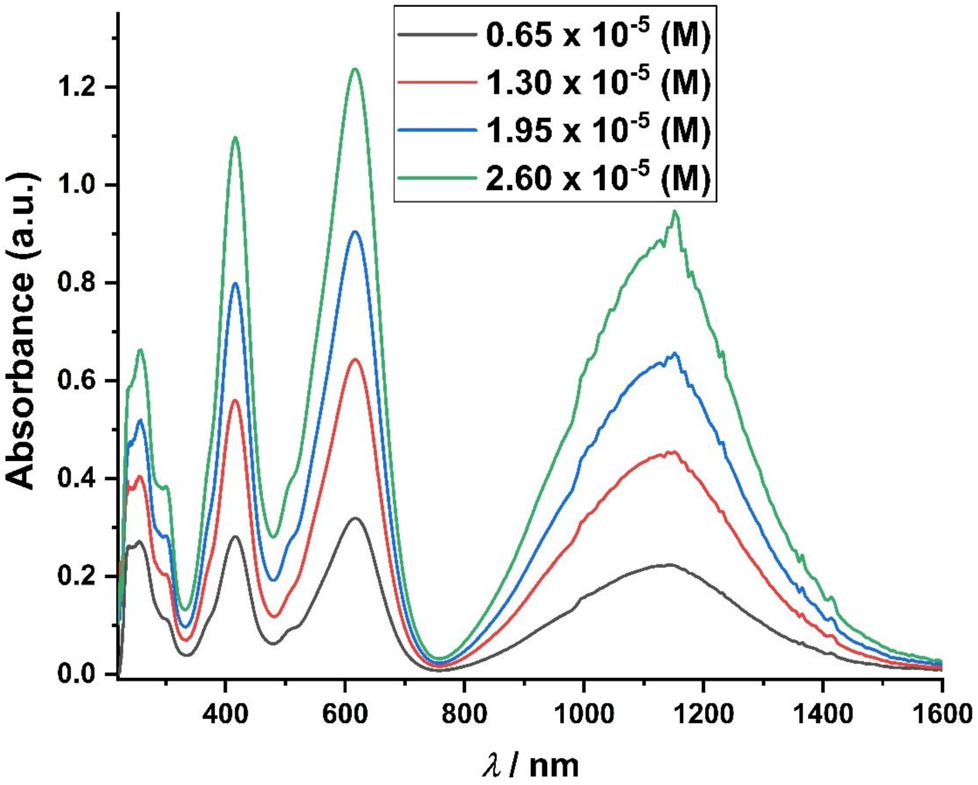

Figure S49. Uv–vis–NIR spectra of **2^Ph^RC** in DCM at room temperature.

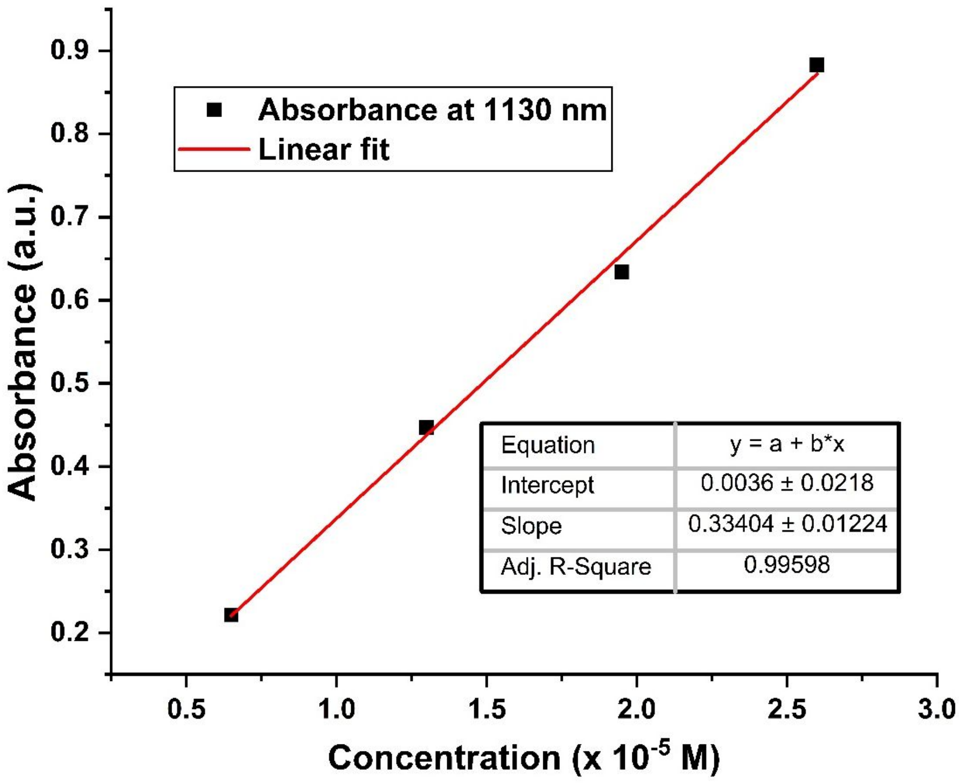

Figure S50. Linear regression of **2^{Ph}RC** at 1130 nm.

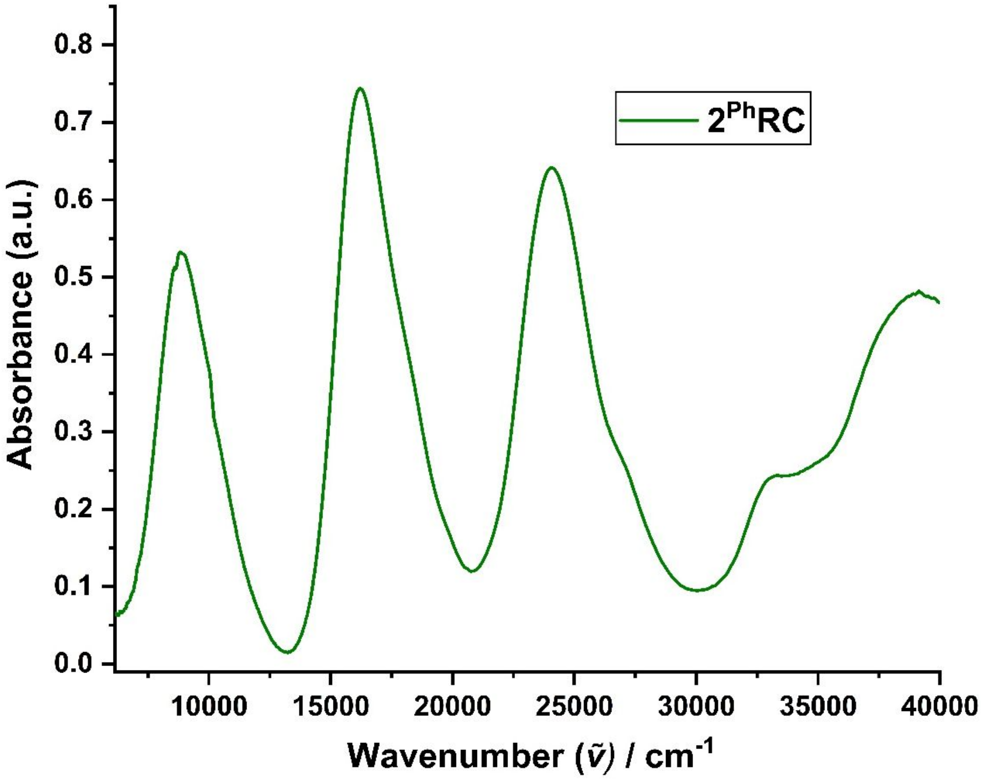

Figure S51. Uv–vis–NIR spectra of **2^{Ph}RC** vs wavenumber in DCM at room temperature.

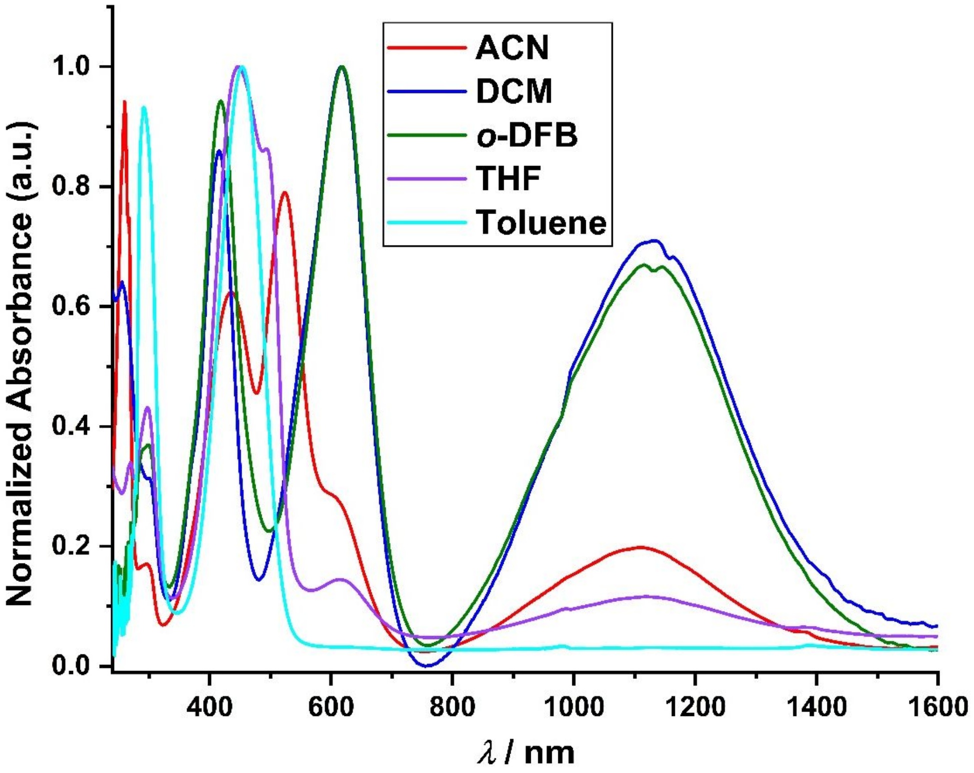

Figure S52. Normalized Uv–vis–NIR spectra of **2^{Ph}RC** in different solvent at room temperature.

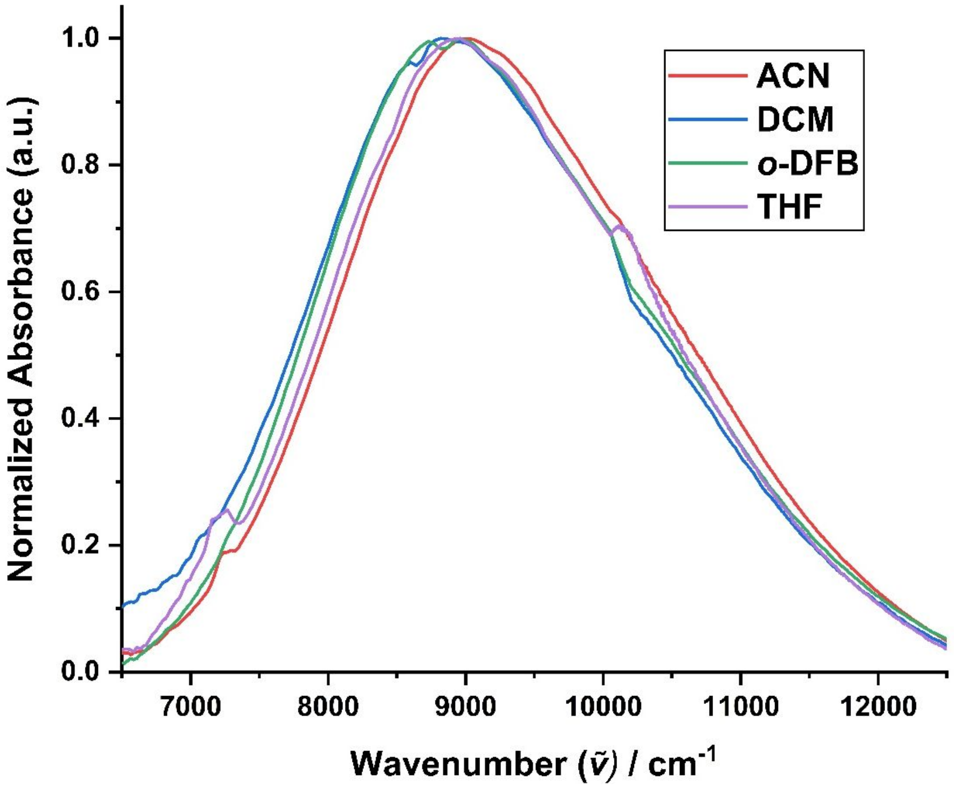

Figure S53. Normalized IV-CT band vs wavenumber from the Uv–vis–NIR spectra of **2^{Ph}RC** in different solvent at room temperature.

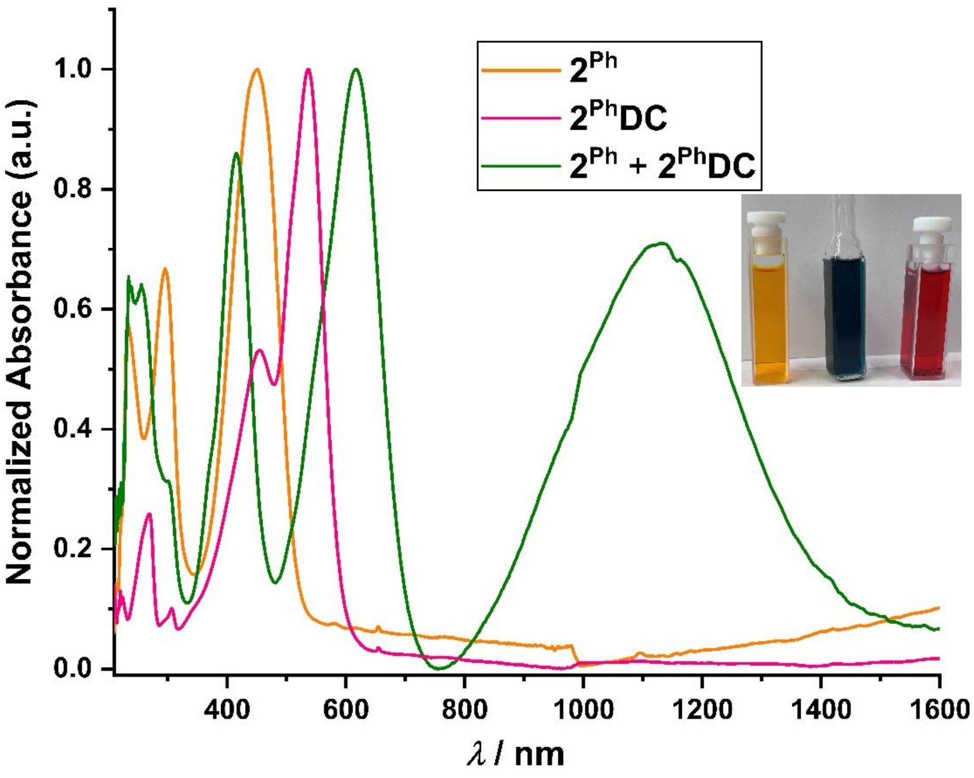

Figure S54. Normalized Uv–vis–NIR spectra of **2**^{Ph}, **2**^{Ph}**DC**, and **2**^{Ph}**RC** in DCM at room temperature. Photograph of **2**^{Ph},(orange), **2**^{Ph}**RC** (green) and **2**^{Ph}**DC** (pink-red) (inset).

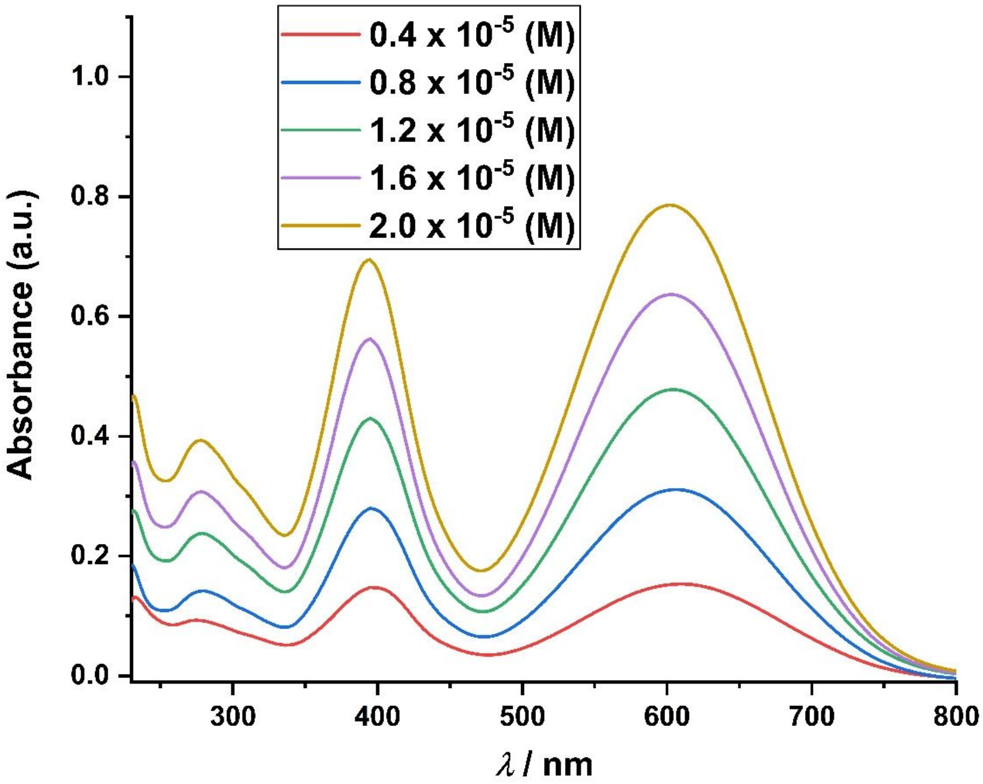

Figure S55. Uv–vis–NIR spectra of **3**^{Py} in DCM at room temperature.

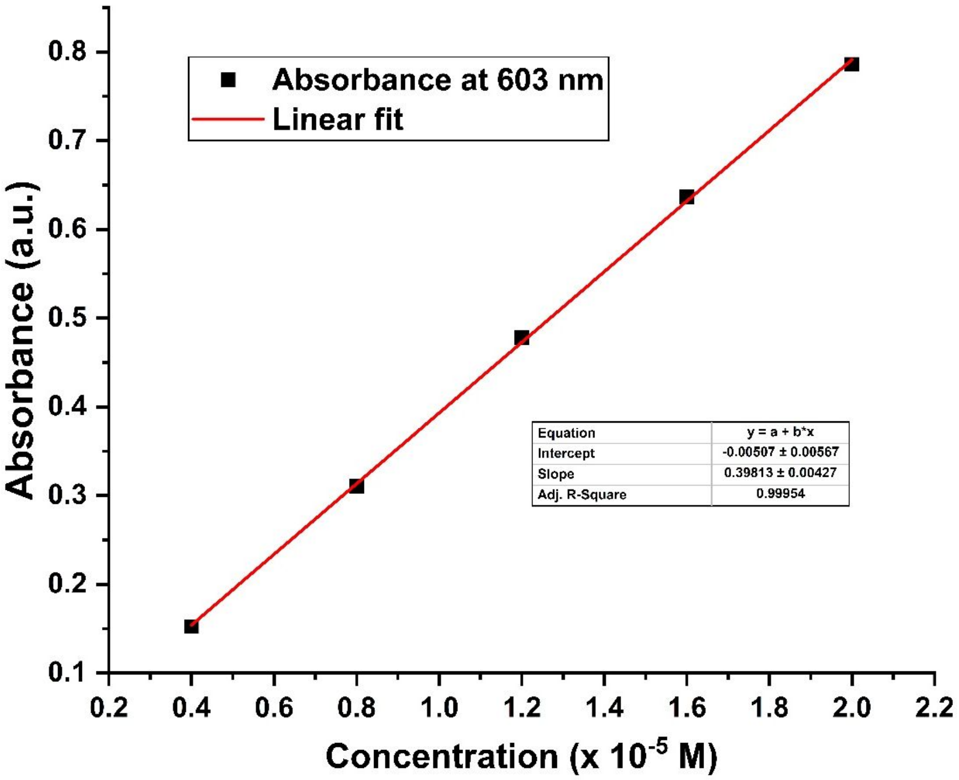

Figure S56. Linear regression of 3^{Py} at 603 nm.

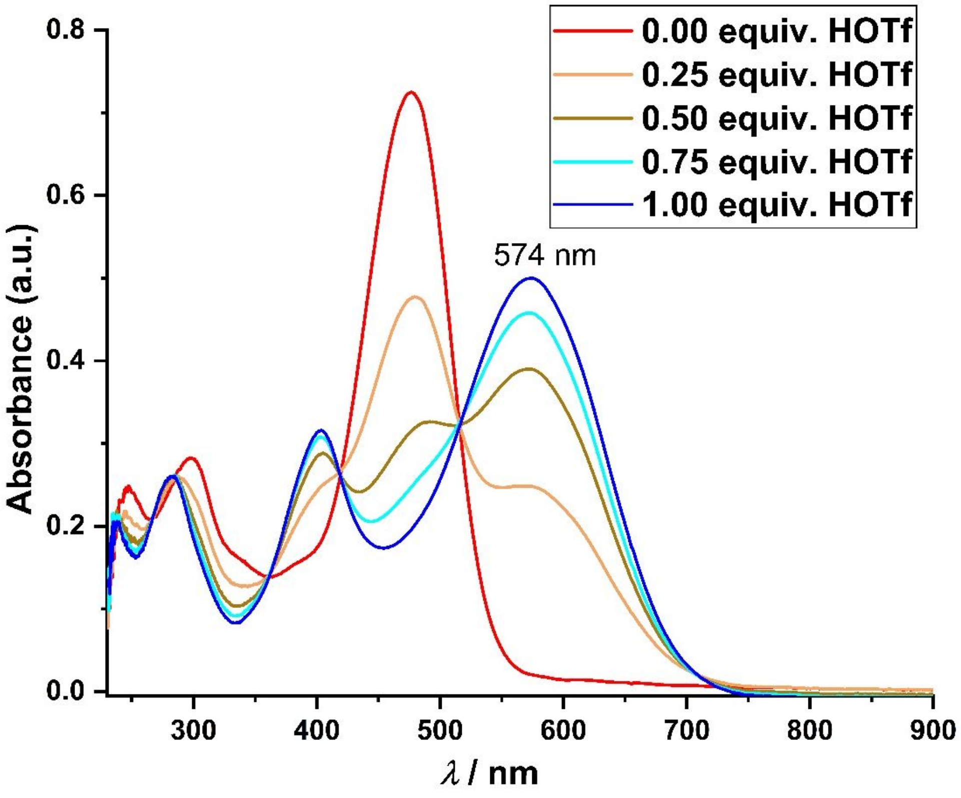

Figure S57. Changes in the Uv–vis–NIR spectra of 2^{Pz} in DCM upon addition of DMF·HOTf to form 3^{Pz} at room temperature.

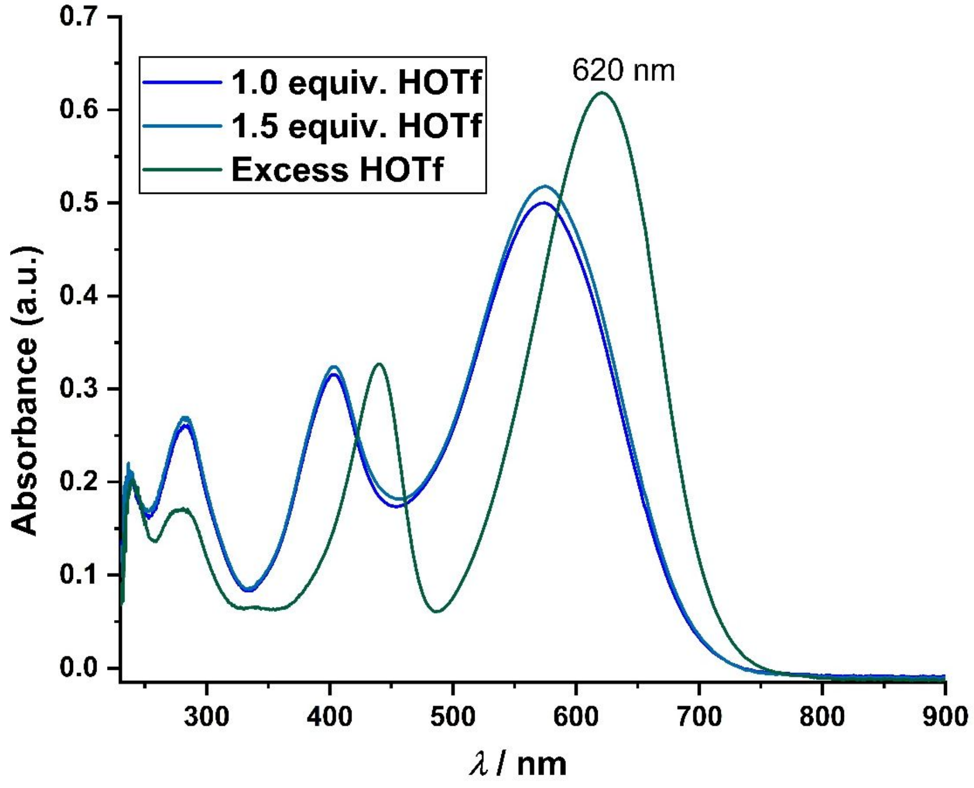

Figure S58. Changes in the Uv–vis–NIR spectra of 3^{Pz} in DCM upon addition of DMF·HOTf to form 4^{Pz} at room temperature.

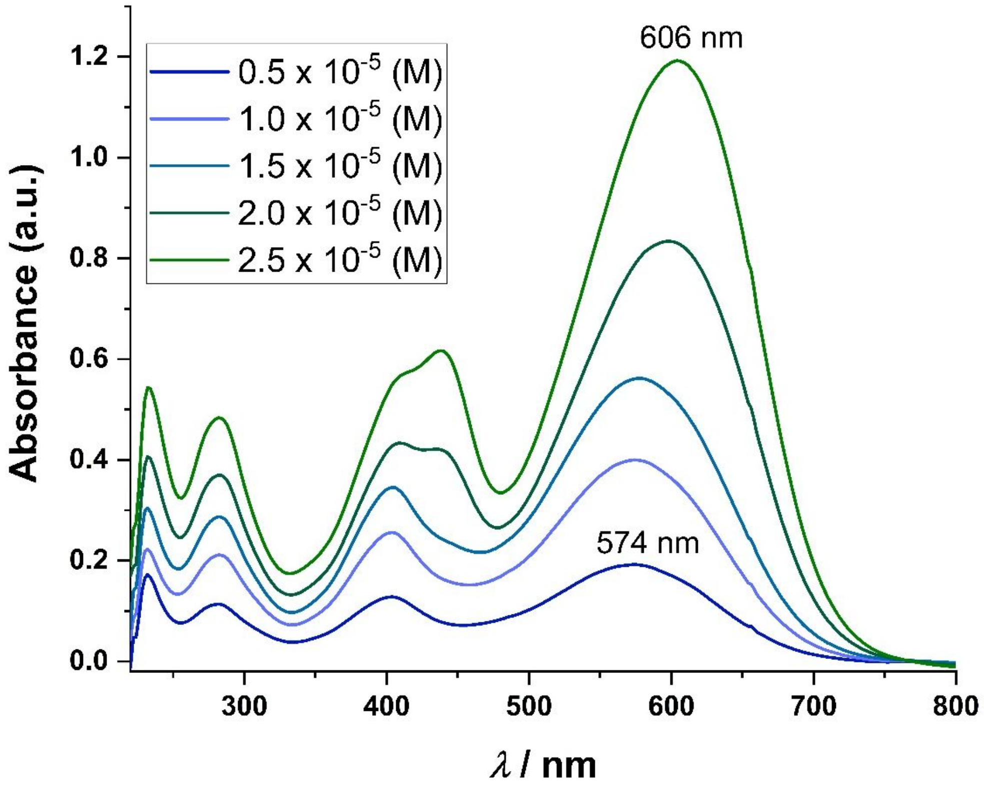

Figure S59. Changes in the Uv–vis–NIR spectra of 4^{Pz} in DCM upon dilution to form 3^{Pz} at room temperature.

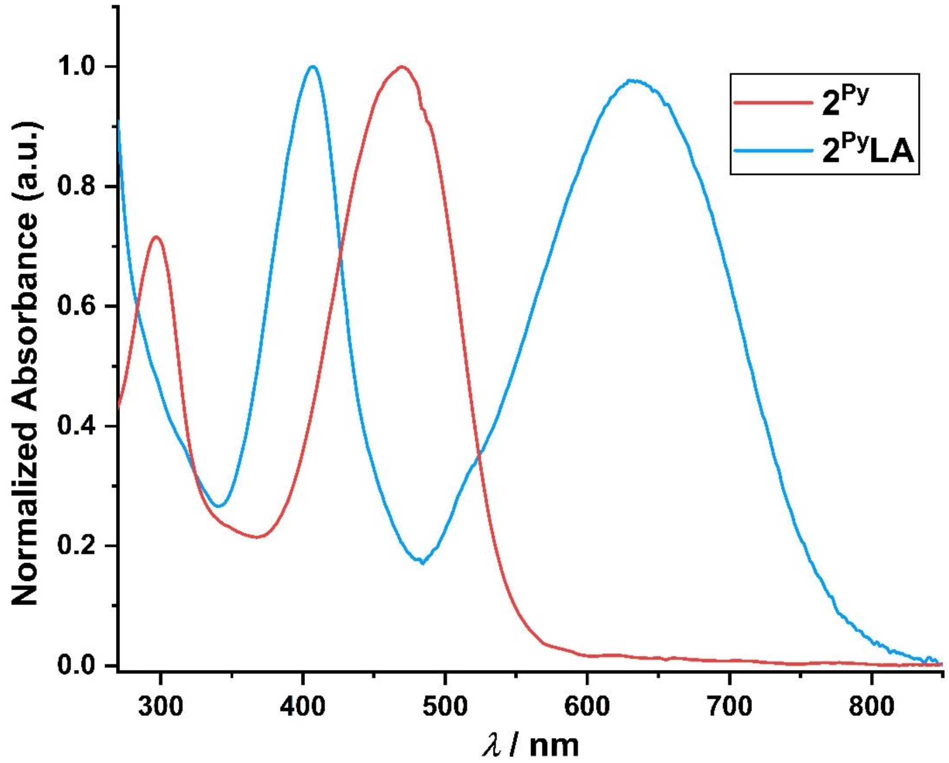

Figure S60. Normalized Uv–vis–NIR spectra of **2**^{Py} and **2**^{Py}**LA** in DCM at room temperature.

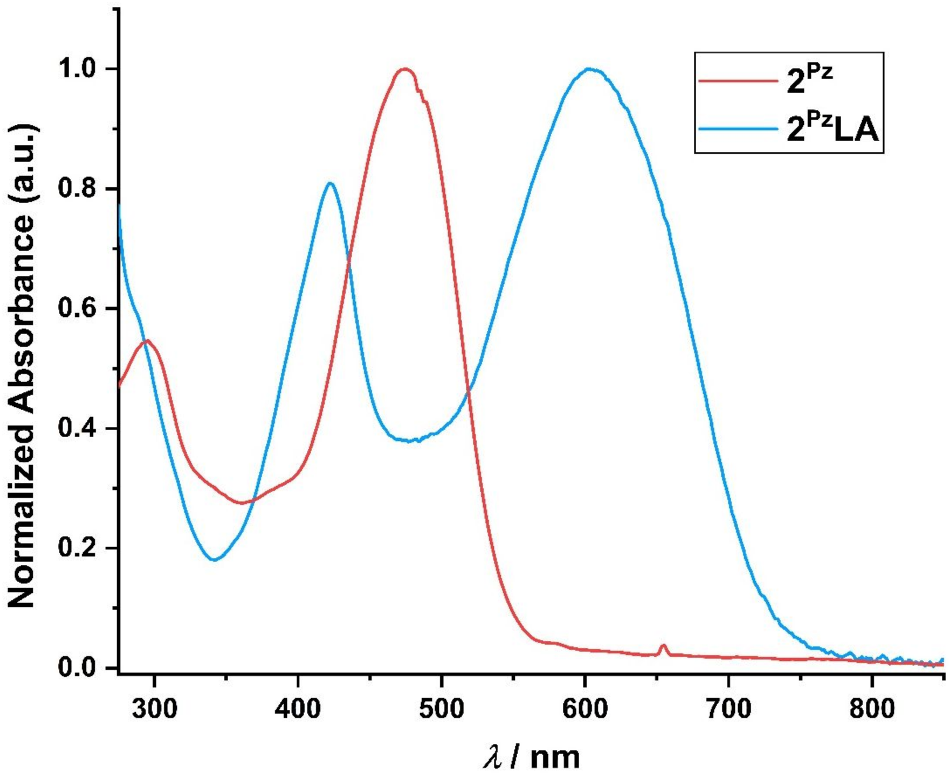

Figure S61. Normalized Uv–vis–NIR spectra of **2**^{Pz} and **2**^{Pz}**LA** in DCM at room temperature.

7. Emission Spectroscopy

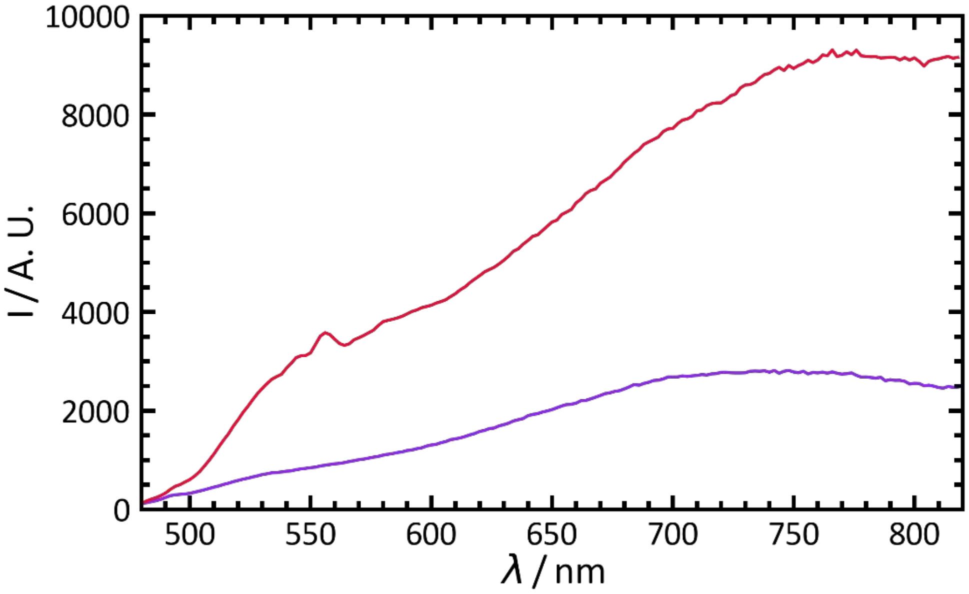

Figure S62. Emission spectra of **2**^{Ph} (excitation wavelength 458 nm, c = 11.6 µmol/L) in DCM at 293 K (violet) and 185 K (red).

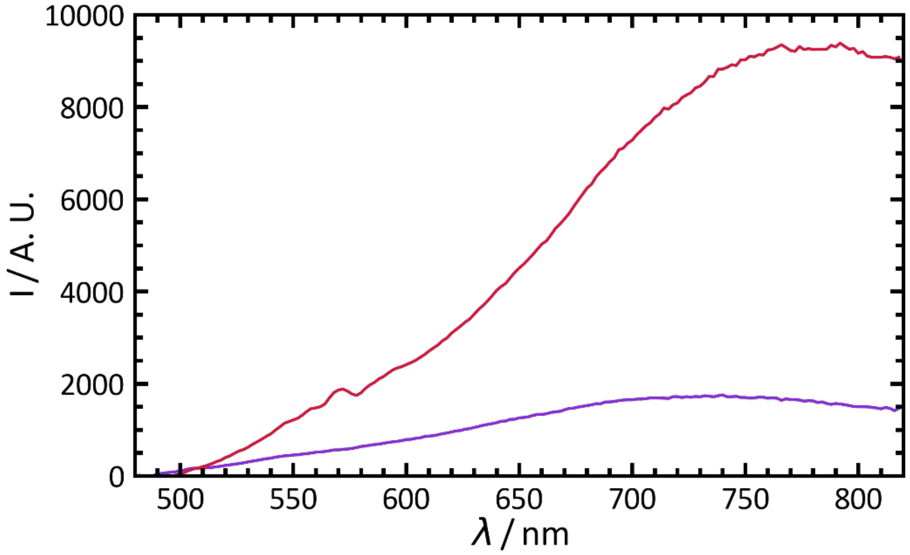

Figure S63. Emission spectra of **2**^{Py} (excitation wavelength 469 nm, c = 10.2 µmol/L) in DCM at 293 K (violet) and 185 K (red).

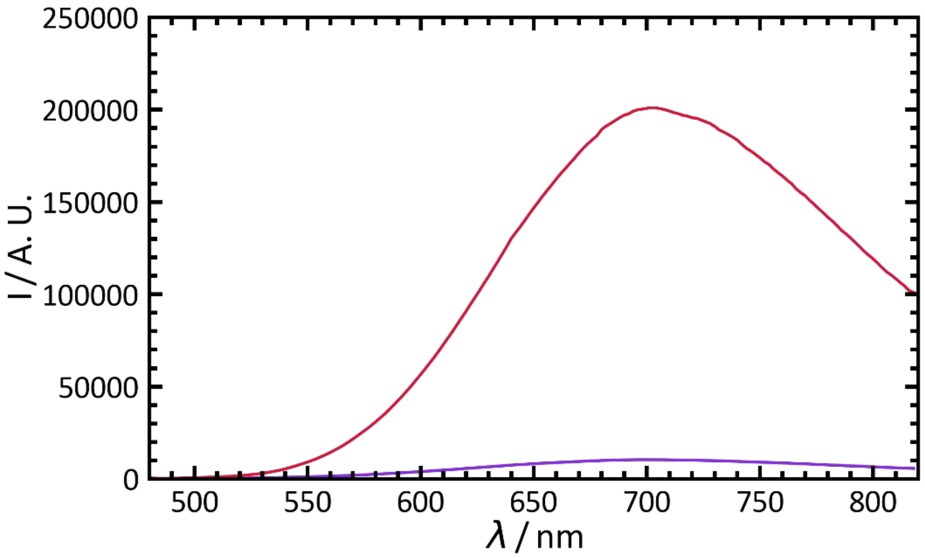

Figure S64. Emission spectra of **2**^{Py} (excitation wavelength 469 nm, c = 11.9 µmol/L) in toluene at 293 K (violet) and 185 K (red).

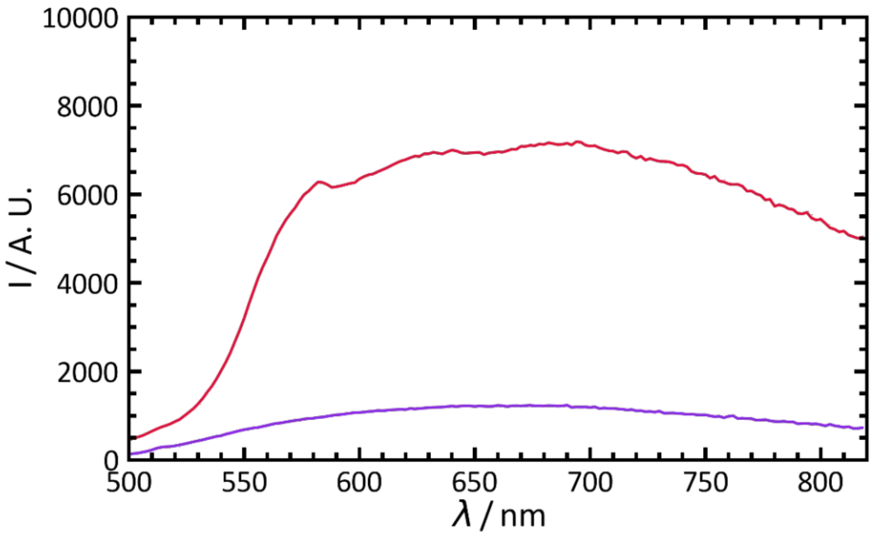

Figure S65. Emission spectra of **2**^{Pz} (excitation wavelength 476 nm, c = 10.0 µmol/L) in DCM at 293 K (violet) and 185 K (red).

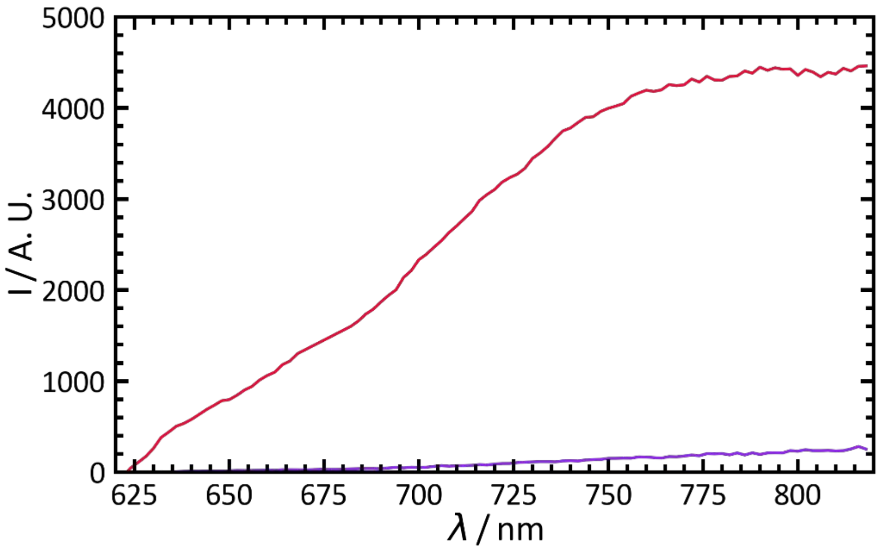

Figure S66. Emission spectra of 3^{Py} (excitation wavelength 599 nm, c = 22.7 µmol/L) in DCM at 293 K (violet) and 185 K (red).

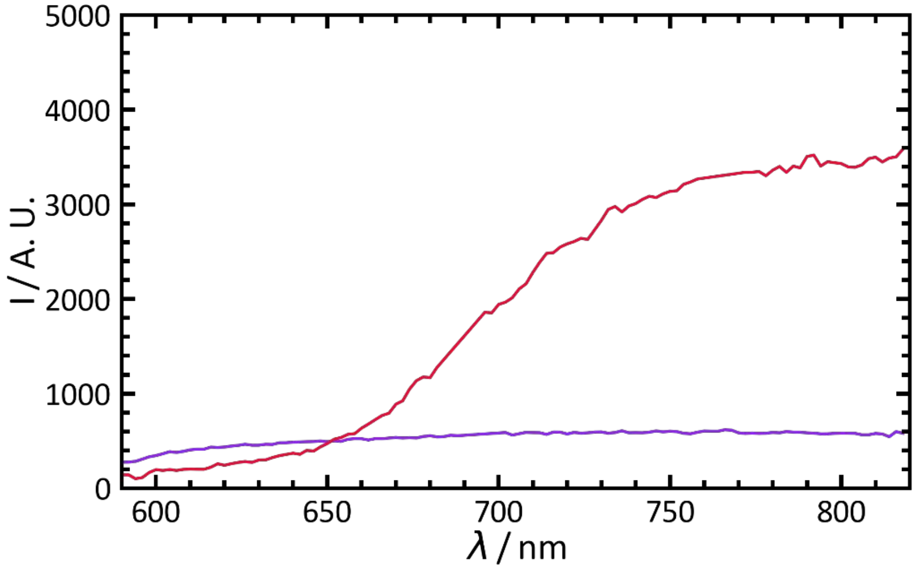

Figure S67. Emission spectra of 3^{Pz} (excitation wavelength 569 nm, c = 16.9 µmol/L) in DCM at 293 K (violet) and 185 K (red).

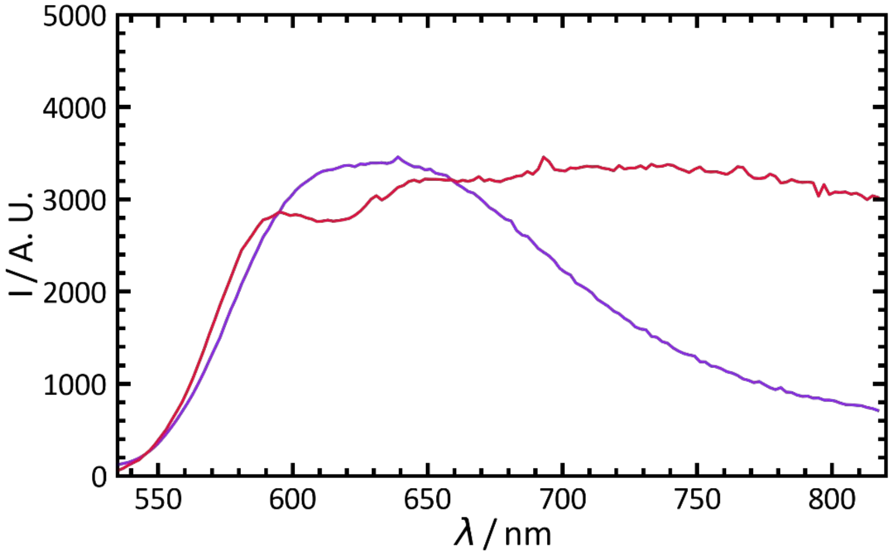

Figure S68. Emission spectra of 3^{Pz} (excitation wavelength 515 nm, c = 12.9 µmol/L) in DCM at 293 K (violet) and 185 K (red).

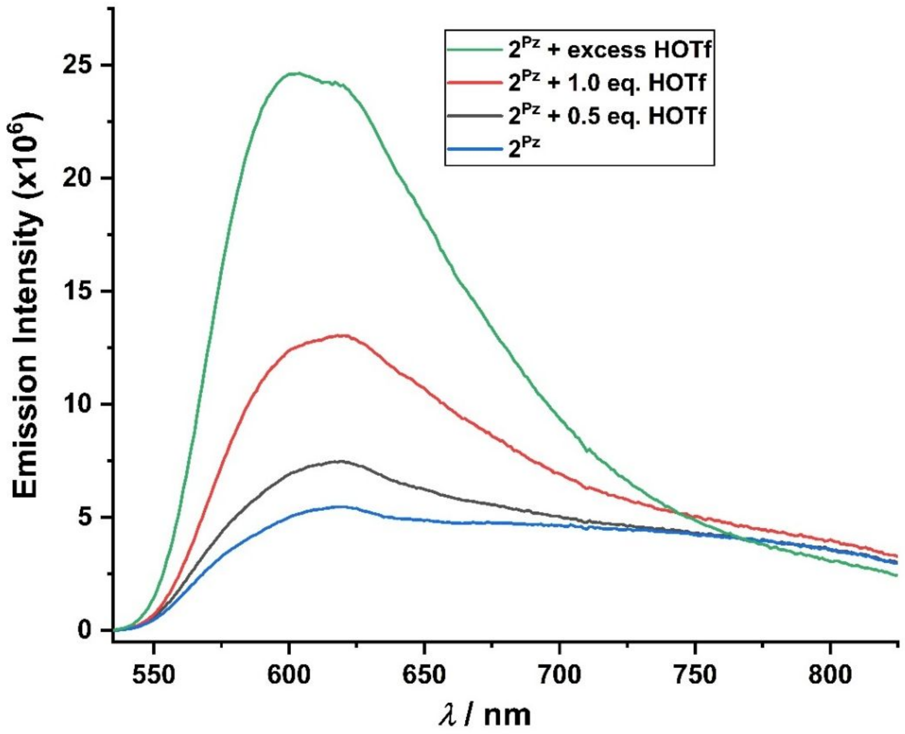

Figure S69. Changes in the emission spectra of $\mathbf{2^{Pz}}$ (excitation wavelength 515 nm) in DCM upon addition of DMF·HOTf at 293 K.

8. Cyclic voltammetry and UV-Vis-NIR Spectro-electrochemistry

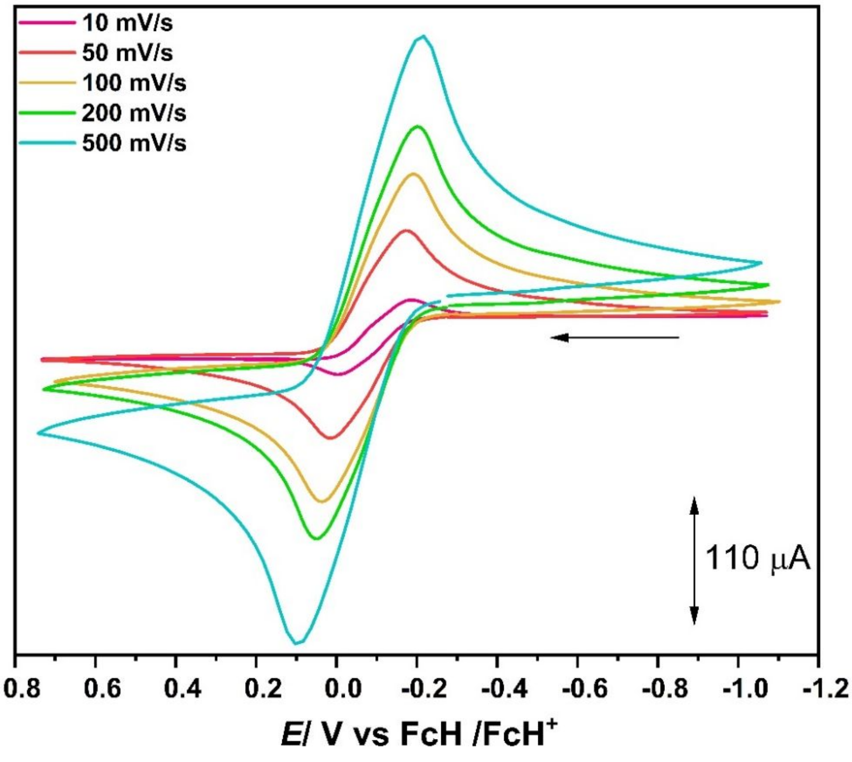

Figure S70. Cyclic voltammograms of $\mathbf{2^{Ph}}$ with 0.1 M NBu_4PF_6 in DCM at different scan rate.

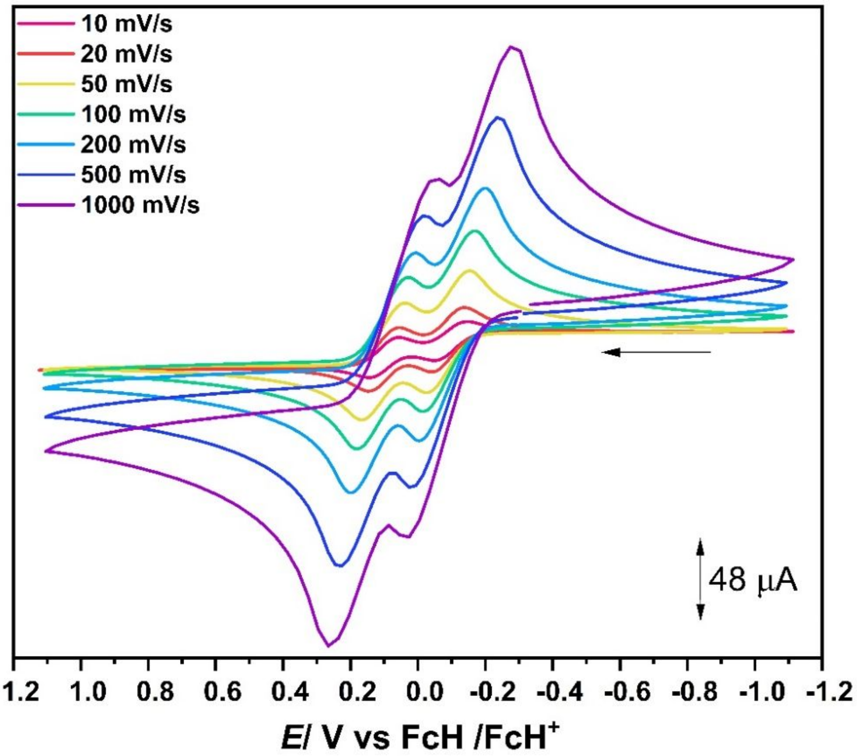

Figure S71. Cyclic voltammograms of $\mathbf{2}^{\mathbf{Ph}}$ with 0.02 M NBu_4BArF_{24} in DCM at different scan rate.

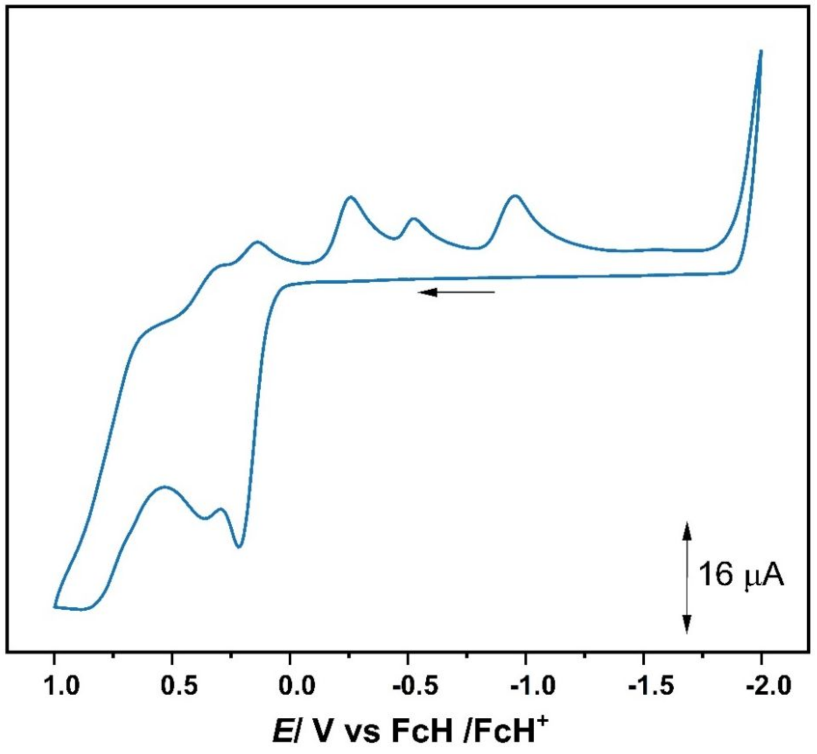

Figure S72. Cyclic voltammogram of $\mathbf{2}^{\mathbf{Py}}$ with 0.02 M NBu_4BArF_{24} in DCM at 100 mV/s scan rate.

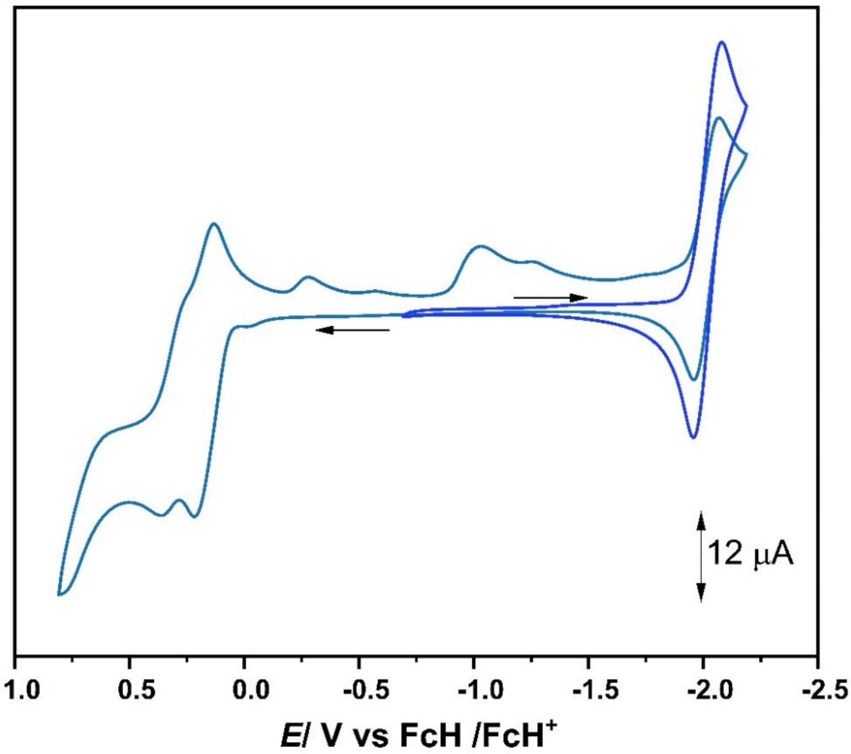

Figure S73. Cyclic voltammograms of 2^{Pz} with 0.02 M NBu_4BArF_{24} in DCM at 100 mV/s scan rate.

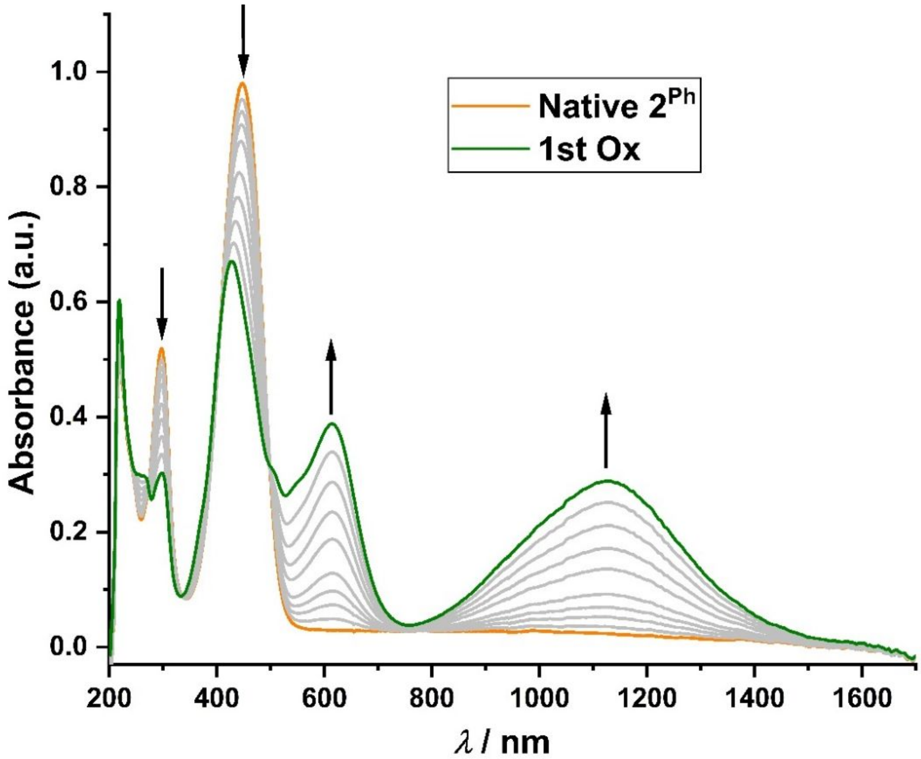

Figure S74. Changes in the Uv-vis-NIR spectra of 2^{Ph} in DCM/0.2M Bu_4NPF_6 during the 1st oxidation with an Au working electrode.

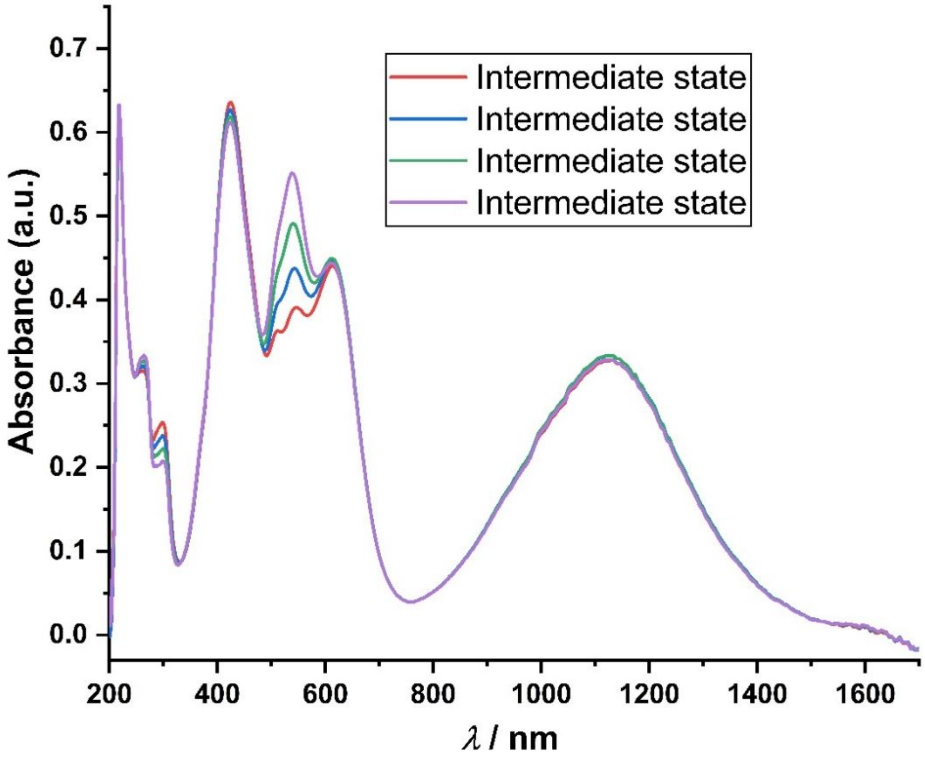

Figure S75. Changes in the Uv-vis-NIR spectra of **2^{Ph}** in DCM/0.2M Bu_4NPF_6 in between 1st and 2nd oxidation when disproportionation reaction competes with redox reaction.

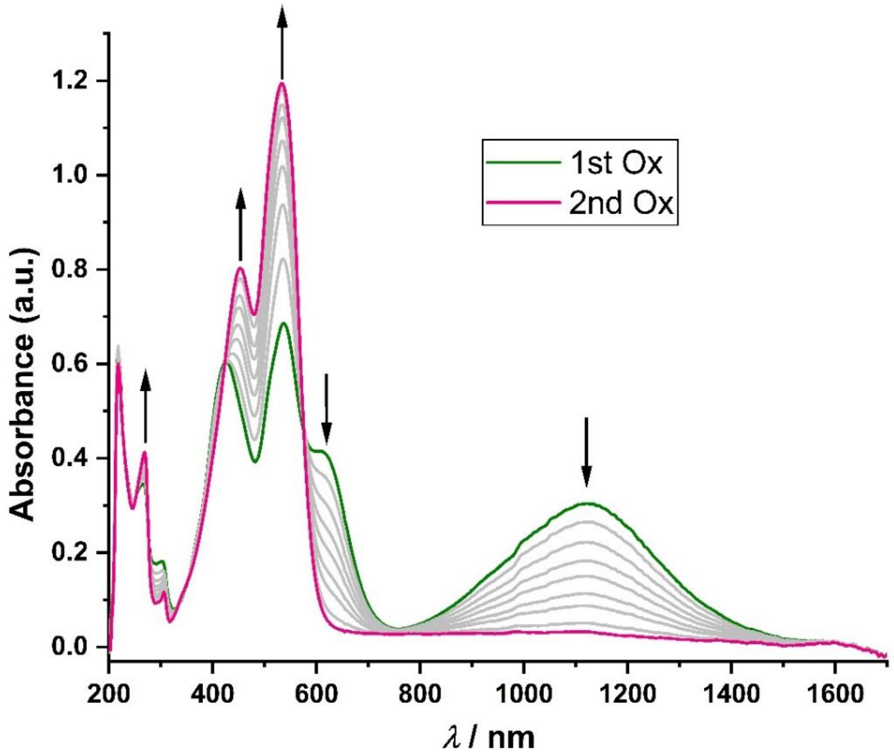

Figure S76. Changes in the Uv-vis-NIR spectra of **2^{Ph}** in DCM/0.2M Bu_4NPF_6 during the 2nd oxidation with an Au working electrode.

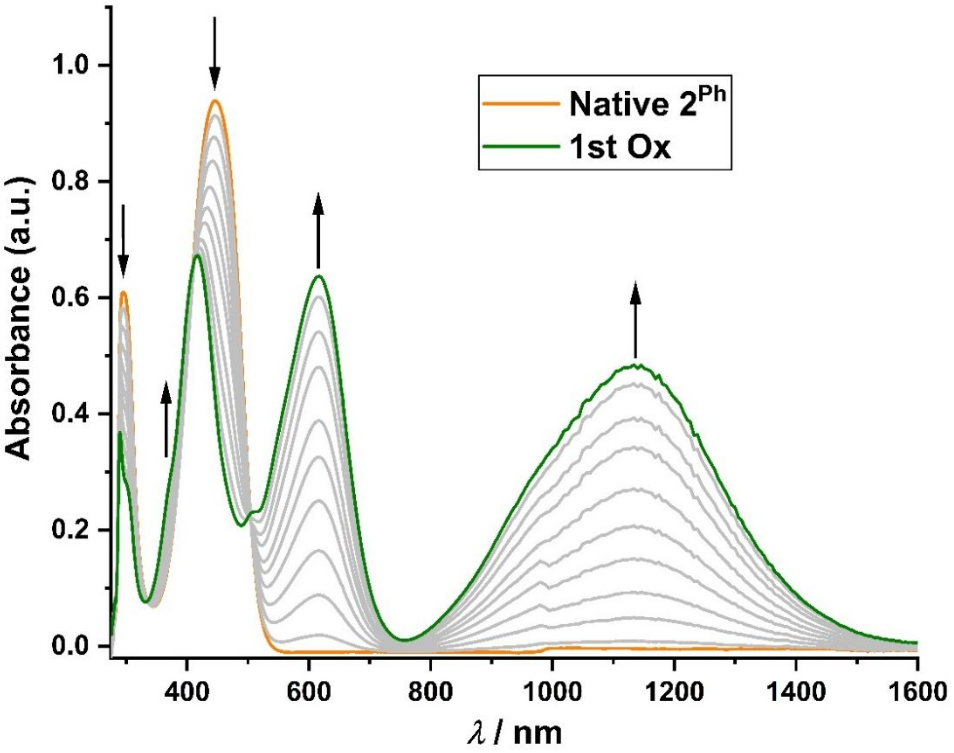

Figure S77. Changes in the Uv-vis-NIR spectra of $\mathbf{2^{Ph}}$ in DCM/0.1M Bu_4NBArF_{24} during the 1st oxidation with an Au working electrode.

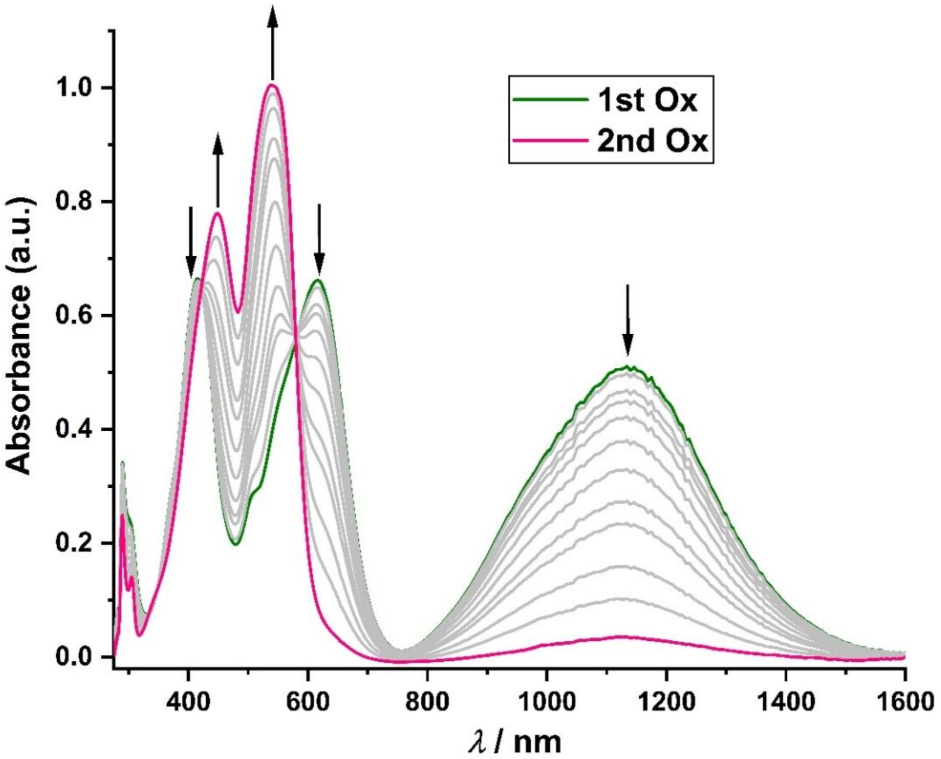

Figure S78. Changes in the Uv-vis-NIR spectra of $\mathbf{2^{Ph}}$ in DCM/0.1M Bu_4NBArF_{24} during the 2nd oxidation with an Au working electrode.

9. EPR Spectroscopy

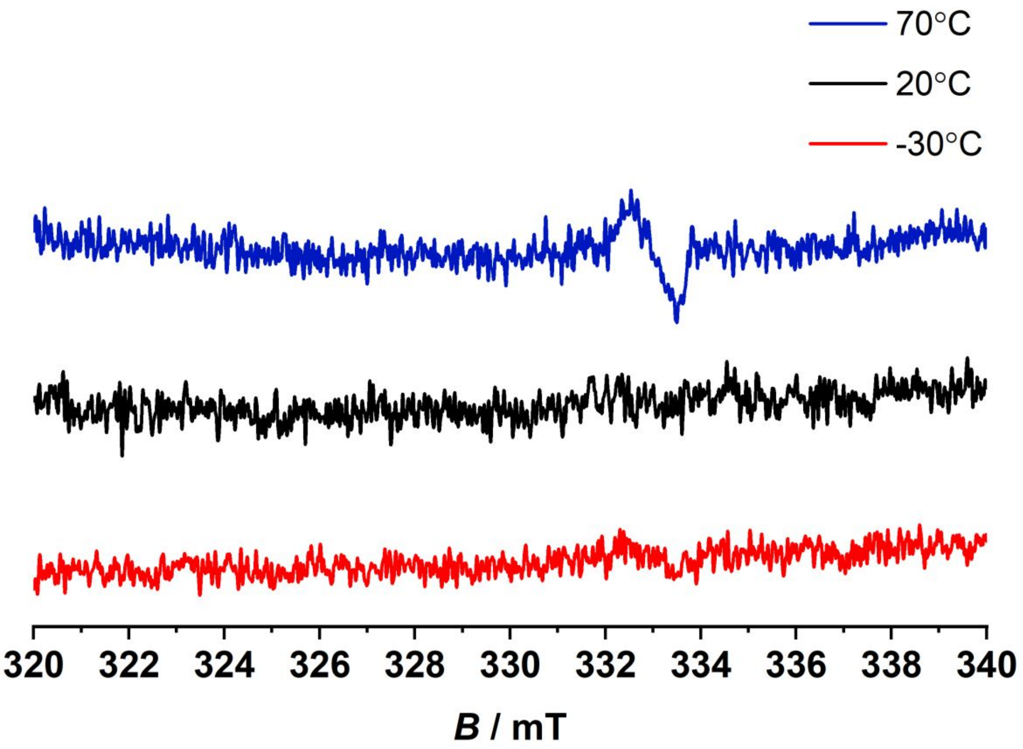

Figure S79. Variation temperature EPR-Spectrum of DCE solution of **2^{Ph}**.

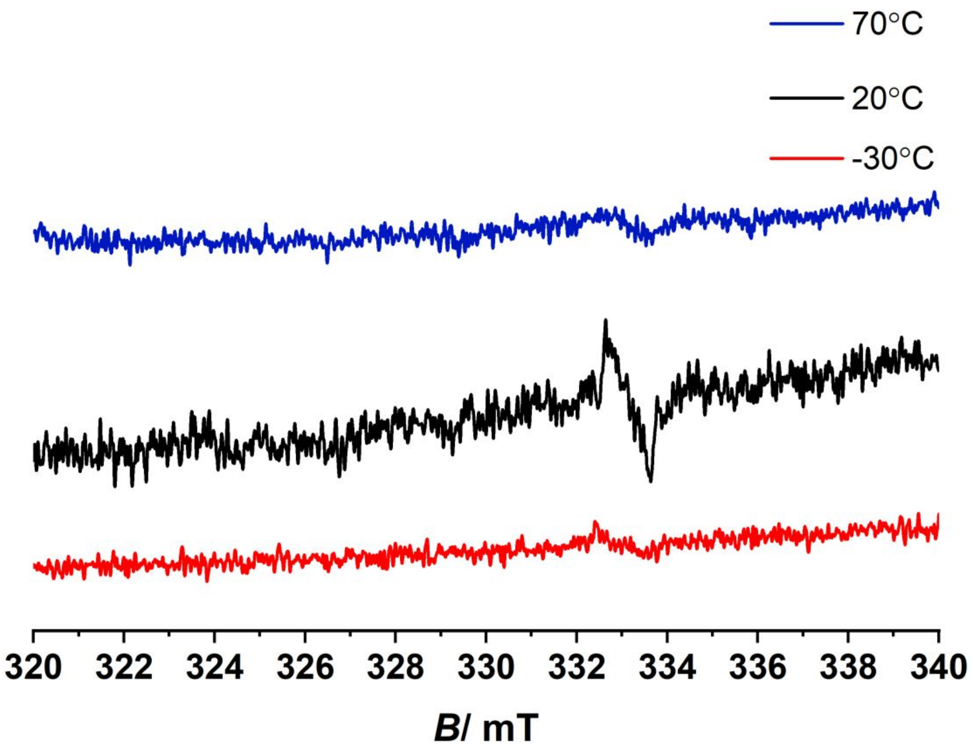

Figure S80. Variation temperature EPR-Spectrum of DCE solution of **2^{Py}**

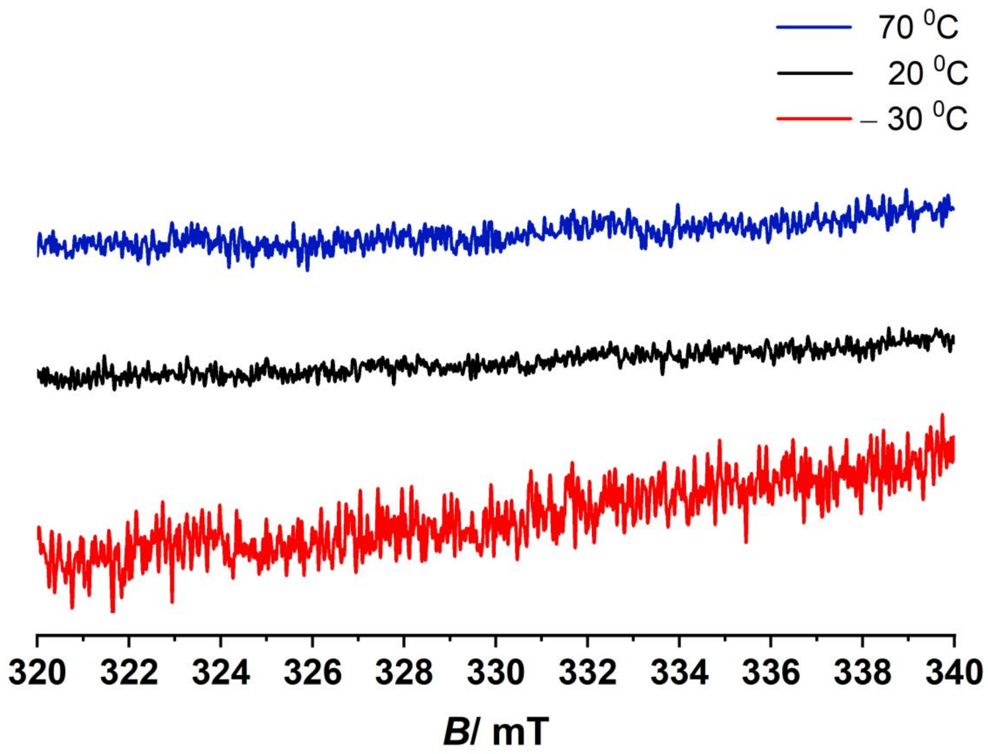

Figure S81. Variation temperature EPR-Spectrum of DCE solution of **2^{Pz}**.

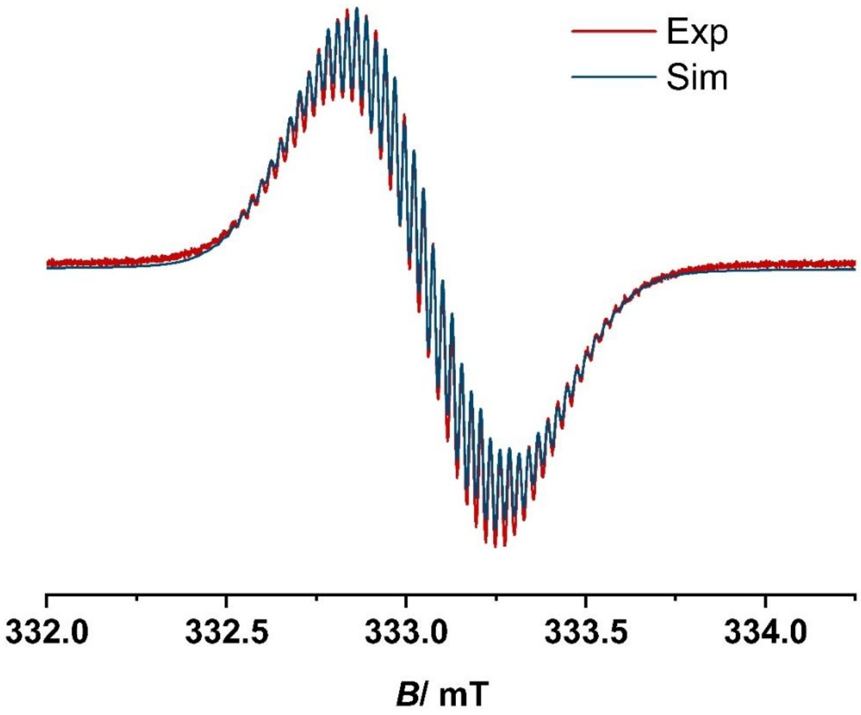

Figure S82. Experimental (red) and simulated (blue) X-band EPR spectra of **2^{Ph}RC** obtained from comproportionation reaction of **2^{Ph}** and **2^{Ph}DC** in DCM. Best-fit simulation parameters: g_{iso} = 2.0031, lwpp =0.0315, a(4H) = 4.4449 MHz (Ph-*H*), a(8H) = 2.2435 MHz (*o*-Ar-*H*), and a(8H) = 0.7417 MHz (*m*-Ar-*H*).

10. Computational Details

Theoretical calculations were performed using ORCA 5.0 and 6.0.[8] Geometry optimisations and frequencies were calculated starting from experimentally determined molecular structures with the PBE0[9] functional and def2-TZVP[10] basis sets. The optimized geometries were employed for single-point and TD-DFT calculations with the PBE0[9] functional and def2-TZVP[10] basis sets. Implicit solvation was approximated using the SMD[11] method together with the CPCM[12] model. CH_2Cl_2 was used as the solvent of choice unless stated otherwise. Population analysis were determined from Löwdin reduced orbital populations. Resolution-of-the-identity (RI) approximations[13] with matching basis-sets (def2/J)[14] were employed to reduce time of calculation. Grimme's D3 method was used for dispersion correction.[15] The visualization software *Chemcraft* was used to plot orbital and electron density figures.[16] $\Delta E_{\mathrm{CSS\text{-}OST}}$ were calculated based on final single point energy of the corresponding diradicaloid optimised corresponding closed shell singlet (CSS) and open shell triplet (OST) state. $\Delta E_{\mathrm{HOMO\text{-}LUMO}}$ were calculated from optimized structure of the diradicaloids in ground state CSS form.[17] The energy of S_1 and T_1 state were calculated using TDDFT with the same functional and basis set based on optimized geometry. For **2**$^{\mathbf{Ph}}$, **2**$^{\mathbf{Py}}$ and **2**$^{\mathbf{Pz}}$, we also performed DFT and CASSCF/NEVPT2 calculations studying the elongation of the C1-C2 (C1'-C2') bonds, for which we used the "relaxed surface scan" feature in Orca, together with the "simul_scan true" option, allowing us to simultaneously elongate both bonds. For each of the structures in the distance scan, we performed single point calculations of the CSS, the OST and the OSS (using the "brokensym" option in Orca). We employed natural orbitals from the broken-symmetry calculations to calculate the diradical character for the three neutral molecules, as well as mono- and diporotonated **2**$^{\mathbf{Pz}}$. For selected initial, intermediate and final structures we also performed CASSCF calculations employing an active space of 2 electrons in 2 orbitals, state averaging over one triplet and three singlet roots, which allowed us to determine the different weights of closed-shell and open-shell configurations in the ground and excited singlet states. For **2**$^{\mathbf{Py}}$ we also studied different active spaces: CAS(4,4), CAS(6,6), and in one case CAS(8,8).

Table S9. Selected thermochemical data of the title structures.

	**2**$^{\mathbf{Ph}}$	**2**$^{\mathbf{Py}}$	**2**$^{\mathbf{Pz}}$	**3**$^{\mathbf{Pz}}$	**4**$^{\mathbf{Pz}}$
E_{CSS} (Hartree)	-1690.6756107	-1706.7098284	-1722.7434185	-1723.1984930	-1723.6282304
E_{OST} (Hartree)	-1690.6541985	-1706.6873906	-1722.7164572	-1723.1650340	-1723.5881418
$\Delta E_{\mathrm{CSS\text{-}OST}}$ (Kcal/mol)	13.44	14.08	16.90	20.99	25.00
E_{HOMO} (eV)	-4.76	-4.89	-5.07	-5.82	-6.56
E_{LUMO} (eV)	-2.09	-2.24	-2.36	-3.28	-3.96
$\Delta E_{\mathrm{HOMO\text{-}LUMO}}$ (eV)	2.67	2.65	2.71	2.54	2.60
E_{S1} (eV)	2.34	2.30	2.35	2.09	2.04
E_{S1} (eV)	0.66	0.70	0.86	1.03	1.24
$\Delta E_{\mathrm{S1\text{-}T1}}$ (eV)	1.68	1.60	1.49	1.06	0.80

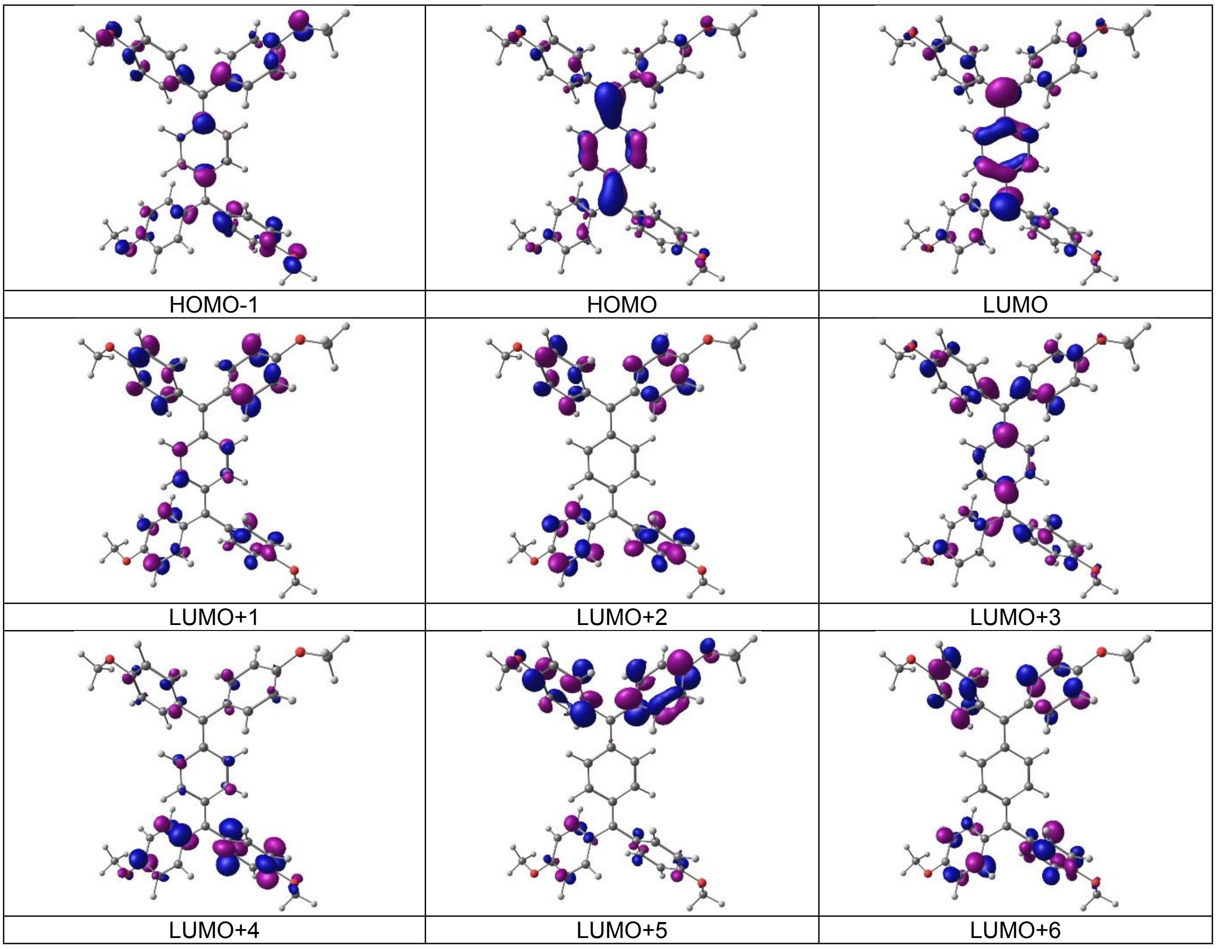

Figure S83. Frontier Molecular Orbitals of $\mathbf{2^{Ph}}$. Isosurface value = 0.045.

Table S10. Selected experimental UV/vis data of $\mathbf{2^{Ph}}$ and their corresponding TD-DFT calculations.

State	λ_{DFT} (λ_{exp}) / nm	f	Main contributing excitation (%)
**1**	484 (450)	2.27	HOMO → LUMO (94)
**9**	309 (297)	0.37	HOMO → LUMO+5 (49)
			HOMO → LUMO+6 (18)
**10**	306 (297)	0.33	HOMO → LUMO+5 (13)
			HOMO → LUMO+6 (73)

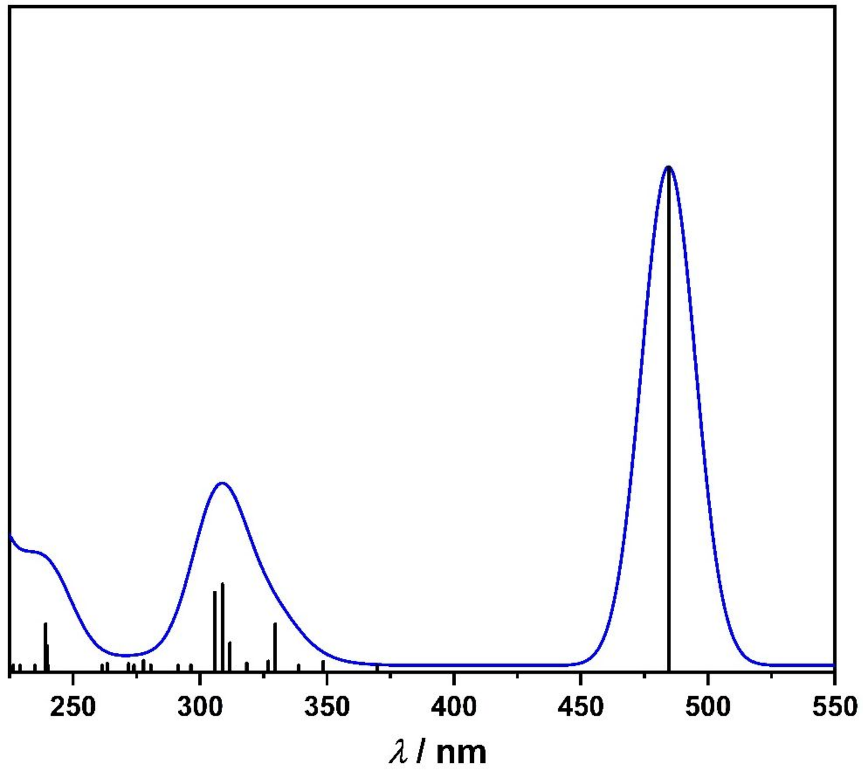

Figure S84. Calculated TD-DFT spectrum with discrete transitions of $\mathbf{2^{Ph}}$.

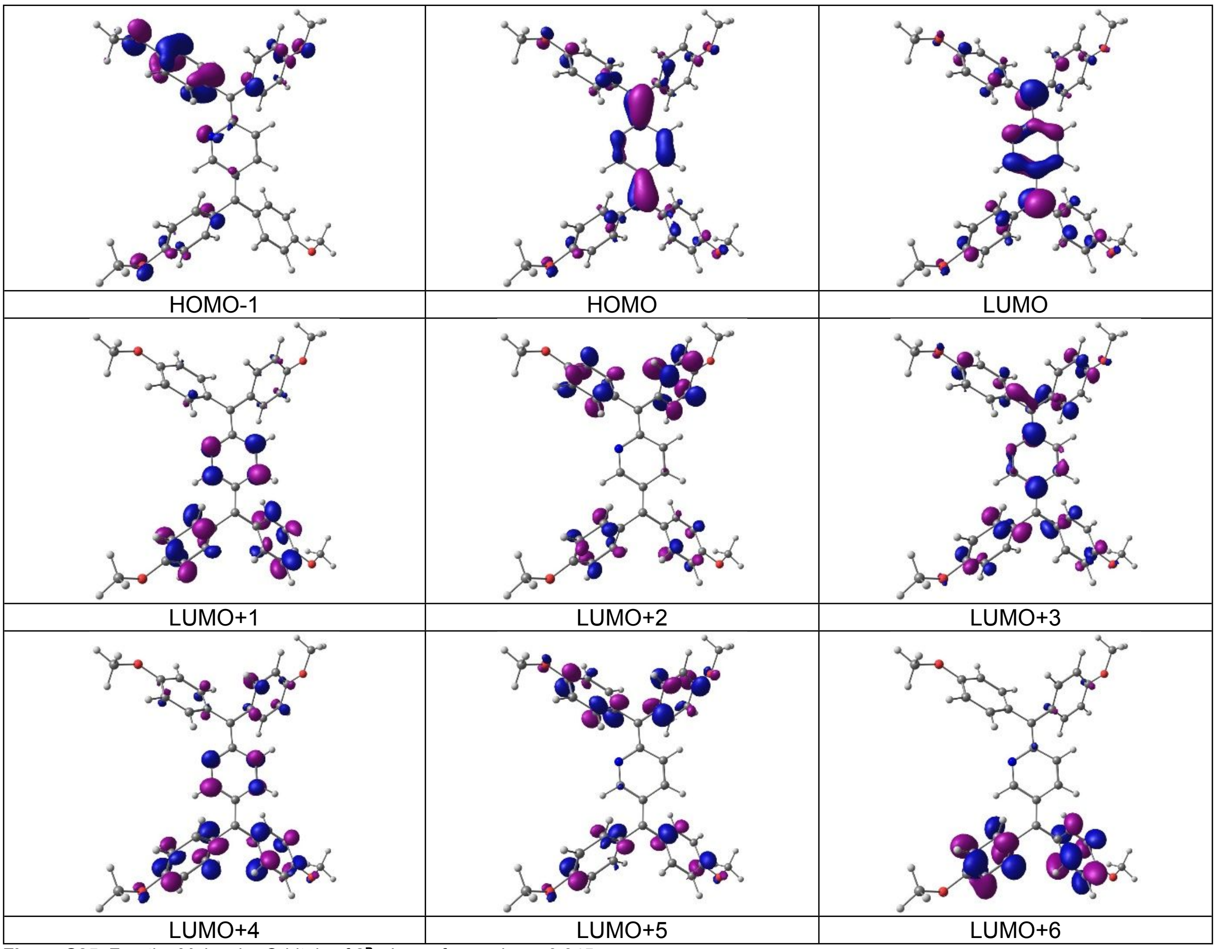

Figure S85. Frontier Molecular Orbitals of $\mathbf{2^{Py}}$. Isosurface value = 0.045.

Table S11. Selected experimental UV/vis data of **2**[Py] and their corresponding TD-DFT calculations.

State	λ_{DFT} (λ_{exp}) / nm	f	Main contributing excitation (%)
**1**	494 (469)	2.15	HOMO → LUMO (94)
**10**	301 (297)	0.61	HOMO → LUMO+5 (89)

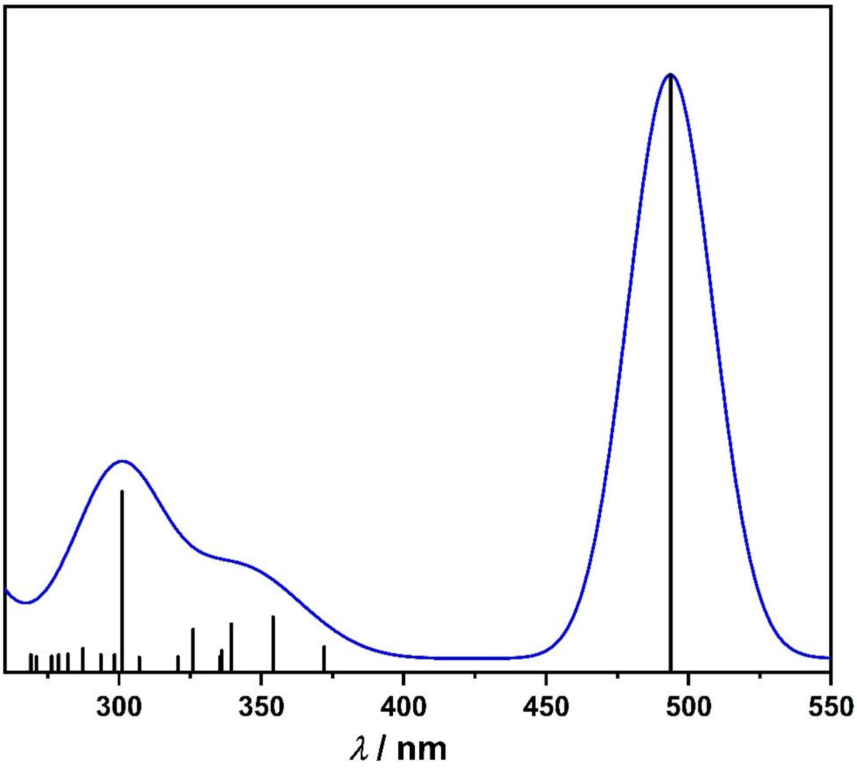

Figure S86. Calculated TD-DFT spectrum with discrete transitions of **2**[Py].

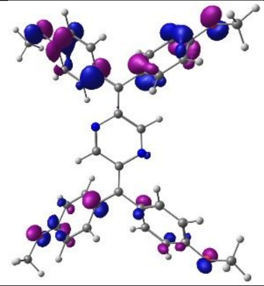	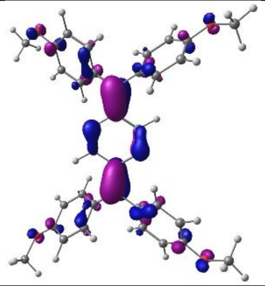	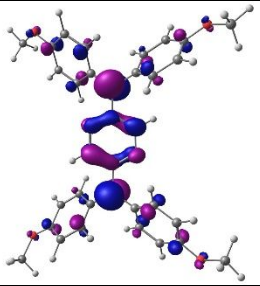
HOMO-1	HOMO	LUMO
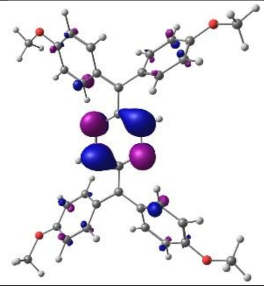	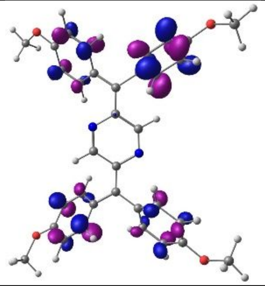	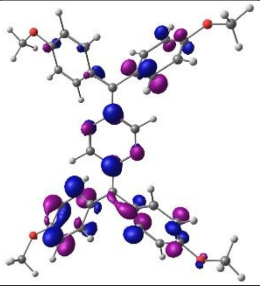
LUMO+1	LUMO+2	LUMO+3

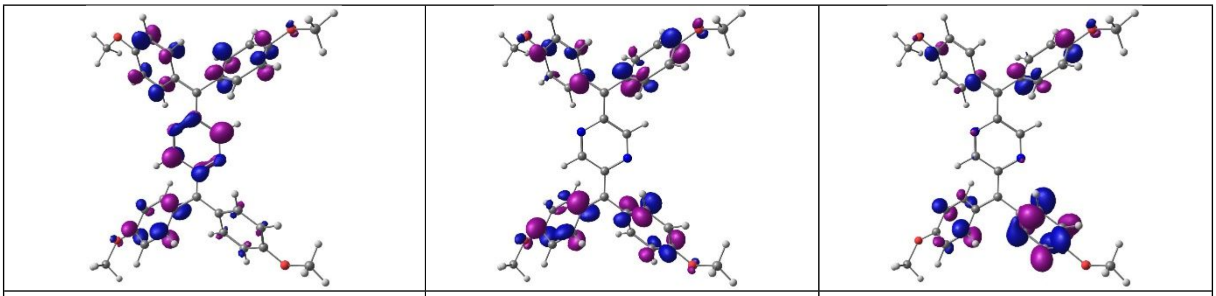

LUMO+4	LUMO+5	LUMO+6

Figure S87. Frontier Molecular Orbitals of **2**^{Pz}. Isosurface value = 0.045.

Table S12. Selected experimental UV/vis data of **2**^{Pz} and their corresponding TD-DFT calculations.

State	λ_{DFT} (λ_{exp}) / nm	f	Main contributing excitation (%)
**1**	488 (475)	2.17	HOMO → LUMO (95)
**11**	290 (295)	0.43	HOMO → LUMO+5 (82)

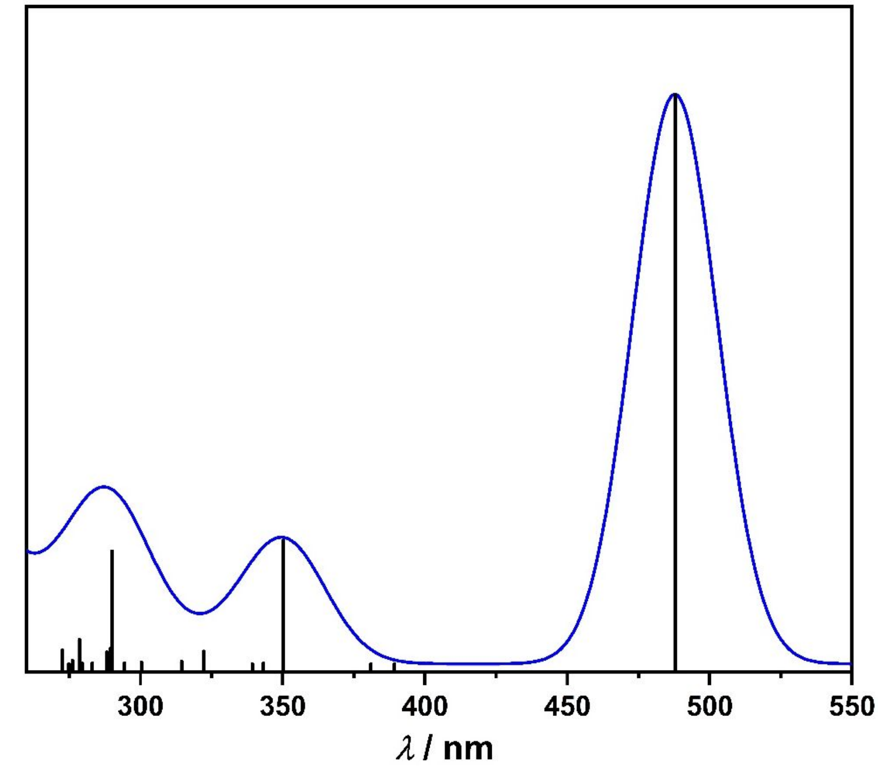

Figure S88. Calculated TD-DFT spectrum with discrete transitions of **2**^{Pz}.

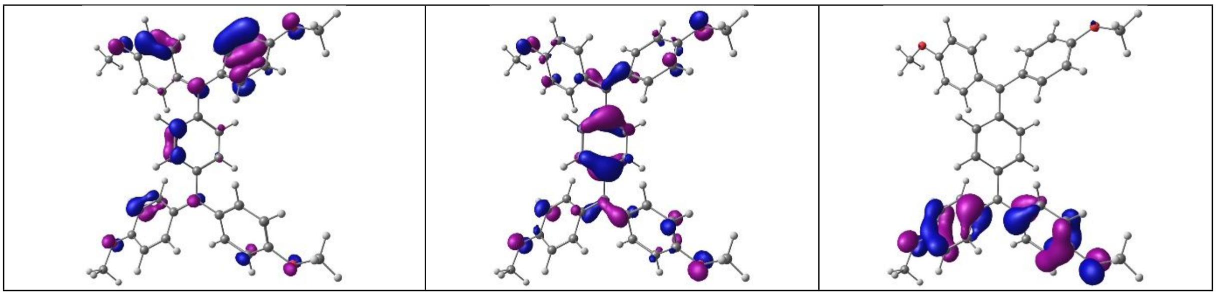

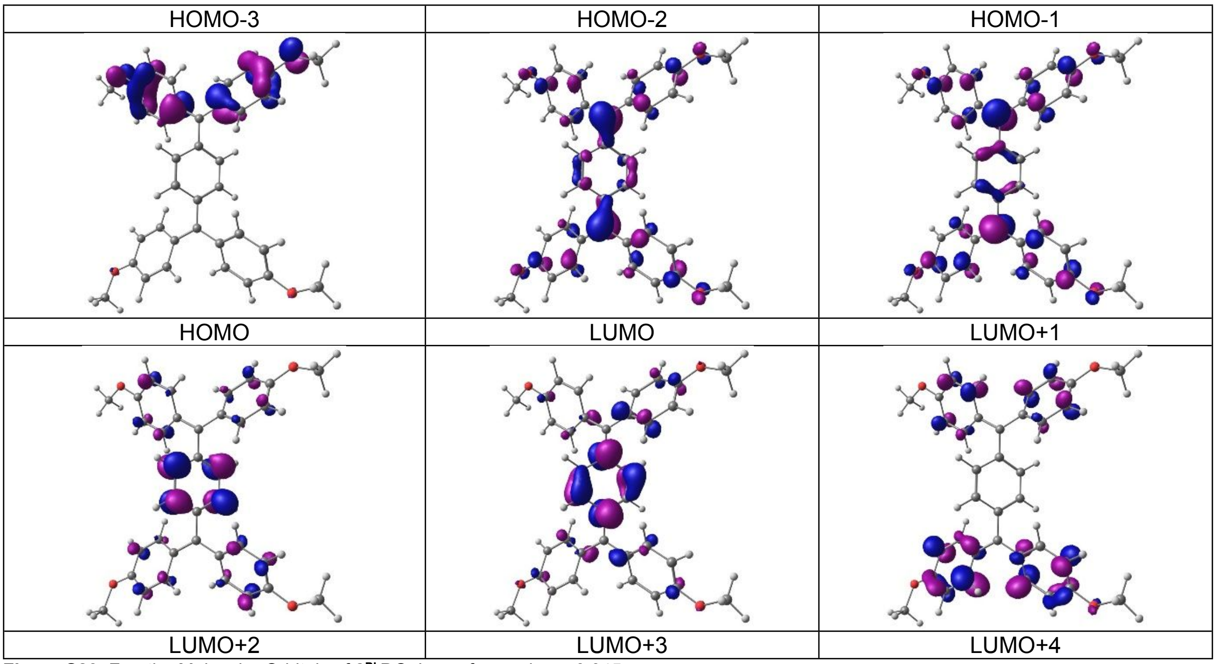

Figure S89. Frontier Molecular Orbitals of **2^{Ph}DC**. Isosurface value = 0.045.

Table S13. Selected experimental UV/vis data of **2^{Ph}DC** and their corresponding TD-DFT calculations.

State	λ_{DFT} (λ_{exp}) / nm	f	Main contributing excitation (%)
**2**	482 (536)	0.80	HOMO−1 → LUMO (84)
			HOMO → LUMO (13)
**3**	426 (512)	1.09	HOMO−2 → LUMO (97)
**5**	384 (454)	1.46	HOMO−1 → LUMO+1 (38)
			HOMO → LUMO+1 (57)

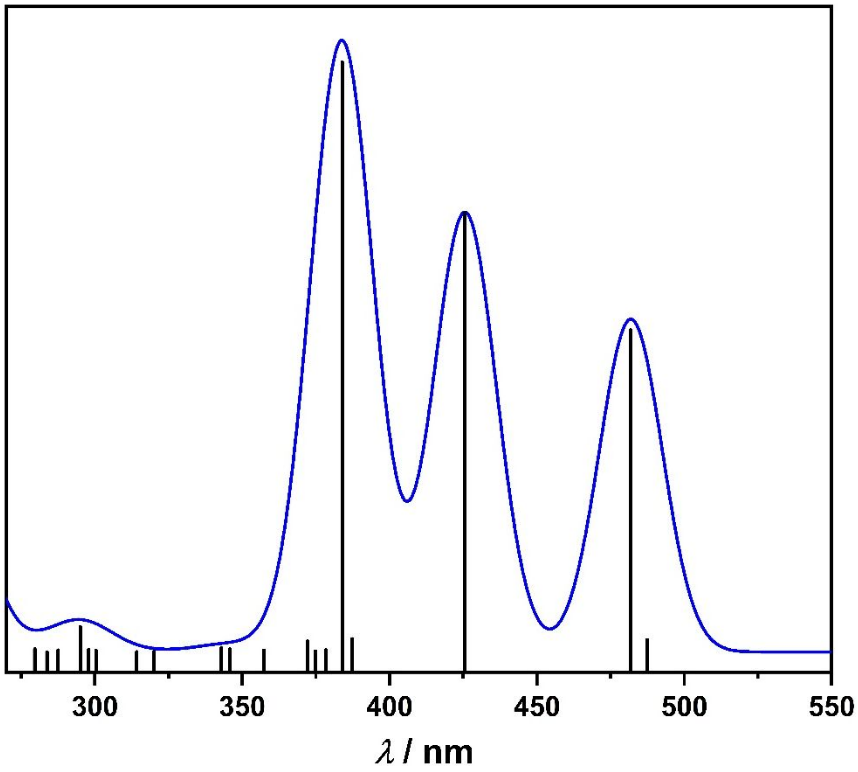

Figure S90. Calculated TD-DFT spectrum with discrete transitions of $\mathbf{2^{Ph}DC}$.

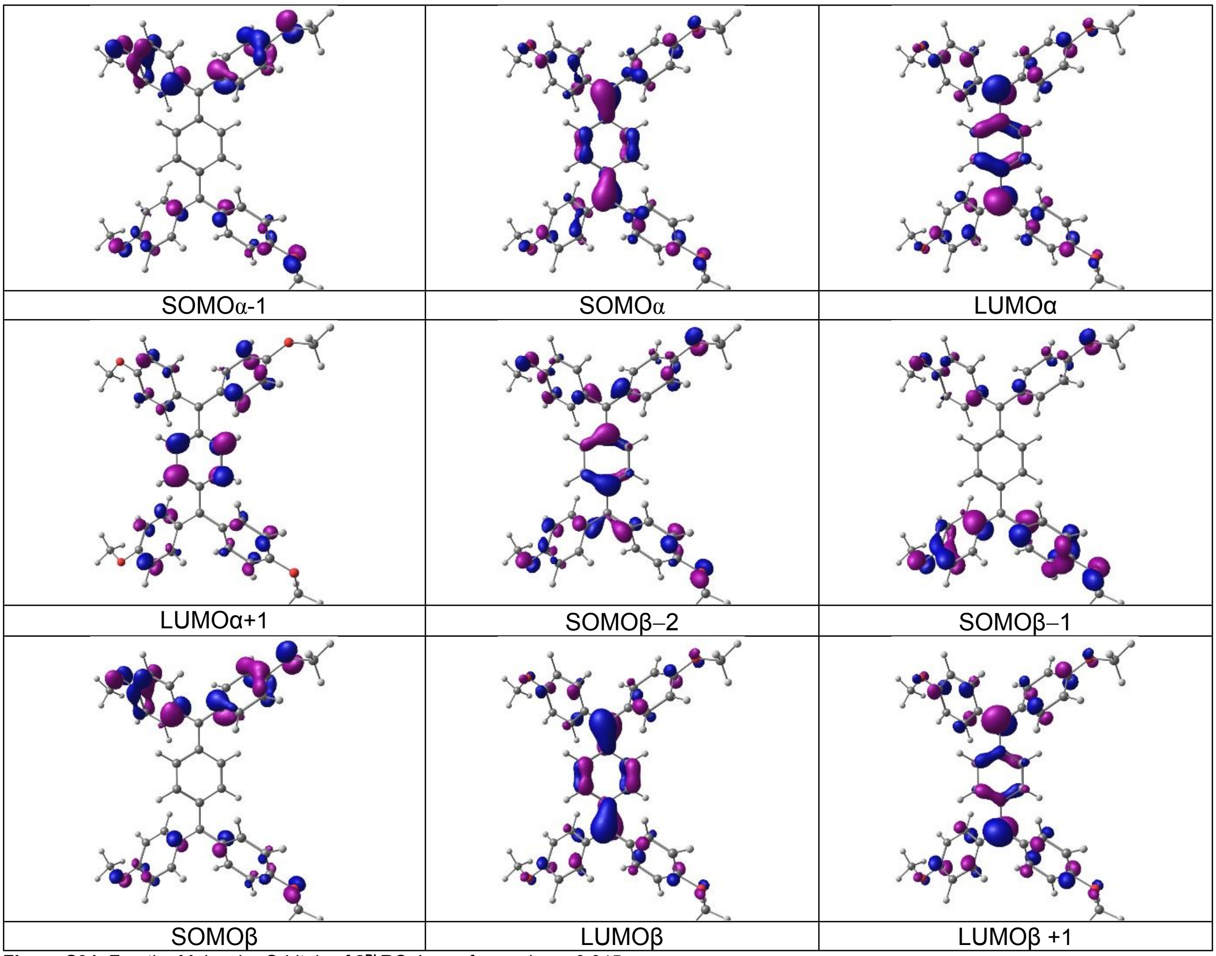

Figure S91. Frontier Molecular Orbitals of $\mathbf{2^{Ph}RC}$. Isosurface value = 0.045.

Table S14. Selected experimental UV/vis data of **2^{Ph}RC** and their corresponding TD-DFT calculations.

State	λ_{DFT} (λ_{exp}) / nm	f	Main contributing excitation (%)
**1**	482 (1130)	0.68	SOMOα → LUMOα (97)
**4**	505 (620)	0.73	SOMOβ−2 → LUMOβ (93)
**6**	418 (418)	0.40	SOMOα−1 → LUMOα (70)

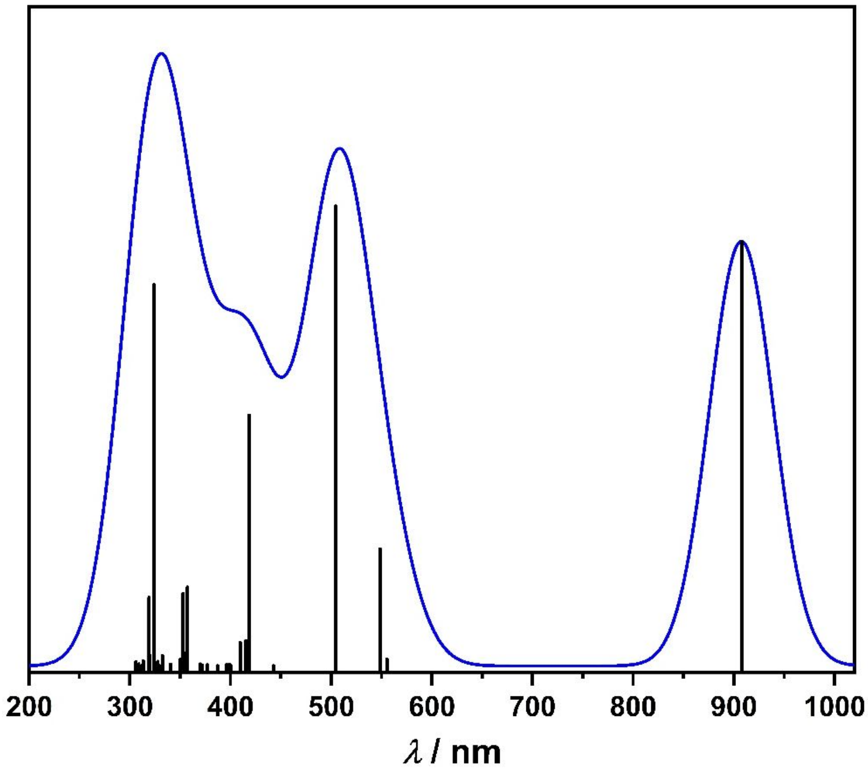

Figure S92. Calculated TD-DFT spectrum with discrete transitions of **2^{Ph}RC**.

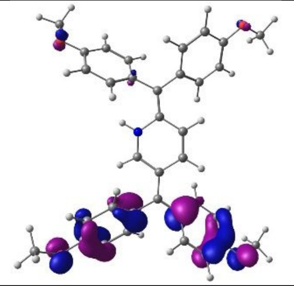	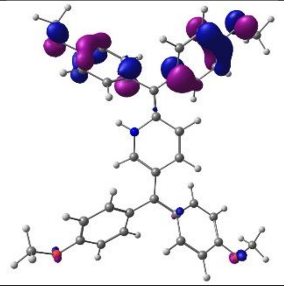	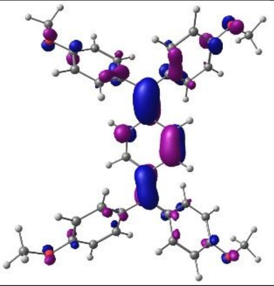
HOMO-2	HOMO-1	HOMO
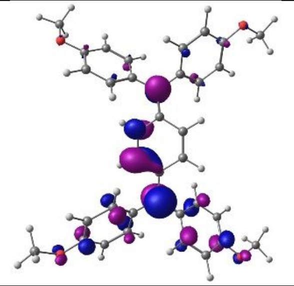	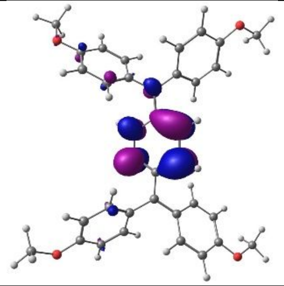	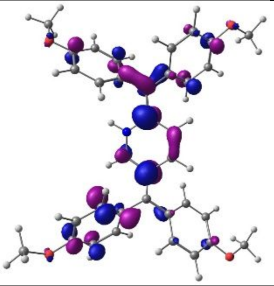
LUMO	LUMO+1	LUMO+2

Figure S93. Frontier Molecular Orbitals of 3^{Py}. Isosurface value = 0.045.

Table S15. Selected experimental UV/vis data of 3^{Py} and their corresponding TD-DFT calculations.

State	λ_{DFT} (λ_{exp}) / nm	f	Main contributing excitation (%)		
1	587 (603)	1.36	HOMO	→	LUMO (92)
2	418 (395)	0.63	HOMO	→	LUMO+1 (90)
3	392 (395)	0.58	HOMO–1	→	LUMO (75)

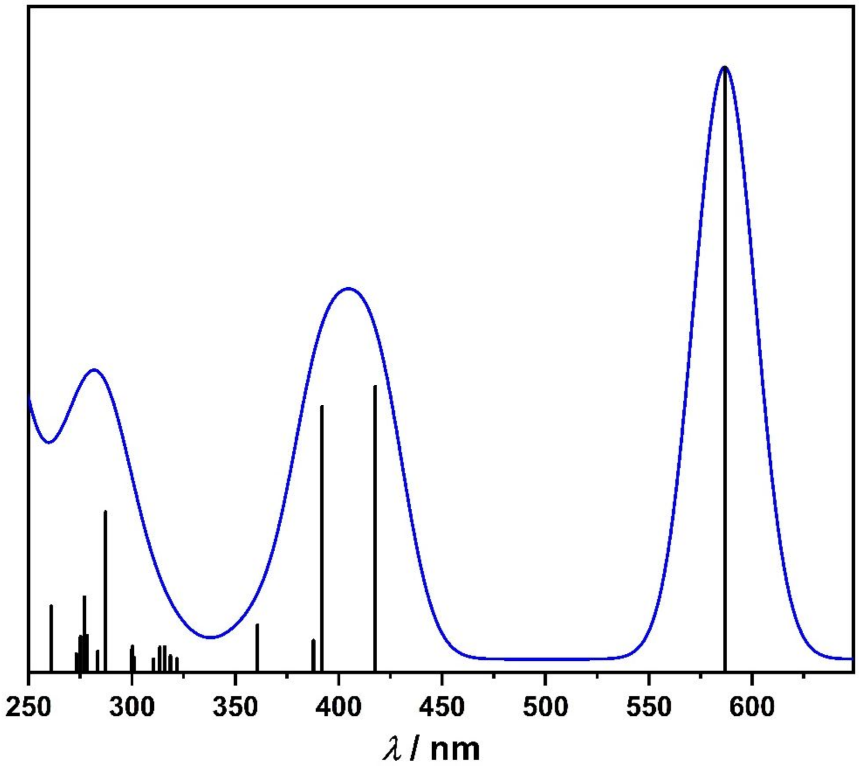

Figure S94. Calculated TD-DFT spectrum with discrete transitions of 3^{Py}.

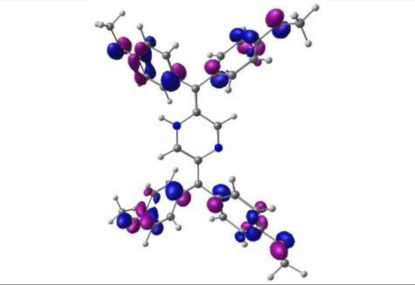	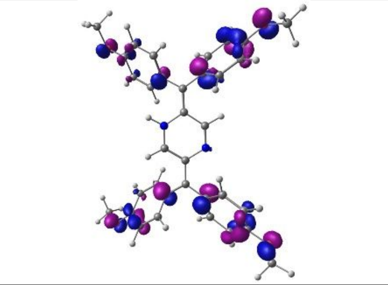	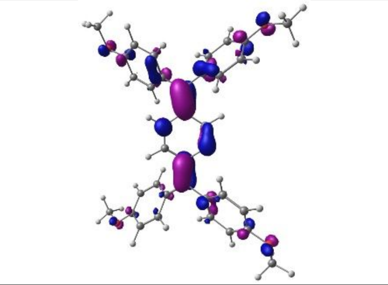
HOMO-2	HOMO-1	HOMO

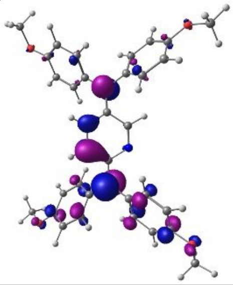	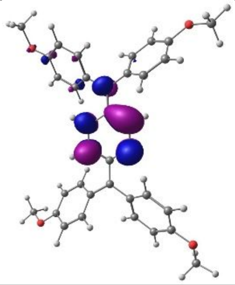	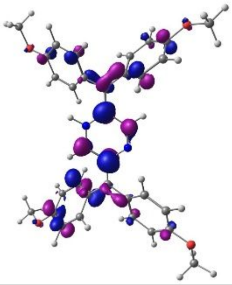
LUMO	LUMO+1	LUMO+2

Figure S95. Frontier Molecular Orbitals of **3^{Pz}**. Isosurface value = 0.045.

Table S16. Selected experimental UV/vis data of **3^{Pz}** and their corresponding TD-DFT calculations.

State	λ_{DFT} (λ_{exp}) / nm	f	Main contributing excitation (%)
**1**	553 (574)	1.44	HOMO → LUMO (91)
**2**	444 (404)	0.58	HOMO → LUMO+1 (89)
**3**	397 (404)	0.63	HOMO−1 → LUMO (83)

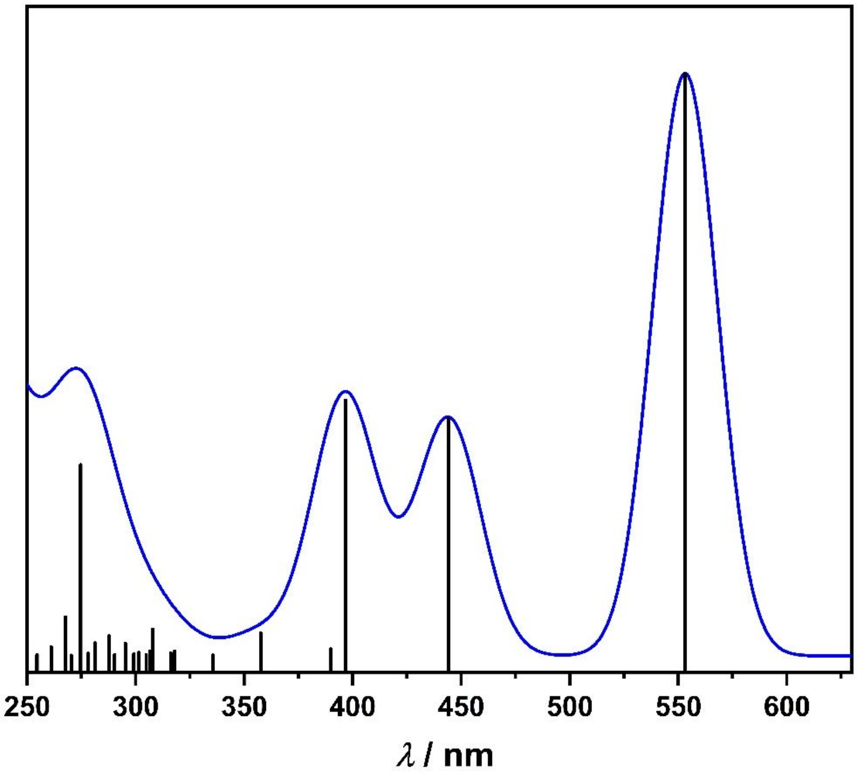

Figure S96. Calculated TD-DFT spectrum with discrete transitions of **3^{Pz}**.

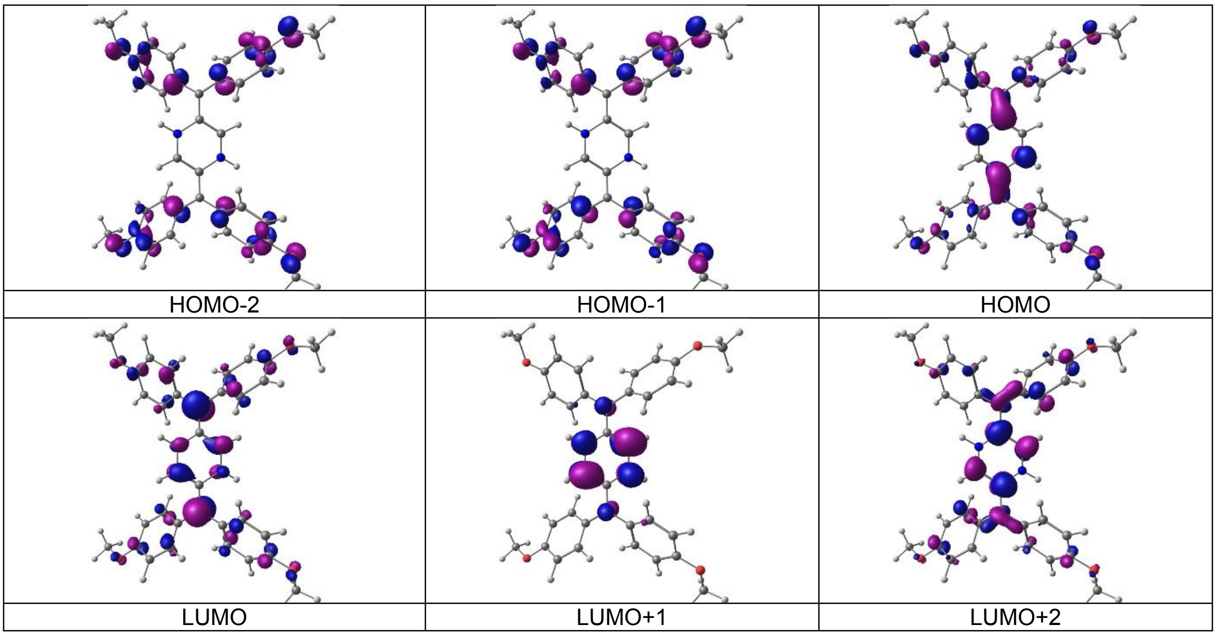

Figure S97. Frontier Molecular Orbitals of **4^{Pz}**. Isosurface value = 0.045.

Table S17. Selected experimental UV/vis data of **4^{Pz}** and their corresponding TD-DFT calculations.

State	λ_{DFT} (λ_{exp}) / nm	f	Main contributing excitation (%)
**2**	549 (620)	1.87	HOMO → LUMO (97)
**3**	421 (441)	0.92	HOMO–1 → LUMO (96)

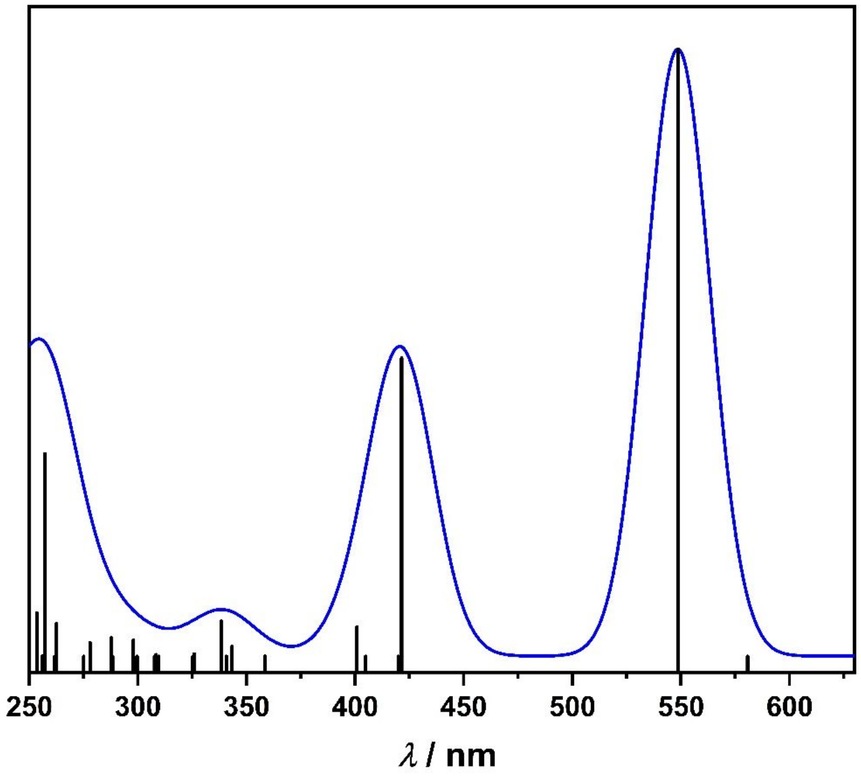

Figure S98. Calculated TD-DFT spectrum with discrete transitions of **4^{Pz}**.

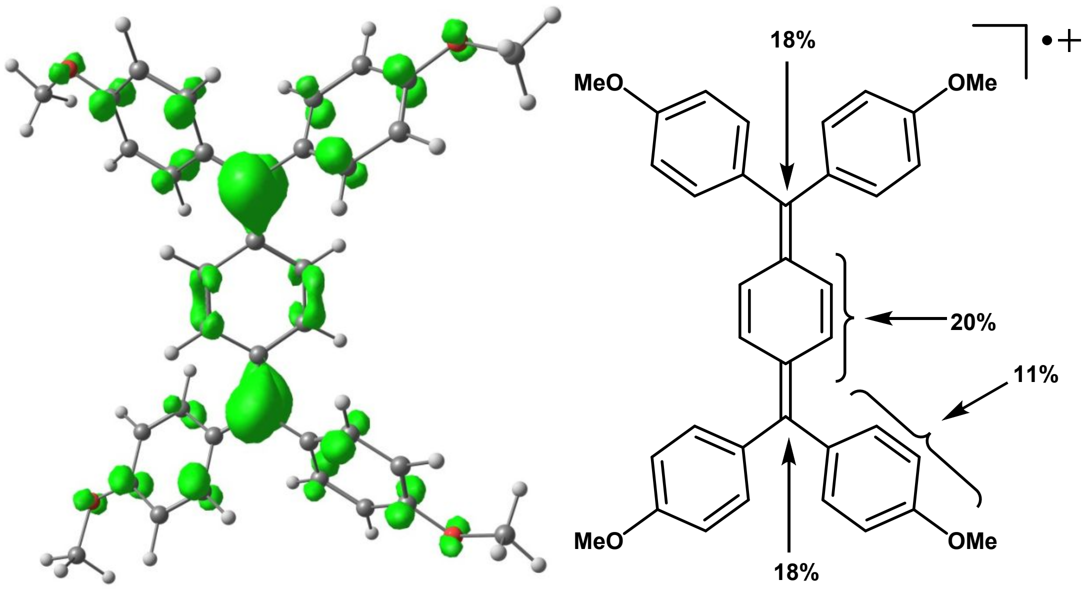

Figure S99. Spin Density Plot of **2^Ph^RC**. Isosurface value = 0.0045 (left). Calculated Löwdin spin densities (right).

Table S18. Summary of state configurations and energies (NEVPT2) for CASSCF(2,2) calculations of the three neutral compounds and three C-C bond distances.

**2^Ph**	C-C distance (Å)	**1.380**	C-C distance (Å)	**1.464**	C-C distance (Å)	**1.544**
State	Composition	Energy	Composition	Energy	Composition	Energy
S0	0.95[02]+0.05[20]	0	0.90[02]+0.10[20]	0	0.77[02]+0.22[20]	0
T0	[11]	11528.5	[11]	7841.2	[11]	3523.8
S1	[11]	12532.7	[11]	8113.2	[11]	3507.5
S2	0.05[02]+0.95[20]	12563.2	0.10[02]+0.90[20]	8039.2	0.22[20]+0.77[02]	3567.3
**2^Pz**	C-C distance (Å)	**1.378**	C-C distance (Å)	**1.460**	C-C distance (Å)	**1.530**
State	Composition	Energy	Composition	Energy	Composition	Energy
S0	0.96[02]+0.04[20]	0	0.92[20]+0.08[02]	0	0.84[02]+0.16[20]	0
T0	[11]	12094.9	[11]	8639.3	[11]	5259.2
S1	[11]	13158.2	[11]	9081.0	[11]	4313.2
S2	0.04[02]+0.96[20]	13626.3	0.08[20]+0.92[02]	9373.7	0.16 [02]+0.84[20]	5531.2
**2^Py**	C-C distance (Å)	**1.379**	C-C distance (Å)	**1.462**	C-C distance (Å)	**1.560**
State	Composition	Energy	Composition	Energy	Composition	Energy
S0	0.94[02]+0.05[20]+0.01 [11]	0	0.78[11]+0.18[20]+0.04 [02]	0	0.74[02]+0.22[20]+0.04 [11]	0
T0	[11]	11647.6	[11]	7999.3	[11]	3351.7
S1	0.98[11]+0.02[02]	12774.5	0.34[20]+0.65[02]	8581.5	0.95[11]+0.05[02]	3853.6
S2	0.95[20]+0.05[02]	13961.1	0.21[11]+0.48[20]+0.30 [02]	9276.8	0.78[20]+0.20[02]+0.01 [11]	4218.3

Table S19. Analysis of the size of the active space on state configurations and energies for **2^Py** with three C-C distances.

**2^Py**									
Distance (Å)	**1.379**	aab		aal		aaf2			
		(2,2)	Energy (cm⁻¹)	(4,4)	Energy (cm⁻¹)	(6,6)	Energy (cm⁻¹)		
S0		0.94[02]+ 0.05[20]	0	0.94[2200]+ 0.05[2020]	0	0.89[222000]+ 0.06[220200]	0		
T0		[11]	11647.6	[2110]	11769.4	0.91[221100]	12244.9		
S1		0.98[11]	12774.5	0.83[2020]+ 0.07[2101]	18904.1	0.83[221100]	20329.8		
S2		0.95[20]+ 0.05[02]	13961.1	0.54[21100] + 0.43[2101]	21297.7				
Distance (Å)	**1.46**	aac		aam		aah3			
		(2,2)	Energy (cm⁻¹)	(4,4)	Energy (cm⁻¹)	(6,6)	Energy (cm⁻¹)		
S0		0.78[11]+ 0.18[20]+	0	0.89[2200]+ 0.10[2020]	0	0.78[222000]+ 0.13[220200]+	0		

		0.04[02]				0.05[221100]			
T0		[11]	7999.3	[2110]	8023.3	[221100]	8359.5		
S1		0.34[20]+ 0.65[02]	8581.5	0.77[2020]+ 0.09[2200]+ 0.07[2101]	13556.1	0.32[221100]+ 0.22[212100]+ 0.21[220200]	19289.4 (2)		
S2		0.21[11]+ 0.48[20]+ 0.30[02]	9276.8	0.61[2110]+ 0.37[2101]	15843.7	0.41[221100]+ 0.30[220200]+ 0.09[122100]	19292.6 (3)		
Distance (Å)	**1.56**	aad		aan2		aaj		aaq	
		(2,2)	Energy (cm^{-1})	(4,4)	Energy (cm^{-1})	(6,6)	Energy (cm^{-1})	(8,8)	Energy (cm^{-1})
S0		0.74[02]+ 0.22[20]+ 0.04[11]	0			0.45[222000]+ 0.43[221100]+ 0.09[220200]	0	0.67[22220000]	0
T0		[11]	3351.7			[221100]	3076.4	[22211000]	3114.8
S1		0.95[11]+ 0.05[02]	3853.6			0.34[222000]+ 0.24[221100]+ 0.14[221010]	17726.4	0.70[22211000]+ 0.11[22201100]	14894.3 (2)
S2		0.78[20]+ 0.20[02]+ 0.01[11]	4218.3			0.41[221010]+ 0.16[220200]+ 0.13[221100]	21391.3	0.26[22202000]+ 0.24[22210100]+ 0.13[22220000]	18655.2 (1)

Table S20. Diradical character calculations for the studied compounds. For the mono- and dicationic pyrazine substituents (**3^{Pz}** and **4^{Pz}**), calculations were performed on the optimized triplet and singlet geometries, while for the neutral compounds three C-C bond distances were explored.

	Code	Spin from Optimized Geometry	C-C Distances (Å)	N [139]	N [140]	y_0
**4^{Pz}**	dac	0	1.3905, 1.3905	2	0	0
	dad	1	1.4373, 1.4332	2	0	0
**3^{Pz}**	eaa	0	1.3962, 1.3684 (NH)	2	0	0
	eab	1	1.4550, 1.4255 (NH)	1.98696	0.01304	$8.6e^{-5}$
**2^{Pz}**	cca	0 (BS)	1.564	1.53655	0.46345	0.16677
		0 (BS)	1.4605	1.8632	0.1368	0.01072
		0 (BS)	1.380	2	0	0
**2^{Py}**	aba	0 (BS)	1.562	1.43693	0.56307	0.26622
		0 (BS)	1.460	1.77263	0.22737	0.03237
		0 (BS)	1.380	2	0	0
**2^{Ph}**	bba	0 (BS)	1.544	1.41547	0.58453	0.29138
		0 (BS)	1.464	1.68809	0.31191	0.06603
		0 (BS)	1.380	2	0	0

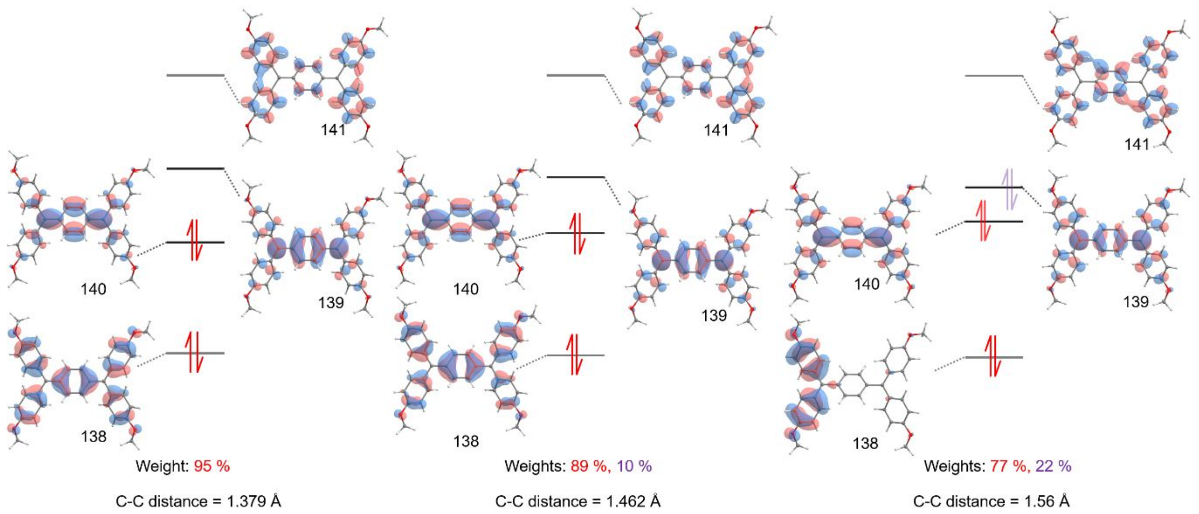

Figure S100. Frontier orbitals for **2^{Ph}** obtained from CASSCF(2,2) calculations for three bond C-C bond distances. Black lines indicate the orbitals in the active space and grey lines indicate the adjacent orbitals.

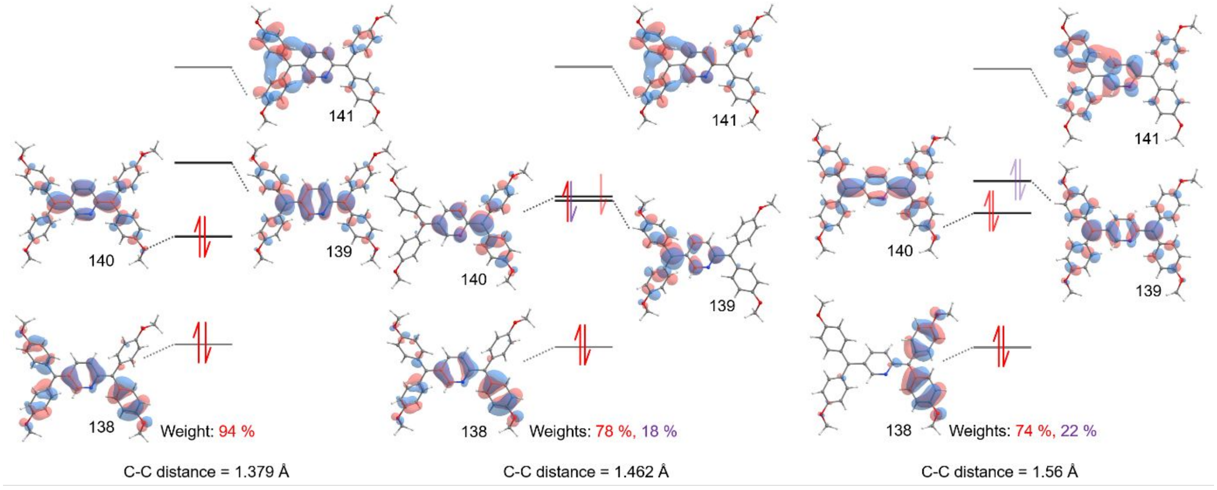

Figure S101. Frontier orbitals for **2**^{Py} obtained from CASSCF(2,2) calculations for three bond C-C bond distances. Black lines indicate the orbitals in the active space and grey lines indicate the adjacent orbitals.

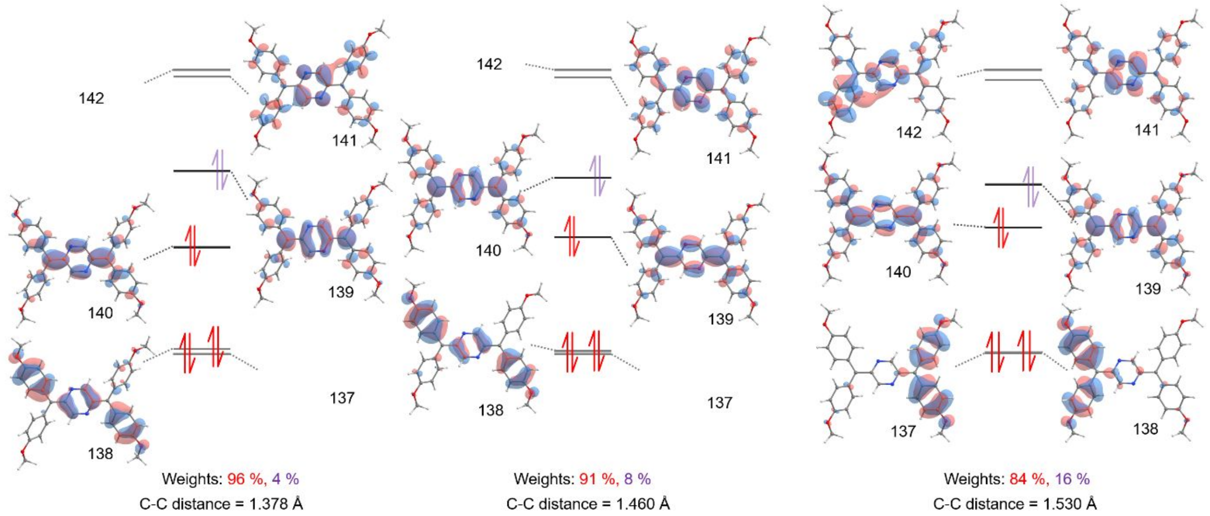

Figure S102. Frontier orbitals for **2**^{Pz} obtained from CASSCF(2,2) calculations for three bond C-C bond distances. Black lines indicate the orbitals in the active space and grey lines indicate the adjacent orbitals.

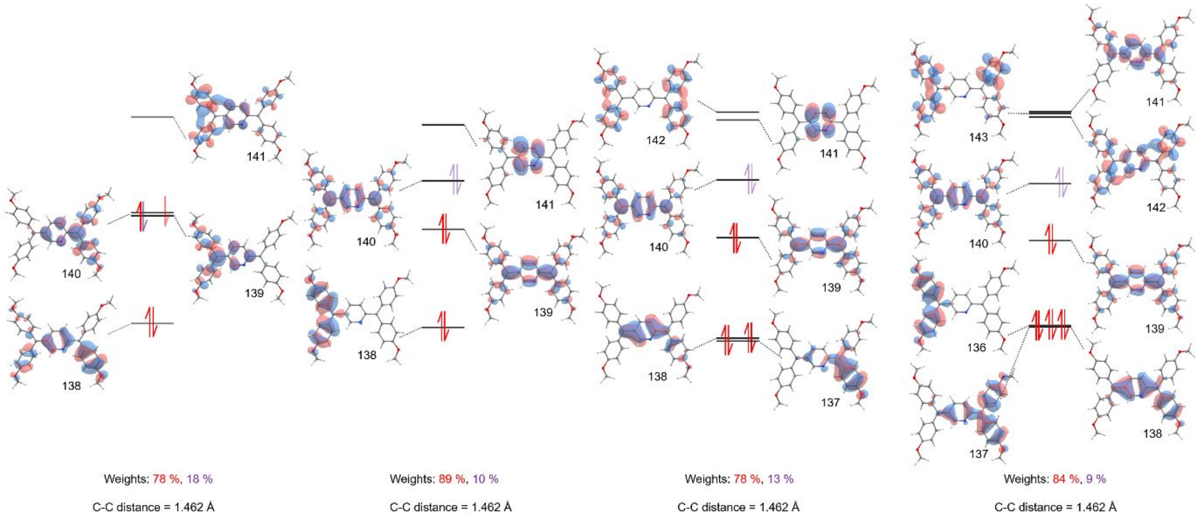

Figure S103. Frontier orbitals and energies for **2**^{Py} at a 1.462 Å C-C bond distance, using different active spaces (CAS(2,2), CAS(4,4), CAS(6,6) and CAS(8,8). Grey lines indicate orbitals not included in the active space.

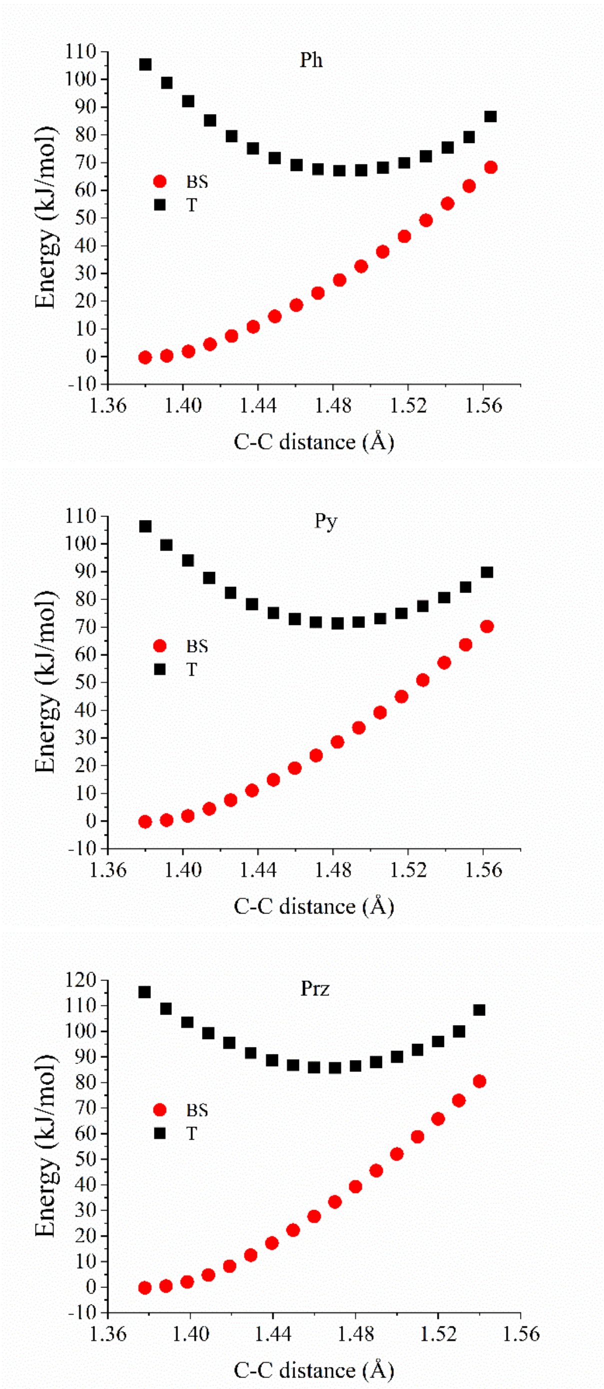

Figure S104. Relative broken symmetry singlet (BS) and triplet (T) energies for $\mathbf{2^{Ph}}$ (top), $\mathbf{2^{Py}}$ (middle) and $\mathbf{2^{Pz}}$ (bottom), as a function of C-C bond distance.

Table S21. Optimised molecular structure of $\mathbf{2^{Ph}}$ (CSS) in XYZ-coordinates.

Atom	x	y	z
C	-1.357233	0.363467	-1.941649
C	-1.104627	1.61983	-1.277721
C	-1.00998	0.352577	0.817414
C	-1.262349	-0.903776	0.15343
H	-1.559536	0.375494	-3.00561
H	-0.806859	0.340975	1.881259
C	-1.337558	-2.101938	0.833781
C	-1.028552	2.817949	-1.958156
C	-1.096744	4.111368	-1.265949

C	-0.246195	5.165138	-1.631318
C	-2.024698	4.361908	-0.254709
C	-0.299795	6.387874	-0.99816
C	-2.099662	5.589787	0.386551
C	-1.22867	6.612069	0.020175
H	0.479621	5.009991	-2.421607
H	-2.725045	3.583444	0.025171
H	0.373072	7.190228	-1.278529
H	-2.844418	5.741257	1.156366
C	-0.87354	2.872255	-3.41785
C	0.024164	2.048284	-4.097049
C	-1.602724	3.794498	-4.182399
C	0.183958	2.112541	-5.473656
C	-1.464831	3.86465	-5.551738
C	-0.568981	3.021215	-6.212149
H	0.633144	1.351611	-3.532537
H	-2.300917	4.458973	-3.68597
H	0.902199	1.462361	-5.95493
H	-2.045354	4.570551	-6.134656
C	-1.493397	-2.157153	2.293405
C	-2.392089	-1.32506	2.97502
C	-0.775229	-3.080582	3.055084
C	-2.548458	-1.399998	4.343167
C	-0.911547	-3.162411	4.432113
C	-1.803688	-2.316275	5.086869
H	-2.995838	-0.623298	2.411376
H	-0.079815	-3.748002	2.558565
H	-3.257095	-0.76117	4.857804
H	-0.322197	-3.8839	4.981907
C	-1.267842	-3.39454	0.139913
C	-0.336336	-3.643401	-0.868361
C	-2.120947	-4.448342	0.499178
C	-0.259651	-4.869952	-1.512159
C	-2.065901	-5.669559	-0.136727
C	-1.132924	-5.892291	-1.151682
H	0.365286	-2.864534	-1.143952
H	-2.84985	-4.294397	1.286818
H	0.488057	-5.02023	-2.279345
H	-2.740916	-6.471825	0.138708
O	-2.018318	-2.319678	6.419722
C	-1.287233	-3.241771	7.204824
H	-1.505664	-4.27262	6.910637
H	-0.210794	-3.062899	7.125751
H	-1.606271	-3.082861	8.232902
O	-1.144027	-7.119307	-1.714568
C	-0.211556	-7.38073	-2.745673
H	-0.35993	-6.705502	-3.593569
H	0.816348	-7.286117	-2.383083
H	-0.390891	-8.406064	-3.062703
O	-1.216339	7.840293	0.580236
C	-2.145257	8.103536	1.614138
H	-3.174389	8.007173	1.255542
H	-1.993196	7.430567	2.463168
H	-1.965582	9.129811	1.927906
O	-0.493354	3.162479	-7.552604
C	0.402374	2.323198	-8.255551
H	1.434975	2.489096	-7.934348
H	0.145861	1.268423	-8.119102
H	0.303007	2.587664	-9.306341
C	-0.937102	1.525061	0.152634
C	-1.430194	-0.808936	-1.276856
H	-0.677736	2.422377	0.700946
H	-1.689526	-1.706344	-1.825066

Table S22. Optimised molecular structure of **2**^{Ph} (OST) in XYZ-coordinates.

Atom	x	y	z
C	-1.7858	0.507453	-1.79784
C	-1.10252	1.612741	-1.27418
C	-0.55551	0.256564	0.66244
C	-1.23741	-0.84989	0.138263
H	-2.28769	0.601174	-2.7542
H	-0.05184	0.163144	1.617831
C	-1.30514	-2.11811	0.863667

C	-1.02792	2.8795	-2.00452
C	-1.16626	4.122717	-1.27992
C	-0.5216	5.306238	-1.6953
C	-1.93898	4.207295	-0.11036
C	-0.64619	6.483385	-0.9938
C	-2.07851	5.38713	0.599935
C	-1.43056	6.540297	0.161181
H	0.112468	5.283937	-2.57338
H	-2.46027	3.325138	0.242043
H	-0.12896	7.379375	-1.31805
H	-2.69808	5.399873	1.486827
C	-0.81016	2.859935	-3.43325
C	-0.09589	1.820824	-4.05183
C	-1.31247	3.869259	-4.28069
C	0.121565	1.784399	-5.4183
C	-1.10921	3.840611	-5.64132
C	-0.38483	2.799429	-6.2277
H	0.317719	1.028052	-3.43992
H	-1.8983	4.674991	-3.85501
H	0.691767	0.967983	-5.84119
H	-1.51752	4.616361	-6.27924
C	-1.47766	-2.10682	2.299497
C	-2.17985	-1.0698	2.947832
C	-0.9492	-3.11501	3.12118
C	-2.34974	-1.05249	4.313016
C	-1.10673	-3.10531	4.496746
C	-1.81427	-2.07113	5.10594
H	-2.61573	-0.27507	2.354196
H	-0.37549	-3.91647	2.671368
H	-2.90682	-0.25558	4.792603
H	-0.66543	-3.89918	5.084633
C	-1.19504	-3.36087	0.132164
C	-0.44719	-3.45187	-1.05268
C	-1.84379	-4.53793	0.558915
C	-0.33659	-4.63152	-1.76857
C	-1.7482	-5.71468	-0.14775
C	-0.98954	-5.77775	-1.31954
H	0.078033	-2.57542	-1.41333
H	-2.45725	-4.51016	1.451378
H	0.264779	-4.64953	-2.66782
H	-2.26883	-6.6054	0.185466
O	-2.02741	-1.96991	6.435407
C	-1.50277	-2.9862	7.267562
H	-1.92205	-3.96356	7.010715
H	-0.41177	-3.03077	7.198181
H	-1.79055	-2.72383	8.283617
O	-0.95121	-6.97428	-1.94408
C	-0.19965	-7.07196	-3.13826
H	-0.58602	-6.39444	-3.90553
H	0.857106	-6.85177	-2.96009
H	-0.30302	-8.10065	-3.47758
O	-1.4968	7.737973	0.78126
C	-2.27819	7.83064	1.956575
H	-3.32753	7.596706	1.753705
H	-1.90227	7.160787	2.735667
H	-2.19574	8.86176	2.294253
O	-0.23165	2.856622	-7.56808
C	0.488271	1.812953	-8.19505
H	1.518786	1.768048	-7.83024
H	0.00432	0.845289	-8.03254
H	0.4904	2.043216	-9.25854
C	-0.4908	1.454055	-0.02403
C	-1.85182	-0.68954	-1.11115
H	0.061607	2.283414	0.402909
H	-2.40591	-1.51786	-1.53771

Table S23. Optimised molecular structure of **2^{Py}** (CSS) in XYZ-coordinates.

Atom	x	y	z
C	-1.31907	0.380192	-1.76289
C	-1.07923	1.662997	-1.13952
C	-1.00933	0.413294	0.933994
C	-1.25579	-0.81463	0.221861
N	-1.40888	-0.75252	-1.15683

H	-1.50288	0.365622	-2.8348
H	-0.82018	0.37004	1.998754
C	-1.3303	-2.0413	0.848847
C	-1.01779	2.829556	-1.8734
C	-1.12915	4.146219	-1.23744
C	-0.31666	5.212379	-1.65063
C	-2.05985	4.40254	-0.22963
C	-0.41048	6.456359	-1.06644
C	-2.17683	5.651774	0.359587
C	-1.34352	6.688643	-0.05349
H	0.411588	5.050158	-2.43701
H	-2.73105	3.612178	0.086001
H	0.232828	7.269835	-1.38158
H	-2.92348	5.808675	1.126351
C	-0.82261	2.81667	-3.32725
C	0.108223	1.975893	-3.93836
C	-1.54951	3.684639	-4.15547
C	0.300731	1.970351	-5.3118
C	-1.37926	3.683809	-5.52202
C	-0.45164	2.823296	-6.11478
H	0.719184	1.325939	-3.32228
H	-2.27331	4.358519	-3.71142
H	1.043178	1.311028	-5.74077
H	-1.95831	4.345082	-6.15626
C	-1.45633	-2.14188	2.310031
C	-2.3696	-1.36128	3.030628
C	-0.69185	-3.06046	3.030357
C	-2.49698	-1.48006	4.398934
C	-0.79848	-3.18567	4.40674
C	-1.70675	-2.39021	5.101803
H	-3.00694	-0.66458	2.498304
H	0.015654	-3.68894	2.501196
H	-3.21682	-0.88087	4.944696
H	-0.17398	-3.90121	4.924562
C	-1.29396	-3.30475	0.107219
C	-0.44671	-3.5088	-0.9832
C	-2.10003	-4.3825	0.502954
C	-0.40511	-4.71606	-1.66331
C	-2.08176	-5.58478	-0.171
C	-1.23191	-5.76324	-1.26362
H	0.211187	-2.70921	-1.29713
H	-2.76537	-4.26344	1.350433
H	0.27812	-4.83265	-2.49395
H	-2.72161	-6.40462	0.134837
O	-1.89483	-2.43849	6.437664
C	-1.11709	-3.35612	7.182248
H	-1.30622	-4.38533	6.863187
H	-0.04919	-3.13801	7.087658
H	-1.42068	-3.23678	8.220304
O	-1.27075	-6.97384	-1.86054
C	-0.42293	-7.18936	-2.97198
H	-0.64985	-6.49206	-3.78386
H	0.63001	-7.08874	-2.6923
H	-0.61345	-8.20775	-3.30466
O	-1.37281	7.936796	0.455393
C	-2.30662	8.211164	1.482939
H	-3.33276	8.063117	1.134036
H	-2.12656	7.580855	2.358641
H	-2.16182	9.255587	1.751197
O	-0.3466	2.894723	-7.45703
C	0.58023	2.036443	-8.09506
H	1.601915	2.240334	-7.76145
H	0.339892	0.986023	-7.90692
H	0.498669	2.242623	-9.16022
C	-0.93209	1.5949	0.289756
H	-0.68931	2.495339	0.840533

Table S24. Optimised molecular structure of **2**^{Py} (OST) in XYZ-coordinates.

Atom	x	y	z
C	-1.74051	0.50645	-1.65543
C	-1.08327	1.644459	-1.17162
C	-0.57163	0.319304	0.768442
C	-1.24689	-0.76561	0.193441

N	-1.82497	-0.64812	-1.01486
H	-2.23621	0.554467	-2.62166
H	-0.09246	0.204409	1.732501
C	-1.3262	-2.05514	0.877139
C	-1.02233	2.882705	-1.94558
C	-1.20197	4.144378	-1.26455
C	-0.58274	5.329976	-1.71176
C	-1.98739	4.245372	-0.10465
C	-0.74348	6.524535	-1.0488
C	-2.16233	5.442757	0.567241
C	-1.53985	6.597647	0.097376
H	0.06082	5.296071	-2.58245
H	-2.49244	3.363109	0.270702
H	-0.24577	7.422721	-1.39653
H	-2.78977	5.467527	1.448305
C	-0.77678	2.812655	-3.36764
C	-0.03189	1.763795	-3.93169
C	-1.28144	3.779133	-4.26244
C	0.21176	1.678483	-5.29118
C	-1.05167	3.701447	-5.61671
C	-0.29751	2.651997	-6.14868
H	0.386536	1.003419	-3.2827
H	-1.89109	4.588675	-3.87982
H	0.80413	0.857011	-5.67151
H	-1.4622	4.443691	-6.2919
C	-1.45691	-2.08348	2.318288
C	-2.15687	-1.07761	3.01475
C	-0.88996	-3.10672	3.093037
C	-2.28483	-1.10058	4.384434
C	-1.00513	-3.13799	4.472224
C	-1.70852	-2.1317	5.131272
H	-2.62713	-0.27581	2.457628
H	-0.3203	-3.88659	2.602048
H	-2.84045	-0.32759	4.903071
H	-0.53507	-3.94112	5.023941
C	-1.25488	-3.27613	0.108556
C	-0.54474	-3.34473	-1.10048
C	-1.89918	-4.45615	0.530981
C	-0.46413	-4.50949	-1.8423
C	-1.83541	-5.61858	-0.20304
C	-1.11364	-5.6606	-1.39833
H	-0.02738	-2.46455	-1.46061
H	-2.48378	-4.44284	1.442949
H	0.108674	-4.51323	-2.76012
H	-2.35244	-6.5125	0.126904
O	-1.88116	-2.07054	6.468006
C	-1.31709	-3.10186	7.255536
H	-1.73259	-4.07726	6.985796
H	-0.22856	-3.12976	7.149872
H	-1.57529	-2.87009	8.28679
O	-1.10385	-6.84215	-2.05045
C	-0.39694	-6.91717	-3.27347
H	-0.80545	-6.21872	-4.00979
H	0.667385	-6.70956	-3.12893
H	-0.52155	-7.93674	-3.63255
O	-1.64208	7.811705	0.677965
C	-2.43607	7.92231	1.843529
H	-3.47717	7.654804	1.639872
H	-2.04978	7.288111	2.646913
H	-2.38292	8.96566	2.147735
O	-0.12013	2.660003	-7.4866
C	0.628136	1.60511	-8.05997
H	1.652449	1.592203	-7.67584
H	0.157051	0.636562	-7.86807
H	0.64539	1.794265	-9.13135
C	-0.49213	1.51583	0.089068
H	0.048515	2.351409	0.519099

Table S25. Optimised molecular structure of **2^{Pz}** (CSS) in XYZ-coordinates.

Atom	x	y	z
C	-1.32811	0.317245	-1.88345
C	-1.09299	1.57111	-1.20866
N	-0.96318	1.531789	0.171736

C	-1.06161	0.389828	0.758025
C	-1.29272	-0.86441	0.082803
N	-1.42518	-0.82482	-1.29726
H	-1.4972	0.33169	-2.95693
H	-0.896	0.376191	1.832174
C	-1.35033	-2.06399	0.759314
C	-1.02977	2.769904	-1.8861
C	-1.10453	4.061288	-1.20196
C	-0.3414	5.147361	-1.65694
C	-1.94417	4.281321	-0.10792
C	-0.39524	6.376406	-1.0369
C	-2.02233	5.515476	0.516315
C	-1.23937	6.573091	0.057522
H	0.318787	5.013312	-2.50597
H	-2.5681	3.473867	0.251366
H	0.210737	7.204207	-1.38668
H	-2.69904	5.6452	1.3502
C	-0.86677	2.801876	-3.34552
C	0.083357	2.016562	-3.99707
C	-1.6476	3.659749	-4.13288
C	0.245692	2.054949	-5.37426
C	-1.5084	3.701074	-5.50241
C	-0.55904	2.896193	-6.13744
H	0.732075	1.374983	-3.41134
H	-2.38738	4.291534	-3.65456
H	1.004346	1.437952	-5.83646
H	-2.12853	4.353474	-6.10622
C	-1.51908	-2.10039	2.217886
C	-2.47425	-1.31008	2.870187
C	-0.75186	-2.96213	3.002938
C	-2.63931	-1.36487	4.238217
C	-0.89582	-3.02078	4.379396
C	-1.84609	-2.21721	5.007268
H	-3.1157	-0.66112	2.284748
H	-0.01147	-3.59418	2.525778
H	-3.39072	-0.76026	4.732746
H	-0.2678	-3.69084	4.950788
C	-1.26558	-3.35273	0.071105
C	-0.41619	-3.56365	-1.01708
C	-2.02823	-4.44403	0.514043
C	-0.32743	-4.79427	-1.6469
C	-1.9643	-5.66953	-0.11206
C	-1.11004	-5.85726	-1.20022
H	0.207255	-2.75162	-1.36699
H	-2.69602	-4.31698	1.358146
H	0.356941	-4.91696	-2.47559
H	-2.57018	-6.50144	0.228008
O	-2.07252	-2.20566	6.336268
C	-1.29423	-3.06399	7.149157
H	-1.44678	-4.11222	6.876266
H	-0.23029	-2.82068	7.075423
H	-1.63344	-2.90171	8.170113
O	-1.10142	-7.08998	-1.7463
C	-0.24629	-7.31994	-2.85028
H	-0.50227	-6.6683	-3.69068
H	0.801642	-7.16525	-2.57728
H	-0.39679	-8.35863	-3.13716
O	-1.23848	7.808595	0.597198
C	-2.08323	8.047827	1.707214
H	-3.13402	7.895504	1.444125
H	-1.82236	7.399949	2.549025
H	-1.92644	9.087531	1.986954
O	-0.4874	3.006454	-7.47917
C	0.460357	2.20537	-8.15951
H	1.4796	2.443466	-7.84152
H	0.268208	1.141098	-7.99531
H	0.347034	2.436098	-9.2167

Table S26. Optimised molecular structure of 2^{Pz} (OST) in XYZ-coordinates.

Atom	x	y	z
C	-1.71764	0.441418	-1.77007
C	-1.07313	1.562259	-1.22668
N	-0.52276	1.46882	-0.00518

C	-0.61698	0.312998	0.624901
C	-1.26233	-0.80829	0.082024
N	-1.81372	-0.71385	-1.13971
H	-2.18449	0.50833	-2.748
H	-0.14773	0.246418	1.60157
C	-1.33282	-2.07207	0.805483
C	-1.00107	2.826905	-1.95167
C	-1.14521	4.067818	-1.22939
C	-0.5276	5.25653	-1.66833
C	-1.90095	4.148641	-0.04854
C	-0.6622	6.438845	-0.97796
C	-2.05147	5.333164	0.64925
C	-1.43035	6.493325	0.187957
H	0.093058	5.235056	-2.5559
H	-2.40061	3.262883	0.32344
H	-0.16635	7.340374	-1.31938
H	-2.65701	5.345138	1.545694
C	-0.79318	2.800341	-3.38251
C	-0.04304	1.784686	-3.99597
C	-1.33885	3.784789	-4.2311
C	0.164615	1.744792	-5.36343
C	-1.14514	3.752741	-5.59257
C	-0.38697	2.733804	-6.17603
H	0.411824	1.015888	-3.38191
H	-1.95011	4.571138	-3.80507
H	0.763343	0.948369	-5.78462
H	-1.58636	4.507394	-6.23365
C	-1.49128	-2.04786	2.243696
C	-2.22412	-1.0299	2.886771
C	-0.91909	-3.02933	3.067096
C	-2.37743	-1.00248	4.253407
C	-1.05985	-3.00938	4.44377
C	-1.79531	-1.99257	5.050012
H	-2.70162	-0.2605	2.290983
H	-0.32418	-3.81498	2.617019
H	-2.95754	-0.22147	4.731452
H	-0.5844	-3.78056	5.035032
C	-1.22867	-3.3158	0.080138
C	-0.50673	-3.41207	-1.12037
C	-1.85369	-4.49294	0.539118
C	-0.39607	-4.60051	-1.81915
C	-1.75905	-5.67874	-0.15183
C	-1.02482	-5.74865	-1.33862
H	-0.00164	-2.53616	-1.50759
H	-2.44777	-4.45874	1.444276
H	0.184832	-4.6249	-2.73151
H	-2.26074	-6.57086	0.205378
O	-1.99461	-1.88334	6.379031
C	-1.42423	-2.87026	7.217716
H	-1.81317	-3.86437	6.978771
H	-0.33351	-2.87743	7.134059
H	-1.70821	-2.60382	8.233655
O	-0.98358	-6.95222	-1.94579
C	-0.26081	-7.05875	-3.15754
H	-0.67575	-6.39821	-3.92446
H	0.796929	-6.82248	-3.00911
H	-0.35948	-8.09385	-3.47799
O	-1.50961	7.694673	0.795881
C	-2.2711	7.786654	1.984928
H	-3.31946	7.532091	1.803704
H	-1.86849	7.13233	2.76365
H	-2.20056	8.82284	2.309296
O	-0.24638	2.785982	-7.51603
C	0.504452	1.763499	-8.1433
H	1.53931	1.758853	-7.78869
H	0.056506	0.780927	-7.9686
H	0.487167	1.986271	-9.20813

Table S27. Optimised molecular structure of **2^{Ph}DC** in XYZ-coordinates.

Atom	x	y	z
C	-1.80613	0.509426	-1.7947
C	-1.09167	1.590274	-1.26454
C	-0.51077	0.248436	0.652603

C	-1.22517	-0.83264	0.122411
H	-2.33943	0.626588	-2.72981
H	0.022275	0.131623	1.587927
C	-1.29616	-2.09845	0.848171
C	-1.02057	2.855906	-1.99097
C	-1.14328	4.074528	-1.2692
C	-0.52028	5.265195	-1.7185
C	-1.88777	4.133116	-0.07042
C	-0.64084	6.430554	-1.01758
C	-2.041	5.305782	0.624348
C	-1.41334	6.471116	0.1568
H	0.113723	5.23572	-2.59542
H	-2.39769	3.245063	0.281377
H	-0.13476	7.333323	-1.33653
H	-2.65001	5.323817	1.517339
C	-0.82928	2.831275	-3.39967
C	-0.13812	1.765236	-4.01638
C	-1.32843	3.863889	-4.23149
C	0.081805	1.738851	-5.36998
C	-1.14236	3.830793	-5.58373
C	-0.42331	2.774756	-6.17112
H	0.279408	0.977311	-3.40207
H	-1.92131	4.657916	-3.7957
H	0.649298	0.928451	-5.80541
H	-1.55528	4.597954	-6.22698
C	-1.48103	-2.07578	2.257595
C	-2.16499	-1.00668	2.887556
C	-0.97531	-3.10824	3.077835
C	-2.36273	-0.99638	4.23827
C	-1.14154	-3.09141	4.439431
C	-1.85207	-2.0379	5.034239
H	-2.58671	-0.21378	2.282777
H	-0.38584	-3.89877	2.630945
H	-2.91776	-0.2008	4.719738
H	-0.70674	-3.8768	5.041714
C	-1.18207	-3.31739	0.125289
C	-0.43906	-3.38011	-1.07416
C	-1.81115	-4.50454	0.575307
C	-0.29185	-4.55402	-1.76803
C	-1.69603	-5.67129	-0.12459
C	-0.92446	-5.71628	-1.29913
H	0.074842	-2.49465	-1.42672
H	-2.4459	-4.47042	1.451563
H	0.316484	-4.57566	-2.66141
H	-2.20689	-6.57119	0.194855
O	-2.07968	-1.93665	6.332566
C	-1.60928	-2.96217	7.204353
H	-2.04709	-3.92535	6.935045
H	-0.51939	-3.02201	7.173091
H	-1.93493	-2.67224	8.199653
O	-0.85899	-6.89136	-1.9015
C	-0.11998	-7.01375	-3.11432
H	-0.53326	-6.35842	-3.88358
H	0.933463	-6.78033	-2.94717
H	-0.22471	-8.05203	-3.41726
O	-1.48473	7.64554	0.759491
C	-2.22675	7.764508	1.970885
H	-3.2787	7.525987	1.801576
H	-1.81185	7.111208	2.740964
H	-2.12758	8.803315	2.273868
O	-0.27913	2.834224	-7.48394
C	0.41804	1.78848	-8.1567
H	1.452333	1.73258	-7.81112
H	-0.08323	0.831111	-8.00149
H	0.394506	2.052014	-9.21064
C	-0.44487	1.443581	-0.03144
C	-1.87233	-0.68571	-1.11031
H	0.140389	2.262587	0.367496
H	-2.4583	-1.50432	-1.50897

Table S28. Optimised molecular structure of **2^{Ph}RC** in XYZ-coordinates.

Atom	x	y	z
C	-1.56878	0.420631	-1.89885

C	-1.09866	1.602587	-1.27289
C	-0.77544	0.31728	0.761381
C	-1.24654	-0.86478	0.135952
H	-1.93708	0.47454	-2.91554
H	-0.40581	0.263796	1.777573
C	-1.32079	-2.09883	0.841347
C	-1.0236	2.837154	-1.97743
C	-1.12824	4.092663	-1.26931
C	-0.40245	5.225114	-1.6882
C	-1.96634	4.235707	-0.15341
C	-0.49432	6.418273	-1.01662
C	-2.08505	5.435891	0.517347
C	-1.3409	6.539244	0.092482
H	0.268941	5.141934	-2.53414
H	-2.56951	3.395255	0.168175
H	0.086031	7.279478	-1.32547
H	-2.76277	5.511809	1.356532
C	-0.84022	2.852488	-3.41099
C	-0.04918	1.890987	-4.05724
C	-1.43592	3.849004	-4.2088
C	0.146771	1.909939	-5.42319
C	-1.26735	3.866194	-5.57061
C	-0.46985	2.898366	-6.19404
H	0.457228	1.136954	-3.46685
H	-2.0688	4.594079	-3.74229
H	0.78621	1.166944	-5.87961
H	-1.74925	4.618215	-6.18389
C	-1.48886	-2.11358	2.277102
C	-2.26843	-1.14435	2.938016
C	-0.88731	-3.10775	3.063153
C	-2.43354	-1.17073	4.300372
C	-1.02709	-3.13294	4.435752
C	-1.80766	-2.16141	5.067027
H	-2.78125	-0.38744	2.357071
H	-0.26511	-3.85507	2.585789
H	-3.05504	-0.43857	4.801924
H	-0.52386	-3.90022	5.007876
C	-1.22973	-3.35572	0.133018
C	-0.39935	-3.50651	-0.98754
C	-1.96233	-4.48194	0.556678
C	-0.29463	-4.7081	-1.65829
C	-1.8843	-5.67637	-0.11469
C	-1.04553	-5.805	-1.22866
H	0.208994	-2.67136	-1.31318
H	-2.62814	-4.39246	1.406361
H	0.377487	-4.7902	-2.50138
H	-2.47028	-6.53239	0.197968
O	-2.01448	-2.10195	6.387094
C	-1.41583	-3.09098	7.210889
H	-1.76981	-4.08954	6.942231
H	-0.32592	-3.05367	7.136668
H	-1.72093	-2.85606	8.227832
O	-1.02414	-7.00781	-1.81314
C	-0.19586	-7.19169	-2.95107
H	-0.48946	-6.51875	-3.76073
H	0.85527	-7.02882	-2.69946
H	-0.34134	-8.2235	-3.26196
O	-1.37596	7.741487	0.677012
C	-2.21446	7.918914	1.808572
H	-3.2622	7.747356	1.548783
H	-1.9216	7.2486	2.620638
H	-2.07979	8.951978	2.120152
O	-0.35238	2.99973	-7.52227
C	0.434473	2.037055	-8.20695
H	1.474623	2.073721	-7.87306
H	0.035306	1.030311	-8.05955
H	0.379552	2.301922	-9.26017
C	-0.70568	1.501849	0.085547
C	-1.64082	-0.76351	-1.22215
H	-0.2826	2.369176	0.57634
H	-2.06561	-1.63013	-1.71264

Table S29. Optimised molecular structure of 3^{Py} in XYZ-coordinates.

Atom	x	y	z
O	14.9906	5.945313	26.9541
O	19.03725	6.626543	15.38755
O	6.265511	5.959942	23.17235
O	9.918563	5.402179	12.95647
N	12.06449	5.603691	18.7497
H	11.48082	5.222507	18.01311
C	11.58783	5.597914	19.96787
H	10.59487	5.183896	20.08351
C	17.7882	6.429039	15.84787
C	13.34698	6.022195	18.38128
C	12.35065	6.020688	21.07215
C	11.51256	6.542254	15.97556
H	11.30312	7.262285	16.75949
C	10.3613	6.0088	22.54787
C	15.11426	6.149111	16.66289
C	14.15461	6.450679	19.48888
H	15.13915	6.839708	19.27074
C	12.28638	6.6591	24.7009
H	11.37001	7.237053	24.71116
C	13.71209	6.003304	17.06073
C	9.525604	6.801004	21.75242
H	9.964427	7.455595	21.00785
C	8.400107	5.23183	23.73555
H	7.938628	4.617437	24.49952
C	12.65354	5.968859	23.53314
C	8.153841	6.817626	21.92959
H	7.545381	7.461703	21.30979
C	14.64995	5.195789	24.6803
H	15.553	4.601119	24.6666
C	13.69547	6.445503	20.75405
H	14.32062	6.827382	21.55064
C	14.2772	5.909738	25.82104
C	13.0857	6.642639	25.81885
H	12.81092	7.19473	26.70975
C	12.71951	5.828471	15.99223
C	15.4556	6.937629	15.5544
H	14.67387	7.452318	15.00758
C	12.97416	4.964053	14.92575
H	13.90542	4.409585	14.9049
C	11.80215	5.992494	22.35594
C	13.84791	5.240076	23.55677
H	14.13753	4.662689	22.68657
C	7.579715	6.022276	22.9209
C	12.06379	4.786001	13.89866
H	12.29551	4.096714	13.09801
C	16.14737	5.489799	17.32948
H	15.91317	4.833214	18.15967
C	9.761985	5.238109	23.55999
H	10.38387	4.614654	24.19126
C	17.47004	5.620107	16.93653
H	18.23827	5.079198	17.4723
C	10.59918	6.381429	14.95537
H	9.676399	6.949413	14.94063
C	16.7655	7.085586	15.1579
H	17.02397	7.709277	14.31009
C	10.8637	5.496726	13.9085
C	5.389923	6.735569	22.36966
H	5.468082	6.450149	21.31725
H	5.601703	7.802616	22.47777
H	4.386526	6.524456	22.73197
C	16.21237	5.226064	27.00453
H	16.91343	5.591677	26.2496
H	16.04307	4.155642	26.86123
H	16.62083	5.400759	27.99717
C	10.15713	4.531746	11.86421
H	9.283493	4.610839	11.221
H	10.26997	3.497722	12.20181
H	11.04909	4.833605	11.3081
C	20.10322	5.977941	16.05748
H	21.01085	6.273763	15.53589
H	19.99368	4.890687	16.012
H	20.16242	6.294793	17.10265

Table S30. Optimised molecular structure of **3**^{Pz} (CSS) in XYZ-coordinates.

Atom	x	y	z
N	-1.01174	4.492267	11.76156
H	-0.17563	4.750212	11.24873
C	-1.73452	3.210113	9.851725
C	-1.15711	4.933313	12.98464
H	-0.36695	5.563781	13.36904
C	-1.90112	3.603513	11.15169
C	-2.46113	2.05614	9.319494
C	-3.02123	2.105451	8.0346
H	-2.92508	3.01104	7.446794
C	-2.59017	0.868517	10.04005
H	-2.12025	0.780745	11.0132
O	1.748967	5.927135	6.387782
C	-0.81259	3.911058	8.952805
C	0.006581	3.191055	8.080999
H	-0.03435	2.107968	8.088698
C	0.881736	3.82394	7.216221
H	1.509018	3.228824	6.566674
C	0.941605	5.217302	7.192697
C	0.113725	5.955521	8.042073
H	0.148344	7.037593	7.996009
C	-0.74408	5.311371	8.906042
H	-1.40299	5.904161	9.531199
O	-4.52765	-1.14051	7.672653
C	-3.84423	-0.14349	8.259967
C	-3.27045	-0.22403	9.527044
H	-3.33502	-1.13155	10.11163
C	-3.70966	1.033034	7.516236
H	-4.15667	1.081567	6.530238
C	-4.69623	-2.34867	8.393587
H	-5.28107	-3.00276	7.750774
H	-5.23606	-2.1759	9.328812
H	-3.73139	-2.81583	8.609719
C	2.59679	5.219396	5.499138
H	2.01437	4.61311	4.80001
H	3.293009	4.578594	6.047165
H	3.153225	5.975349	4.949674
N	-3.14743	3.612235	13.22916
C	-2.39773	5.002887	15.07492
C	-2.97384	3.198169	12.02668
H	-3.72022	2.524749	11.61752
C	-2.24746	4.527708	13.77066
C	-1.72589	6.229056	15.4794
C	-1.10993	6.329285	16.7388
H	-1.12416	5.475939	17.40627
C	-1.68627	7.349368	14.64268
H	-2.20664	7.32166	13.69194
O	-5.53928	2.315779	18.83726
C	-3.22454	4.314501	16.04403
C	-3.90638	5.036971	17.0317
H	-3.83622	6.117988	17.04151
C	-4.699	4.412198	17.97471
H	-5.22731	5.0084	18.70592
C	-4.80849	3.02084	17.96561
C	-4.11978	2.278172	16.99827
H	-4.19968	1.197758	17.01776
C	-3.35549	2.913535	16.05192
H	-2.81999	2.322891	15.32115
O	0.230135	9.660943	16.72467
C	-0.4199	8.584101	16.26427
C	-1.04901	8.517455	15.02157
H	-1.06209	9.370075	14.3566
C	-0.45749	7.476068	17.11987
H	0.039352	7.542545	18.08047
C	0.310753	10.80532	15.89022
H	0.888086	11.54041	16.44607
H	0.820936	10.57003	14.95244
H	-0.68398	11.20553	15.67709
C	-6.24508	3.017472	19.84889
H	-5.55765	3.57668	20.48879
H	-6.97876	3.699993	19.4121

H	-6.75664	2.258964	20.43673

Table S31. Optimised molecular structure of 3^{Pz} (OST) in XYZ-coordinates.

Atom	x	y	z
N	-0.84407	4.264146	11.83375
H	0.047703	4.383247	11.36468
C	-1.67568	3.20463	9.820403
C	-0.96973	4.725366	13.08767
H	-0.10287	5.204179	13.51843
C	-1.83822	3.628949	11.1715
C	-2.43154	2.071855	9.342664
C	-2.86839	2.010036	8.003755
H	-2.6534	2.836147	7.336911
C	-2.75164	0.989382	10.1772
H	-2.39094	0.975746	11.19939
O	1.857095	5.932278	6.444522
C	-0.74683	3.897102	8.950754
C	-0.02021	3.197288	7.976768
H	-0.13313	2.122293	7.90434
C	0.860897	3.835847	7.126468
H	1.414364	3.254207	6.401981
C	1.033344	5.217813	7.220963
C	0.311773	5.938588	8.180705
H	0.439323	7.013393	8.230313
C	-0.55286	5.291253	9.028126
H	-1.12318	5.877923	9.739781
O	-4.62293	-1.1189	7.854627
C	-3.91098	-0.12218	8.393724
C	-3.47593	-0.09337	9.720228
H	-3.68449	-0.91466	10.39203
C	-3.59733	0.943828	7.540811
H	-3.95158	0.913396	6.51726
C	-4.98131	-2.21548	8.681053
H	-5.55958	-2.88728	8.051191
H	-5.59262	-1.88517	9.524809
H	-4.09253	-2.73318	9.051038
C	2.604698	5.250359	5.449474
H	1.942862	4.758156	4.732191
H	3.272877	4.511878	5.900239
H	3.191509	6.012479	4.942234
N	-3.19501	3.92488	13.13108
C	-2.37462	5.072317	15.10429
C	-3.0326	3.481225	11.9148
H	-3.88426	3.01413	11.43316
C	-2.17015	4.564964	13.75607
C	-1.74861	6.320422	15.48531
C	-1.29551	6.545135	16.80022
H	-1.39149	5.755519	17.53548
C	-1.56616	7.364183	14.56489
H	-1.91949	7.245907	13.54653
O	-5.45165	2.018749	18.65518
C	-3.17236	4.306457	16.02719
C	-3.89035	4.924096	17.06441
H	-3.86642	6.003288	17.15496
C	-4.66127	4.201375	17.95561
H	-5.20971	4.725155	18.72697
C	-4.73505	2.81328	17.84004
C	-4.02915	2.172876	16.81596
H	-4.08468	1.093	16.74114
C	-3.27265	2.902337	15.93113
H	-2.72246	2.378032	15.15964
O	0.043393	9.884757	16.67865
C	-0.54138	8.758813	16.23117
C	-0.97938	8.564547	14.92094
H	-0.8773	9.344827	14.17877
C	-0.70339	7.731525	17.16518
H	-0.34359	7.884064	18.17617
C	0.232219	10.94742	15.7614
H	0.718998	11.74394	16.31988
H	0.872338	10.6403	14.92952
H	-0.72464	11.30643	15.37204
C	-6.17306	2.626272	19.71212
H	-5.5015	3.157657	20.39225

H	-6.92591	3.319924	19.32732
H	-6.66466	1.815662	20.24556

Table S32. Optimised molecular structure of **4**^{Pz} (CSS) in XYZ-coordinates.

Atom	x	y	z
N	-0.95194	4.522484	11.7457
H	-0.09384	4.739308	11.25066
C	-1.7013	3.232996	9.80197
C	-1.09648	4.966217	12.96811
H	-0.29217	5.564363	13.37308
C	-1.87395	3.66479	11.11241
C	-2.38838	2.041428	9.349237
C	-2.92623	1.980573	8.050108
H	-2.83507	2.838679	7.395167
C	-2.52585	0.91593	10.17254
H	-2.05481	0.907497	11.14911
O	1.602835	6.003327	6.25708
C	-0.82842	3.942523	8.888473
C	-0.07549	3.237444	7.940406
H	-0.12639	2.155487	7.92138
C	0.756616	3.885825	7.051521
H	1.338367	3.306662	6.347882
C	0.838099	5.280592	7.075459
C	0.076612	6.004584	8.003572
H	0.129301	7.08646	7.989873
C	-0.73152	5.347063	8.895789
H	-1.34074	5.92934	9.578101
O	-4.41428	-1.27942	7.955681
C	-3.74638	-0.24236	8.463029
C	-3.18998	-0.21603	9.743796
H	-3.25239	-1.07641	10.39557
C	-3.60385	0.868973	7.619754
H	-4.04356	0.830797	6.630437
C	-4.60358	-2.42913	8.7689
H	-5.18153	-3.12633	8.167442
H	-5.15829	-2.17641	9.675812
H	-3.64432	-2.8799	9.034818
C	2.383517	5.326421	5.280788
H	1.745446	4.761406	4.59711
H	3.103503	4.655714	5.755917
H	2.911564	6.10288	4.733007
N	-3.12072	3.765456	13.12083
H	-3.97881	3.548614	13.61588
C	-2.37138	5.054926	15.06457
C	-2.97618	3.321721	11.89842
H	-3.78048	2.72356	11.49346
C	-2.19872	4.623161	13.75412
C	-1.68425	6.246427	15.51739
C	-1.14652	6.307199	16.81658
H	-1.23782	5.449083	17.47149
C	-1.54661	7.371943	14.69414
H	-2.01755	7.380443	13.71753
O	-5.67582	2.284541	18.60912
C	-3.24434	4.345396	15.97799
C	-3.99711	5.050438	16.92621
H	-3.94601	6.132383	16.94543
C	-4.82927	4.402041	17.81502
H	-5.41088	4.981179	18.5188
C	-4.911	3.007292	17.79084
C	-4.14969	2.283335	16.86255
H	-4.20258	1.201466	16.87605
C	-3.34148	2.940876	15.97041
H	-2.73242	2.35862	15.28794
O	0.341783	9.567071	16.91125
C	-0.32615	8.530078	16.40381
C	-0.88243	8.503831	15.12299
H	-0.81989	9.364232	14.47125
C	-0.46885	7.418732	17.24703
H	-0.02924	7.456846	18.23639
C	0.531229	10.7168	16.0981
H	1.109153	11.41395	16.69964
H	1.086027	10.46409	15.19123
H	-0.42798	11.16763	15.8321

C	-6.45633	2.961407	19.58558
H	-5.81813	3.526181	20.26933
H	-7.17622	3.632333	19.11061
H	-6.9845	2.184942	20.13324

Table S33. Optimised molecular structure of **4**^{Pz} (OST) in XYZ-coordinates.

Atom	x	y	z
N	-0.68601	4.228612	11.82192
H	0.208783	4.318031	11.35897
C	-1.59811	3.168876	9.813851
C	-0.80508	4.695366	13.10261
H	0.063734	5.162527	13.53686
C	-1.706	3.590656	11.17932
C	-2.31039	1.999439	9.409499
C	-2.82982	1.884035	8.098042
H	-2.7183	2.711001	7.408047
C	-2.52012	0.925565	10.29981
H	-2.07805	0.957447	11.2885
O	1.433144	6.110647	6.209602
C	-0.79025	3.911448	8.895583
C	-0.15486	3.264154	7.817663
H	-0.22758	2.187144	7.731153
C	0.604682	3.958443	6.907054
H	1.103458	3.425467	6.109611
C	0.73663	5.347255	7.036834
C	0.109499	6.014178	8.105345
H	0.210811	7.090137	8.175731
C	-0.62242	5.311179	9.019319
H	-1.12461	5.848116	9.815338
O	-4.41313	-1.31762	8.157312
C	-3.72781	-0.28358	8.619122
C	-3.20666	-0.20152	9.917777
H	-3.31987	-1.0209	10.61377
C	-3.53213	0.776678	7.715895
H	-3.96427	0.700425	6.725736
C	-4.67095	-2.42089	9.021525
H	-5.25359	-3.12448	8.433068
H	-5.24508	-2.10024	9.893172
H	-3.73597	-2.8874	9.338624
C	2.076593	5.510367	5.088596
H	1.344367	5.023831	4.441015
H	2.826361	4.787523	5.416985
H	2.558358	6.325707	4.555724
N	-3.00601	3.905833	13.12143
H	-3.9079	3.844203	13.58349
C	-2.27956	5.083125	15.09519
C	-2.88904	3.434725	11.88468
H	-3.76831	3.001841	11.43115
C	-1.99406	4.571355	13.7828
C	-1.619	6.282536	15.55244
C	-1.31235	6.460745	16.91666
H	-1.5425	5.66987	17.61994
C	-1.27033	7.322625	14.67544
H	-1.51036	7.23827	13.62179
O	-5.78892	2.189846	18.35602
C	-3.19151	4.349646	15.94357
C	-4.03323	5.018203	16.84797
H	-4.01157	6.100572	16.88832
C	-4.9127	4.336991	17.6631
H	-5.55508	4.893649	18.33177
C	-4.97443	2.941582	17.60519
C	-4.14195	2.252818	16.71691
H	-4.18283	1.170249	16.69635
C	-3.27164	2.941818	15.90634
H	-2.6069	2.381186	15.25843
O	0.238107	9.699979	17.00524
C	-0.36252	8.62625	16.47671
C	-0.66119	8.478107	15.11935
H	-0.42844	9.261379	14.41087
C	-0.69425	7.600728	17.36875
H	-0.44324	7.719224	18.41619
C	0.600284	10.76488	16.14053
H	1.070265	11.5154	16.77169

H	1.310165	10.42778	15.38056
H	-0.28126	11.19295	15.65621
C	-6.64006	2.84075	19.2869
H	-6.05799	3.402308	20.02211
H	-7.33582	3.512418	18.77734
H	-7.19529	2.050528	19.78659

Table S34. Optimised molecular structure of 2^{Ph} (C-C distance 1.380 Å) in XYZ-coordinates.

Atom	x	y	z
C	-1.36077	0.361666	-1.94148
C	-1.09496	1.614768	-1.27985
C	-0.99781	0.35268	0.814936
C	-1.26458	-0.90029	0.153453
H	-1.57577	0.378488	-3.00264
H	-0.78197	0.336552	1.875941
C	-1.3432	-2.0977	0.834952
C	-1.0179	2.812715	-1.96057
C	-1.09085	4.103534	-1.26683
C	-0.24849	5.164164	-1.6284
C	-2.02026	4.343886	-0.25636
C	-0.31118	6.383323	-0.99231
C	-2.10256	5.5685	0.388967
C	-1.23984	6.597048	0.026324
H	0.476488	5.014161	-2.42003
H	-2.71344	3.557204	0.017222
H	0.353591	7.193705	-1.26628
H	-2.84901	5.711483	1.158628
C	-0.86345	2.86828	-3.41857
C	0.021296	2.034012	-4.09953
C	-1.57962	3.801625	-4.18122
C	0.178032	2.097898	-5.47593
C	-1.44239	3.872522	-5.54899
C	-0.56169	3.017588	-6.21138
H	0.620244	1.329847	-3.53406
H	-2.26555	4.47432	-3.67968
H	0.886761	1.43872	-5.95889
H	-2.01062	4.586051	-6.1335
C	-1.49733	-2.1523	2.293114
C	-2.38106	-1.30727	2.977423
C	-0.79234	-3.08566	3.052655
C	-2.5323	-1.37676	4.345037
C	-0.92604	-3.16327	4.429434
C	-1.7995	-2.30211	5.08641
H	-2.97517	-0.599	2.412169
H	-0.11125	-3.7636	2.551401
H	-3.2277	-0.72902	4.86524
H	-0.34627	-3.89414	4.976909
C	-1.27177	-3.38858	0.140661
C	-0.33828	-3.63138	-0.86528
C	-2.12078	-4.44612	0.495708
C	-0.25726	-4.85569	-1.51179
C	-2.05965	-5.66467	-0.14175
C	-1.12595	-5.88113	-1.1552
H	0.358757	-2.84659	-1.1346
H	-2.84996	-4.29384	1.28299
H	0.492703	-5.00069	-2.27769
H	-2.73017	-6.47223	0.126571
O	-2.00894	-2.29681	6.421557
C	-1.29489	-3.22369	7.201482
H	-1.53289	-4.25466	6.918084
H	-0.21363	-3.06957	7.118682
H	-1.60251	-3.0555	8.231994
O	-1.13406	-7.10895	-1.72012
C	-0.20985	-7.36347	-2.74863
H	-0.35997	-6.68917	-3.5987
H	0.822205	-7.26881	-2.39393
H	-0.38532	-8.38882	-3.06917
O	-1.23359	7.825246	0.589763
C	-2.15446	8.077951	1.621961
H	-3.18755	7.97874	1.271701
H	-1.99806	7.405694	2.472466

H	-1.9815	9.104514	1.939909
O	-0.48783	3.161132	-7.55312
C	0.38777	2.314977	-8.25607
H	1.426882	2.46174	-7.94199
H	0.11907	1.261193	-8.12489
H	0.290802	2.581347	-9.30702
C	-0.91866	1.522363	0.148053
C	-1.44035	-0.80797	-1.27455
H	-0.64235	2.418986	0.688687
H	-1.71679	-1.70466	-1.81508

Table S35. Optimised molecular structure of **2**^{Ph} (C-C distance 1.464 Å) in XYZ-coordinates.

Atom	x	y	z
C	-1.39633	0.374691	-1.9374
C	-1.09109	1.605843	-1.27644
C	-0.94579	0.34905	0.805283
C	-1.25192	-0.88197	0.144512
H	-1.63862	0.399396	-2.99255
H	-0.70304	0.325045	1.860342
C	-1.33442	-2.15139	0.869127
C	-1.01149	2.876297	-1.99956
C	-1.10051	4.146263	-1.29027
C	-0.30378	5.240338	-1.66499
C	-1.99439	4.343852	-0.23603
C	-0.37715	6.445789	-1.00596
C	-2.08395	5.553462	0.434069
C	-1.26812	6.614255	0.05444
H	0.397077	5.123927	-2.48333
H	-2.65602	3.534485	0.048222
H	0.25292	7.279837	-1.29076
H	-2.80212	5.661519	1.235716
C	-0.84655	2.9063	-3.44692
C	-0.0061	2.014927	-4.11689
C	-1.51319	3.864409	-4.22817
C	0.152263	2.048372	-5.49315
C	-1.37025	3.907034	-5.59573
C	-0.53669	2.995286	-6.24406
H	0.558735	1.293053	-3.5394
H	-2.1688	4.575404	-3.73942
H	0.825695	1.345687	-5.9652
H	-1.90096	4.639015	-6.19262
C	-1.50044	-2.17902	2.316494
C	-2.3411	-1.27703	2.987324
C	-0.84542	-3.13545	3.096584
C	-2.4969	-1.31573	4.35466
C	-0.98769	-3.18391	4.473037
C	-1.81571	-2.26665	5.113574
H	-2.89991	-0.55184	2.408276
H	-0.19329	-3.85126	2.610003
H	-3.15903	-0.62488	4.862691
H	-0.44702	-3.93378	5.03471
C	-1.24749	-3.4222	0.160569
C	-0.34953	-3.62355	-0.88935
C	-2.05133	-4.51274	0.530427
C	-0.26178	-4.83362	-1.55933
C	-1.98	-5.71836	-0.12858
C	-1.08401	-5.89083	-1.18409
H	0.316385	-2.81656	-1.17044
H	-2.75639	-4.39296	1.344638
H	0.459956	-4.94471	-2.35739
H	-2.61621	-6.54923	0.151915
O	-2.02723	-2.22817	6.447501
C	-1.36439	-3.17796	7.24506
H	-1.65466	-4.19962	6.977242
H	-0.27642	-3.08115	7.163557
H	-1.66588	-2.97721	8.27152
O	-1.08006	-7.10891	-1.76897
C	-0.19329	-7.31973	-2.83963
H	-0.39841	-6.63637	-3.67072
H	0.849593	-7.20058	-2.52652
H	-0.35292	-8.34445	-3.1702
O	-1.2743	7.831961	0.63934
C	-2.15774	8.039651	1.713632

H	-3.20127	7.916288	1.404552
H	-1.94656	7.357423	2.54408
H	-2.00077	9.065128	2.043081
O	-0.45536	3.113597	-7.58746
C	0.368805	2.207222	-8.27766
H	1.415111	2.298745	-7.96674
H	0.039696	1.173077	-8.12926
H	0.285452	2.462212	-9.33257
C	-0.86999	1.52367	0.134188
C	-1.47244	-0.79991	-1.26627
H	-0.56924	2.418966	0.663977
H	-1.77351	-1.69524	-1.79588

Table S36. Optimised molecular structure of **2^{Ph}** (C-C distance 1.544 Å) in XYZ-coordinates.

Atom	x	y	z
C	-1.5895	0.435894	-1.88661
C	-1.08853	1.59578	-1.27207
C	-0.73552	0.303792	0.744847
C	-1.23683	-0.85593	0.130434
H	-1.95875	0.493855	-2.90337
H	-0.36653	0.246435	1.761772
C	-1.31968	-2.19385	0.896614
C	-1.00799	2.934855	-2.03648
C	-1.12435	4.176128	-1.30488
C	-0.42134	5.32964	-1.70162
C	-1.94022	4.293996	-0.17304
C	-0.52176	6.512342	-1.00747
C	-2.05247	5.479548	0.532865
C	-1.33954	6.601139	0.119978
H	0.232976	5.275524	-2.56347
H	-2.51946	3.436488	0.146633
H	0.036735	7.389263	-1.3124
H	-2.70715	5.521139	1.392987
C	-0.82164	2.924848	-3.46937
C	-0.06791	1.934729	-4.11188
C	-1.39163	3.917884	-4.28896
C	0.108035	1.918663	-5.48495
C	-1.22652	3.911743	-5.65388
C	-0.47429	2.909993	-6.26906
H	0.410673	1.170004	-3.5126
H	-1.99517	4.692943	-3.83209
H	0.710578	1.138371	-5.93031
H	-1.68397	4.671666	-6.2761
C	-1.50136	-2.18089	2.330135
C	-2.25607	-1.18268	2.974556
C	-0.93561	-3.1684	3.147141
C	-2.42589	-1.17233	4.339105
C	-1.09658	-3.16766	4.521875
C	-1.84499	-2.1641	5.13029
H	-2.7334	-0.41785	2.37447
H	-0.33431	-3.94573	2.690971
H	-3.02117	-0.40709	4.822812
H	-0.62649	-3.94529	5.108904
C	-1.20645	-3.43666	0.166783
C	-0.39056	-3.55843	-0.96454
C	-1.91324	-4.58761	0.564263
C	-0.28107	-4.74551	-1.6687
C	-1.81596	-5.7715	-0.1283
C	-0.99727	-5.86446	-1.25476
H	0.190751	-2.70265	-1.28515
H	-2.56827	-4.52989	1.425319
H	0.374138	-4.79026	-2.5283
H	-2.37809	-6.64607	0.176763
O	-2.06387	-2.0708	6.460498
C	-1.49755	-3.05574	7.289069
H	-1.87525	-4.05373	7.04173
H	-0.40446	-3.05868	7.219346
H	-1.79082	-2.80358	8.30653
O	-0.96342	-7.06726	-1.87003
C	-0.15671	-7.19598	-3.0144
H	-0.47322	-6.50996	-3.80751
H	0.898775	-7.01505	-2.78402
H	-0.27717	-8.22164	-3.35844

O	-1.37619	7.802559	0.737228
C	-2.18313	7.927504	1.882083
H	-3.2381	7.744132	1.651504
H	-1.86465	7.241207	2.674113
H	-2.06527	8.953006	2.227441
O	-0.36752	2.985394	-7.61405
C	0.371611	1.985731	-8.27071
H	1.41853	1.983152	-7.94851
H	-0.05816	0.992594	-8.10099
H	0.324722	2.220052	-9.33266
C	-0.66377	1.495185	0.063374
C	-1.66159	-0.7554	-1.20508
H	-0.23966	2.365289	0.549764
H	-2.08677	-1.62532	-1.69098

Table S37. Optimised molecular structure of **2^{Py}** (C-C distance 1.379 Å) in XYZ-coordinates.

Atom	x	y	z
C	-1.23887	0.362779	-1.77828
C	-1.02553	1.649686	-1.15058
C	-0.95745	0.398914	0.919908
C	-1.20024	-0.82639	0.203928
N	-1.3288	-0.76777	-1.17371
H	-1.40264	0.345505	-2.85348
H	-0.76866	0.352065	1.984453
C	-1.29363	-2.05062	0.831771
C	-0.98041	2.820285	-1.87811
C	-1.13391	4.129196	-1.2354
C	-0.3486	5.222076	-1.62779
C	-2.08505	4.350196	-0.24049
C	-0.48802	6.457436	-1.03686
C	-2.24548	5.590074	0.357511
C	-1.43908	6.653541	-0.03547
H	0.393012	5.085518	-2.40619
H	-2.73456	3.535304	0.056763
H	0.132175	7.294583	-1.33358
H	-3.00734	5.7176	1.114576
C	-0.76703	2.822029	-3.32876
C	0.183125	2.000727	-3.93323
C	-1.49268	3.686417	-4.16086
C	0.393291	2.008449	-5.30365
C	-1.30215	3.700997	-5.5234
C	-0.35714	2.857972	-6.10946
H	0.792105	1.355564	-3.3108
H	-2.2299	4.34602	-3.71831
H	1.149983	1.362054	-5.7271
H	-1.87752	4.358287	-6.164
C	-1.41385	-2.13451	2.295155
C	-2.33436	-1.35741	3.007803
C	-0.63431	-3.03025	3.024206
C	-2.45364	-1.45575	4.377533
C	-0.7336	-3.1351	4.402282
C	-1.64797	-2.3423	5.089307
H	-2.9818	-0.67916	2.464388
H	0.075575	-3.65758	2.497542
H	-3.1779	-0.86132	4.92135
H	-0.09683	-3.83451	4.926917
C	-1.29349	-3.31955	0.104377
C	-0.52947	-3.53342	-1.04345
C	-2.06437	-4.39573	0.569132
C	-0.53413	-4.74882	-1.70732
C	-2.09001	-5.60526	-0.08744
C	-1.3232	-5.79332	-1.23583
H	0.090316	-2.73209	-1.41923
H	-2.66867	-4.2655	1.458934
H	0.083525	-4.87126	-2.58685
H	-2.70242	-6.42471	0.268892
O	-1.82974	-2.37012	6.428106
C	-1.04452	-3.26318	7.178763

H	-1.22302	-4.30211	6.881277
H	0.023247	-3.04009	7.079653
H	-1.34275	-3.13168	8.217297
O	-1.40333	-7.01285	-1.81352
C	-0.65526	-7.23542	-2.98267
H	-0.94872	-6.54846	-3.78369
H	0.419512	-7.13156	-2.79792
H	-0.86811	-8.25759	-3.29078
O	-1.51002	7.898284	0.482491
C	-2.45591	8.135415	1.496433
H	-3.47724	7.964093	1.140011
H	-2.27065	7.504829	2.372502
H	-2.34523	9.181114	1.777194
O	-0.23561	2.943711	-7.45129
C	0.699318	2.102657	-8.08155
H	1.71918	2.309644	-7.7399
H	0.469292	1.046277	-7.90671
H	0.628919	2.313825	-9.14691
C	-0.88538	1.581531	0.278081
H	-0.64786	2.484175	0.827375

Table S38. Optimised molecular structure of **2**[Py] (C-C distance 1.462 Å) in XYZ-coordinates.

Atom	x	y	z
C	-1.31964	0.387138	-1.77235
C	-1.06451	1.651174	-1.14294
C	-0.96971	0.402702	0.916377
C	-1.23884	-0.80196	0.19604
N	-1.40943	-0.74937	-1.16407
H	-1.51211	0.376005	-2.84242
H	-0.76405	0.347706	1.977311
C	-1.31757	-2.10159	0.861012
C	-0.99953	2.890762	-1.91536
C	-1.16404	4.181805	-1.26142
C	-0.40615	5.295647	-1.65736
C	-2.09337	4.376646	-0.23752
C	-0.55251	6.520561	-1.04827
C	-2.25727	5.605157	0.380562
C	-1.4801	6.688026	-0.01964
H	0.322829	5.18067	-2.45086
H	-2.72482	3.549026	0.062694
H	0.047193	7.371307	-1.34866
H	-3.00155	5.710399	1.158362
C	-0.75408	2.862047	-3.35031
C	0.150826	1.968385	-3.92725
C	-1.40469	3.759554	-4.21306
C	0.384163	1.938742	-5.29254
C	-1.18627	3.739996	-5.57061
C	-0.29061	2.824738	-6.12606
H	0.707343	1.298153	-3.28317
H	-2.10926	4.47016	-3.79733
H	1.103147	1.235823	-5.6911
H	-1.70505	4.4232	-6.23221
C	-1.42566	-2.16723	2.317506
C	-2.31231	-1.353	3.035708
C	-0.66149	-3.07662	3.050295
C	-2.41253	-1.42963	4.40762
C	-0.74385	-3.16049	4.43018
C	-1.62311	-2.33081	5.120043
H	-2.95117	-0.6665	2.492895
H	0.02654	-3.72726	2.523096
H	-3.11113	-0.8073	4.953889
H	-0.11872	-3.87018	4.955134
C	-1.29912	-3.34965	0.1182
C	-0.5803	-3.5165	-1.06912
C	-2.01111	-4.4644	0.594059
C	-0.57328	-4.71818	-1.75558
C	-2.02121	-5.66132	-0.0836
C	-1.30124	-5.80021	-1.26947
H	-0.00414	-2.68802	-1.45448
H	-2.58449	-4.37104	1.508477
H	0.00796	-4.80223	-2.66401
H	-2.58868	-6.50854	0.282228
O	-1.78468	-2.33475	6.461314

C	-1.01554	-3.24117	7.212917
H	-1.23019	-4.27806	6.93279
H	0.056816	-3.05217	7.094646
H	-1.29368	-3.08624	8.253781
O	-1.36379	-7.01064	-1.86659
C	-0.66796	-7.18313	-3.07614
H	-1.02567	-6.49158	-3.84642
H	0.41034	-7.04409	-2.94259
H	-0.86004	-8.20614	-3.39484
O	-1.55837	7.924511	0.516267
C	-2.48121	8.132829	1.5575
H	-3.5085	7.949154	1.225256
H	-2.26291	7.494182	2.419995
H	-2.38023	9.176547	1.849121
O	-0.13849	2.880455	-7.46614
C	0.746022	1.965709	-8.06606
H	1.771406	2.105317	-7.70737
H	0.438128	0.931157	-7.88091
H	0.710577	2.165011	-9.13545
C	-0.89628	1.590956	0.272392
H	-0.64254	2.492127	0.816965

Table S39. Optimised molecular structure of 2^{Py} (C-C distance 1.560 Å) in XYZ-coordinates.

Atom	x	y	z
C	-1.48914	0.434857	-1.75909
C	-1.04825	1.630109	-1.16756
C	-0.75388	0.357154	0.842107
C	-1.21854	-0.77938	0.159736
N	-1.57519	-0.71842	-1.13322
H	-1.81131	0.442782	-2.7967
H	-0.43044	0.273531	1.871342
C	-1.3173	-2.15764	0.883783
C	-0.98627	2.959805	-1.98099
C	-1.18897	4.211466	-1.29068
C	-0.5269	5.386658	-1.69524
C	-2.04839	4.319515	-0.19007
C	-0.70941	6.58119	-1.03959
C	-2.24295	5.516682	0.476565
C	-1.57127	6.660806	0.055396
H	0.161291	5.341032	-2.53071
H	-2.59823	3.44443	0.134384
H	-0.18286	7.475764	-1.34995
H	-2.92913	5.549545	1.312149
C	-0.7199	2.904976	-3.39807
C	0.09807	1.917349	-3.96172
C	-1.27464	3.849431	-4.28382
C	0.349179	1.857842	-5.3215
C	-1.03391	3.800114	-5.63606
C	-0.21835	2.801911	-6.17211
H	0.569212	1.191374	-3.3104
H	-1.92751	4.619021	-3.88991
H	0.997087	1.080951	-5.70456
H	-1.4799	4.521547	-6.3102
C	-1.42743	-2.17092	2.330072
C	-2.22011	-1.24093	3.024204
C	-0.75002	-3.12278	3.099506
C	-2.32225	-1.25994	4.39646
C	-0.83898	-3.15067	4.48092
C	-1.62902	-2.21418	5.141162
H	-2.78432	-0.50744	2.460804
H	-0.12117	-3.84916	2.598503
H	-2.94907	-0.54887	4.92112
H	-0.28312	-3.89796	5.031114
C	-1.29234	-3.38318	0.129808
C	-0.6381	-3.49892	-1.10466
C	-1.93517	-4.53931	0.616526
C	-0.61507	-4.68688	-1.81203
C	-1.92475	-5.72313	-0.08149
C	-1.26185	-5.81114	-1.30596
H	-0.1266	-2.63847	-1.51121
H	-2.4732	-4.48525	1.555049
H	-0.08644	-4.72753	-2.75495
H	-2.43773	-6.6	0.295039

O	-1.78691	-2.15448	6.481601
C	-1.11086	-3.10685	7.265186
H	-1.42604	-4.12624	7.017942
H	-0.02507	-3.02782	7.14469
H	-1.3729	-2.89152	8.299458
O	-1.30205	-7.01384	-1.92063
C	-0.66449	-7.13555	-3.16792
H	-1.09613	-6.45219	-3.90703
H	0.411401	-6.94546	-3.08931
H	-0.82367	-8.16233	-3.49252
O	-1.68882	7.875031	0.634844
C	-2.54579	7.99333	1.743885
H	-3.5803	7.749854	1.479103
H	-2.22496	7.348448	2.568989
H	-2.49245	9.033421	2.060166
O	-0.03914	2.833657	-7.51022
C	0.765816	1.835996	-8.08903
H	1.792308	1.878348	-7.70934
H	0.356807	0.836295	-7.9078
H	0.771657	2.032644	-9.15952
C	-0.67448	1.557033	0.180725
H	-0.29481	2.437956	0.684751

Table S40. Optimised molecular structure of **2^Pz^** (C-C distance 1.378 Å) in XYZ-coordinates.

Atom	x	y	z
C	-1.29575	0.310192	-1.88843
C	-1.10375	1.567092	-1.20423
N	-1.01231	1.528582	0.176242
C	-1.10057	0.384443	0.756055
C	-1.29027	-0.87277	0.071624
N	-1.38391	-0.83407	-1.30869
H	-1.43441	0.322456	-2.96648
H	-0.96377	0.373123	1.834413
C	-1.34753	-2.07481	0.742974
C	-1.04268	2.768885	-1.87568
C	-1.12867	4.061341	-1.19947
C	-0.42525	5.166467	-1.70244
C	-1.91225	4.267112	-0.06275
C	-0.48433	6.39913	-1.09366
C	-1.99295	5.504615	0.551933
C	-1.27133	6.580367	0.042559
H	0.192901	5.042099	-2.58338
H	-2.4794	3.441971	0.343271
H	0.074816	7.243087	-1.47895
H	-2.62278	5.619863	1.423632
C	-0.86478	2.797992	-3.33507
C	0.123791	2.050456	-3.96872
C	-1.66961	3.616616	-4.13797
C	0.301383	2.086976	-5.34419
C	-1.51427	3.656891	-5.50476
C	-0.5256	2.889481	-6.12201
H	0.788026	1.440145	-3.36765
H	-2.43749	4.220795	-3.6691
H	1.090278	1.498204	-5.79213
H	-2.15012	4.277777	-6.12413
C	-1.52129	-2.10685	2.202936
C	-2.5102	-1.35325	2.84535
C	-0.7228	-2.92724	2.997405
C	-2.6774	-1.40088	4.212663
C	-0.86992	-2.97945	4.373636
C	-1.85297	-2.21098	4.991116
H	-3.17344	-0.73752	2.24886
H	0.041225	-3.53332	2.524504
H	-3.45253	-0.8249	4.703333
H	-0.21626	-3.61826	4.951904
C	-1.26052	-3.36592	0.063368
C	-0.47139	-3.56902	-1.06977
C	-1.96929	-4.47134	0.55792
C	-0.38941	-4.80456	-1.68864
C	-1.90944	-5.70183	-0.05536
C	-1.11611	-5.88061	-1.18751
H	0.099339	-2.74322	-1.4695

H	-2.59256	-4.34865	1.43544
H	0.245131	-4.91794	-2.55718
H	-2.47357	-6.54575	0.322744
O	-2.08554	-2.19423	6.320987
C	-1.28104	-3.00723	7.140173
H	-1.38644	-4.06616	6.881263
H	-0.22526	-2.72401	7.07308
H	-1.62961	-2.8511	8.159337
O	-1.11045	-7.12114	-1.72091
C	-0.33186	-7.33916	-2.87175
H	-0.65772	-6.70408	-3.70232
H	0.730806	-7.15853	-2.67738
H	-0.47386	-8.38402	-3.14178
O	-1.27656	7.822547	0.571379
C	-2.05072	8.043802	1.724828
H	-3.11371	7.859889	1.535763
H	-1.71973	7.413091	2.556649
H	-1.90989	9.090155	1.989569
O	-0.4424	2.997227	-7.46518
C	0.537295	2.234293	-8.12613
H	1.546568	2.509842	-7.80193
H	0.387598	1.161787	-7.96221
H	0.429543	2.453621	-9.18671

Table S41. Optimised molecular structure of **2**^{Pz} (C-C distance 1.460 Å) in XYZ-coordinates.

Atom	x	y	z
C	-1.30913	0.325295	-1.88761
C	-1.09219	1.561924	-1.19762
N	-0.97564	1.528959	0.169186
C	-1.06559	0.378932	0.751506
C	-1.28216	-0.85783	0.061456
N	-1.39947	-0.82477	-1.30533
H	-1.46212	0.343073	-2.96313
H	-0.91301	0.361957	1.827143
C	-1.34395	-2.1312	0.773005
C	-1.03098	2.835387	-1.90906
C	-1.1334	4.10928	-1.2209
C	-0.47404	5.241829	-1.72959
C	-1.88508	4.281344	-0.05446
C	-0.54884	6.464279	-1.10417
C	-1.97762	5.507808	0.578319
C	-1.30369	6.61006	0.059266
H	0.124768	5.144169	-2.62695
H	-2.41775	3.436849	0.357623
H	-0.02368	7.327555	-1.49457
H	-2.58134	5.595226	1.471492
C	-0.84584	2.841813	-3.35928
C	0.113345	2.045942	-3.98394
C	-1.61871	3.679203	-4.17775
C	0.290615	2.05463	-5.35913
C	-1.46131	3.693265	-5.54417
C	-0.50399	2.877769	-6.14965
H	0.756573	1.4232	-3.37334
H	-2.36695	4.315018	-3.71933
H	1.056631	1.429993	-5.7982
H	-2.0737	4.32778	-6.17335
C	-1.52412	-2.13982	2.223975
C	-2.47851	-1.33294	2.858285
C	-0.76153	-2.98336	3.033194
C	-2.64493	-1.35287	4.225656
C	-0.91062	-3.00958	4.409272
C	-1.85718	-2.18817	5.016012
H	-3.11749	-0.70084	2.252997
H	-0.02086	-3.62467	2.569972
H	-3.39374	-0.73658	4.708234
H	-0.28454	-3.66727	4.996908
C	-1.24704	-3.40442	0.081707
C	-0.49193	-3.57767	-1.08211
C	-1.91637	-4.53414	0.583371
C	-0.40456	-4.80281	-1.71866
C	-1.84723	-5.75499	-0.04603
C	-1.08809	-5.90216	-1.2064
H	0.047723	-2.73509	-1.48914

H	-2.51876	-4.43512	1.47814
H	0.202439	-4.89135	-2.60954
H	-2.38096	-6.61569	0.33843
O	-2.08713	-2.141	6.345344
C	-1.32165	-2.97942	7.17634
H	-1.47839	-4.03574	6.933332
H	-0.25357	-2.7484	7.104482
H	-1.66115	-2.7913	8.193164
O	-1.07178	-7.13557	-1.7547
C	-0.33215	-7.32129	-2.93673
H	-0.70396	-6.68406	-3.74602
H	0.732553	-7.11803	-2.77981
H	-0.46015	-8.3659	-3.21454
O	-1.32591	7.844796	0.603555
C	-2.06369	8.03013	1.787052
H	-3.12729	7.818833	1.633729
H	-1.68522	7.398755	2.597786
H	-1.94215	9.076718	2.060184
O	-0.41715	2.961594	-7.49402
C	0.529928	2.149143	-8.14362
H	1.549991	2.389507	-7.82534
H	0.337629	1.086438	-7.96164
H	0.428826	2.355228	-9.20751

Table S42. Optimised molecular structure of **2^{Pz}** (C-C distance 1.530 Å) in XYZ-coordinates.

Atom	x	y	z
C	-1.40643	0.352383	-1.87316
C	-1.08266	1.552516	-1.20004
N	-0.85206	1.519727	0.130667
C	-0.93939	0.361544	0.730247
C	-1.26455	-0.83825	0.057414
N	-1.4938	-0.8058	-1.27364
H	-1.63259	0.373146	-2.93466
H	-0.7125	0.341211	1.791658
C	-1.3372	-2.17138	0.804669
C	-1.01532	2.886776	-1.94577
C	-1.12757	4.138792	-1.23818
C	-0.50602	5.299665	-1.73769
C	-1.85157	4.271866	-0.04641
C	-0.59885	6.50736	-1.08804
C	-1.95877	5.483092	0.611583
C	-1.329	6.613038	0.096041
H	0.079623	5.233345	-2.64646
H	-2.35203	3.407603	0.365696
H	-0.10305	7.390379	-1.47284
H	-2.53938	5.537361	1.522563
C	-0.83019	2.871255	-3.38758
C	0.063317	1.99632	-4.01
C	-1.54202	3.754281	-4.2172
C	0.237712	1.979604	-5.38443
C	-1.38374	3.745661	-5.58319
C	-0.49143	2.855319	-6.18251
H	0.657378	1.328458	-3.39741
H	-2.24608	4.443275	-3.76607
H	0.951482	1.292878	-5.8189
H	-1.94942	4.417416	-6.21752
C	-1.52473	-2.15383	2.246096
C	-2.4168	-1.26597	2.868846
C	-0.82836	-3.03899	3.074292
C	-2.59015	-1.25611	4.234243
C	-0.9883	-3.0377	4.449205
C	-1.87339	-2.14017	5.040372
H	-3.00208	-0.5919	2.254767
H	-0.12895	-3.73445	2.625658
H	-3.29111	-0.57755	4.705112
H	-0.41497	-3.73166	5.048908
C	-1.22805	-3.42419	0.097237
C	-0.49741	-3.56032	-1.08998
C	-1.86015	-4.5818	0.59082
C	-0.39286	-4.77159	-1.74869
C	-1.77036	-5.78932	-0.05972
C	-1.03269	-5.89829	-1.23876
H	0.010455	-2.69826	-1.49775

H	-2.45193	-4.51253	1.495345
H	0.193423	-4.8284	-2.6559
H	-2.27509	-6.66949	0.320029
O	-2.10572	-2.05775	6.367641
C	-1.40837	-2.93926	7.213749
H	-1.64141	-3.98428	6.98332
H	-0.32578	-2.78872	7.144855
H	-1.73776	-2.71326	8.226174
O	-0.99606	-7.12226	-1.80768
C	-0.27918	-7.26851	-3.00897
H	-0.68731	-6.62952	-3.79921
H	0.78283	-7.03769	-2.87315
H	-0.38432	-8.31184	-3.30087
O	-1.36906	7.837054	0.663931
C	-2.08084	7.980931	1.86877
H	-3.14203	7.743512	1.738506
H	-1.66491	7.345677	2.657906
H	-1.98045	9.02535	2.15836
O	-0.40083	2.919687	-7.52786
C	0.481152	2.032165	-8.17073
H	1.516927	2.19771	-7.85551
H	0.209446	0.988676	-7.97902
H	0.394337	2.235646	-9.23639

11. References

(1) (a) Oliveira, C. C.; Pfaltz, A.; Correia, C. R. D. Quaternary Stereogenic Centers through Enantioselective Heck Arylation of Acyclic Olefins with Aryldiazonium Salts: Application in a Concise Synthesis of (R)-Verapamil. *Angew. Chem., Int. Ed.* **2015**, *54*, 14036– 14039. (b) Favier, I.; Duñach, C. New protic salts of aprotic polar solvents. *Tetrahedron Lett.* **2004**, *45*, 3393–3395. (c) Bartlett, P. N.; Cook, D. C.; George, M. W.; Ke, J.; Levason, W.; Reid, G.; Su, W.; Zhang, W. Phase Behaviour and Conductivity Study on Multi-Component Mixtures for Electrodeposition in Supercritical Fluids. *Phys. Chem. Chem. Phys*. **2010**, *12*, 492– 501.

(2) Fulmer, G. R.; Miller, A. J. M.; Sherden, N. H.; Gottlieb, H. E.; Nudelman, A.; Stoltz, B. M.; Bercaw, J. E.; Goldberg, K. I. NMR Chemical Shifts of Trace Impurities: Common Laboratory Solvents, Organics, and Gases in Deuterated Solvents Relevant to the Organometallic Chemist. *Organometallics* **2010**, *29*, 2176– 2179.

(3) (a) Krejčik, M.; Daněk, M.; Hartl, F. J. Electroanal. Chem. Interfacial Electrochem. 1991, 317, 179– 187 (b) Klein, J.; Stuckmann, A.; Sobottka, S.; Suntrup, L.; van der Meer, M.; Hommes, P.; Reissig, H.-U.; Sarkar, B. Ruthenium Complexes with Strongly Electron-Donating Terpyridine Ligands: Effect of the Working Electrode on Electrochemical and Spectroelectrochemical Properties. *Chem. Eur. J.* **2017**, *23*, 12314−12325.

(4) (a) A. L. Single-crystal structure validation with the program PLATON. *J. Appl. Crystallogr*. **2003**, *36*, 7−13. (b) Sheldrick, G. M. A short history of SHELX. *Acta Crystallogr. A* **2008**, *64*, 112–122.

(5) Sheldrick, G. M. Crystal structure refinement with SHELXL. *Acta Crystallogr. C* **2015**, *71*, 3–8.

(6) Sheldrick, G. M. SHELXT – Integrated space-group and crystal-structure determination. *Acta Crystallogr. A* **2015**, *71*, 3–8.

(7) Plietzsch, O.; Schade, A.; Hafner, A.; Huuskonen, J.; Rissanen, K.; Nieger, M.; Muller, T.; Brase, S. Synthesis and Topological Determination of Hexakis-Substituted 1,4-Ditritylbenzene and Nonakis-Substituted 1,3,5-Tritritylbenzene Derivatives: Building Blocks for Higher Supramolecular Assemblies. *Eur. J. Org. Chem*. **2013**, *2013*, 283−299.

(8) (a) Neese, F. The ORCA program system. *Wiley Interdiscip. Rev.: Comput. Mol. Sci*. **2012**, *2*, 73–78. (b) Neese, F. Software update: The ORCA program system—Version 5.0. *Wiley Interdiscip. Rev.: Comput. Mol. Sci.* **2022**, *12*, e1606. (c) Neese, F. Software update: The ORCA program system—Version 6.0. *Wiley Interdiscip. Rev.: Comput. Mol. Sci.* **2025**, *15*, e70019.

(9) Adamo, C.; Barone, V. Toward reliable density functional methods without adjustable parameters: The PBE0 model. *J. Chem. Phys.* **1999**, *110*, 6158–6170.

(10) Weigend, F.; Ahlrichs, R. Balanced basis sets of split valence, triple zeta valence and quadruple zeta valence quality for H to Rn: Design and assessment of accuracy. *Phys. Chem. Chem. Phys.* **2005**, *7*, 3297–3305.

(11) Marenich, A. V.; Cramer, C. J.; Truhlar, D. G. Universal solvation model based on solute electron density and on a continuum model of the solvent defined by the bulk dielectric constant and atomic surface tensions. *J. Phys. Chem. B* **2009**, *113*, 6378–6396.

(12) Barone, V.; Cossi, M. Quantum Calculation of Molecular Energies and Energy Gradients in Solution by a Conductor Solvent Model. *J. Phys. Chem. A* **1998**, *102*, 1995– 2001.

(13) (a) Petrenko, T.; Kossmann, S.; Neese, F. Efficient time-dependent density functional theory approximations for hybrid density functionals: analytical gradients and parallelization. *J. Chem. Phys*. **2011**, *134*, 54116. (b) Neese, F.; Olbrich, G. Efficient use of the resolution of the identity approximation in time-dependent density functional calculations with hybrid density functionals. *Chem. Phys. Lett*. **2002**, *362*, 170–178. (c) O. Vahtras; J. Almlöf; M.W. Feyereisen. Integral approximations for LCAO-SCF calculations. *Chem. Phys. Lett*. **1993**, *213*, 514–518. (d) Neese, F.; Wennmohs, F.; Hansen, A.; Becker, U. Efficient, approximate and parallel Hartree–Fock and hybrid DFT calculations. A 'chain-of-spheres' algorithm for the Hartree– Fock exchange. *Chem. Phys*. **2009**, *356*, 98–109.

(14) (a) Eichkorn, K.; Treutler, O.; Öhm, H.; Häser, M.; Ahlrichs, R. Auxiliary basis sets to approximate Coulomb potentials (Chem. Phys. Letters 240 (1995) 283-290). *Chem. Phys. Lett*. **1995**, *242*, 652–660. (b) Eichkorn, K.; Weigend, F.; Treutler, O.; Ahlrichs, R. Auxiliary basis sets for main row atoms and transition metals and their use to approximate Coulomb potentials. *Theor. Chem. Acc*. **1997**, *97*, 119–124. (c) Weigend, F. Accurate Coulomb-fitting basis sets for H to Rn. *Phys. Chem. Chem. Phys*. **2006**, *8*, 1057–1065.

(15) Grimme, S.; Antony, J.; Ehrlich, S.; Krieg, H. A consistent and accurate ab initio parametrization of density functional dispersion correction (DFT-D) for the 94 elements H-Pu. *J. Chem. Phys*. **2010**, *132*, 154104.

(16) *Chemcraft - graphical software for visualization of quantum chemistry computations. https://www.chemcraftprog.com.*

(17) (a) Maiti, A.; Chandra, S.; Sarkar, B.; Jana, A. Acyclic Diaminocarbene-Based Thiele, Chichibabin, and Müller Hydrocarbons. *Chem. Sci*. **2020**, *11*, 11827– 11833. (b) Pei, Z.; Magann, N. L.; Sowden, M. J.; Murphy, R. B.; Gardiner, M. G.; Sherburn, M. S.; Coote, M. L. Computational and Experimental Confirmation of the Diradical Character of para-Quinonedimethide. *J. Am. Chem. Soc*. **2023**, *145*, 16037– 16044.
